# FGFR inhibitors promote the autophagic degradation of IFN-γ-induced PD-L1 and alleviate the PD-L1-mediated transcriptional suppression of FGFR3-TACC3 in non-muscle-invasive bladder cancer

**DOI:** 10.1038/s41419-025-07821-8

**Published:** 2025-07-02

**Authors:** Yu-Chen Lin, Cheng-Ying Chu, Tsung-Han Hsieh, Bo-Jyun Lin, Jing-Ping Liou, Yun Yen, Chun-Han Chen

**Affiliations:** 1 Graduate Institute of Medical Sciences, College of Medicine, Taipei Medical University, Taipei, Taiwan; 2 Department of Pharmacology, School of Medicine, College of Medicine, Taipei Medical University, Taipei, Taiwan; 3 CRISPR Gene Targeting Core, Taipei Medical University, Taipei, Taiwan; 4 TMU Research Center of Cancer Translational Medicine, Taipei Medical University, Taipei, Taiwan; 5 Precision Health Center, Taipei Medical University, Taipei, Taiwan; 6 School of Pharmacy, College of Pharmacy, Taipei Medical University, Taipei, Taiwan; 7 Ph.D. Program for Cancer Biology and Drug Discovery, College of Medical Science and Technology, Taipei Medical University, Taipei, Taiwan; 8 Center for Cancer Translational Research, Tzu Chi University, Hualien, Taiwan; 9 Cell Physiology and Molecular Image Research Center, Wan Fang Hospital, Taipei Medical University, Taipei, Taiwan

**Keywords:** Bladder cancer, Macroautophagy, Immune evasion, Lysosomes

## Abstract

Bladder cancer (BC) is the second most prevalent genitourinary malignancy worldwide. Treatment options remain limited for patients with Bacillus Calmette–Guérin (BCG)-unresponsive non-muscle-invasive bladder cancer (NMIBC). Up to 70% of NMIBC cases harbor fibroblast growth factor receptor 3 (FGFR3) alterations, and FGFR inhibition has shown potential to enhance the efficacy of immune checkpoint inhibitor (ICI). Interferon (IFN)-γ, a cytokine produced by activated T cells and associated with better response to immunotherapy in BC, is a key inducer of PD-L1 expression in the tumor microenvironment. However, the interaction between FGFR inhibitors and IFN-γ-induced PD-L1 expression in FGFR3-activated NMIBC cells remains unclear. Here, we show that FGFR inhibitors significantly reduced IFN-γ-induced PD-L1 expression in NMIBC cells harboring *FGFR3-TACC3* fusions. Mechanistically, FGFR inhibitors restored IFN-γ-suppressed SIRT1 expression, promoted LC3B deacetylation and nuclear export, and enhanced autophagy-lysosomal degradation of PD-L1. Blocking autophagy, overexpression SIGMAR1, or inhibiting lysosomal activity significantly reversed PD-L1 degradation. Notably, we demonstrate for the first time that IFN-γ-induced PD-L1 directly binds to the FGFR3 promoter and represses *FGFR3-TACC3* transcription–an effect that can be rescued by FGFR inhibitors or PD-L1 knockdown. Functionally, FGFR inhibitors ameliorated PD1/PD-L1-mediated T cell suppression in co-culture assays. Together, these findings highlight a novel mechanism by which FGFR inhibitors suppress IFN-γ-induced PD-L1 via autophagy and suggest a potential strategy to improve ICI therapy in FGFR3-altered NMIBC.

## Introduction

Bladder cancer (BC) is the second most prevalent genitourinary malignancy worldwide, with the latest statistics reporting approximately 613,791 new cases and 220,349 deaths [1]. Although conventional treatments such as surgery, chemotherapy, and radiation therapy have provided incremental improvements in patient outcomes, their efficacy remains limited, particularly in advanced or metastatic disease [2–4]. Furthermore, the heterogeneity of BC, characterized by distinct molecular subtypes with varying genetic alterations, poses an enormous challenge to the development of targeted therapies [5]. Approximately 75% of patients with BC are diagnosed with non–muscle-invasive BC (NMIBC); transurethral resection of bladder tumors (TURBT) combined with intravesical Bacillus Calmette–Guérin (BCG) vaccination is the standard treatment for these patients [6]. However, approximately 20–30% of patients experience recurrence within the first year, which might progress to muscle-invasive or metastatic disease, with lifestyle-altering radical cystectomy being the most common therapy [7]. Recently, pembrolizumab has been approved for the treatment of high-risk patients with NMIBC who are BCG-unresponsive, and ineligible for or have declined to undergo cystectomy. However, 59% of patients did not respond to pembrolizumab, and 51% of the initial responders had recurrent disease [8]. Hence, there is a significant need for effective strategies to enhance the efficacy of immunotherapy in patients with NMIBC.

Genetic alternations in FGFR3, including activating mutations and gene fusions, have been observed in 70% of patients with NMIBC [9]. According to their molecular characteristics, FGFR3 alternations are enriched in the UROMOL class 1 and class 3 NMIBC subtypes, which resemble the consensus luminal papillary subtype in patients with muscle-invasive bladder cancer (MIBC) [10]. However, erdafitinib, the only FGFR inhibitor approved for patients with locally advanced or metastatic MIBC, is currently unsuitable for NMIBC treatment [11]. Despite the association of FGFR3 alternations with a non-T cell-inflamed tumor microenvironment and the reduced sensitivity to immune checkpoint inhibitors (ICIs) [12, 13], recent studies have indicated that patients with BC respond equally to ICIs regardless of FGFR3 status [14, 15]. A preclinical study showed that FGFR inhibitors increased anti-PD-1 treatment efficacy in an FGFR3 mutant-driven murine high-grade NMIBC model by abrogating ICI-induced regulatory T cell (Treg) expansion in the tumor microenvironment [9]. Notably, *FGFR3-TACC3* gene fusions represent a distinct and clinically actionable oncogenic driver in BC. Compared to canonical FGFR3 mutations, BC cell lines harboring FGFR3-TACC3 fusions are much more sensitive to FGFR inhibitors than those with FGFR3 mutation, likely due to ligand-independent dimerization and constitutive activation of oncogenic signaling [16, 17]. Additionally, FGFR3-TACC3 fusion causes mitotic defect by removing endogenous TACC3 from the mitotic spindle, potentially promoting aneuploidy and tumor progression in BC [18]. Clinically, this fusion is enriched in young Asian patients with BC who have never smoked, and is correlated with higher mRNA expression levels compared to FGFR3-intact BC patients [19, 20]. A patient with metastatic BC with *FGFR3-TACC3* fusion showed a remarkable response to sequential treatment with FGFR3 inhibition and anti-PD-L1 blockade, underscoring the potential synergy between FGFR inhibitors and ICIs in this molecular subgroup [21]. Collectively, these findings highlight the unique functional and therapeutic relevance of *FGFR3-TACC3* fusions in BC.

As a vital cytokine produced by tumor-infiltrating lymphocytes, interferon (IFN)-γ promotes immunosurveillance by modulating the tumor microenvironment, promoting antigen presentation, and boosting the activity of cytotoxic T cells [22]. Controversially, IFN-γ can also upregulate PD-L1 on tumor cells and diminish the effectiveness of antitumor immunity by interacting with PD-1 on tumor-infiltrating T cells [23]. Recently, an IFN-γ^+^ gene signature (*IFNG*, *CD274*, *LAG3*, and *CXCL9*) was reported to be associated with improved clinical outcomes of durvalumab treatment in patients with BC, highlighting the crucial role of IFN-γ in successful ICI treatment for BC [24]. Interestingly, FGFR3 has been shown to suppress IFN-γ functions in BC cells, as the inhibition of the FGFR3 pathway reactivates downstream targets of IFN-γ (B2M, CXCL10, and PD-L1) [25, 26]. FGFR3 reportedly phosphorylates and destabilizes PD-L1 via NEDD4-dependent proteasomal degradation in FGFR3-activated BC cells. Moreover, FGFR3 inhibition elevates PD-L1 protein levels, inhibiting the antitumor activity of CD8^+^ T cells [27]. Given that IFN-γ is crucial to the anti-tumor immunity in microenvironment, the association between FGFR3 and PD-L1 levels in the presence of IFN-γ is largely underexplored in BC, prompting us to evaluate the effect of FGFR inhibitors on IFN-γ-induced PD-L1 and its molecular mechanisms in FGFR3-activated NMIBC cells.

In this study, we report that FGFR inhibitors significantly suppress IFN-γ-induced PD-L1 by promoting its autophagy-lysosomal degradation in NMIBC cells harboring *FGFR3-TACC3* fusions. For the first time, we revealed that IFN-γ-induced PD-L1 directly binds to the *FGFR3* promoter and suppresses the transcription of *FGFR3-TACC3*, which could be reversed by FGFR inhibitors. Moreover, FGFR inhibitors ameliorated PD1/PD-L1-mediated T cell suppression in an RT-112/Jurkat-hPD1 co-culture assay. These findings highlight the potential benefits of combining FGFR inhibitors with ICIs in patients with NMIBC harboring FGFR3 alterations.

## Results

### Luminal-type BC cells harboring *FGFR3-TACC3* gene fusions have lower basal PD-L1 levels

**Fig. 1:**
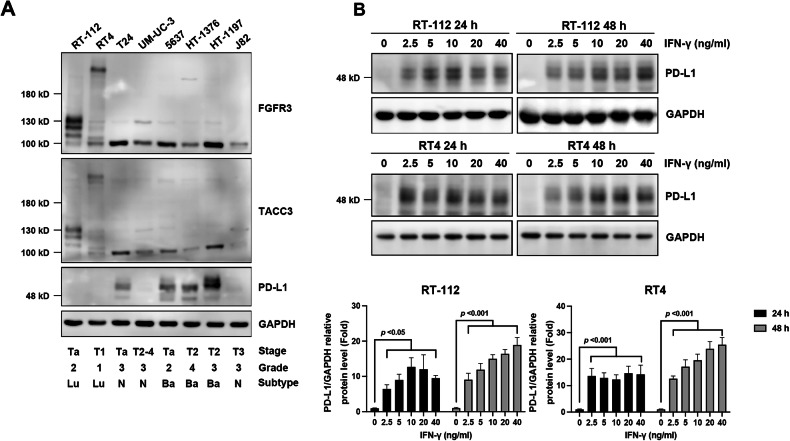
Luminal-type bladder cancer (BC) cells harboring *FGFR3-TACC3* gene fusions have lower basal levels of PD-L1. **A** Basal levels of FGFR3, TACC3, and PD-L1 in different BC cell lines were analyzed via western blotting. Lu, luminal-type; Ba, basal-type; N, none-type. **B** Interferon (IFN)-γ concentration-dependently induced PD-L1 expression in RT-112 and RT4 cells. Briefly, cells were treated with vehicle control or the indicated concentrations of IFN-γ for 24 or 48 h and subjected to western blotting. The band intensities of each protein were quantified using ImageJ software and normalized to that of GAPDH. Fold changes compared to the control (CTRL) group are expressed as the means ± standard deviations (*n* = 3). One-way ANOVA with Dunnett’s multiple comparison was used to evaluate statistical significance.

We firstly examined the basal levels of PD-L1 in various BC cell lines [5]; RT-112 and RT4 cells harbored the *FGFR3-TACC3* gene fusion. Interestingly, basal-type BC cells (5637, HT1376, and HT1197) expressed relatively high PD-L1 levels. In contrast, luminal-type BC cells (RT-112 and RT4) have the lowest basal PD-L1 expression levels (Fig. 1A). *FGFR3-TACC3* fusions are generated by intrachromosomal rearrangements of FGFR3 on chromosome 4p16, which lead to the loss of an miR-99a binding site within the 3′-untranslated region of FGFR3 and enhance tumor progression compared to wild-type FGFR3 [28, 29]. Specifically, RT-112 and RT4 cells express higher levels of *FGFR3-TACC3* fusion genes than wild-type *FGFR3* (Supplementary Fig. S1A, B). Because FGFR3 activation is a key molecular feature in patients with luminal-type BC who are suitable for treatment with FGFR inhibitors [10], RT-112 and RT4 cells were selected for subsequent experiments. Our data demonstrated that treatment with IFN-γ led to a concentration-dependent increase in PD-L1 expression in RT-112 and RT4 cells (Fig. 1B). Specifically, 10 ng/ml of IFN-γ was selected as the optimal concentration to induce PD-L1 expression with minimal cytotoxicity in BC cells (data not shown).

### FGFR inhibitors decrease IFN-γ-induced PD-L1 expression in luminal BC cells

**Fig. 2:**
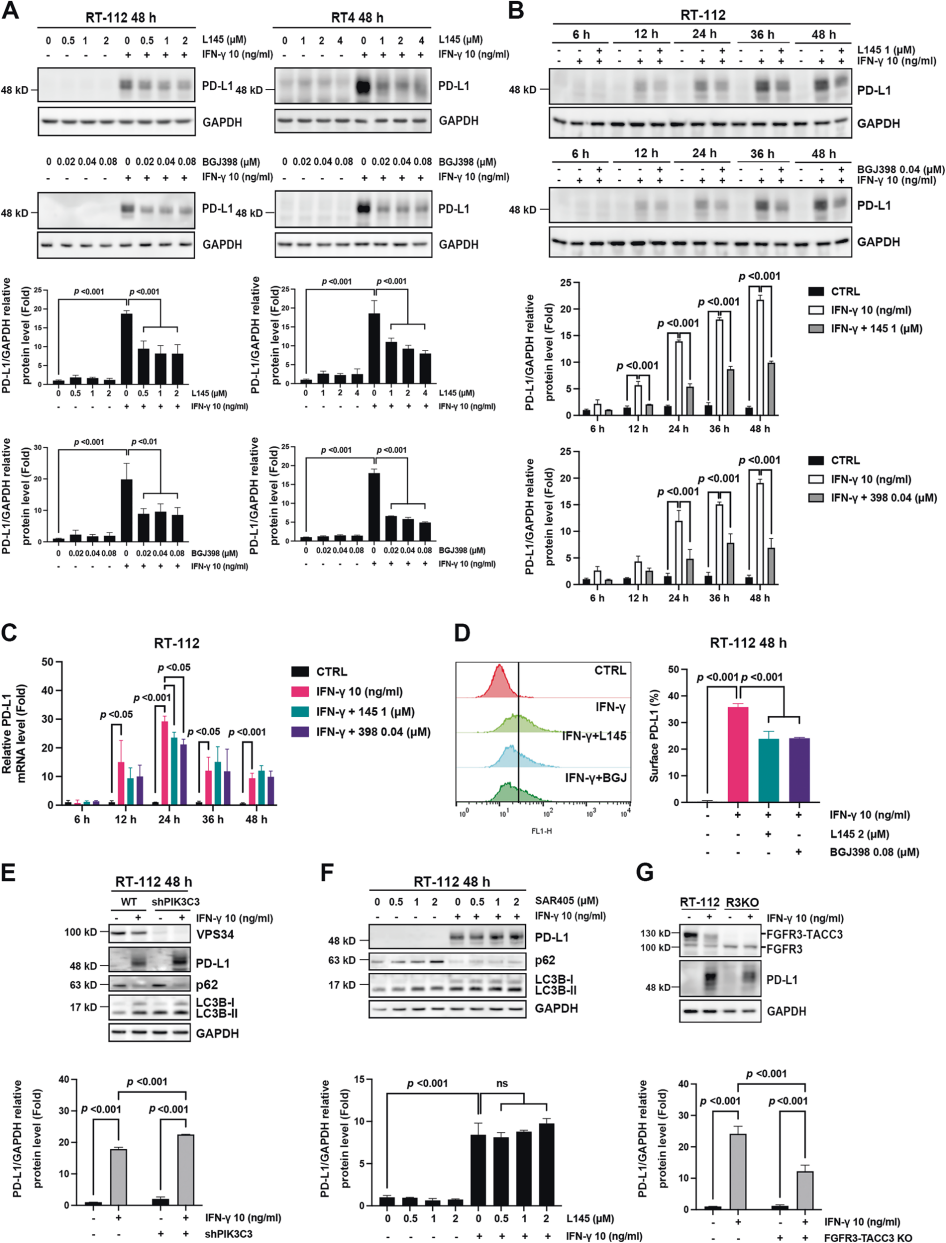
FGFR inhibitors decrease IFN-γ-induced PD-L1 expression in luminal bladder cancer cells. **A** RT-112 and RT4 cells were treated with vehicle control or the indicated concentrations of MPT0L145 (L145) or BGJ398 in the presence or absence of interferon (IFN)-γ (10 ng/ml) for 48 h and subjected to western blot analysis. **B**, **C** RT-112 cells were treated with vehicle control (CTRL) or IFN-γ (10 ng/ml) with or without L145 (1 μM) or BGJ398 (0.04 μM) for the indicated times, and subjected to western blot (**B**) and (**C**) RT-qPCR analyses. The band intensities of each protein were quantified using ImageJ software and normalized to that of GAPDH. Fold changes compared to the control group are expressed as the means ± standard deviations (*n* = 3). **D** RT-112 cells were exposed to IFN-γ (10 ng/ml) with or without L145 (2 μM) or BGJ398 (0.08 μM) for 48 h, stained with αPD-L1 antibody, and subjected to flow cytometry analysis. Data are expressed as the mean ± standard deviation (*n* = 3). **E** Wild-type (WT) or PIK3C3 (VPS34)-knockdown RT-112 cells were treated with vehicle control or IFN-γ (10 ng/ml) for 48 h and subjected to western blot analysis. The band intensities of each protein were quantified using ImageJ software and normalized to that of GAPDH. Fold changes compared to the control group are expressed as the means ± standard deviations (*n* = 3). **F** RT-112 cells were treated with various concentrations of SAR405 in the presence or absence of IFN-γ (10 ng/ml) for 48 h, and the protein lysates were analyzed via western blotting. The band intensities of each protein were quantified using ImageJ software and normalized to that of GAPDH. Fold changes compared to the control group are expressed as the means ± standard deviations (*n* = 3). **G** Wild-type (WT) or *FGFR3-TACC3*-knockout RT112 cells were exposed to vehicle control (CTRL) or IFN-γ (10 ng/ml) for 48 h and subjected to western blot analysis. The band intensities of each protein were quantified using ImageJ software and normalized to that of GAPDH. Data are expressed as the mean ± standard deviation (*n* = 3). Statistical analyses were performed by one-way ANOVA with Tukey’s multiple comparison for data in (**A**–**D**, **F**); two-way ANOVA with Tukey’s multiple comparison was used for data in (**E**, **G**). For time-course experiments (**B**, **C**), the comparisons were performed independently at each time point.

We further investigated the effects of FGFR inhibitors on IFN-γ-induced PD-L1 expression in RT-112 and RT4 cells. MPT0L145 (L145), a dual FGFR/VPS34 inhibitor discovered in our laboratory [30], and BGJ398 (infigratinib), a selective FGFR1-FGFR3 inhibitor currently undergoing clinical trials [31], were employed in this study. Concentrations below the IC_50_ of these drugs were selected for subsequent studies in RT-112 and RT4 cells (Supplementary Fig. S1C). The data showed that L145 and BGJ398 suppressed the protein levels of IFN-γ-induced PD-L1 in RT-112 and RT4 cells in a concentration-dependent manner (Fig. 2A). This result was also confirmed when the cells were treated with erdafitinib, which is clinically used in adults with locally advanced or metastatic urothelial carcinoma with susceptible FGFR3 genetic alterations (Supplementary Fig. S1D) [32]. Time-course analysis revealed that L145 and BGJ398 remarkably decreased PD-L1 expression as early as 12 h of treatment; the inhibitory effect persisted for 48 h in RT-112 cells (Fig. 2B). Furthermore, L145 and BGJ398 marginally decreased the mRNA levels of *PD-L1* at 12 and 24 h of treatment; however, this effect was not sustained at 36 and 48 h (Fig. 2C). PD-L1 on the membranes of cancer cells functionally interacts with PD-1 on the surface of immune cells, leading to the inhibitory signals to T cells, suppressing their activity [33]. The data revealed that L145 and BGJ398 considerably decreased IFN-γ induced PD-L1 expression on the cell surface of RT-112 cells (Fig. 2D).

Since L145 is a dual inhibitor of both FGFRs and VPS34 [30], we further examined the contribution of VPS34 inhibition to PD-L1 downregulation. VPS34 knockdown using lentivirus-delivered shRNA against *PIK3C3* gene (Fig. 2E) or VPS34-specific inhibitors (SAR405) (Fig. 2F) did not decrease IFN-γ-induced PD-L1 expression in RT-112 cells, excluding the possible inhibitory effect of L145 on VPS34. Meanwhile, IFN-γ had a weak effect on PD-L1 induction in RT-112-*FGFR3-TACC3*-knockout cells [34], suggesting that FGFR3-TACC3 signaling is crucial to IFN-γ-induced PD-L1 (Fig. 2G). Collectively, our findings demonstrate that FGFR inhibitors significantly suppress IFN-γ-induced PD-L1 expression in luminal BC cells.

### FGFR inhibitors promote the autophagic degradation of IFN-γ-induced PD-L1 in luminal BC cells

**Fig. 3:**
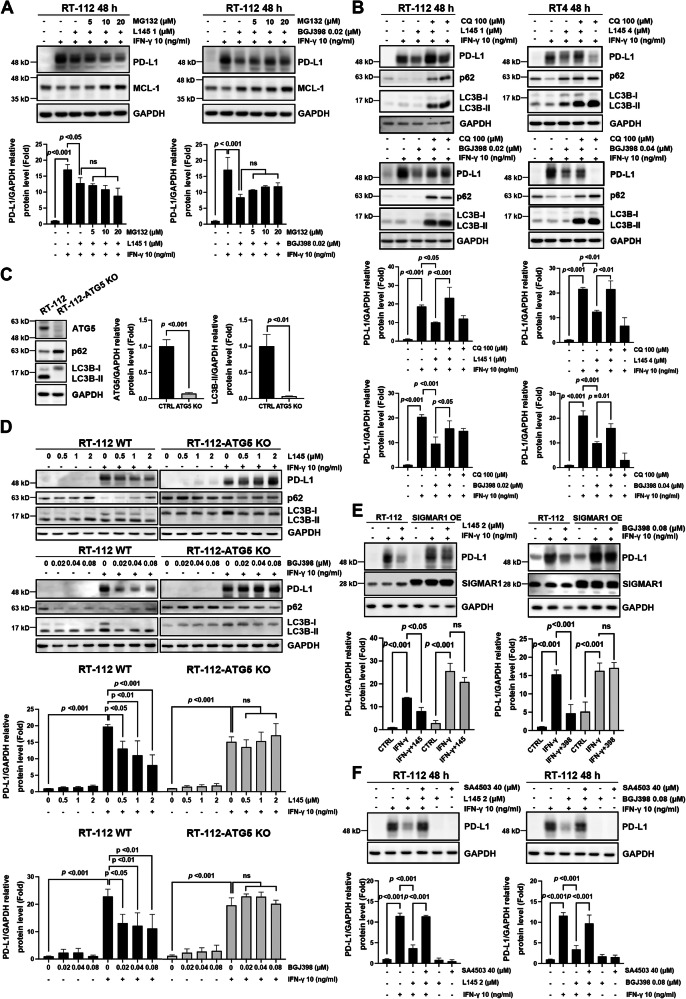
Autophagy is involved in FGFR inhibitor-mediated PD-L1 downregulation. **A** RT-112 cells were treated with interferon (IFN)-γ (10 ng/ml) alone or in combination with MPT0L145 (L145, left) or BGJ398 (right) in the presence or absence of MG132 for 48 h and subjected to western blot analysis. MCL-1 protein served as a positive control for proteasomal degradation. The band intensities of each protein were quantified using ImageJ software and normalized to that of GAPDH. Fold changes compared to the control group are expressed as the means ± standard deviations (*n* = 3). **B** RT-112 and RT4 cells were treated with IFN-γ (10 ng/ml) alone or in combination with the indicated concentrations of MPT0L145 (L145) or BGJ398 in the presence or absence of chloroquine (CQ, 100 μM) for 48 h and subjected to western blotting. The band intensities of each protein were quantified using ImageJ software and normalized to that of GAPDH. Fold changes compared to the control group are expressed as the mean ± standard deviation (n = 3). **C**, **D** ATG5 knockdown reversed the FGFR inhibitor-suppressed PD-L1 expression. **C** Protein lysates from RT-112 and RT-112-ATG5KO cells were analyzed via western blotting to confirm the blockade of LC3-dependent autophagy. Fold changes compared to RT-112 cells are expressed as the means ± standard deviations (*n* = 3). **D** RT-112 and RT-112-ATG5KO cells were treated with the indicated concentrations of L145 or BGJ398 in the presence or absence of IFN-γ for 48 h and subjected to western blot analysis. The band intensities of each protein were quantified using ImageJ software and normalized to that of GAPDH. Fold changes compared to the control group are expressed as the mean ± standard deviation (n = 3-4). **E** Overexpression of SIGMAR1 rescued FGFR inhibitor-mediated PD-L1 suppression. Briefly, RT-112 cells were transiently transfected with SIGMAR1-expressing plasmids, treated with IFN-γ in the presence or absence of L145 (2 μM) or BGJ398 (0.08 μM) for 48 h, and subjected to western blot analysis. **F** RT-112 cells were treated with IFN-γ (10 ng/ml) alone or combined with MPT0L145 (L145, *left*) or BGJ398 (right) in the presence or absence of SA4503 (40 μM) for 48 h and subjected to western blot analysis. The band intensities of each protein were quantified using ImageJ software and normalized to that of GAPDH. Fold changes compared to the control (CTRL) group are expressed as the means ± standard deviations (*n* = 3). Statistical analyses were performed by one-way ANOVA with Tukey’s multiple comparison for data in (**A**, **B**, **D**–**F**); unpaired two-tailed Student’s *t*-test was used for data in (**C**).

The abovementioned data showed that FGFR inhibitors only had marginal effects on the transcription of PD-L1 before 24 h, but their inhibitory activity on protein levels continued for 48 h (Fig. 2B, C). Next, we focused on the effect of FGFR inhibitors on the post-translational regulation of IFN-γ-induced PD-L1. Previous studies have suggested that the proteasome and autophagy-lysosomal pathways contribute to the protein degradation of PD-L1 [35]. First, we found that the combination of a proteasome inhibitor (MG132) with IFN-γ and FGFR inhibitors at concentrations that increase the levels of known proteasome substrate MCL-1 had no rescue effect on FGFR inhibitor-suppressed PD-L1, excluding the possibility of proteasomal degradation (Fig. 3A). Importantly, treatment with the lysosome inhibitor chloroquine significantly reversed the FGFR inhibitor-mediated degradation of PD-L1 in RT-112 and RT4 cells (Fig. 3B, and Supplementary Fig. S1E). Further, we knocked out *ATG5* in RT-112 cells using CRISPR/Cas9 [30], in which autophagy was compromised, as evidenced by the lack of LC3B-II conversion and elevated cargo protein p62 expression (Fig. 3C). The results demonstrated that the suppressive effects of L145 and BGJ398 on IFN-γ-induced PD-L1 expression were completely abrogated in *ATG5*-knockout RT-112 cells (Fig. 3D). SIGMAR1 (sigma-1 receptor), a ligand-regulated integral membrane scaffolding protein, maintains the stability of PD-L1 by preventing its autophagic degradation [36]. Notably, either overexpression of SIGMAR1 (Fig. 3E) or treatment with SA4503, a SIGMAR1 activator (Fig. 3F), significantly prevented the degradation of IFN-γ-induced PD-L1 mediated by L145 and BGJ398 in RT-112 cells. In addition, SIGMAR1 overexpression profoundly abrogated the colocalization of PD-L1 and LC3B following combined treatment with IFN-γ and FGFR inhibitors (Supplementary Fig. S2A). Together, our findings demonstrate that FGFR inhibitors promote the autophagy-lysosomal degradation of IFN-γ-induced PD-L1 in luminal BC cells.

### IFN-γ blocks the nuclear export of LC3B by decreasing SIRT1-mediated lysine deacetylation

**Fig. 4:**
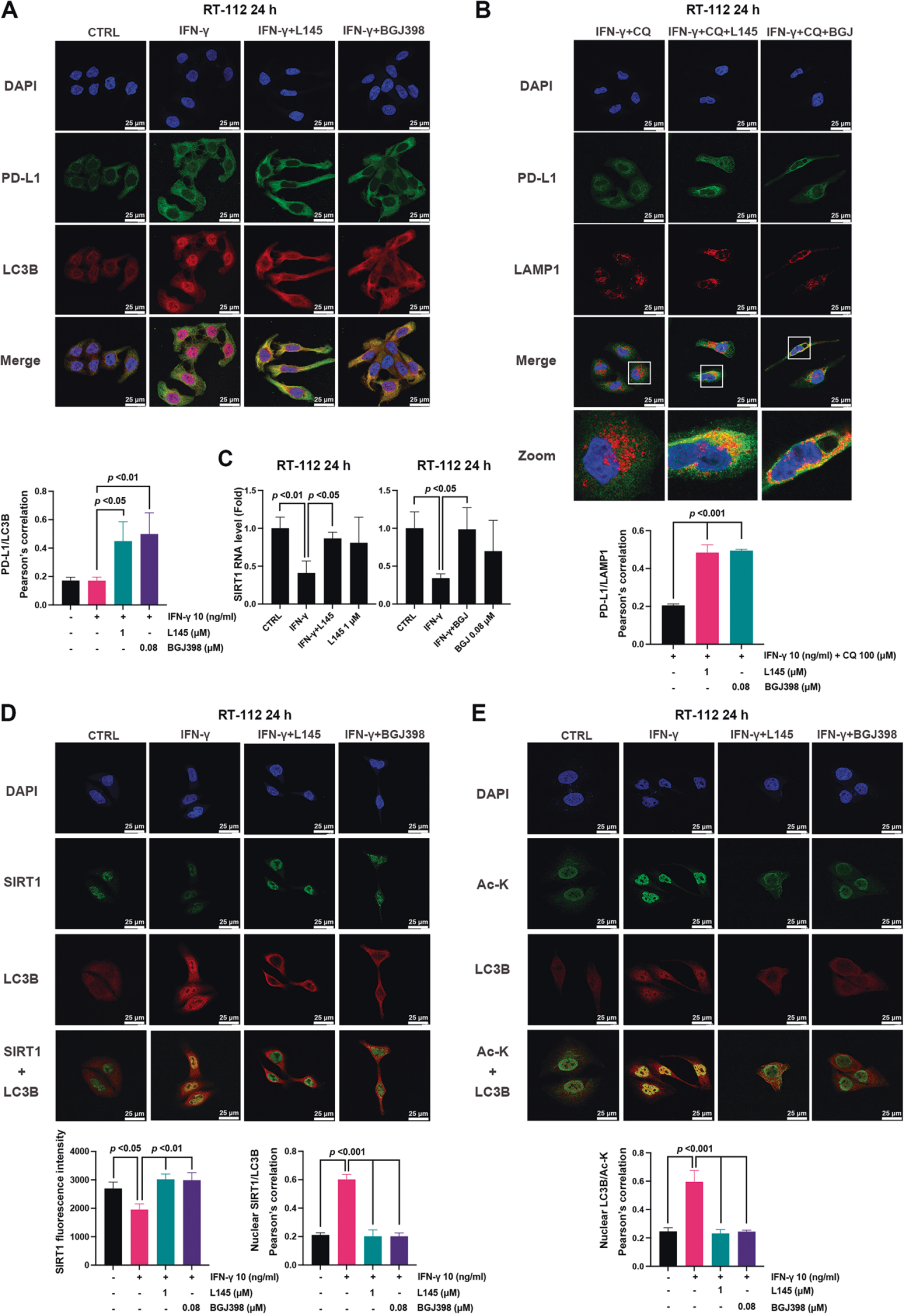
IFN-γ blocks the nuclear export of LC3B by decreasing SIRT1-mediated lysine deacetylation. **A** RT-112 cells were treated with interferon (IFN)-γ (10 ng/ml) alone or in combination with L145 (1 μM) or BGJ398 (0.08 μM) for 24 h and subjected to immunofluorescence staining with antibodies against PD-L1 (green) and LC3B (red). Images were acquired using confocal microscopy and Pearson’s correlation is presented as the mean ± standard (n = 4). **B** RT-112 cells treated with IFN-γ combined with chloroquine (CQ, 100 μM) in the presence of L145 (1 μM) or BGJ398 (0.08 μM) were stained with anti-PD-L1 (green) and anti-LAMP1 (red) antibodies and evaluated using confocal microscopy. Pearson’s correlation are presented as the mean ± standard deviation (n = 3). **C** RT-112 cells were treated with IFN-γ (10 ng/ml) alone or in combination with L145 (1 μM) or BGJ398 (0.08 μM) for 24 h and subjected to RT-qPCR analysis. Data are expressed as the mean ± standard deviation (n = 4). **D**, **E** RT-112 cells were treated with IFN-γ (10 ng/ml) alone or in combination with L145 (1 μM) or BGJ398 (0.08 μM) for 24 h, and subjected to immunofluorescence staining with antibodies against (**C**) SIRT1 (green) and LC3B (red) or (**D**) acetyl-lysine (Ac-K, green) and LC3B (red). Fluorescence intensity or Pearson’s correlation are presented as the means ± standard deviations (*n* = 3). One-way ANOVA with Tukey’s multiple comparison was used for statistical analysis.

To further elucidate the mechanism underlying the FGFR inhibitor-mediated autophagy-lysosomal degradation of IFN-γ-induced PD-L1, we used confocal microscopy to examine the localization of PD-L1 and LC3B in RT-112 cells. The data revealed that the majority of LC3B is localized in the nucleus, and PD-L1 is present surrounding the nuclear area in the presence of IFN-γ (Fig. 4A). Treatment with L145 or BGJ398 notably induced the nuclear export of LC3B and enhanced its colocalization with PD-L1 in the cytosol (Fig. 4A). Meanwhile, we observed that FGFR inhibitors significantly increased the colocalization of PD-L1 and LAMP1, a lysosomal marker, further confirming the lysosomal degradation of PD-L1 in RT-112 cells (Fig. 4B). A previous study indicated that nuclear export of LC3B is regulated by SIRT1-mediated deacetylation to facilitate autophagy initiation [37]. IFN-γ reportedly suppresses *SIRT1* transcription during chronic inflammation [38]. RT-qPCR analysis revealed that the mRNA expression of *SIRT1* decreased following IFN-γ treatment and that this was reversed upon treatment with L145 or BGJ398 (Fig. 4C). Confocal microscopy revealed that IFN-γ significantly decreased nuclear SIRT1 expression and increased its colocalization with LC3B in nucleus, which could be significantly reversed by L145 or BGJ398 treatment (Fig. 4D). Meanwhile, IFN-γ significantly increased the colocalization of acetyl-lysine and LC3B in the nucleus, suggesting increased levels of acetylated LC3B; this was abrogated by L145 or BGJ398 treatment in RT-112 cells (Fig. 4E). We then knocked down *SIRT1* using siRNA to confirm whether the rescue effects of FGFR inhibitors were mediated by SIRT1-dependent deacetylation and the nuclear export of LC3B. SIRT1 knockdown retained LC3B in the nucleus (Supplementary Fig. S2B) and its nuclear colocalization with acetyl-lysine (Supplementary Fig. S2C). Collectively, these findings revealed that IFN-γ ameliorates the nuclear export of LC3B by downregulating SIRT1-mediated deacetylation. FGFR inhibitors counteract this effect, promoting LC3B export and the autophagic degradation of PD-L1 in luminal-type BC cells.

### FGFR inhibitors abrogate PD1/PD-L1-mediated T cell suppression

So far, we found that FGFR inhibitors abrogate IFN-γ-induced PD-L1 expression in luminal BC cells. To confirm whether PD-L1 downregulation plays a physiological role in modulating anticancer immunity, we established a co-culture assay to examine T cell activity. Jurkat-hPD-1 cells stably expressing T cell receptor (TCR), CD28 stimulatory receptor, human PD-L1, and an NFAT-inducible Lucia luciferase reporter gene were used (Supplementary Fig. S3A). Upon stimulation with anti-CD3 and anti-CD28 antibodies, NFAT activation increased luciferase activity in Jurkat-hPD-1 cells. Next, we pre-treated RT-112 cells with IFN-γ to induce PD-L1 expression for 48 h and then co-cultured them with Jurkat-hPD-1 cells for 24 h in the presence of anti-CD3 and anti-CD28 antibodies. Luciferase activity decreased after co-incubation, suggesting that the interaction between PD-1 and PD-L1 suppressed T cell activation. However, luciferase activity was rescued when RT-112 cells were pre-treated with IFN-γ in the presence of FGFR inhibitors (Supplementary Fig. S3B). These data suggest that FGFR inhibitors reactivate T cell activity by ameliorating PD-1/PD-L1-mediated suppression.

### IFN-γ-induced PD-L1 suppresses *FGFR3-TACC3* gene transcription in luminal BC cells

**Fig. 5:**
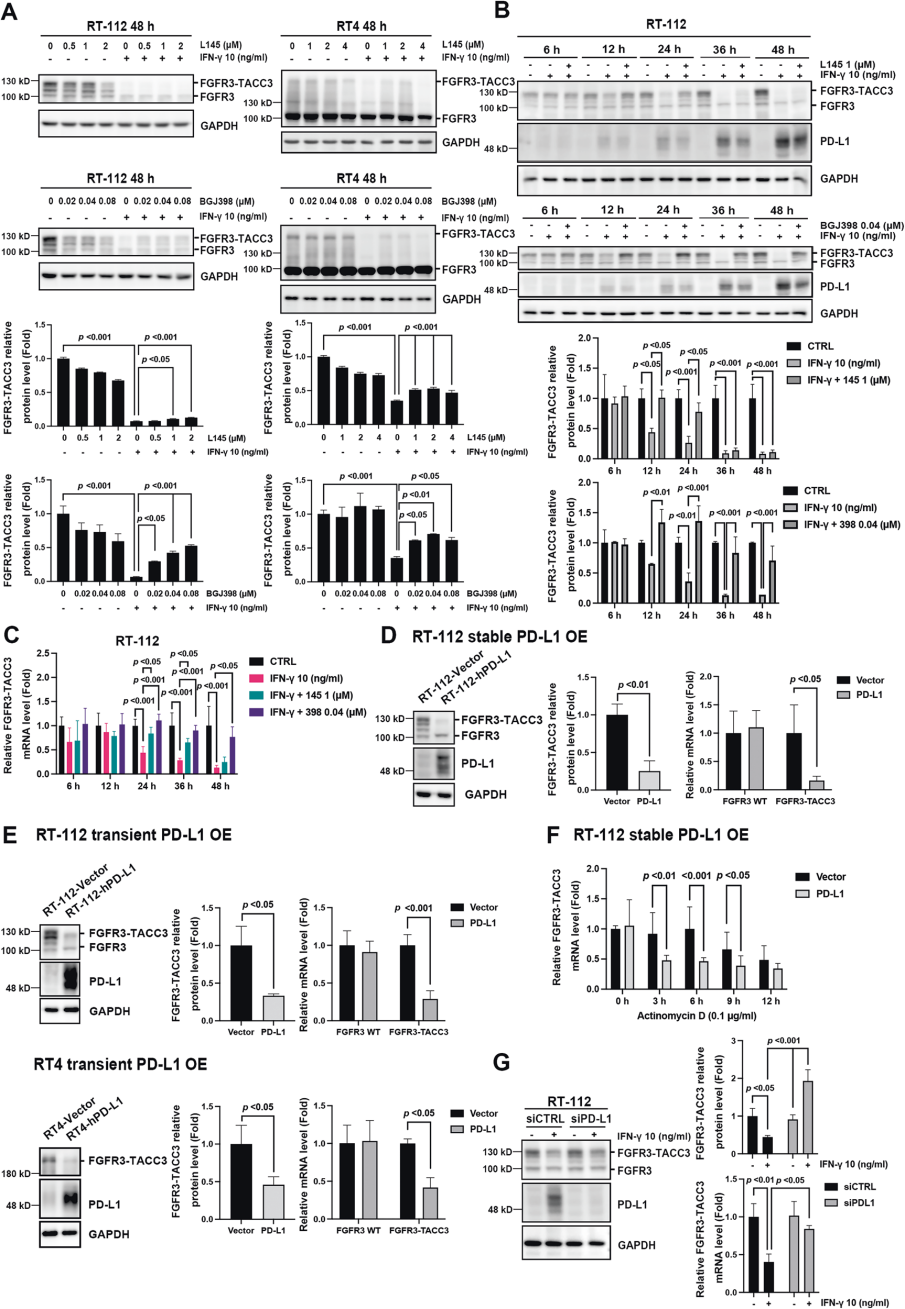
IFN-γ downregulates *FGFR3-TACC3* transcription in luminal bladder cancer cells. **A**–**C** Interferon (IFN)-γ concentration- and time-dependently downregulated FGFR3-TACC3 expression. **A** RT-112 and RT4 cells were treated with vehicle control (CTRL) or the indicated concentrations of MPT0L145 (L145) or BGJ398 in the presence or absence of IFN-γ (10 ng/ml) for 48 h and subjected to western blot analysis. **B**, **C** RT-112 cells were treated with vehicle control (CTRL) or IFN-γ (10 ng/ml) with or without L145 (1 μM) or BGJ398 (0.04 μM) for the indicated times and subjected to western blot (**B**) and (**C**) RT-qPCR analysis. The band intensities of each protein were quantified using ImageJ software and normalized to that of GAPDH. Fold changes compared to the control group are expressed as the means ± standard deviations (*n* = 3). One-way ANOVA with Tukey’s multiple comparison was used for statistical analysis in (**A**–**C**). For time-course experiments (**B**, **C**), the comparisons were performed independently at each time point. **D**–**G** Enforced expression of PD-L1 suppressed the transcription of the *FGFR3-TACC3* gene. Briefly, RT-112 and RT4 cells were stably (**D**) or transiently (**E**) transfected with a control vector or PD-L1 expression plasmid and subjected to western blotting and RT-qPCR analysis. Fold changes in FGFR3-TACC3 levels compared to the vector group are expressed as the means ± standard deviations (*n* = 3). Statistical analyses in (**D**–**E**) were performed by unpaired two-tailed Student’s *t*-test for western blot; two-way ANOVA with Sidak’s multiple comparison for qPCR analysis. **F** RT-112 cells stably transfected with a control vector or PD-L1 expression plasmid were exposed to actinomycin D to block transcription at the indicated times and subjected to RT-qPCR. Fold changes in FGFR3-TACC3 levels compared to the vector group are expressed as the mean ± standard deviation (n = 3). Two-way ANOVA with Sidak’s multiple comparison was used for statistical analysis. **G** RT-112 cells were transiently transfected with control siRNA (siCTRL) or siPD-L1, treated with vehicle control (CTRL) or IFN-γ (10 ng/ml) for 24 h, and subjected to western blotting and RT-qPCR analysis. The band intensities of each protein were quantified using ImageJ software and normalized to GAPDH. Fold changes compared to the control group are expressed as the means ± standard deviations (*n* = 3). Two-way ANOVA with Tukey’s multiple comparison was used for statistical analysis.

A previous study has shown that FGFR activation inhibits IFN-γ signaling, whereas FGFR inhibition enhances the antitumor activity of anti-PD-1 antibodies [26]. However, whether IFN-γ modulates the FGFR signaling pathway remains largely elusive. In RT-112 and RT4 cells, IFN-γ significantly decreased the expression of the FGFR3-TACC3 fusion protein, but not wild-type FGFR3; this effect was partially rescued after 48 h of treatment with FGFR inhibitors (Fig. 5A). Time-course analysis showed that IFN-γ treatment decreased FGFR3-TACC3 expression as early as 12 h after treatment, which correlated with the upregulation of PD-L1 expression in RT-112 cells. Co-treatment with L145 or BGJ398 significantly reversed this phenomenon from 12 to 48 h (Fig. 5B). RT-qPCR analysis further revealed that IFN-γ significantly suppressed the transcription of *FGFR3-TACC3* in RT-112 cells from 24 to 48 h of treatment, which was rescued by L145 or BGJ398 treatment (Fig. 5C). These data suggest that IFN-γ transcriptionally inhibits FGFR3-TACC3 expression in RT-112 cells.

In a time-course analysis, we observed an inverse correlation between PD-L1 production and FGFR3-TACC3 expression (Fig. 5B). PD-L1 reportedly regulates gene expression in the nucleus [39], prompting us to examine the effect of PD-L1 on *FGFR3-TACC3* transcription. Stable PD-L1 overexpression in RT-112 cells resulted in a significant downregulation of both the protein and mRNA levels of *FGFR3-TACC3* without affecting the expression of wild-type FGFR3 (Fig. 5D), which was further confirmed by the transient overexpression of PD-L1 in RT-112 and RT4 cells (Fig. 5E). Stable PD-L1 overexpression had no pronounced effect on the mRNA stability of *FGFR3-TACC3* upon treatment with a transcription inhibitor (Fig. 5F). Furthermore, the siRNA-mediated knockdown of PD-L1 partially rescued the IFN-γ-mediated downregulation of the protein and mRNA levels of *FGFR3-TACC3* in RT-112 cells (Fig. 5G). Our results revealed, for the first time, that IFN-γ-induced PD-L1 transcriptionally suppresses *FGFR3-TACC3* expression in luminal BC cells, which can be reversed by FGFR inhibitors.

### IFN-γ-induced PD-L1 suppresses the promoter activity of FGFR3 in luminal BC cells

**Fig. 6:**
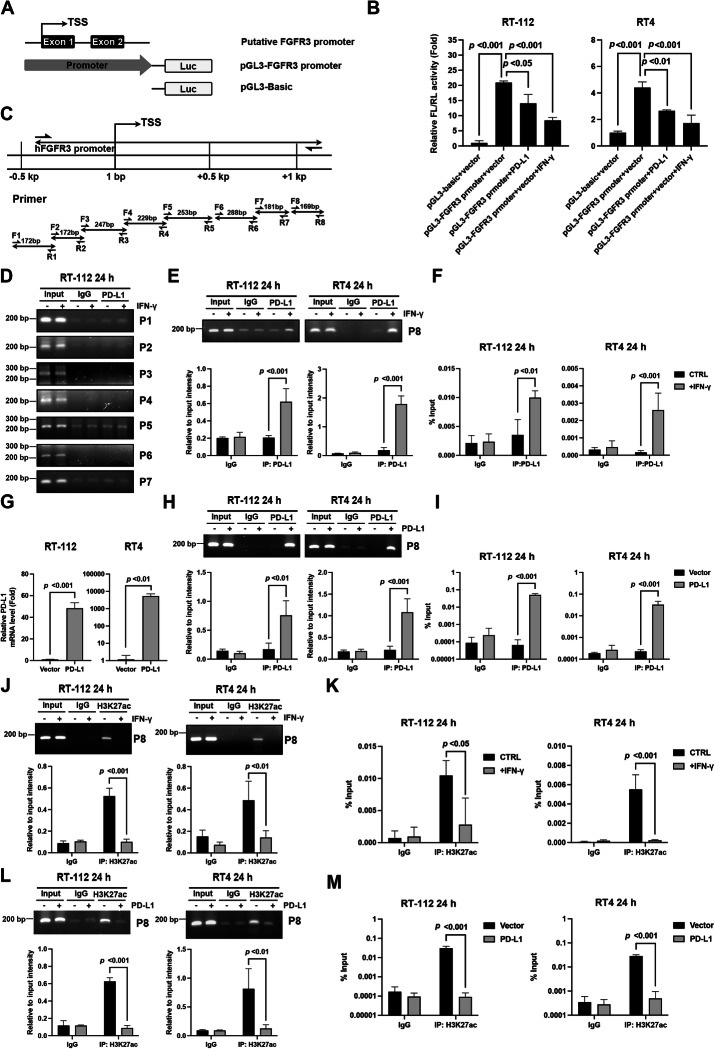
IFN-γ-induced PD-L1 suppresses the promoter activity of FGFR-TACC3 in luminal bladder cancer cells. **A** The putative promoter region of *FGFR3* (chr4: 1,793,293–1,808,867) containing the transcription starting site and exon 1/2 was cloned into the pCL3 plasmid. **B** RT-112 or RT4 cells were co-transfected with pGL3-basic or pGL3-FGFR3 promoter and pRL-SV40P plasmids, control vector, or PD-L1 plasmids, treated with IFN-γ (10 ng/ml) for 24 h, and subjected to a luciferase reporter assay. The ratio of Firefly to Renilla luciferase activity (FL/RL) relative to the activity of the pGL3-basic+vector is expressed as the mean ± standard deviation (n = 3). One-way ANOVA with Tukey’s multiple comparison was used for statistical analysis. **C** Schematic diagram depicting the design of different primer sets for the chromatin immunoprecipitation (ChIP) assay. **D**–**F** RT-112 and RT4 cells were treated with IFN-γ (10 ng/ml) for 24 h, and the protein lysates were subjected to a ChIP assay using normal IgG or anti-PD-L1 antibodies, and the DNA fragments were amplified via PCR (**D**, **E**) or qPCR (**F**). Data are expressed as the mean ± standard deviation (n = 3). **G**, **I** RT-112 and RT4 cells were transfected with control vector or PD-L1 expression plasmids for 24 h. PD-L1 overexpression was confirmed using RT-qPCR (**G**). The protein lysates were subjected to a ChIP assay using normal IgG or anti-PD-L1 antibodies, and DNA fragments were amplified using conventional PCR (**H**) or qPCR (**I**). Data are expressed as the mean ± standard deviation (n = 3). **J**–**M** RT-112 and RT4 cells were treated with IFN-γ (10 ng/ml) (**J**, **K**) or transfected with control vector or PD-L1 plasmids (**L**, **M**) for 24 h, and the protein lysates were subjected to a ChIP assay using normal IgG or anti-H3K27ac antibodies. The DNA fragments were amplified via PCR (**J**, **L**) or qPCR (**K**, **M**). Data are expressed as the mean ± standard deviation (n = 3). Statistical analyses were performed by unpaired two-tailed Student’s *t*-test for data in G; two-way ANOVA with Sidak’s multiple comparison for data in (**E**, **F**, **H**, **M**).

To elucidate the molecular mechanism underlying the PD-L1-mediated transcriptional suppression of *FGFR3-TACC3* in luminal BC cells, we cloned the putative promoter region of *FGFR3* (chr4: 1793293–1808867) containing the transcription starting site and exon 1/2, and examined promoter activity using a dual-luciferase assay (Fig. 6A). The data showed that PD-L1 overexpression or IFN-γ treatment significantly decreased the promoter activity of *FGFR3* in RT-112 and RT4 cells (Fig. 6B). Next, we designed eight primer sets covering the promoter region and performed chromatin immunoprecipitation (ChIP) to confirm whether IFN-γ-induced PD-L1 physically binds to the FGFR3 promoter (Fig. 6C). The data demonstrate that IFN-γ-induced PD-L1 did not interact with promoter regions 1 to 7 (Fig. 6D). Interestingly, IFN-γ-induced PD-L1 significantly bound to promoter region 8 (chr4: 1794235–1794403) of FGFR3 in RT-112 and RT4 cells, as determined via conventional (Fig. 6E) and real-time PCR analysis (Fig. 6F). The enforced expression of PD-L1 (Fig. 6G) further confirmed its binding to the promoter region 8 of FGFR3 (Fig. 6H, I) Additionally, IFN-γ significantly suppressed the distribution of the active histone marker, acetylated H3K27 (H3K27ac), at promoter region 8 in RT-112 and RT4 cells as evidenced via conventional (Fig. 6J) and real-time PCR (Fig. 6K). PD-L1 overexpression recapitulated the decreased distribution of H3K27ac surrounding the promoter region 8 in RT-112 and RT4 cells (Fig. 6L, M). In summary, our findings demonstrate that IFN-γ-induced PD-L1 binds to the FGFR3 promoter and suppresses *FGFR3-TACC3* transcription in luminal-type BC cells.

## Discussion

**Fig. 7:**
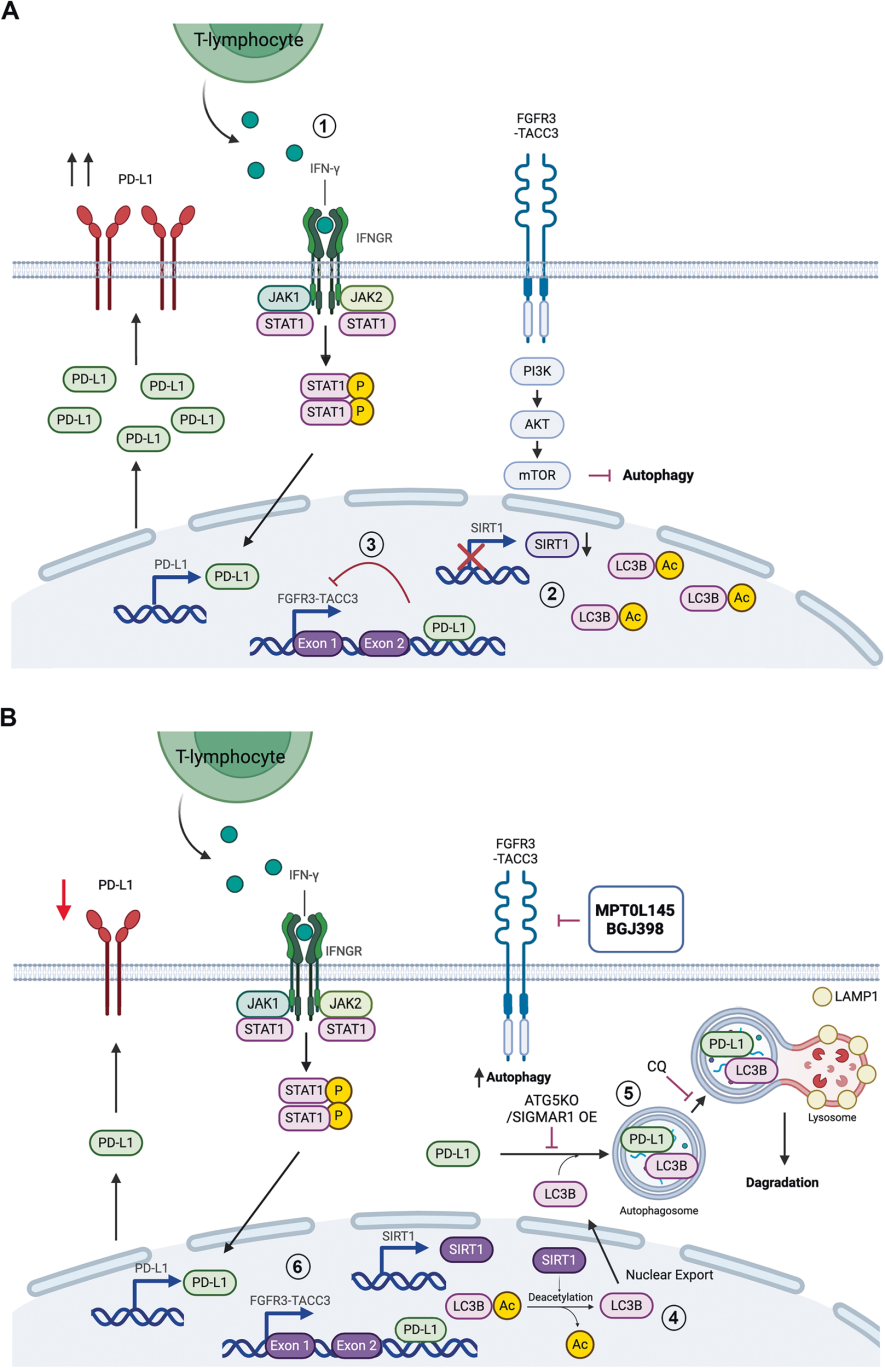
Proposed mechanisms underlying the FGFR inhibitor-mediated suppression of IFN-γ-induced PD-L1 in luminal-type bladder cancer (BC) harboring *FGFR3-TACC3* fusion genes. **A** In this study, we observed that interferon (IFN)-γ promotes the transcription of PD-L1, ameliorates the deacetylation and nuclear export of LC3B, and transcriptionally suppresses *FGFR3-TACC3* expression. (1) In the tumor microenvironment, IFN-γ is primarily secreted by T lymphocytes, which activates the Jak/STAT pathway via the IFN-γ receptor (IFNGR), and increases PD-L1 transcription in luminal BC cells. (2) SIRT1 reportedly deacetylates LC3B, facilitating its nuclear exportation and participation in autophagosome formation in the cytosol. In this study, we observed that IFN-γ reduces *SIRT1* transcription and increases the nuclear accumulation of acetylated LC3B, potentially preventing the autophagic degradation of PD-L1 in the cytosol. (3) For the first time, our data demonstrated that IFN-γ-induced or ectopically expressed PD-L1 binds to the promoter of *FGFR3* (chr4: 1794235–1794403) and inhibits *FGFR3-TACC3* transcription. **B** FGFR inhibitors suppress IFN-γ-induced PD-L1 by promoting autophagy-lysosomal degradation. (4) FGFR inhibitors reversed the transcriptional suppression of *SIRT1* mediated by IFN-γ, increasing the deacetylation of LC3B and its colocalization with PD-L1 in the cytosol. (5) ATG5 knockout and enforced expression of SIGMAR1, a protein known to protect PD-L1 from autophagic degradation, reversed PD-L1 degradation. Chloroquine (CQ), a lysosomal inhibitor, reversed the degradation of PD-L1. Confocal microscopy further showed an increased colocalization of PD-L1 and LAMP1, a lysosomal marker, confirming that FGFR inhibitors can inhibit IFN-γ-induced PD-L1 through autophagy-lysosomal degradation. (6) Finally, our data demonstrated that knocking down PD-L1 or treatment with FGFR inhibitors rescued the transcriptional suppression of FGFR3-TACC3 level mediated by IFN-γ. Created with BioRender (https://www.biorender.com/).

The current study investigated the impact of FGFR inhibitors on IFN-γ-induced PD-L1 expression and their potential effect on antitumor immunity in luminal-type BC cells harboring *FGFR3-TACC3* fusions. The results demonstrate that FGFR inhibitors significantly suppressed IFN-γ-induced PD-L1 by promoting autophagy-lysosomal degradation (Fig. 7). Mechanistically, IFN-γ decreases the nuclear export of LC3B by downregulating SIRT1-mediated deacetylation, preventing the autophagic degradation of PD-L1. FGFR inhibitors, on the other hand, restore SIRT1 expression and promote the deacetylation and nuclear export of LC3B, facilitating the autophagy-lysosomal degradation of PD-L1. For the first time, our data demonstrated that IFN-γ-induced or ectopically expressed PD-L1 binds to the FGFR3 promoter (chr4: 1794235–1794403), and inhibits *FGFR3-TACC3* transcription. These findings reveal the complex interplay between FGFR signaling and IFN-γ-mediated immune regulation, highlighting a new understanding in FGFR3-driven BC.

Prior study reports that FGFR3 destabilizes PD-L1 via NEDD4-mediated proteasomal degradation, and that pharmacological inhibition of FGFR3 upregulates PD-L1 expression, thereby impairing CD8^+^ T cell responses [27]. These findings contradict our observation that FGFR inhibitors suppress IFN-γ-induced PD-L1 via autophagic degradation. The discrepancy may be attributed to the prior study’s focus on basal PD-L1 levels, and the upregulation of PD-L1 correlates with stronger antiproliferative effects of FGFR inhibitors [27]. In our study, FGFR inhibitor concentrations were below the IC_50_ in luminal-type BC cells (Supplementary Fig. S1C). Growing evidence indicates that the PD-L1 protein undergoes degradation via multiple mechanisms, including proteasomal and lysosomal pathways [40]. Consistent with our findings, FGFR signaling has been shown to enhance IFN-γ-induced PD-L1 expression via glycosylation, while FGFR inhibitors suppressed PD-L1 induction in head and neck squamous cell carcinoma [41]. Furthermore, HIP1R reportedly binds PD-L1 and facilitates its autophagy-lysosomal pathway, whereas DHHC3-mediated palmitoylation of PD-L1 prevents its autophagic degradation [42, 43]. Together, these data suggest that post-translational modifications may play a role in modulating IFN-γ-induced PD-L1 expression in FGFR3-TACC3-driven BC.

Previous studies have shown that activation of FGFR suppresses the IFN-γ-stimulated JAK/STAT signaling by upregulating SOCS1, whereas FGFR inhibition restores the expression of IFN-γ target genes and sensitizes antitumor activity of anti-PD-1 antibodies [25, 26]. In contrast, our study uncovers a novel role of IFN-γ-induced PD-L1 in directly binding to the FGFR3 promoter and suppressing *FGFR3-TACC3* transcription, without affecting wild-type FGFR3 (Figs. 5 and 6). These findings uncover a potential reciprocal negative feedback loop between FGFR3-TACC3 and IFN-γ signaling in luminal-type BC cells, wherein constitutive activation of FGFR3 signaling inhibits the IFN-γ pathway [26]; while IFN-γ suppresses *FGFR3-TACC3* transcription through PD-L1 induction. Recent studies have identified a novel regulatory pathway involving nuclear PD-L1 in chromatin remodeling, in which the HDAC2-mediated deacetylation of PD-L1 promotes its nuclear translocation by interacting with key regulatory proteins involved in endocytosis and nuclear translocation [44]. The nuclear transportation of PD-L1 is associated with cancer cell proliferation and the upregulation of immune checkpoint molecules such as PD-L1, PD-L2, VISTA, and B7-H3, leading to acquired resistance to PD-L1/PD-1 blockade [39, 45]. Therefore, an important implication of our study is that the FGFR inhibitor-mediated autophagic degradation of PD-L1 could potentially overcome acquired resistance to ICIs and improve therapeutic outcomes in patients with BC.

Currently, pembrolizumab is approved for the treatment of high-risk patients with NMIBC who are BCG-unresponsive and ineligible for or have declined to undergo cystectomy [8]. However, caution is advised when administering anti-PD-1 therapy to patients with NMIBC harboring FGFR3 activation. Okato et al. developed a murine model that concurrently expresses human equivalents of *Trp53*^*R273H*^ and *FGFR3*^*S249C*^, exhibiting high-grade NMIBC features. Upon anti-PD-1 treatment, tumors demonstrate hyperprogression, which can be attributed to the abundance of Treg cells within the tumor microenvironment; erdafitinib treatment can reverse this by targeting FGFR1 on the Treg cell surface [9]. Recently, the oral or intravesical administration of erdafitinib has shown clinical benefits in high-grade NMIBC patients harboring FGFR alterations who experienced disease recurrence after BCG therapy and refused or were ineligible for radical cystectomy [46, 47]. These observations further support the rationale for combining ICIs and FGFR inhibitors in patients with high-grade NMIBC refractory to BCG treatment.

There are several areas that warrant further investigation to deepen our understanding of the mechanisms described in this study. First, the upstream signals through which IFN-γ suppresses SIRT1 transcription and the functional consequences of SIRT1 inhibition remain unclear. SIRT1, a NAD^+^-dependent deacetylase, is known to suppress inflammatory cytokines by deacetylating the NF-κB P65 subunit [48–50]. Our data show that IFN-γ significantly increases the transcription of IL-6, IL-8, and TNF-α in RT-112 cells, and the co-treatment with FGFR inhibitors further enhances this effect (Supplementary Fig. S3C). However, SIRT1 knockdown did not affect the transcription of these inflammatory cytokines, suggesting that IFN-γ induces their expression through a SIRT1-independent mechanism (Supplementary Fig. S3D). Prior studies indicate that IFN-γ represses SIRT1 via CIITA and HDAC4 recruitment to its promoter [38, 51]. Consistent with this, IFN-γ markedly increases CIITA protein levels in RT-112 cells, which could be reversed by FGFR inhibitors (Supplementary Fig. S3E). These findings suggest that IFN-γ may transcriptionally repress SIRT1 through CIITA-associated chromatin remodeling. In addition, while PD-L1 binds directly to the FGFR3 promoter, the cofactors that contribute to the repression of *FGFR3-TACC3* transcription remain to be elucidated. IFN-γ reportedly represses M2-like genes in human macrophages by downregulating histone H3 lysine 27 acetylation (H3K27ac) and reducing STAT6 recruitment to enhancers [52]. Our data show that IFN-γ suppresses the distribution of H3K27ac at the promoter region of FGFR3 (Fig. 6J, K). Western blotting revealed a global reduction in active histone marks (H3K4ac, H3K27ac), which was reversed by FGFR inhibitors in RT-112 cells (Supplementary Fig. S3F). These findings suggest that IFN-γ-mediated epigenetic modifications may contribute to the chromatin remodeling in luminal BC. Finally, although FGFR inhibitors abrogated PD1/PD-L1-mediated T cell suppression (Supplementary Fig. S3), their effects on primary immune cells or the microenvironment in murine tumor models harboring *FGFR3-TACC3* fusion warrant further investigation.

In conclusion, our findings demonstrate that FGFR inhibitors significantly reduce IFN-γ-induced PD-L1 expression in luminal-type BC cells by promoting autophagy-lysosomal degradation. Additionally, we revealed that IFN-γ-induced PD-L1 suppressed *FGFR3-TACC3* transcription, suggesting a feedback loop between them. These results underscore the potential of combining FGFR inhibitors with ICIs to enhance the antitumor immune responses in FGFR3-TACC3-driven cancers. Further investigation of this regulatory axis may provide new therapeutic avenues for treating luminal-type BC.

## Materials and methods

### Cell culture and reagents

RT-112, RT4, UM-UC-3, and J82 cells were obtained and cultured as previously described [34]. T24, HT-1197, HT-1376, and 5637 cells were purchased from Bioresource Collection and Research Center (Hsinchu, Taiwan). RT-112-*PIK3C3* (VPS34)-KD, RT-112-*ATG5*-KO and RT-112-*FGFR3-TACC3*-KO cells were generated from our previous studies [30, 34]. The cells were cultured in RPMI 1640 (RT-112 and 5637), McCoy’s 5 A (RT4 and T24), and EMEM (UM-UC-3, J82, HT-1197, and HT-1376) culture media at 37 °C with 5% CO_2_, supplemented with 10% FBS and 1× antibiotic-antimycotic (Thermo Fisher Scientific; Waltham, MA, USA). MPT0L145 was synthesized by Dr. Jing-Ping Liou according to a previously described method [30]. BGJ398 (HY-13311), erdafitinib (HY-18708), MG132 (HY-13259), SA4503 (HY-14813), and chloroquine (HY-17589A) were purchased from MedChem Express (Monmouth Junction, NJ, USA). Actinomycin D was purchased from Cayman (Ann Arbor, MI, USA). IFN-γ was purchased from PeproTech (Rocky Hill, NJ, USA). Anti-human CD3 and CD28 antibodies were purchased from BioLegend (San Diego, CA, USA). Other chemicals used in this study were purchased from Sigma-Aldrich (St. Louis, MO, USA).

### Cell viability analysis

The cells were seeded in 96-well plates and treated with MPT0L145, BGJ398 or erdafitinib for 24 or 48 h, and the cell viability was determined by reacting with 3-(4,5-dimethylthiazol-2-yl)-2,5-diphenyltetrazolium bromide for 1 h. The formazan crystals formed were dissolved in DMSO, and the absorbance at 570 nm was measured using a spectrophotometer. Cell viability was expressed as the percentages of absorbance of the treatment group with respect to DMSO-treated control group.

### Western blot analysis

The cells were seeded in a petri dish and cultured overnight. The cells were then treated with the specified concentrations of the drug for specified times. Afterward, the cells were lysed and subjected to western blotting. The same amount of protein was separated according to molecular weight using SDS-PAGE and specific antibodies were used to identify the target proteins. The antibodies used in this study were obtained from the following sources: PD-L1 (CS13684) and ATG5 (CS12994) were obtained from Cell Signaling Technology (Danvers, MA, USA); FGFR3 (ab133644) and acetyl-histone H3 (Lys27) (ab4729) were purchased from Abcam (Cambridge, UK); TACC3 (PA5-36349) was obtained from Thermo Fisher Scientific; VPS34 (GTX129528), P62 (GTX100685), SIGMAR1 (GTX115389) and GAPDH (GTX100118) were obtained from GeneTex (Irvine, CA, USA); LC3B (NB100-2220) was purchased from Novus (Littleton, CO, USA); MCL-1 (sc-819) and CIITA (sc-13556) were obtained from Santa Cruz (Dallas, TX, USA); Flag (F3165) and acetyl-histone H3 (Lys4) (07-539) were purchased from Sigma-Aldrich.

### Reverse transcription quantitative PCR (RT-qPCR)

Total RNA was isolated using TRIzol reagent (Thermo Fisher Scientific), and RNA was reverse-transcribed into complementary DNA using a HiScript I First Strand cDNA Synthesis Kit from Bionovas (Toronto, ON, Canada). Quantitative real-time PCR (qPCR) analysis was performed on a StepOne Real-Time PCR System (Thermo Fisher Scientific) using gene-specific primers (Supplementary Table S1) and the RealQ Plus 2X Master Green (A325402; Ampliqon, Odense, Denmark). The relative expression levels of the targeted genes were normalized to the that of the internal control 18S rRNA gene.

### FACScan flow cytometry analysis

The cells are seeded in a 6-well plate (3 × 10^5^ cells/well) and treated with DMSO or different drugs for a specified time. The cells were detached with trypsin, washed once with PBS, and incubated with mouse anti-PD-L1 (14-5983-82; Thermo Fisher Scientific) antibodies in PBS on ice for 1 h. The cells were washed once with PBS and incubated with a CF594-conjugated goat anti-rabbit IgG (H + L) (20955; Biotium, Fremont, CA, USA) in PBS on ice for 1 h. The cells were washed three times with PBS, and the distribution of fluorescent signals was analyzed using flow cytometry.

### Plasmid/siRNA transfection and lentiviral expression system

To overexpress PD-L1, a cDNA ORF clone of human PD-L1 in pcDNA3.1 was purchased from SinoBiological (HG10084, Wayne, PA, USA). A lentivirus expression plasmid, pLenti4-PD-L1, was constructed by inserting full-length PD-L1 amplified using pairs of primers (5′- AATGAATTCATGAGGATATTTGCTGTCTTTA -3′ and 5′- AATCTCGAGTTACGTCTCCTCCAAATGTG-3′) into the *Eco*RI and *Xho*I sites of the pLenti4 expression vector (Thermo Fisher Scientific). For transient PD-L1 and SIGMAR1 overexpression, the cells were seeded in 6-well plates, and transfected with pLenti4 vector, pLenti-PD-L1 plasmids or Flag-tagged SIGMAR1 (OHU23479D; GenScript, Piscataway, NJ, USA) by using a TurboFect transfection reagent (Thermo Fisher Scientific) following the manufacturer’s instructions. For the stable overexpression of PD-L1, the cells were transduced with lentiviral particles, and selected using 2 μg/ml puromycin (InvivoGen, San Diego, CA, USA). For siRNA-mediated knockdown experiments, non-targeting control siRNA pool or ON-TARGET *plus* siRNA pools against human PD-L1 and SIRT1 (Horizon Discovery; Cambridge, UK) were transfected using Lipofectamine RNAiMAX (Thermo Fisher Scientific).

### Immunofluorescence and confocal microscopy

Briefly, 5×10^4^ cells were plated on coverslips in 6-well plates and cultured for 24 h. Treat with a different drug for 24 h. Cells were treated as indicated and fixed with 10% formalin for 10 min at room temperature. After fixation, the cells were permeabilized with 0.1% Triton X-100 for 15 min at room temperature. Next, the cells were blocked with 2% BSA in PBS for 45 min at room temperature. The cells were then incubated with anti-LC3B (NB100-2220; Novus), anti-PD-L1 (66248-1-1 g; Proteintech, Rosemont, IL, USA), anti-LAMP1 (CS9091; Cell Signaling Technology), anti-SIRT1 (CS8469; Cell Signaling Technology), or anti-acetyl-lysine (sc-32268; Santa Cruz Biotechnology, Dallas, Texas, USA) antibodies at a 1:100 dilution in 0.1% BSA in PBS for 1 h at room temperature. After incubation for 1 h at room temperature with CF488-conjugated goat anti-mouse IgG (H + L) (20956; Biotium) or CF594-conjugated goat anti-rabbit IgG (H + L) (20955; Biotium), the samples were counterstained with mounting buffer containing DAPI (23004; Biotium) for 24 h at room temperature. Samples were observed using a laser scanning confocal Stellaris 8 Confocal Microscope (Leica Microsystems, Inc., Wetzlar, Germany). Fluorescence intensity was determined by using Volocity software (Quorum Technologies, Guelph, ON, Canada). Pearson’s correlation coefficient was used to quantify colocalization.

### Promoter luciferase assay

To examine the functionality of the FGFR3 promoter, we cloned a 1.5-kb region encompassing the putative sequence (chr4: 1,793293–1,808,867) containing the region 446 bp upstream of exon 1, exon 1, intron 1, exon 2 and the first 355 bp of intron 2 into the pGL3-basic vector (Promega; Madison, WI, USA) using the forward primer 5′-TCCTCCACCTGAGGAATTGCCGCTCACAC-3′ and the reverse primer 5′-TCCGTCTGCGCAGAACCCGAATAACAACAG-3′. The accuracy of the constructs was validated via Sanger sequencing. To conduct the luciferase assay, RT-112 cells were seeded in a 24-well plates and co-transfected with pGL3-basic or pGL3-FGFR3 promoter along with pRL-SV40P plasmids (as an internal control) for 24 h. Luciferase activity was measured using the Dual-Luciferase^®^ Reporter Assay System (Promega).

### Chromatin immunoprecipitation (ChIP) assay

Treated cells were cross-linked using 1% formaldehyde for 10 minutes, and the reaction was quenched by adding glycine to a final concentration of 0.125 M for 5 min at room temperature. Subsequently, the cells were harvested via scraping, rinsed twice with PBS, and resuspended in a lysis buffer (1% SDS, 10 mM EDTA, 50 mM Tris-HCl, pH 8), followed by centrifugation at 1000 × *g* for 5 min. The resulting pellet was subjected to probe sonication to shear DNA to an average fragment size of 200 to 700 base pairs, and the chromatin is diluted with a ChIP Dilution Buffer (0.01% SDS, 0.11% Triton X-100, 1.2 mM EDTA, 16.7 mM Tris-HCl, pH 8) to a total volume of 1 ml. with a 100-μl aliquot of the diluted chromatin serving as the 10% input sample. Next, 4 μg of a mouse IgG polyclonal isotype control antibody (GTX35009; GeneTex), mouse anti-PD-L1 (14-5983-82; Thermo Fisher Scientific), or rabbit anti-acetyl-histone H3 (Lys27) (ab4729; Abcam) were added to 1000 μg of diluted chromatin, followed by overnight incubation at 4 °C with rotation. Subsequently, 30 μl of protein A/G magnetic beads (88803; Thermo Fisher Scientific) were added and incubated for 2 h at 4 °C with rotation, and the beads were pelleted using a magnetic separation rack. The beads were then washed three times with 1 ml of a low-salt wash buffer (1% SDS, 1% Triton X-100, 2 mM EDTA, 0.5 mM NaCl, 20 mM Tris-HCl pH 8.0), followed by a single wash with 1 ml of high-salt wash buffer (1% SDS, 1% Triton X-100, 2 mM EDTA, 150 mM NaCl, 20 mM Tris-HCl pH 8.0). After pelleting the magnetic beads, the chromatin was eluted by ChIP elution buffer (1% SDS and 0.1 M NaHCO_3_) under 65 °C for 30 min in a water bath, and the DNA fragments were de-crosslinked and purified using a DNA Purification kit (#14209, Cell Signaling Technology) according to the manufacturer’s instructions. For traditional PCR analysis, the DNA fragments were amplified using a Taq PCR master mix (RT803R; Bioman, New Taipei City, Taiwan) following the manufacturer’s instructions. The PCR products were then analyzed via 1.5% agarose gel electrophoresis, and the band intensities were quantified using ImageJ software (version 1.51, National Institutes of Health; Bethesda, MD, USA).

For qPCR analysis, the DNA fragments were amplified on a StepOne Real-Time PCR System (Thermo Fisher Scientific) using a RealQ Plus 2X Master Green (A325402; Ampliqon). The efficiency of immunoprecipitation was calculated using the percent input method [53] and the equation shown below, in which the signals obtained from each immunoprecipitation are expressed as a percentage of the total input chromatin. The primer pairs used in ChIP assay were shown in Supplementary Table S2.

Percent Input=10% × 2^(C[T] 10 % Input Sample-C[T] IP Sample)^

C[T]=CT=Average threshold cycle of PCR reaction

### T-cell activation assay

Jurkat-Lucia™ TCR-hPD-1 cells (Invivogen) stably expressing TCR, hCD28, hPD-1, and NFAT-inducible Lucia luciferase reporter gene were used in this assay. RT-112 cells were treated with IFN-γ (10 ng/ml) alone or in combination with MPT0L145 (L145) or BGJ398 for 48 h, and then trypsinized and co-cultured with Jurkat-Lucia™ TCR-hPD-1 cells at a ratio of 1:5 (RT-112: Jurkat) in the presence of anti-CD3 (2 μg/ml) and anti-CD28 (4 μg/ml) antibodies for another 24 h. The supernatants were then collected, and T cell activation was measured using a QUANTI-Luc™ luciferase detection reagent (Invivogen) according to the manufacturer’s instructions.

### Statistical analysis

Data analysis was performed using GraphPad Prism software (10.4.1). Results are expressed as means ± standard deviation (SD) based on at least three independent experiments. Statistical comparisons between two groups were conducted using an unpaired two-tailed Student’s *t*-test. One-way or two-way analysis of variance (ANOVA) with post-hoc analysis was used for comparisons among multiple groups. Details on group sizes and statistical methods are provided in the figure legends. A *p*-value of <0.05 was considered statistically significant.

## Supplementary information

Supplementary materials

Original WB data

## Data Availability

All data generated or analyzed during this study are available from the corresponding author upon reasonable request.
