# Supplementary material for: FGFR inhibitors promote the autophagic degradation of IFN-γ-induced PD-L1 and alleviate the PD-L1-mediated transcriptional suppression of FGFR3-TACC3 in non-muscle-invasive bladder cancer
Source: Cell Death Dis. 2025 Jul 2;16(1):485. doi: 10.1038/s41419-025-07821-8 (PMC12222871; doi:10.1038/s41419-025-07821-8)
Supplement: Supplementary file 1 — Supplementary materials [file 41419_2025_7821_MOESM1_ESM.docx]

**FGFR Inhibitors Promote the Autophagic Degradation of IFN-γ-induced PD-L1 and Alleviate the PD-L1-mediated Transcriptional Suppression of FGFR3-TACC3 in Non-muscle-invasive Bladder Cancer**

**Yu-Chen Lin^1,2^, Cheng-Ying Chu^3,4^, Tsung-Han Hsieh^5^, Bo-Jyun Lin^1,2^, Jing-Ping Liou^6^, Yun Yen^7,8^, Chun-Han Chen^1,2,9*^**

^1^ Graduate Institute of Medical Sciences, College of Medicine, Taipei Medical University, Taipei 110301, Taiwan

^2^ Department of Pharmacology, School of Medicine, College of Medicine, Taipei Medical University, Taipei 110301, Taiwan

^3^ CRISPR Gene Targeting Core, Taipei Medical University, Taipei 110301, Taiwan

^4^ TMU Research Center of Cancer Translational Medicine, Taipei Medical University, Taipei 110301, Taiwan

^5^ Precision Health Center, Taipei Medical University, Taipei 110301, Taiwan

^6^ School of Pharmacy, College of Pharmacy, Taipei Medical University, Taipei 110301, Taiwan

^7^ Ph.D. Program for Cancer Biology and Drug Discovery, College of Medical Science and Technology, Taipei Medical University, Taipei 110301, Taiwan

^8^ Center for Cancer Translational Research, Tzu Chi University, Hualien 970374, Taiwan

^9^ Cell Physiology and Molecular Image Research Center, Wan Fang Hospital, Taipei Medical University, Taipei 116079, Taiwan

**^*^Corresponding author:**

Chun-Han Chen, Ph.D.

Associate Professor, Department of Pharmacology, School of Medicine, College of Medicine, Taipei Medical University, Taipei 110, Taiwan

E-mail address: [brianchc@tmu.edu.tw](mailto:brianchc@tmu.edu.tw)

**Supplementary Figures**

**
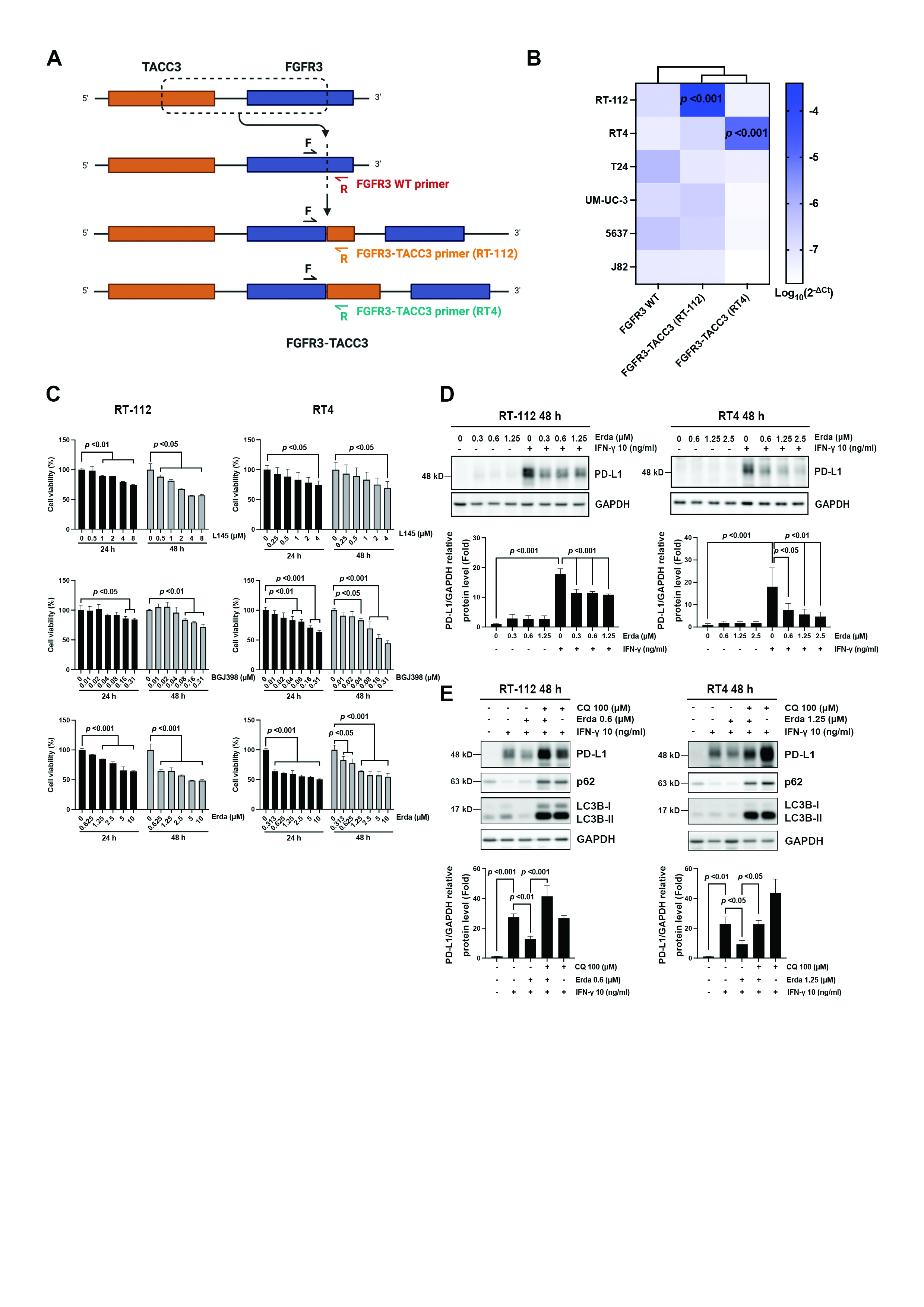
**

**Supplementary Figure S1. Erdafitinib suppresses IFN-γ-induced PD-L1 in luminal bladder cancer cells.**

(A) Schematic diagram depicting the design of specific primer sets for wild-type (WT) *FGFR3*, and the *FGFR3-TACC3* fusions in RT-112 and RT4 cells. (B) Total RNA from different bladder cancer cell lines were collected using TRIzol reagent and subjected to RT-qPCR analysis with the indicated primers. Relative gene expression was calculated using the Log_10_ 2^-ΔCt^, and the data are expressed as the mean ± standard deviation (n=3). Two-way ANOVA with Dunnett’s multiple comparison was used for statistical analysis. (C) RT-112 and RT4 cells were treated with MPT0L145 (L145), BGJ398, or erdafitinib for 24 or 48 h, and the cell viability was measured using the MTT assay. Data are expressed as the mean ± standard deviation (n=3). One-way ANOVA with Dunnett’s multiple comparison was used for statistical analysis. (D) RT-112 or RT4 cells were exposed to indicated concentrations of Erdafitinib (Erda) in the presence or absence of IFN-γ (10 ng/mL) for 48 h and subjected to western blot analysis. The band intensities of each protein were quantified using ImageJ software and normalized to that of GAPDH. Fold changes compared to the control group are expressed as the mean ± standard deviation (n=3). (E) RT-112 and RT4 cells were treated with IFN-γ (10 ng/ml) alone or in combination with the indicated concentrations of erdafitinib (Erda) in the presence or absence of chloroquine (CQ, 100 μM) for 48 h and subjected to western blotting. Data are expressed as the mean ± standard deviation (n=3). One-way ANOVA with Tukey’s multiple comparison was used for analyze data in D–E.


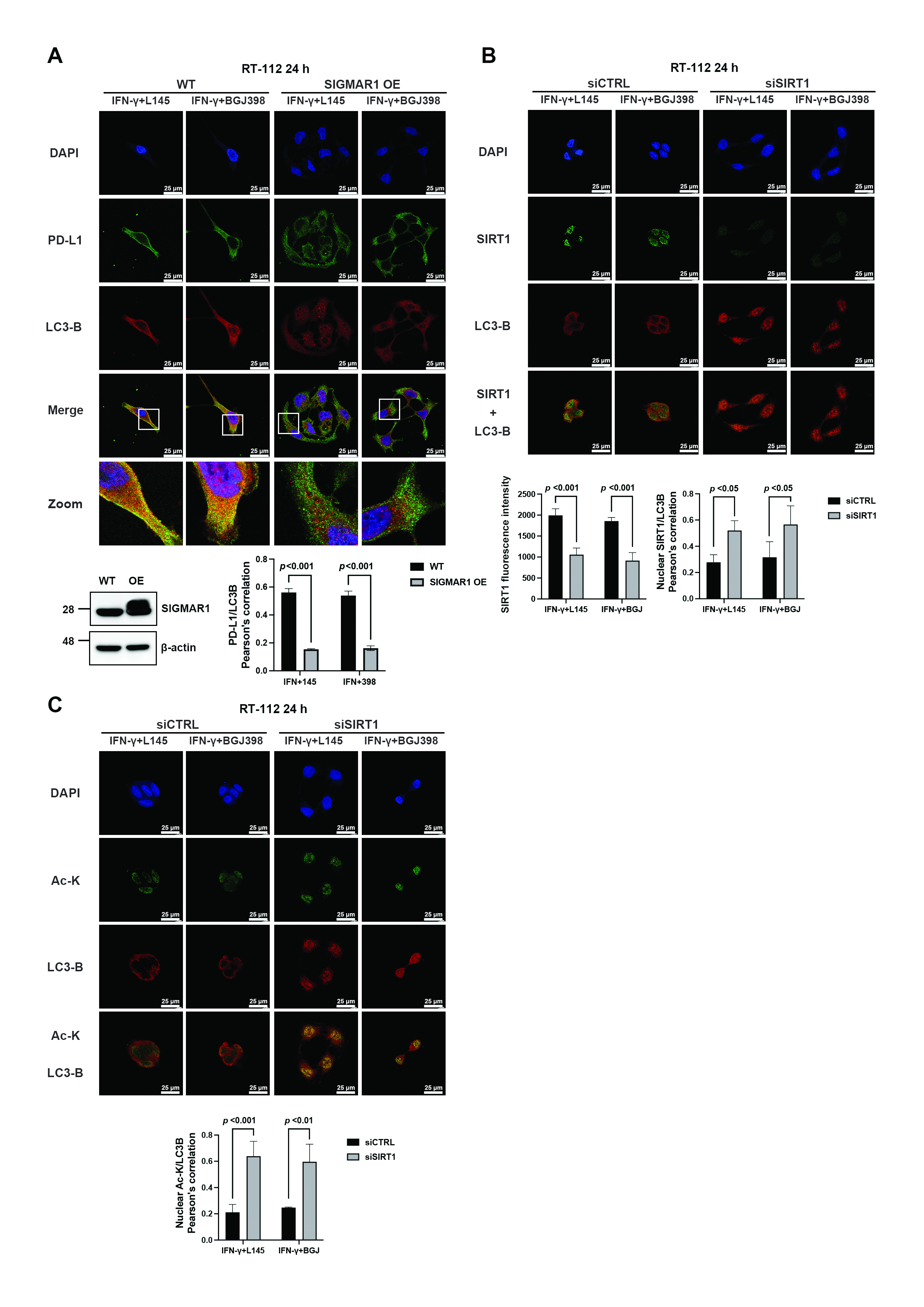


**Supplementary Figure S2. The FGFR inhibitor-mediated nuclear export and deacetylation of LC3B in RT-112 cells depends on SIRT1.**

(A) RT-112 cells were transfected with SIGMAR1 plasmid (SIGMAR1 OE), treated with IFN-γ (10 ng/mL) in the L145 (1 μM) or BGJ398 (0.08 μM) for 24 hours, and subjected to immunofluorescence staining with antibodies against PD-L1 (green) and LC3B (red) and compared with WT group. Following SIGMAR1 OE in cells, WB was performed to confirm the upregulation of SIGMAR1 protein expression. Fluorescence intensity and Pearson’s correlation values are expressed as means ± standard deviations (n=3). FGFR inhibitors mediated nuclear export and deacetylation of LC3B is dependent on SIRT1 in RT-112 cells. RT-112 cells were transfected with control siRNA (siCTRL) or siRNA against SIRT1 (siSIRT1), and then exposed to IFN-γ (10 ng/mL) with L145 (1 μM) or BGJ398 (0.08 μM) for 24h, followed by immunofluorescence staining with andibodies against (B) SIRT1 (green) and LC3B (red) or (C) acetyl-lysine (Ac-K, green) and LC3B (red). Fluorescence intensity or Pearson’s correlation is presented by mean ± standard deviations (n=3). Statistical analyses were performed using two-way ANOVA with Sidak’s multiple comparisons.


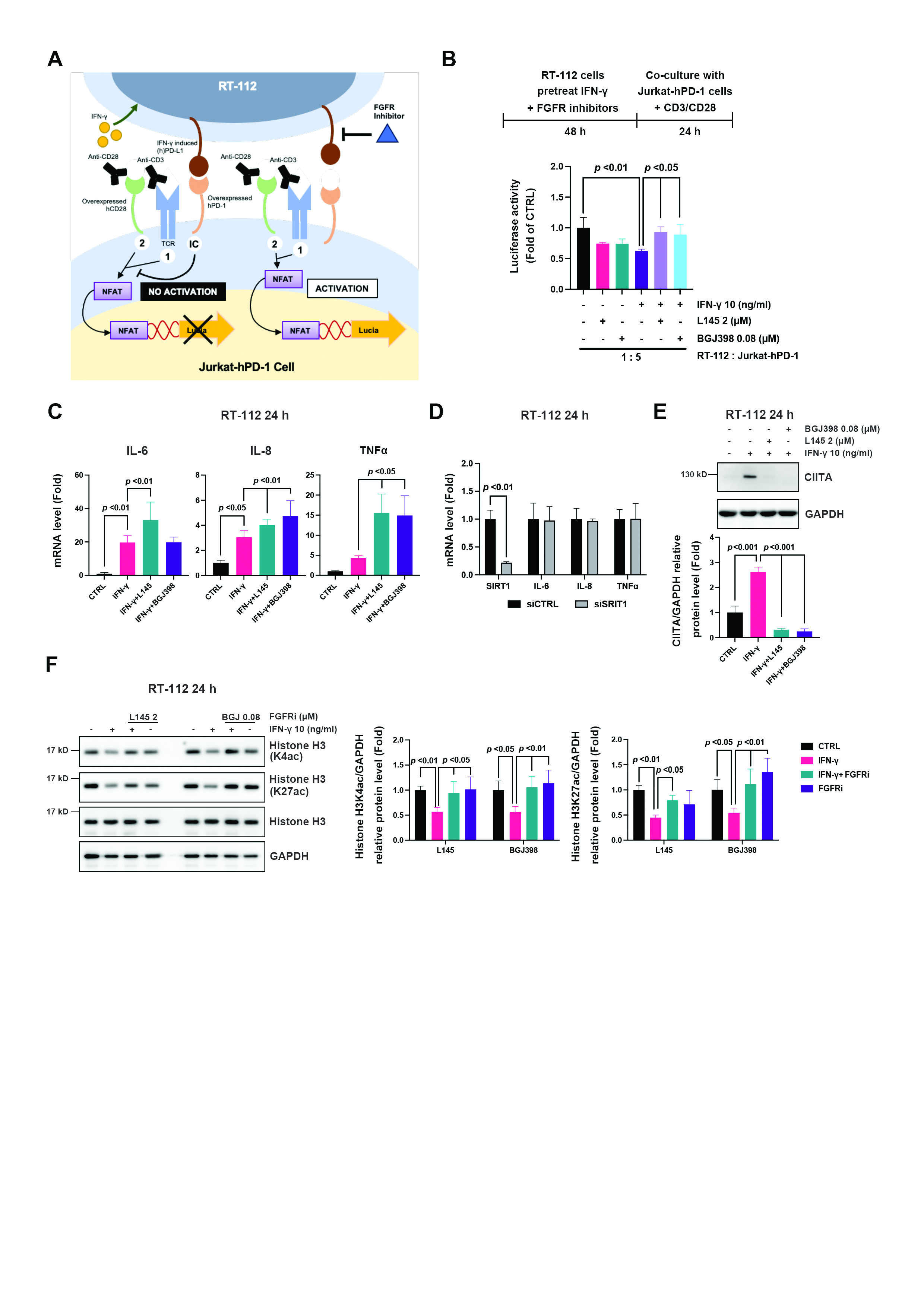


**Supplementary Figure S3. FGFR inhibitors abrogate PD1/PD-L1-mediated T cell suppression.**

(A) Schematic diagram depicts the study design the co-culture assay. Jurkat-hPD-1 cells stably express TCR, CD28 stimulatory receptor, human-PD-L1 and an NFAT-inducible Lucia luciferase reporter gene were co-culture with RT-112 to examine the T-cell activity. (B) RT-112 cells were pre-treated with IFN-γ alone or in combination with MPT0L145 (L145) or BGJ398 for 48 h, and then trypsinezed and co-cultured with Jurkat-hPD-1 cells in the presence of CD3 (2 μg/mL) and CD28 (4 μg/mL) for another 24 h. The supernatants were collected and analyzed by measuring the Lucia activity. Data are expressed as the mean ± standard deviations (n=3). (C) RT-112 cells were treated with control or IFN-γ (10 ng/ml) with or without L145 (2 μM) or BGJ398 (0.08 μM) for 24 h, and subjected to RT-qPCR analyses. Fold changes compared to the control group are expressed as the mean ± S.D. (n=3). (D) RT-112 cells were transiently transfected with control siRNA (siCTRL) or siSIRT1, and subjected to western blotting. The band intensities of each protein were quantified using ImageJ software and normalized to GAPDH. Fold changes compared to the control group are expressed as the means ± standard deviations (n=3). (E) RT-112 cells were treated with interferon IFN-γ (10 ng/ml) alone or in combination with MPT0L145 (L145 2 μM, left) or BGJ398 (0.08 μM, right) 24 h and subjected to western blot analysis. The band intensities of each protein were quantified using ImageJ software and normalized to that of GAPDH. Fold changes compared to the control group are expressed as the mean ± standard deviations (n=3). (F) RT-112 cells were treated with interferon IFN-γ (10 ng/ml) alone or in combination with MPT0L145 (L145 2 μM, left) or BGJ398 (0.08 μM, right) 24 h and subjected to western blot analysis. The band intensities of each protein were quantified using ImageJ software and normalized to that of GAPDH. Fold changes compared to the control group are expressed as the mean ± standard deviations (n=3-5). Statistical analyses were performed using one-way ANOVA with Tukey’s multiple comparisons for data in B–C and E–F; unpaired two-tailed Student’s *t*-test for data in D.

**Supplementary Tables**

**Supplementary Table S1. The primer sequences for real-time PCR**

| **Gene** | **Forward Primer (5′ to 3′)** | **Reverse Primer (5′ to 3′)** |
| --- | --- | --- |
| CD274 (PD-L1) | AATGCTGCACTTCAGATCACA | TGACTGGATCCACAACCAAA |
| FGFR-WT | AGAGGCCCACCTTCAAGC | CAATGTGAGGGGTCCCTAGC |
| FGFR3-TACC3 (RT-112) | AGAGGCCCACCTTCAAGC | CTCACACCTGCTCCTCAGC |
| FGFR3-TACC3 (RT4) | AGAGGCCCACCTTCAAGC | GCCAGACACTTTTCCTGGAG |
| SIRT1 | TAGACACGCTGGAACAGGTTGC | CTCCTCGTACAGCTTCACAGTC |
| IL-6 | AGACAGCCACTCACCTCTTCAG | TTCTGCCAGTGCCTCTTTGCTG |
| IL-8 | GAGAGTGATTGAGAGTGGACCAC | CACAACCCTCTGCACCCAGTTT |
| TNFα | CTCTTCTGCCTGCTGCACTTTG | ATGGGCTACAGGCTTGTCACTC |
| 18sRNA | CGGCGACGACCCATTCGAAC | GAATCGAACCCTGATTCCCCGTC |

**Supplementary Table S2. The primer sequences for chromatin immunoprecipitation**

| **Gene** | **Forward Primer (5′ to 3′)** | **Reverse Primer (5′ to 3′)** |
| --- | --- | --- |
| FGFR3-P1 | GGCCCCAGGTCAGTCAAC | AGCTCAGAGGGGAGGTCTC |
| FGFR3-P2 | GAGACCTCCCCTCTGAGCTC | GTCTCGAGGGCGTGGGAG |
| FGFR3-P3 | CTCCCACGCCCTCGAGAC | GACCGGCAGAGCAGGACC |
| FGFR3-P4 | GGTCCTGCTCTGCCGGTC | GCTGCAGGGACCTCTGTG |
| FGFR3-P5 | CACAGAGGTCCCTGCAGC | GGACAGGGACGAAAGCGG |
| FGFR3-P6 | CCTCCTGTAGTCTCCCGAGC | TTCTTACCTGCCGCTCGCC |
| FGFR3-P7 | CGGCAGGTAAGAAGGGACC | AAGTCGCCGCGCTAGCTC |
| FGFR3-P8 | CGCAGCTACCCTCCAAGTG | AACTTTCCGTCTGCGCAGAA |
