# Supplementary material for: FGFR inhibitors promote the autophagic degradation of IFN-γ-induced PD-L1 and alleviate the PD-L1-mediated transcriptional suppression of FGFR3-TACC3 in non-muscle-invasive bladder cancer
Source: Cell Death Dis. 2025 Jul 2;16(1):485. doi: 10.1038/s41419-025-07821-8 (PMC12222871; doi:10.1038/s41419-025-07821-8)

F1A

Cell line

FGFR3, TACC3, PD-L1, GAPDH

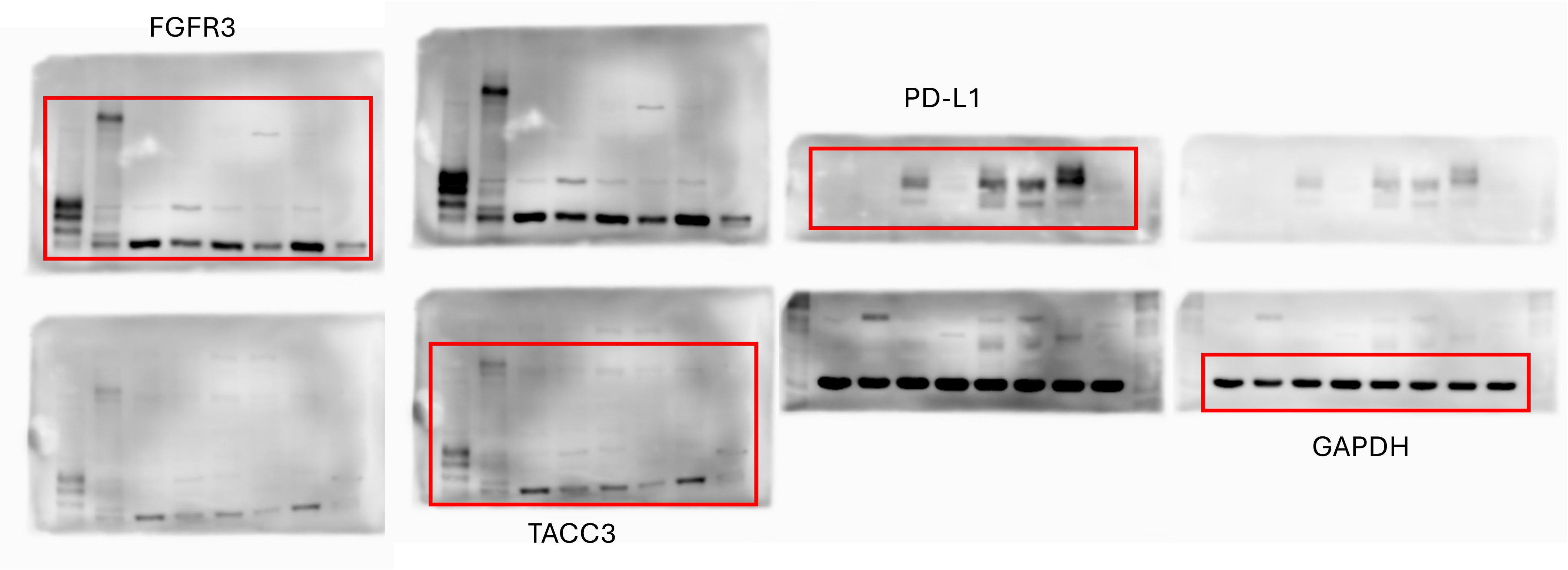

F1B RT-112

2022.01.07 RT-112 **24h n =1**

PD-L1, GAPDH

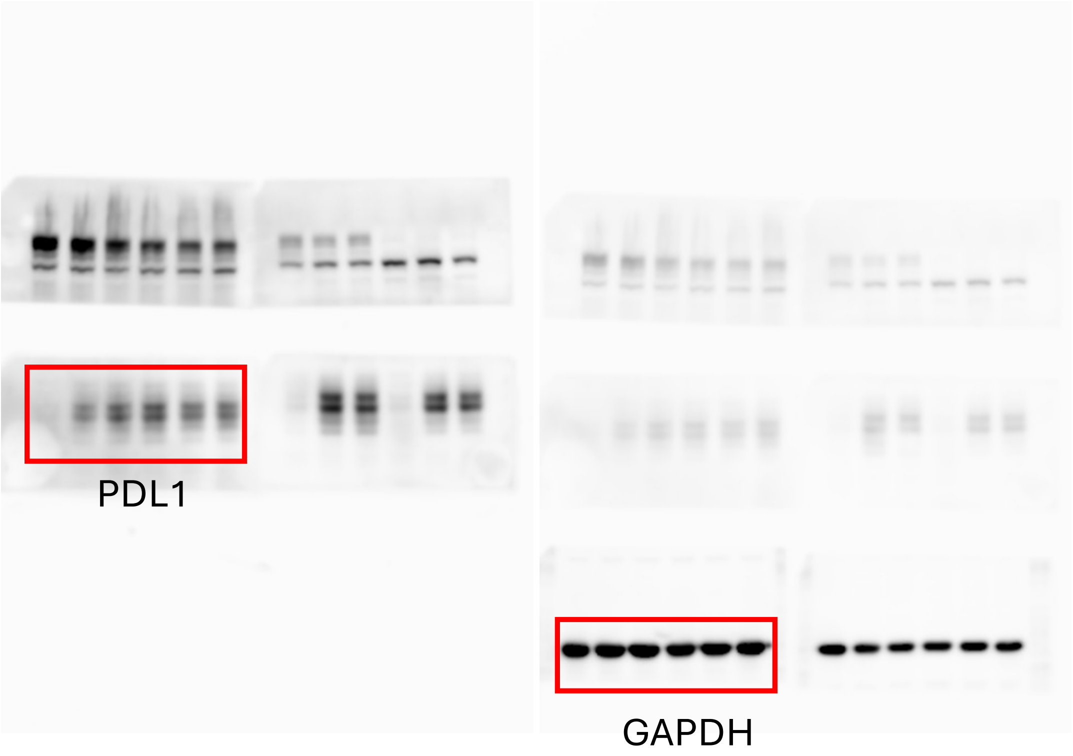

2022.01.05 RT-112 **48h n=1**

PD-L1, GAPDH

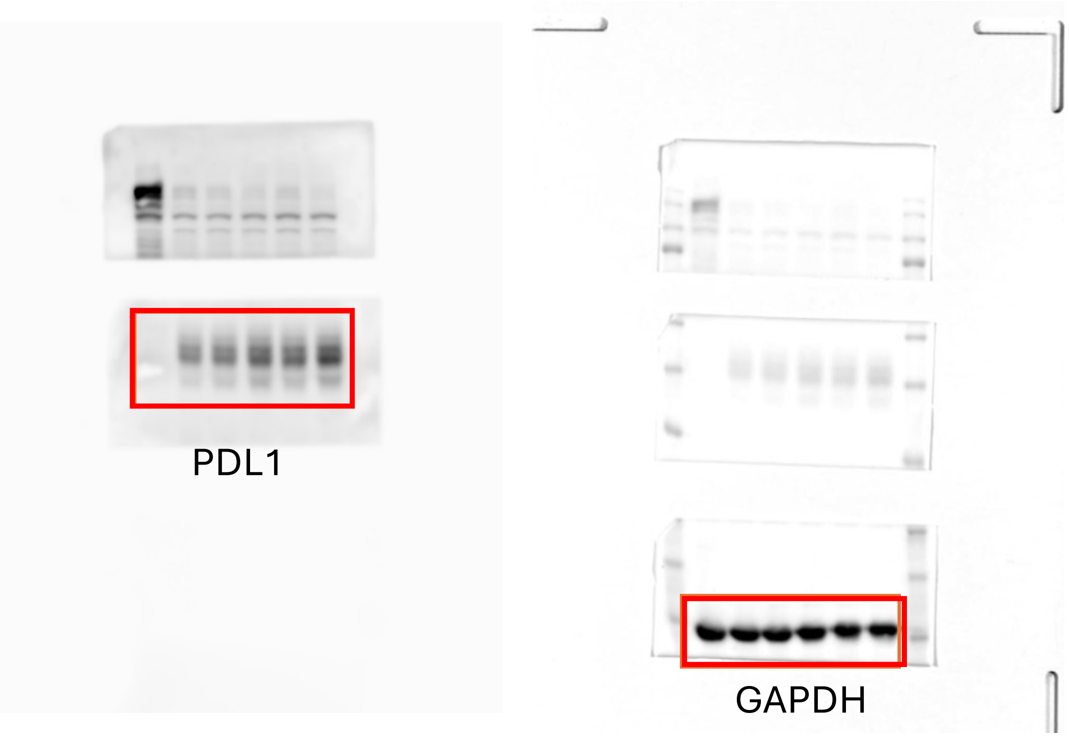

F1B RT4

2022.06.17 RT4 24,48h n=1

PD-L1, GAPDH

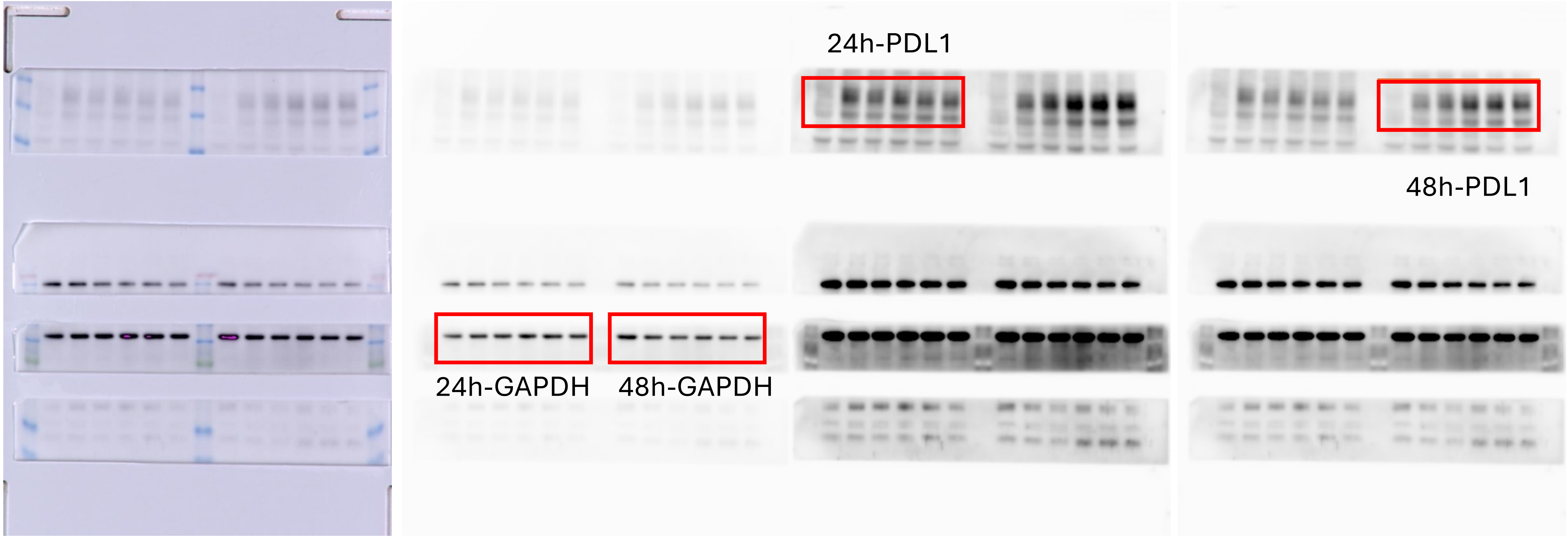

2024.10.19 RT-112, RT4 24,48h n=2
PD-L1, GAPDH

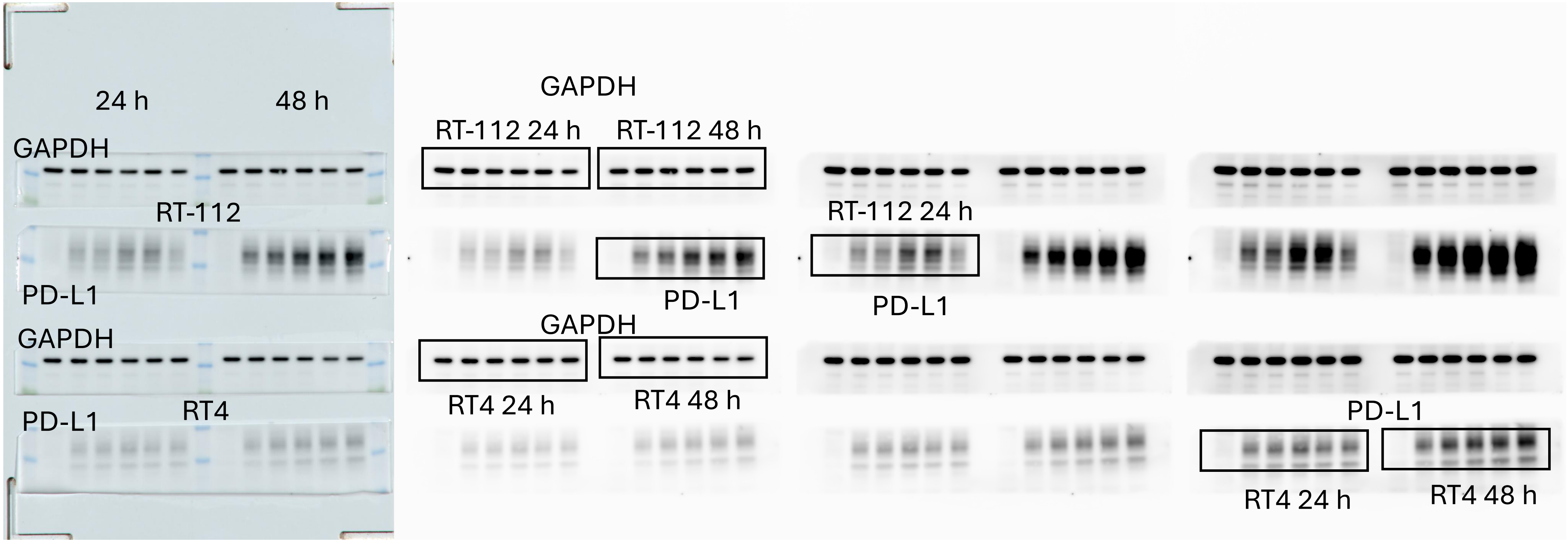

2024.10.27 RT-112, RT4 24, 48h n=3
PD-L1, HSP90

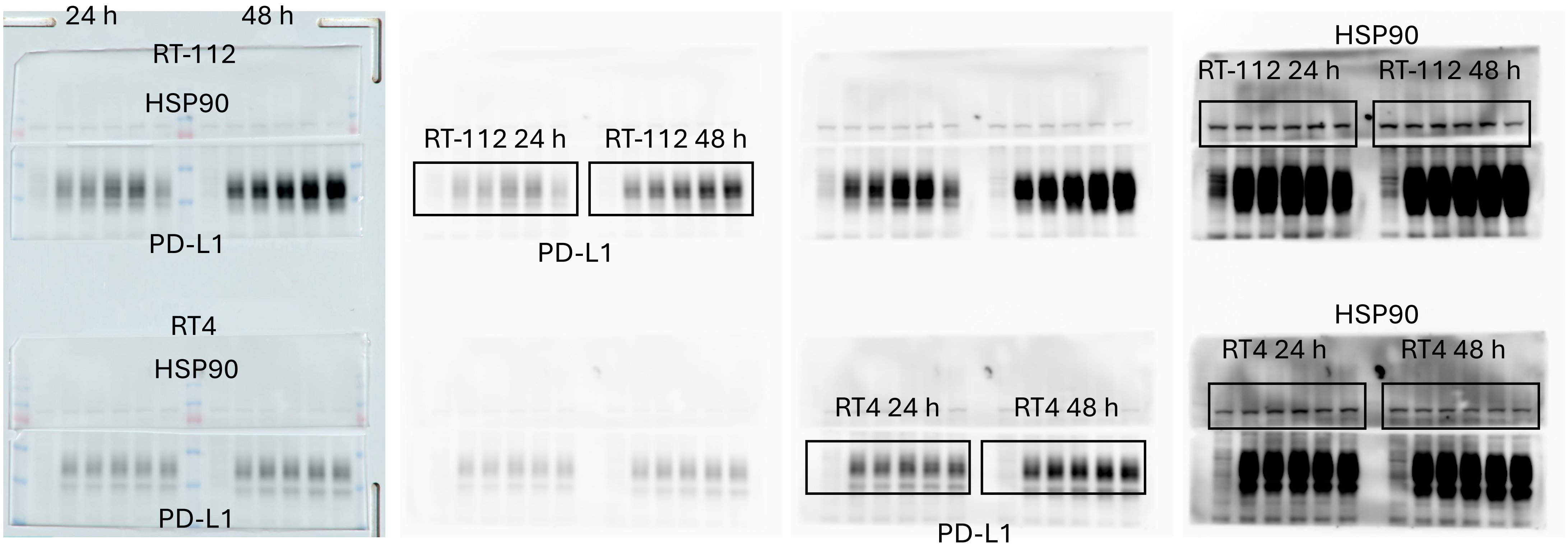

2021.1.05 RT112 n=1
L145-PD-L1, GAPHD
BGJ398-PD-L1, GAPHD

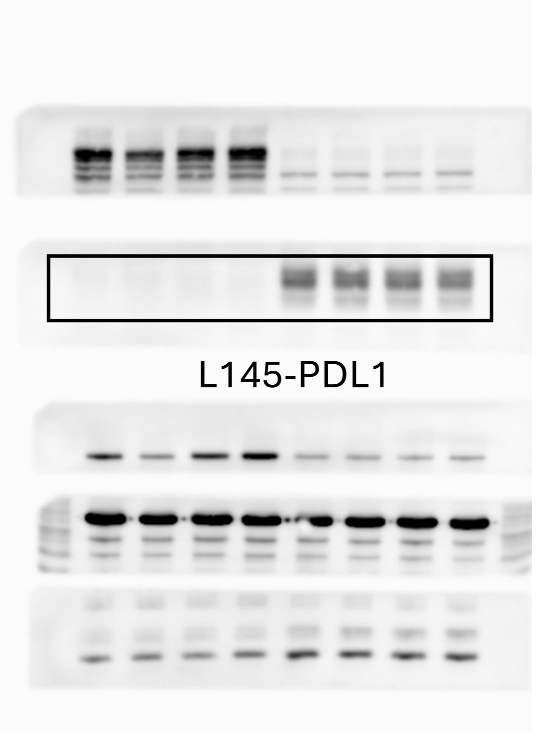

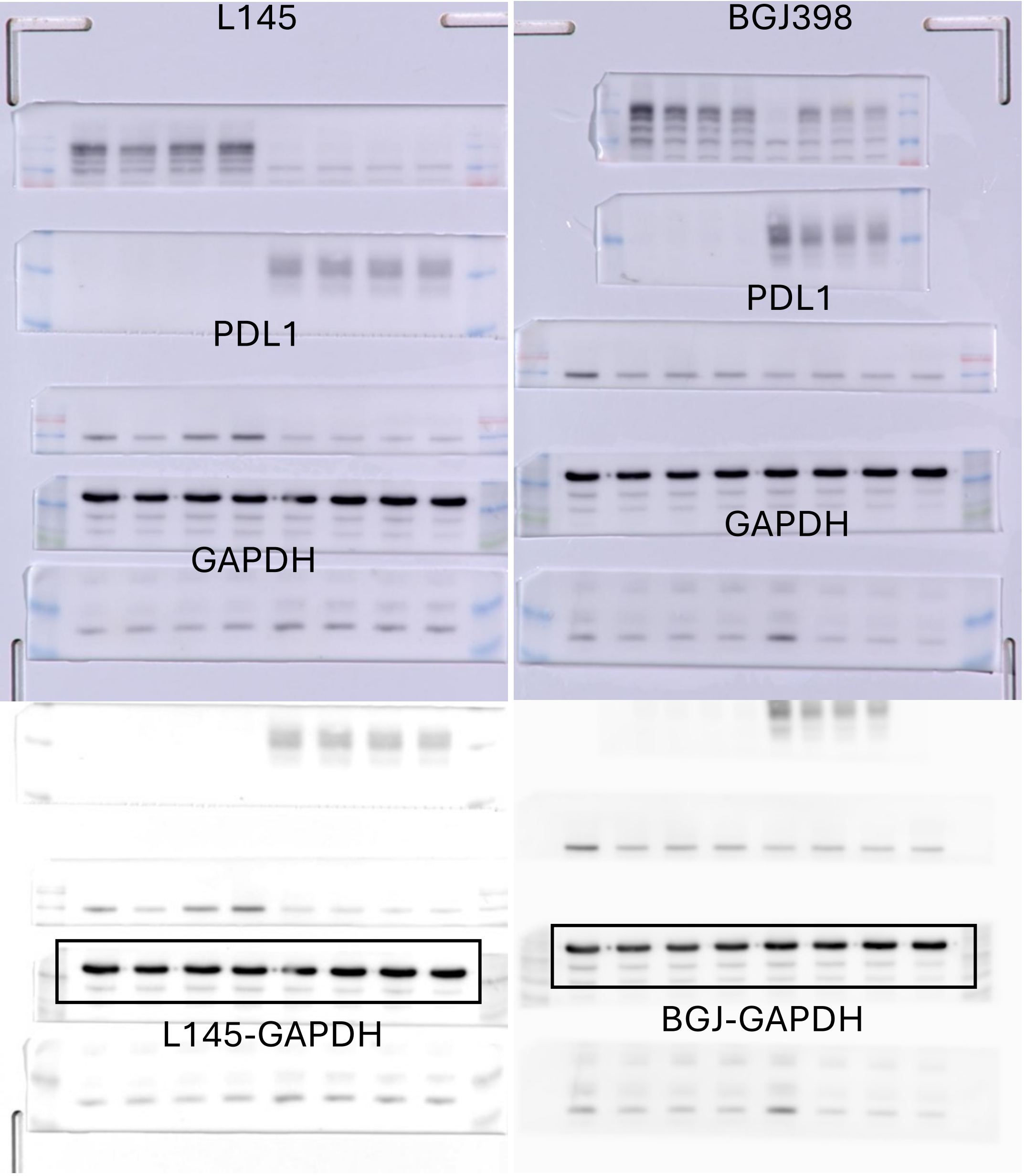

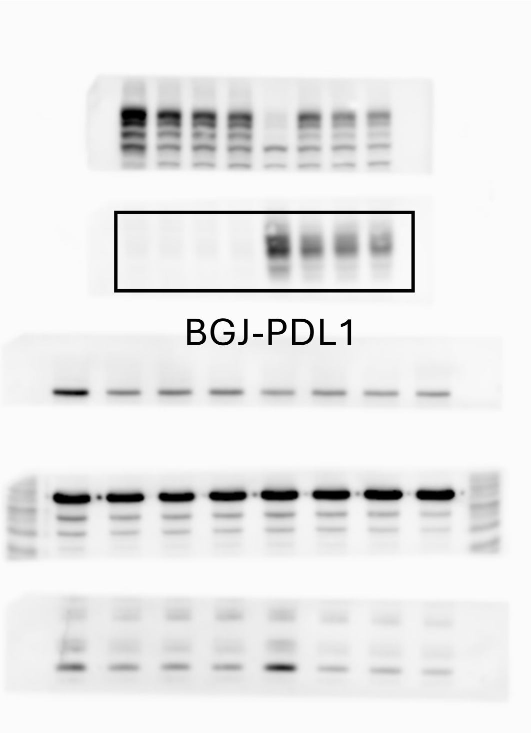

F2 A RT-112 (BGJ398)

2021.1.26 RT112 n=2
L145-FGFR3, PD-L1, GAPHD
BGJ398-FGFR3, PD-L1, GAPHD

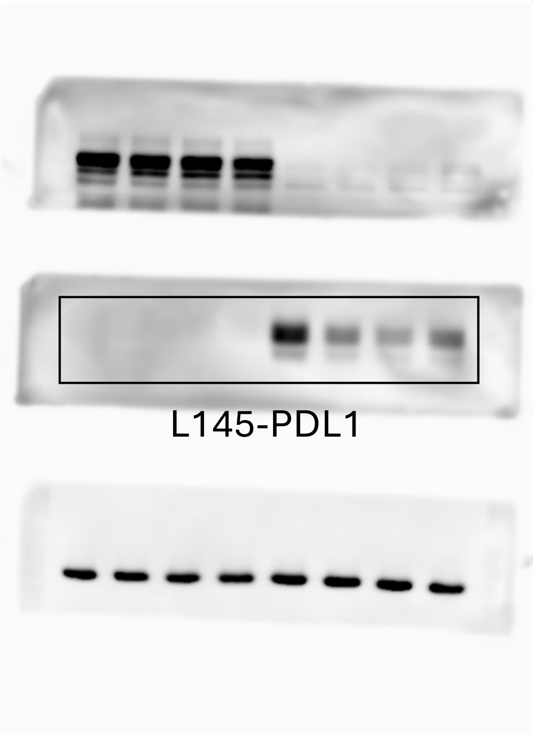

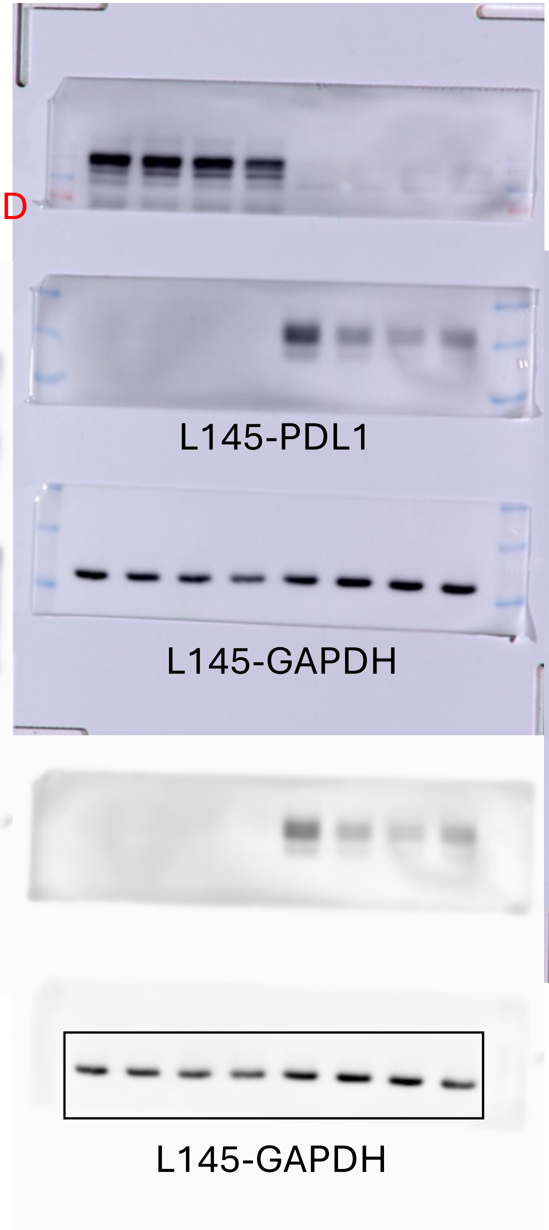

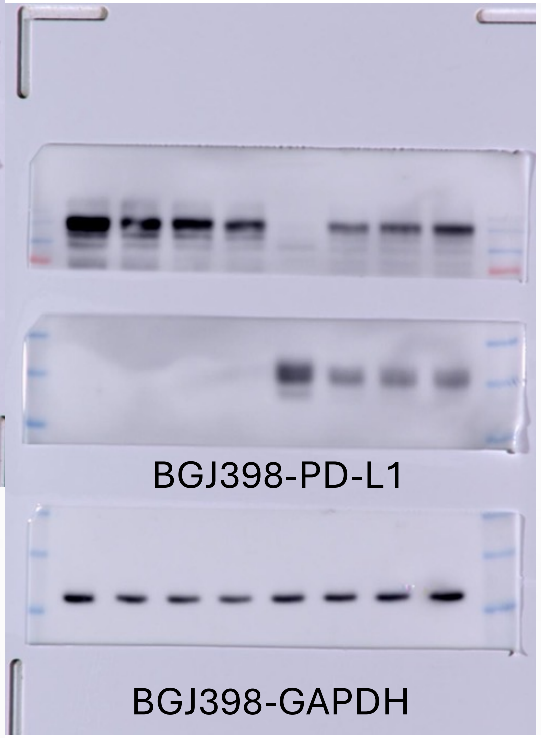

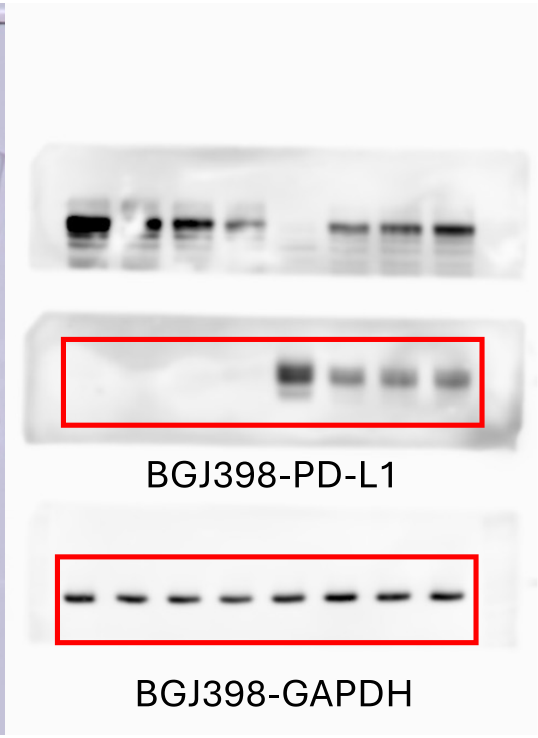

F2 A RT-112 (L145)

2022.4.21 RT112 n=3

L145-PD-L1, GAPHD

BGJ398-PD-L1, GAPHD

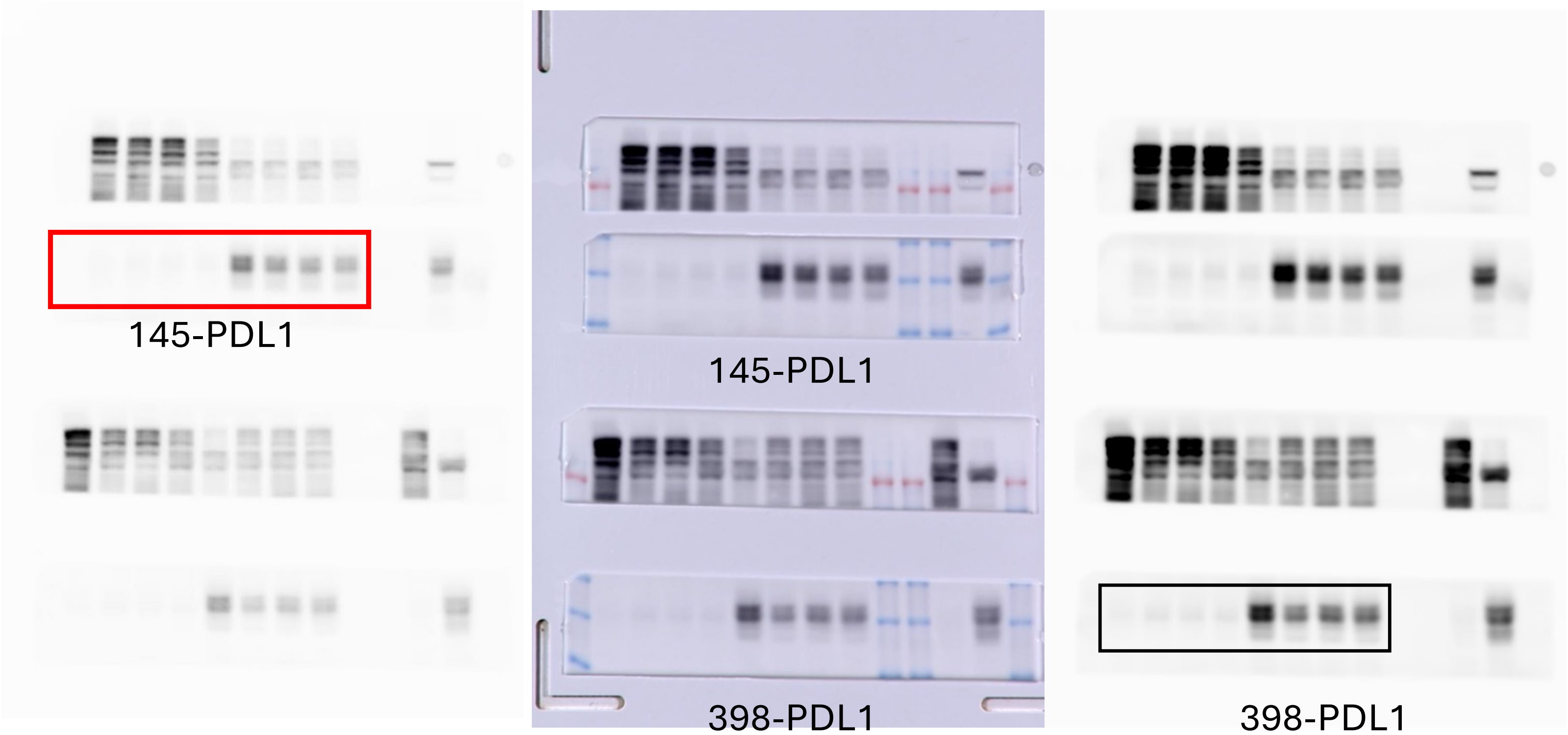

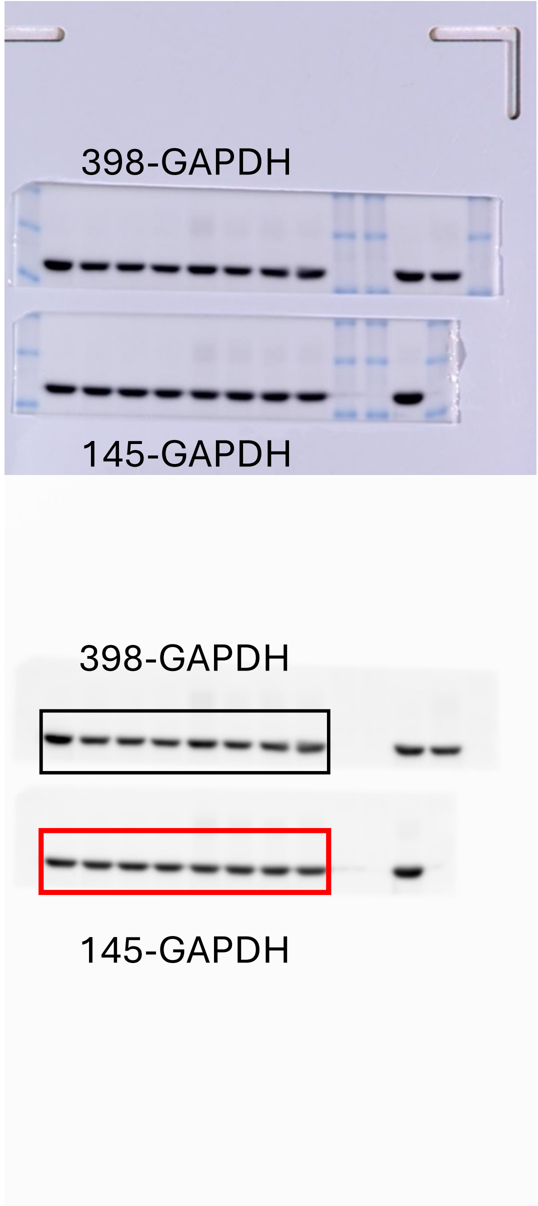

2023.3.3 RT4 n=1

L145-PD-L1,GAPDH

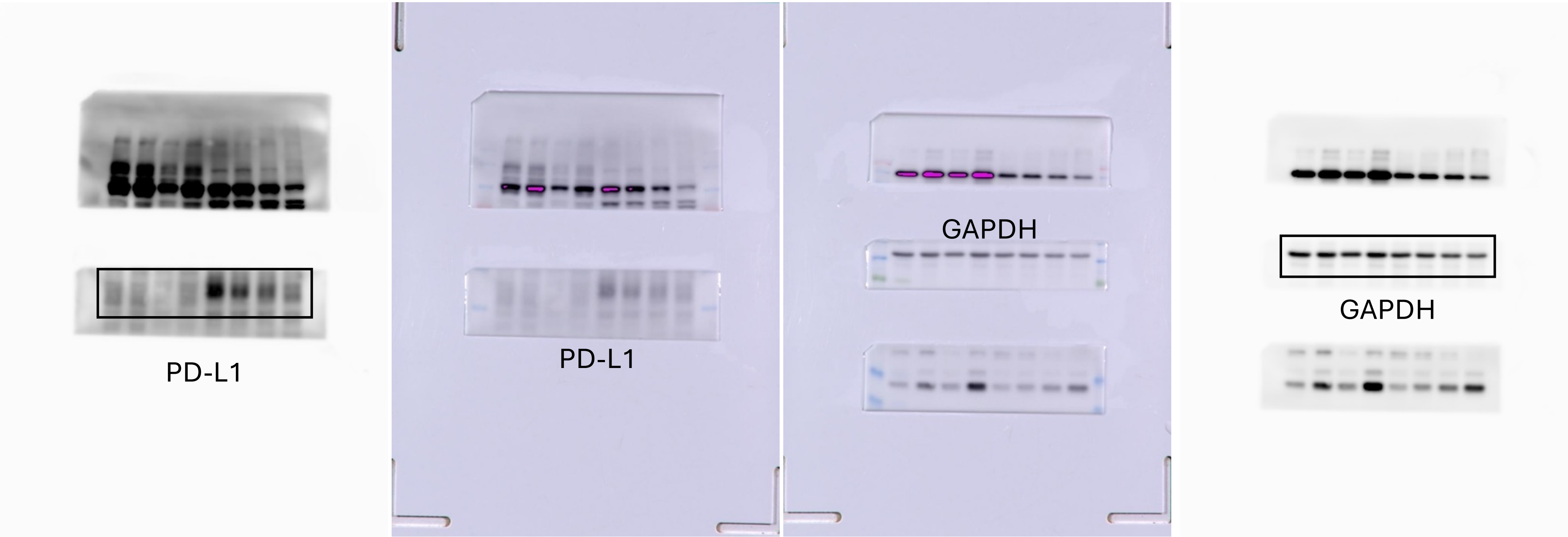

2023.3.9 RT4 n=2
L145-PD-L1,GAPDH

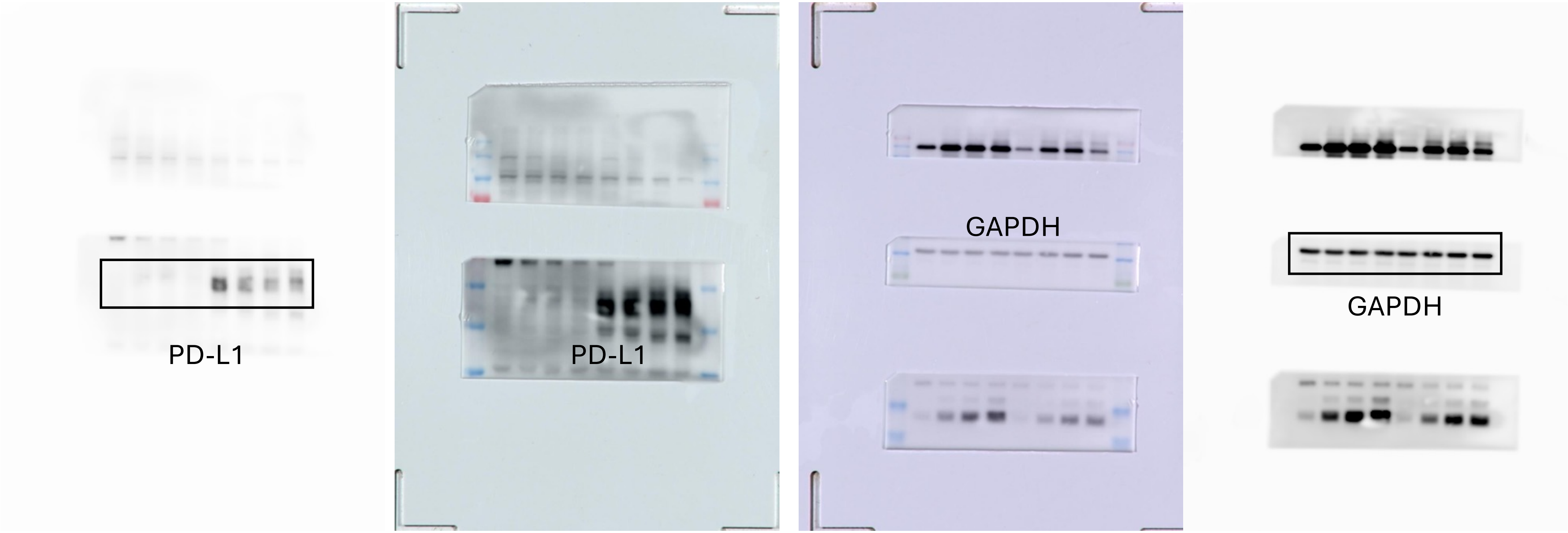

F2A RT4 (L145)

2023.3.17 RT4 n=3

L145-PD-L1,GAPDH

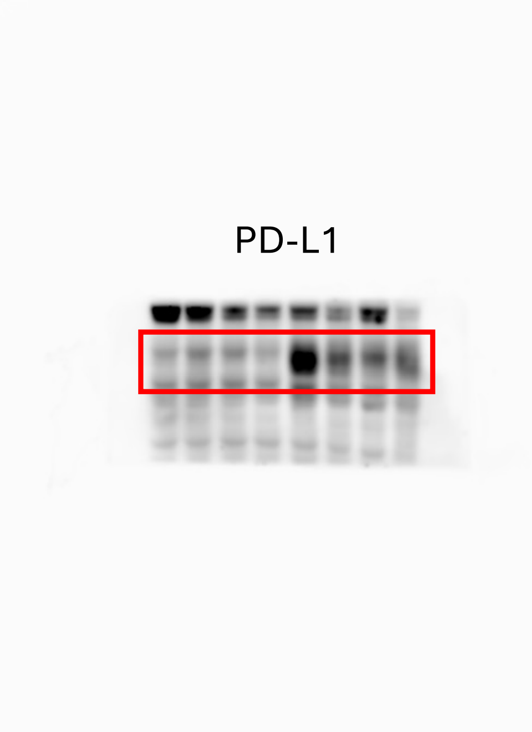

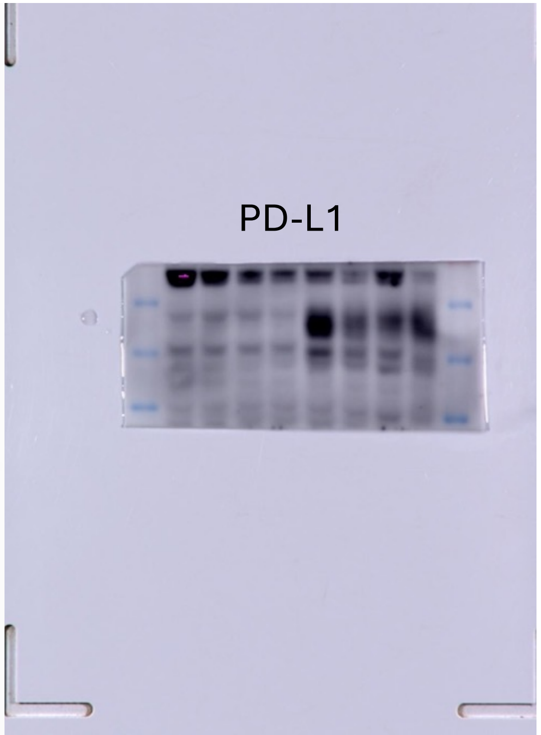

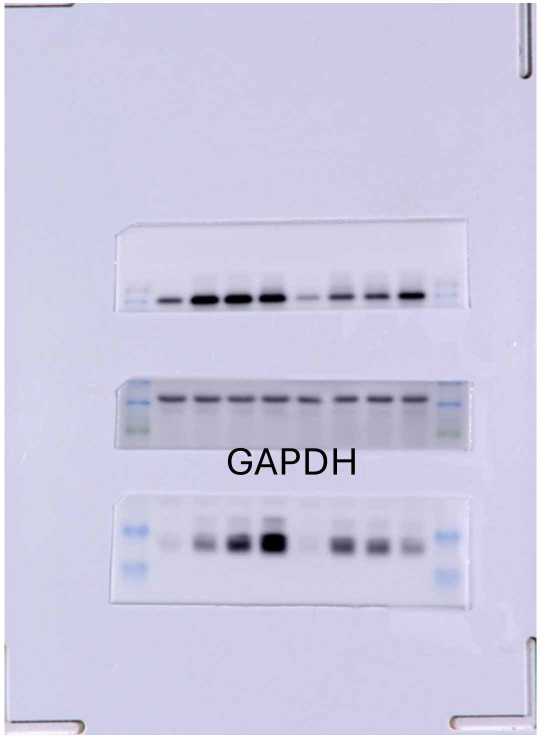

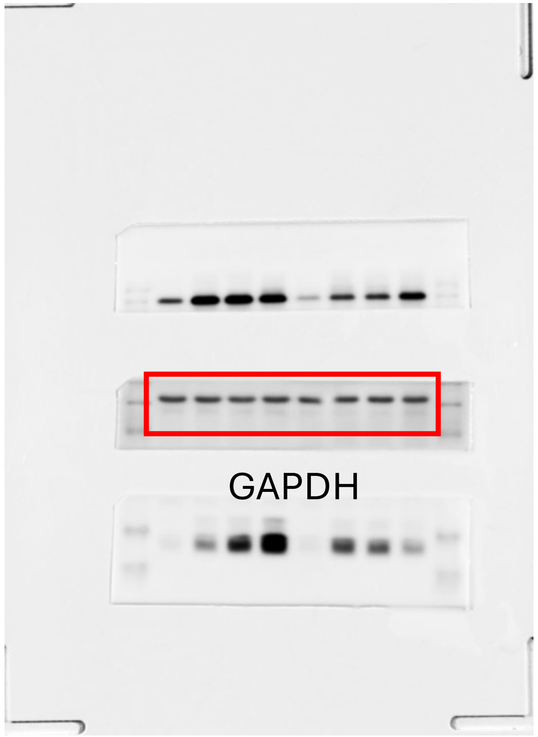

2022.7.13 RT4 n=1
BGJ398-PD-L1, GAPDH

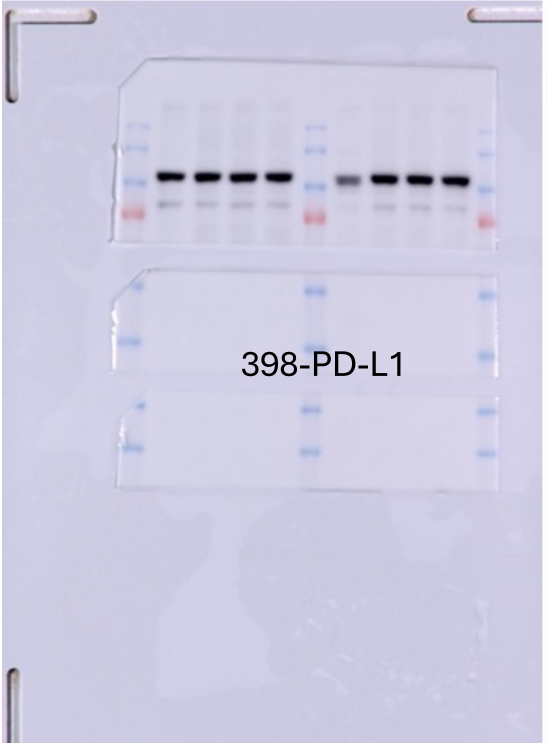

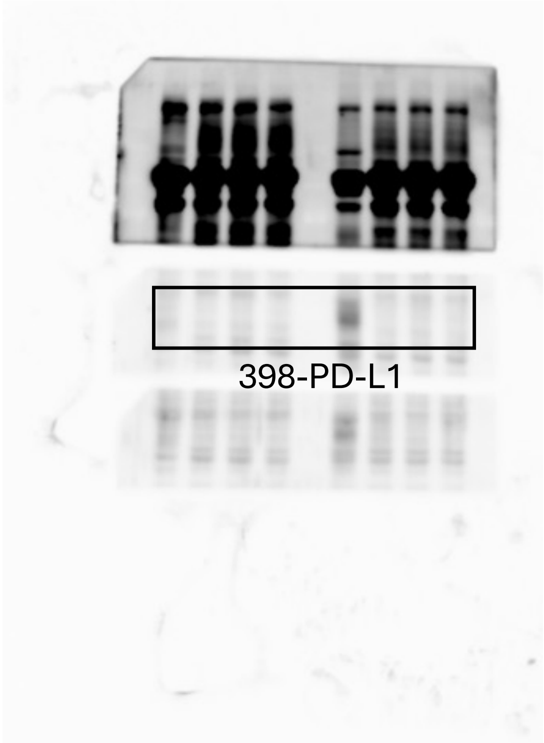

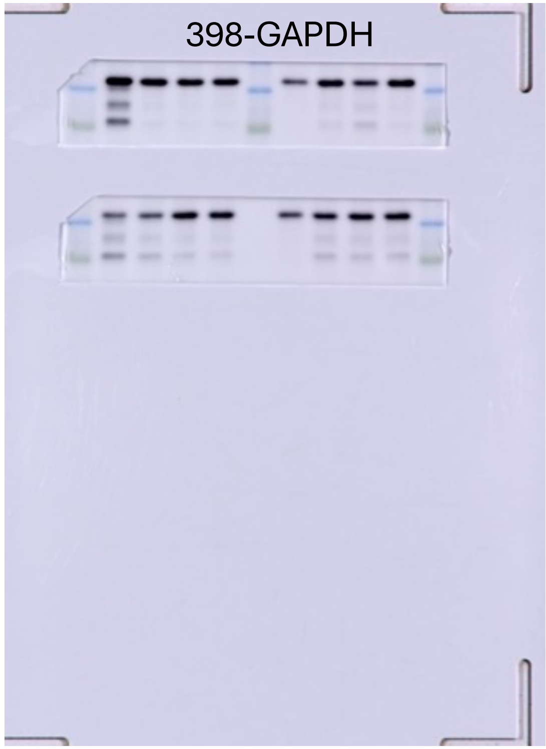

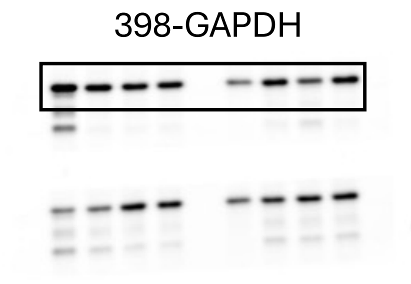

2022.8.15 RT4 n=2

BGJ398-PD-L1, GAPDH

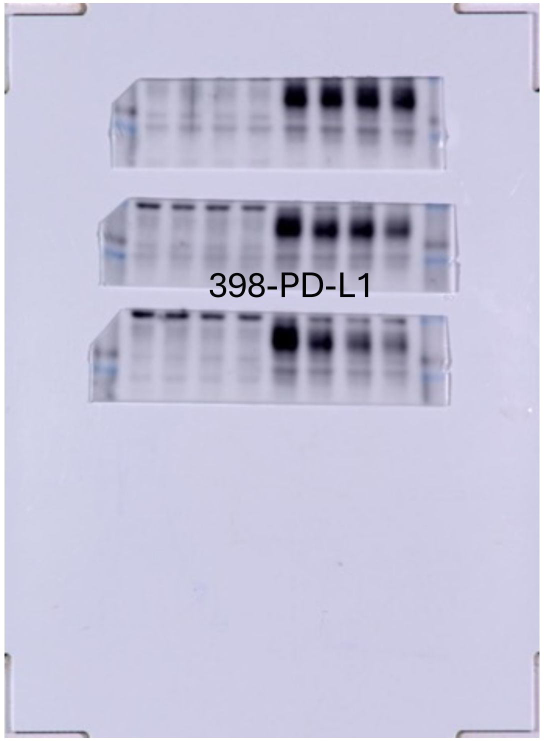

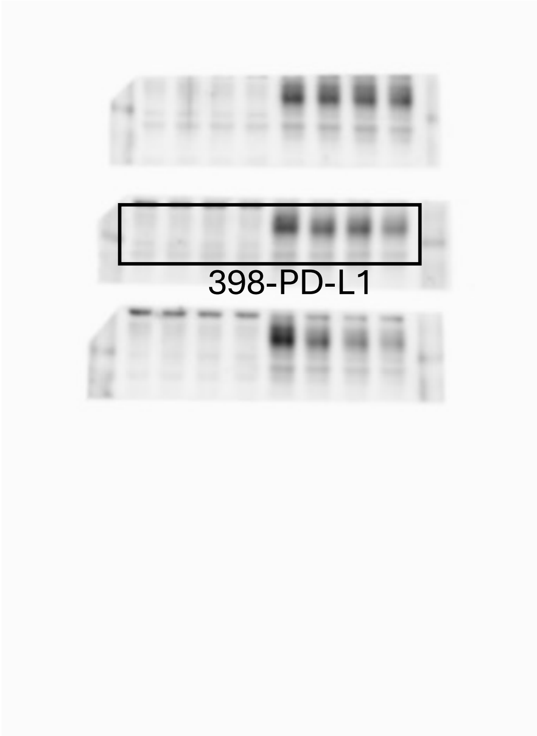

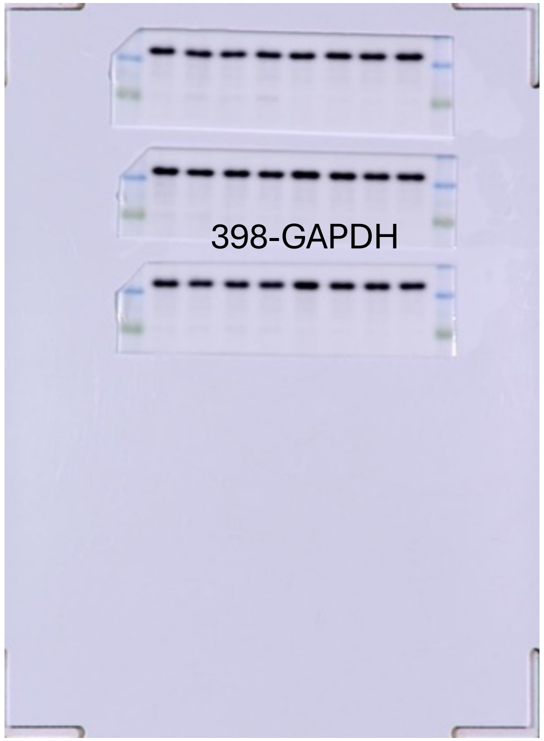

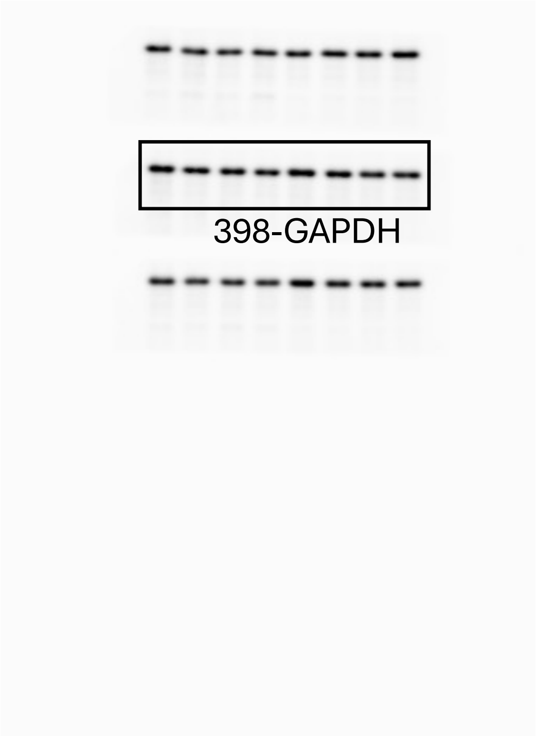

F2A RT4 (BGJ398)

2022.9.14 RT4 n=3

BGJ398-PD-L1, GAPDH

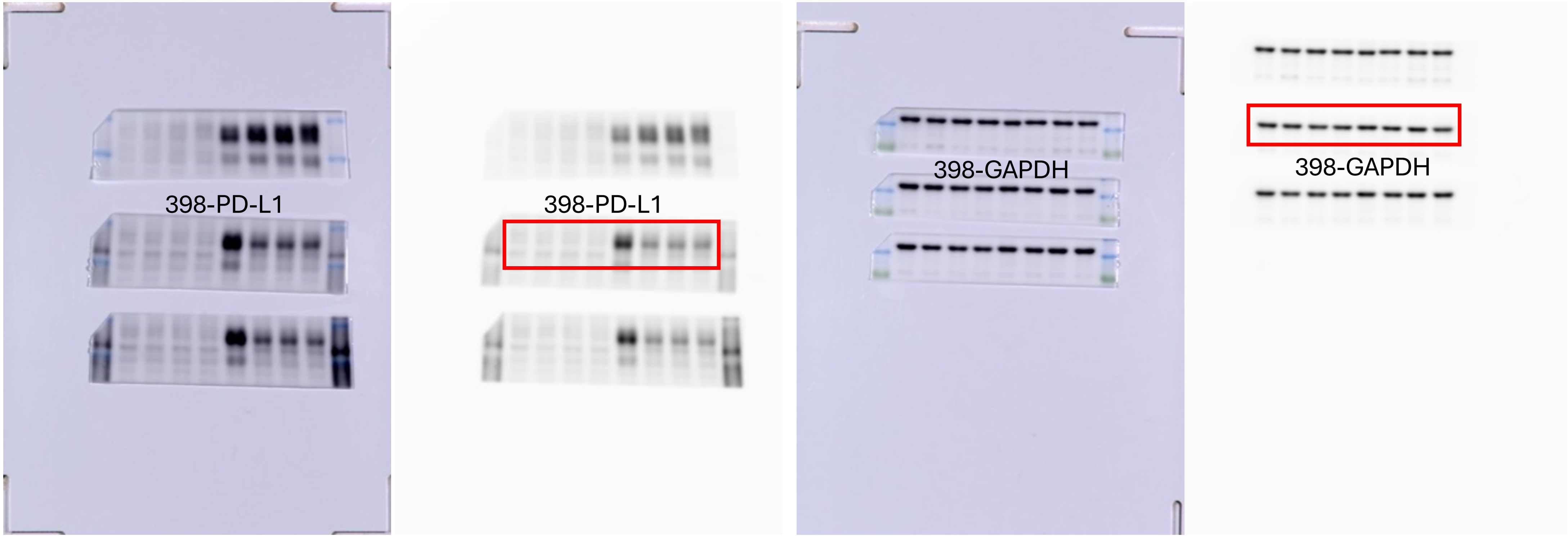

F2B RT-112 time course (L145)

2022.4.19 RT112 n=1

L145-PD-L1,GAPDH

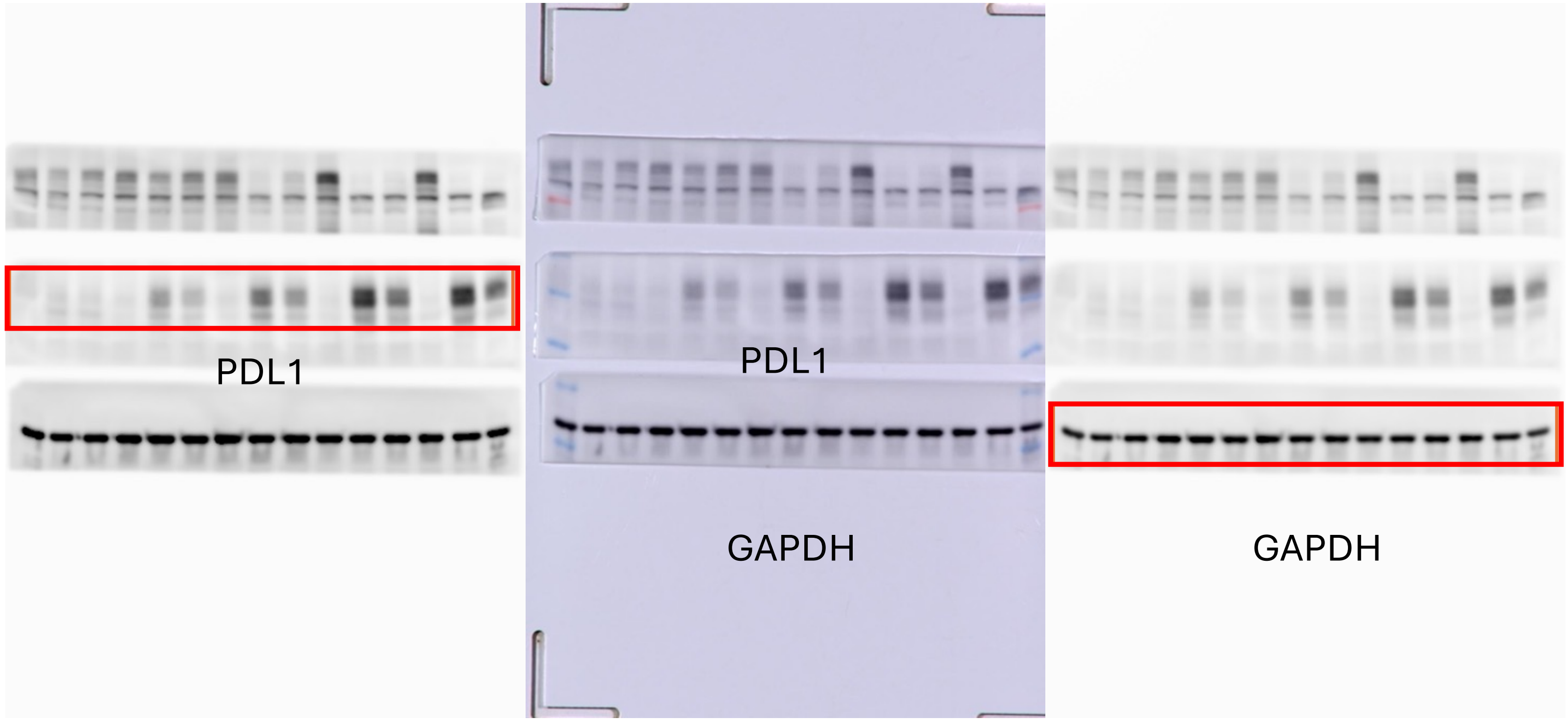

F2B RT-112 time course (BGJ398)

2022.4.19 RT112 n=1

BGJ398-PD-L1,GAPDH

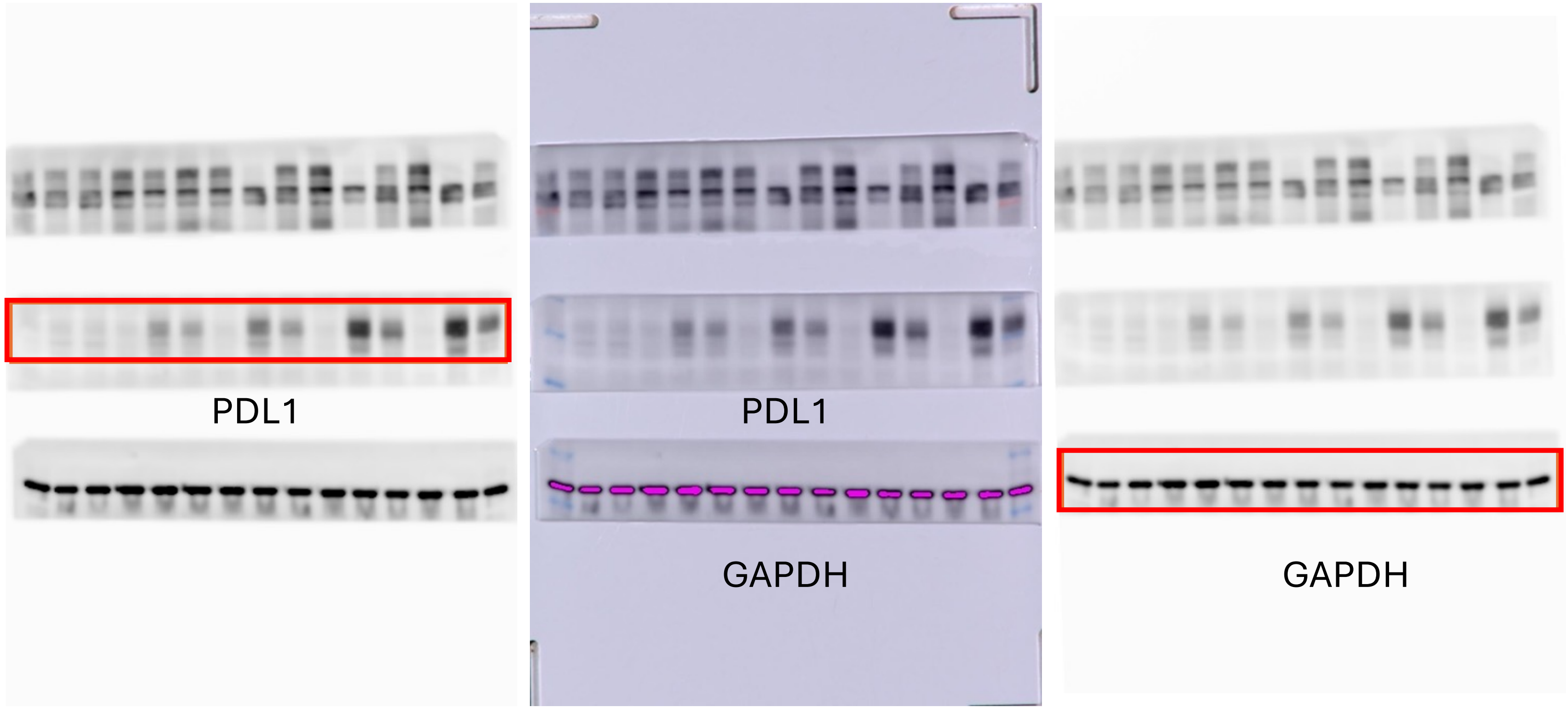

2022.5.20 RT112 n=2
L145-PD-L1,GAPDH

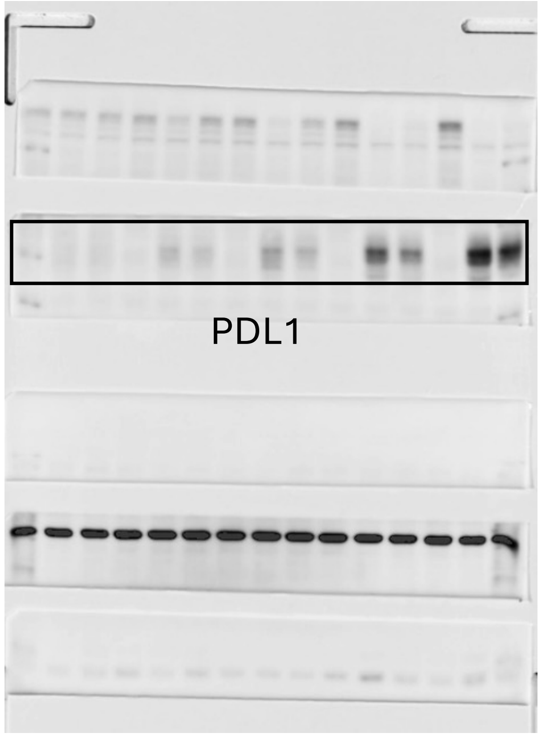

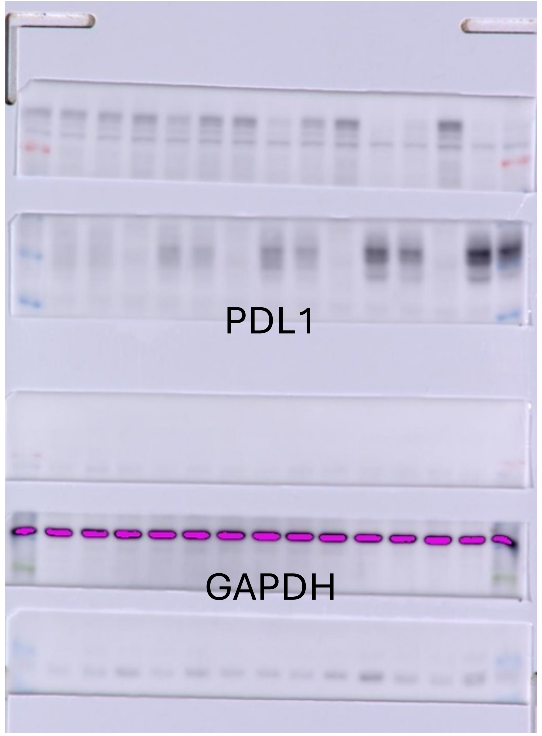

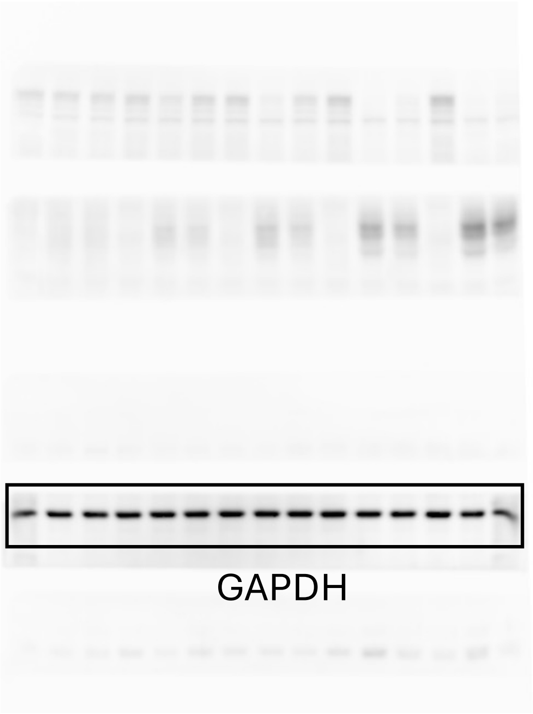

2022.5.20 RT112 n=2
BGJ398-PD-L1,GAPDH

2022.6.9 補 GAPDH

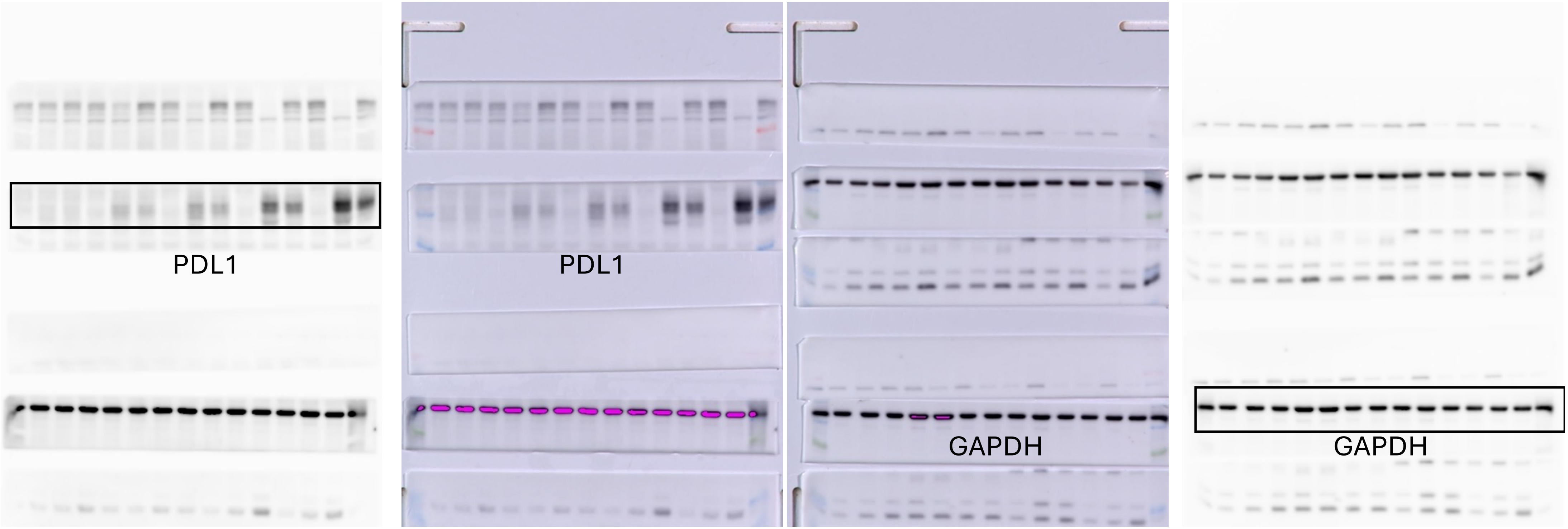

2022.6.17 RT112 n=3
L145-PD-L1,GAPDH

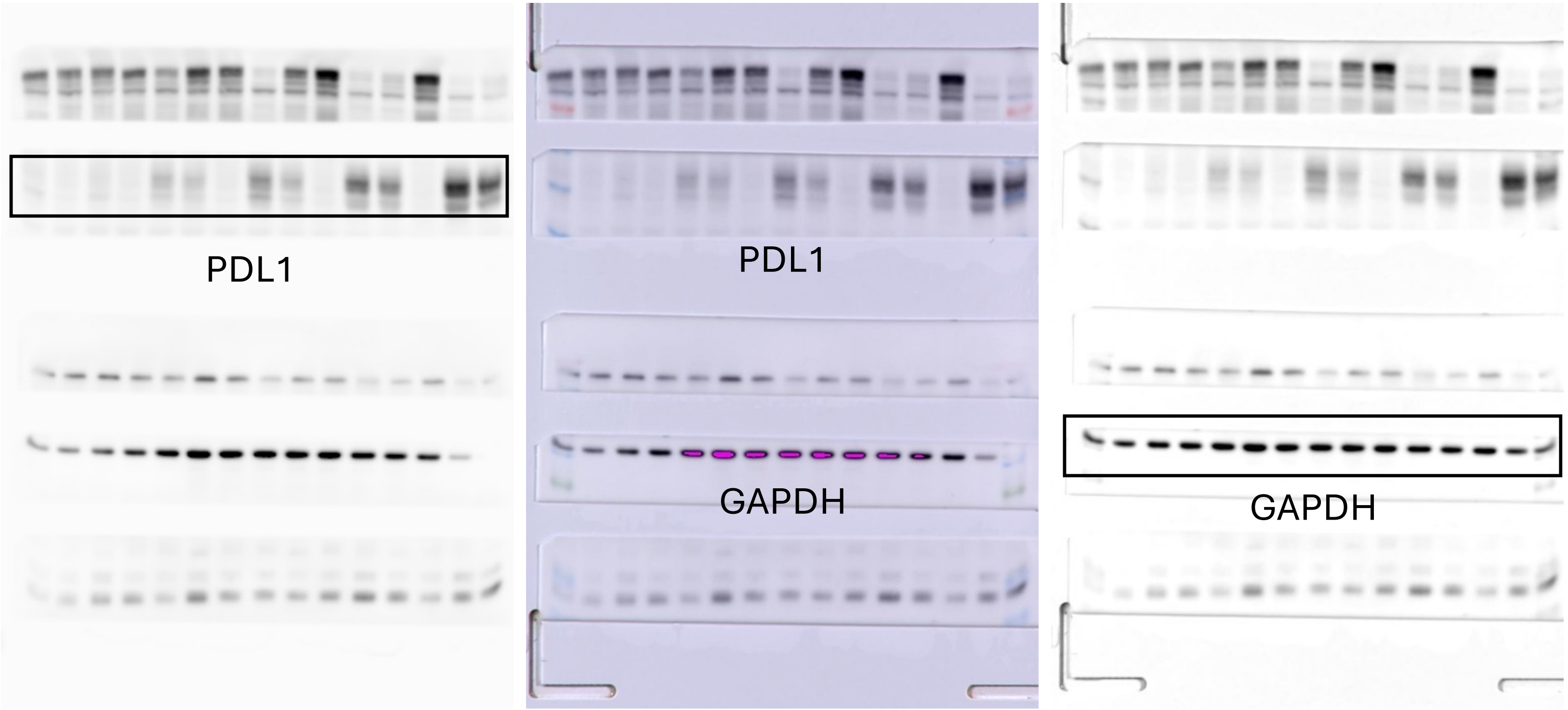

2022.6.17 RT112 n=3
BGJ398-PD-L1,GAPDH

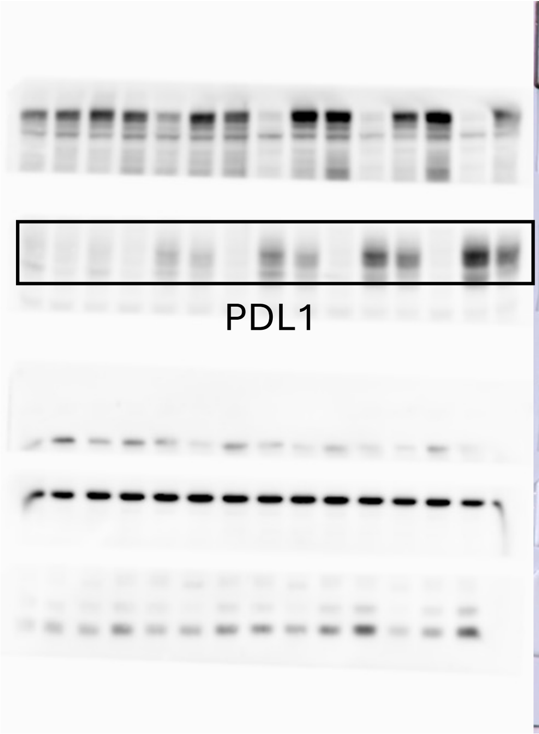

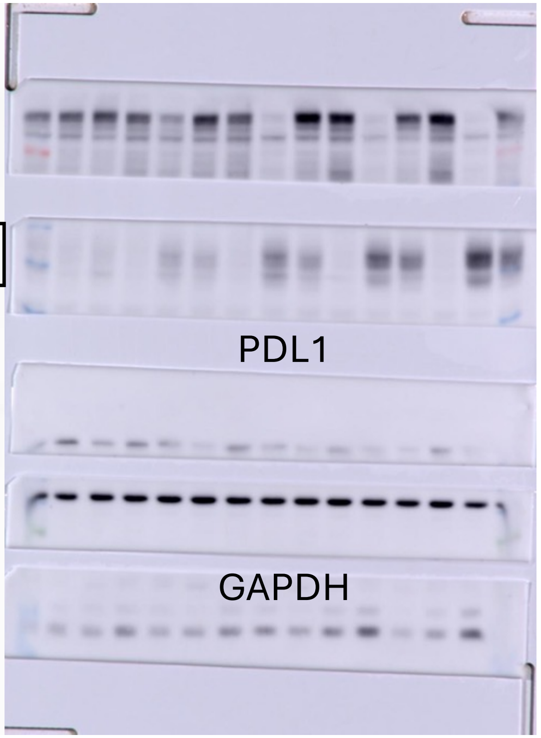

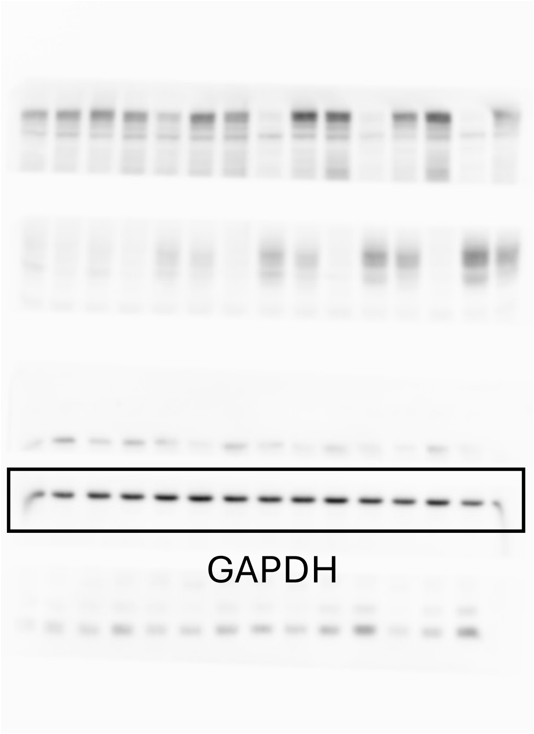

F2E RT-112-PIK3C3 KD

2019.02.20 RT112-PIK3C3 KD n=1

VPS34, PD-L1, p62, LC3, GAPDH

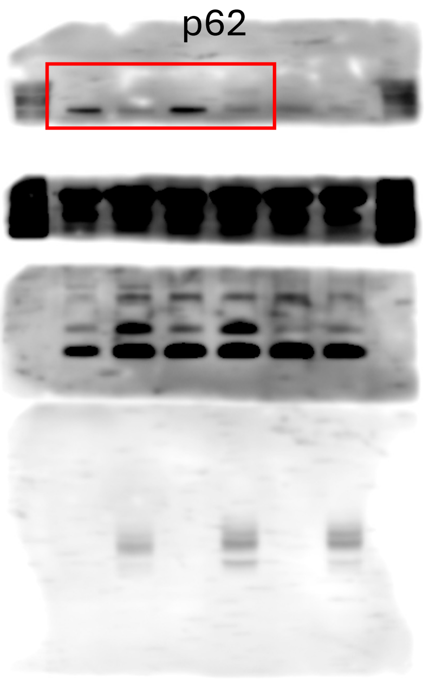

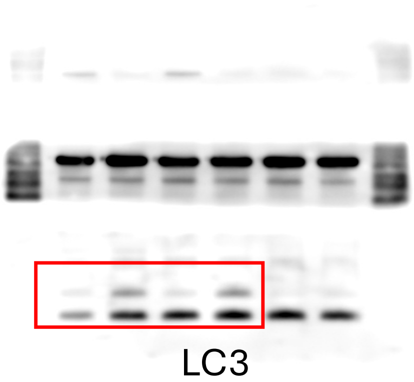

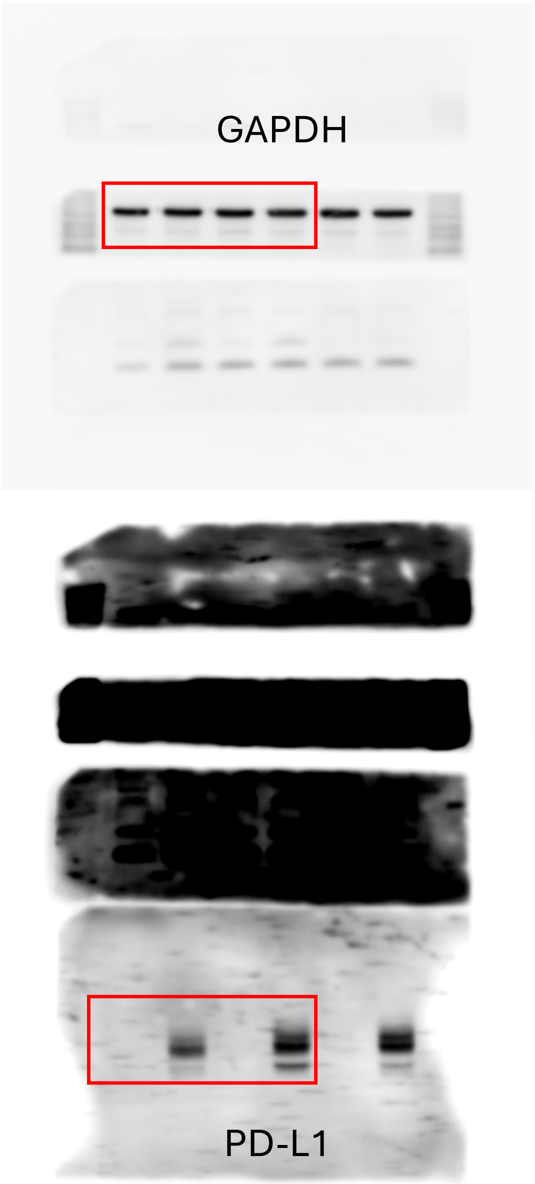

2019.02.26

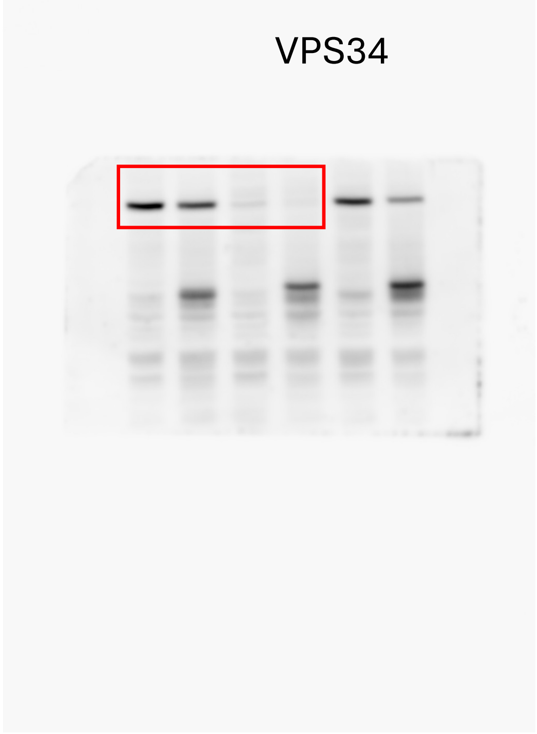

2024.10.17 RT112-PIK3C3 KD n=2
VPS34, PD-L1, p62, LC3, GAPDH

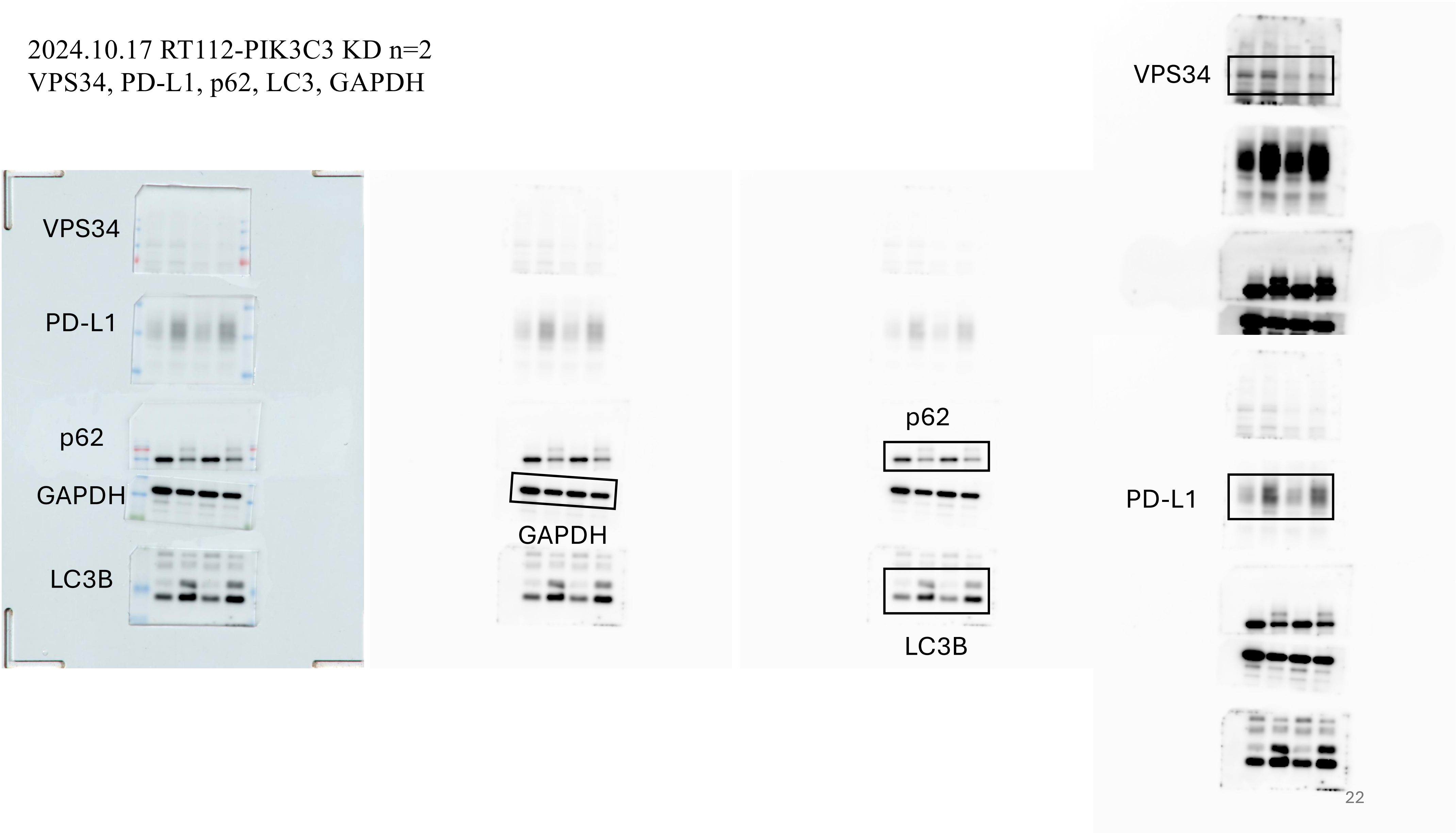

2024.10.17 RT112-PIK3C3 KD n=3
VPS34, PD-L1, p62, LC3, GAPDH

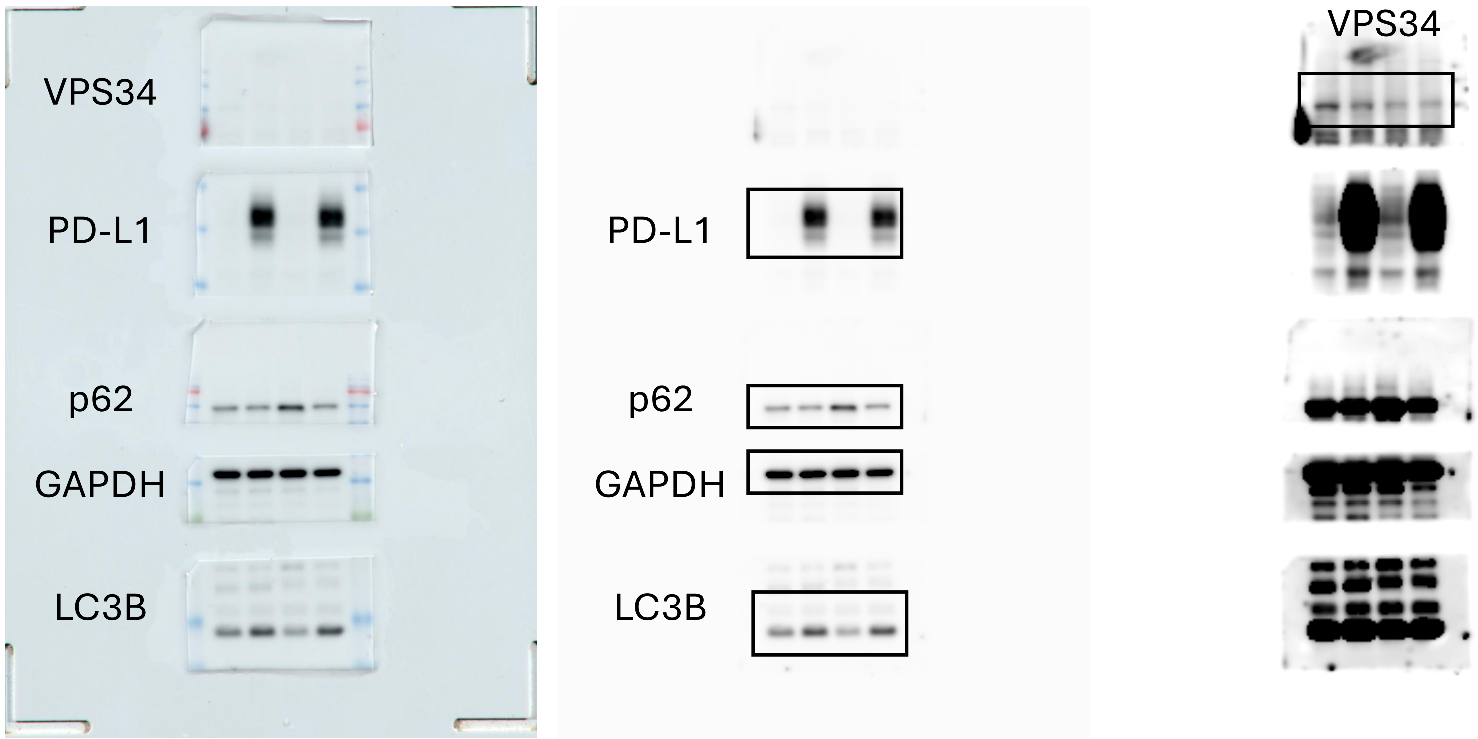

F2F RT-112 (SAR405)

2019.01.23 RT-112 (SAR405) n=1

PD-L1, p62, LC3, GAPDH

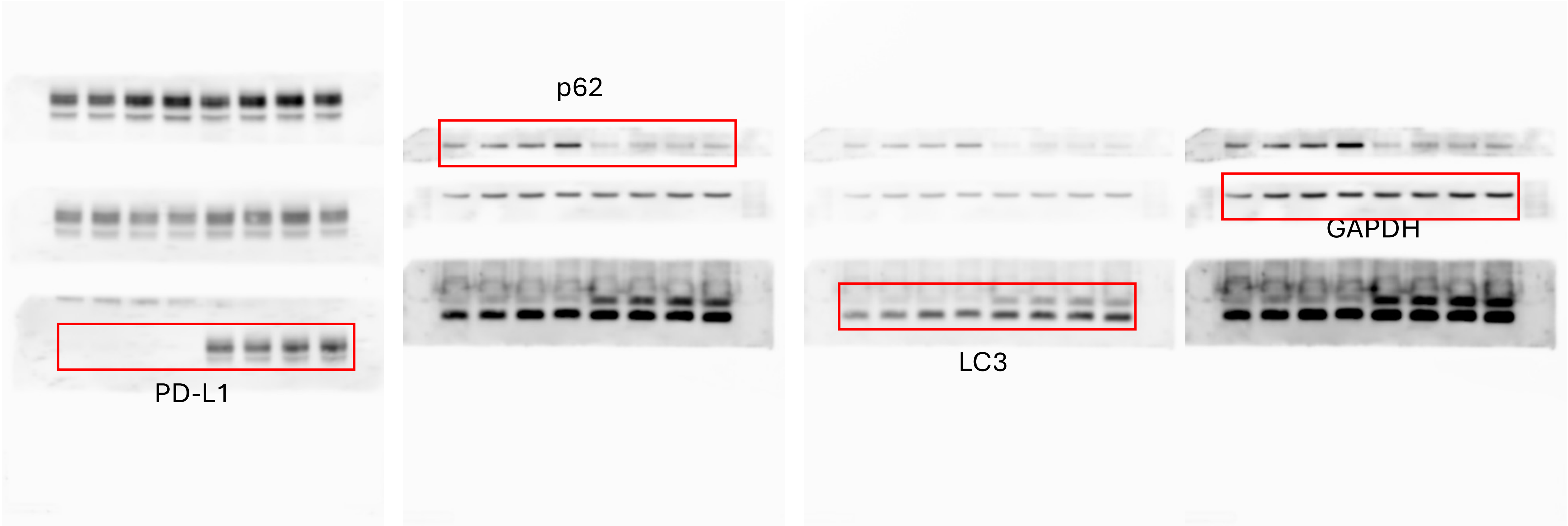

2019.01.28 RT-112 (SAR405) n=2
PD-L1, p62, LC3, GAPDH

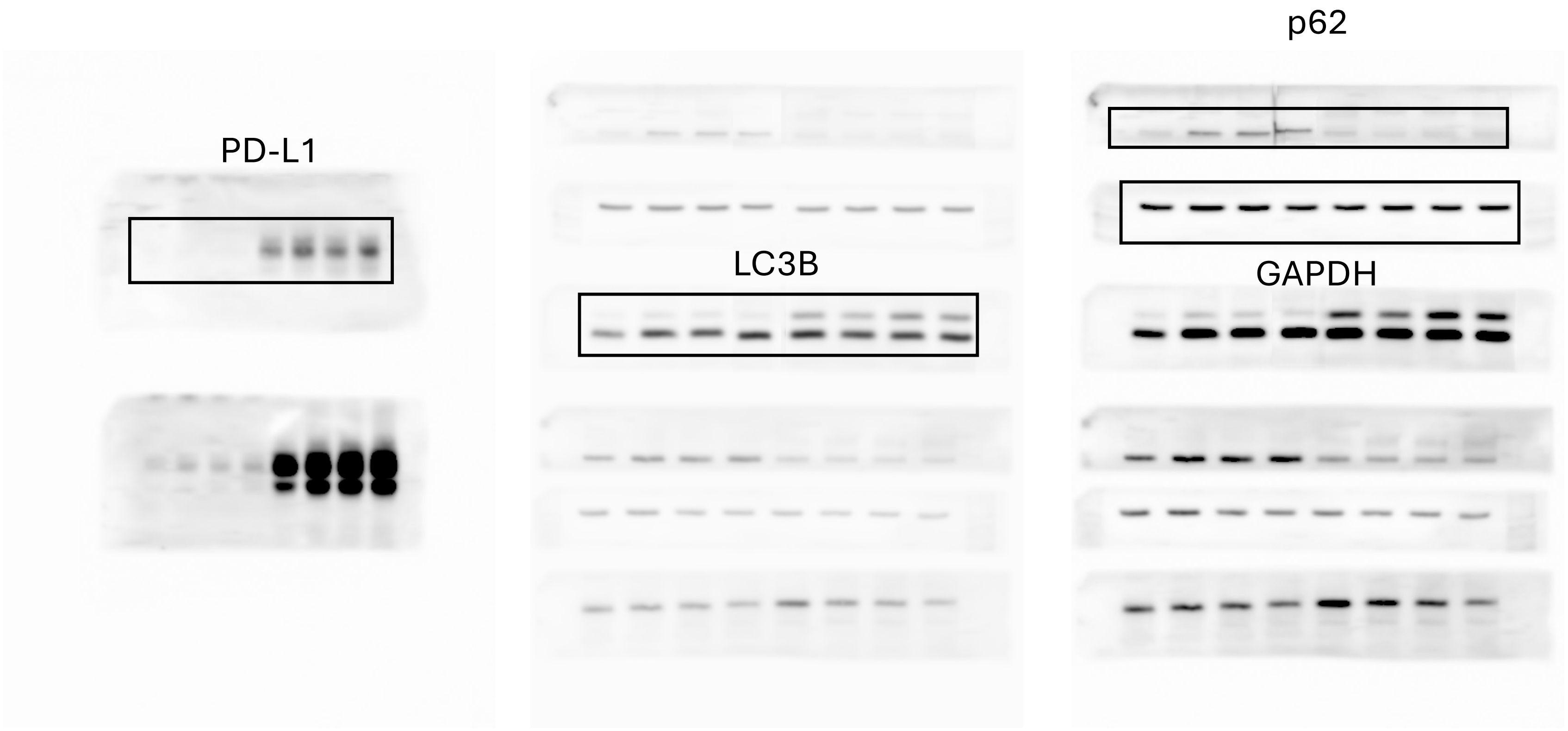

2024.10.15 RT112 (SAR405) n=3
PD-L1, p62, LC3, GAPDH

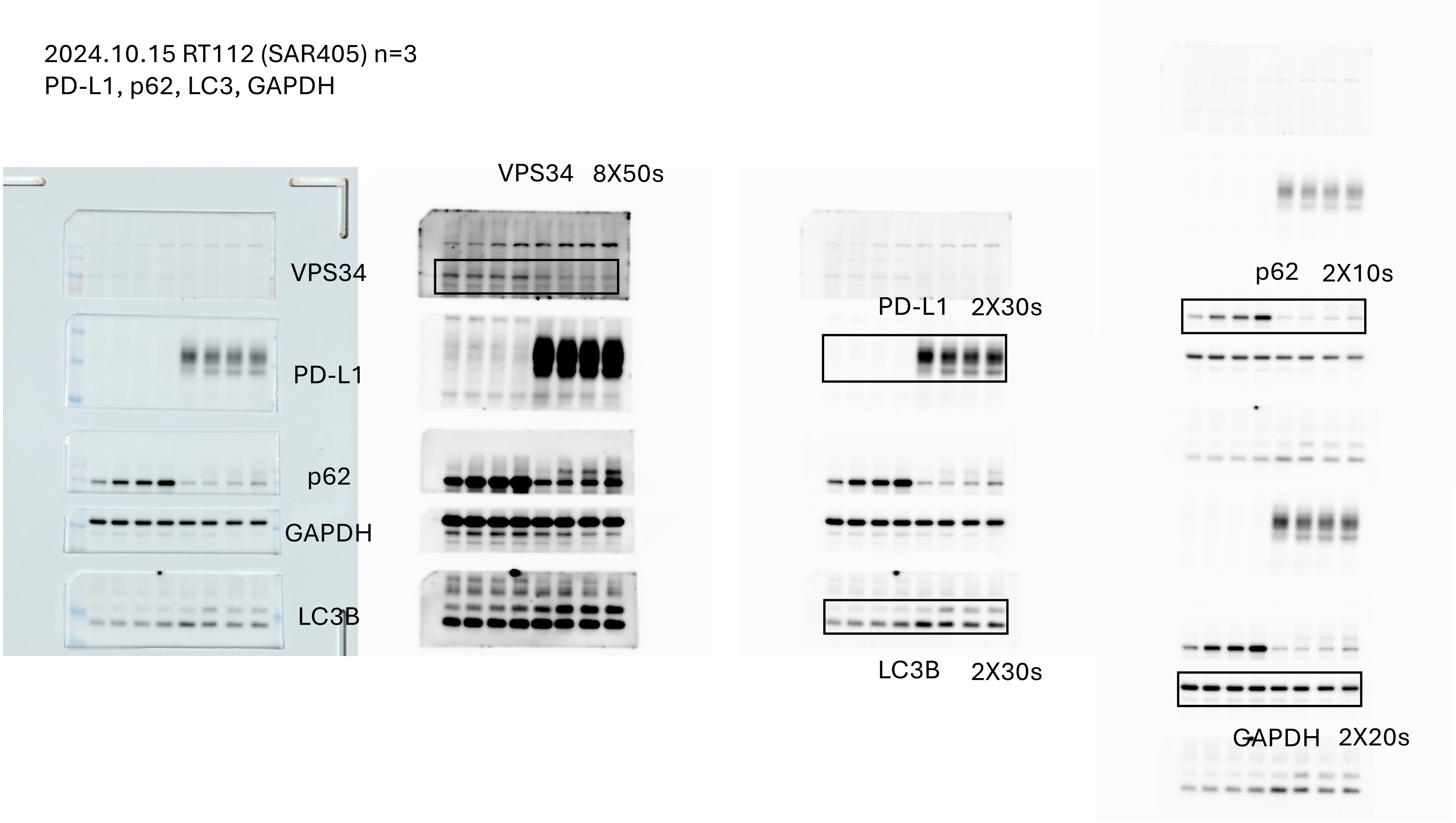

F2G RT-112 (FGFR3-TACC3 KO)

2023.05.19 RT112-FGFR3-TACC3 KO n=1

FGFR3, PD-L1, p62, LC3, GAPDH

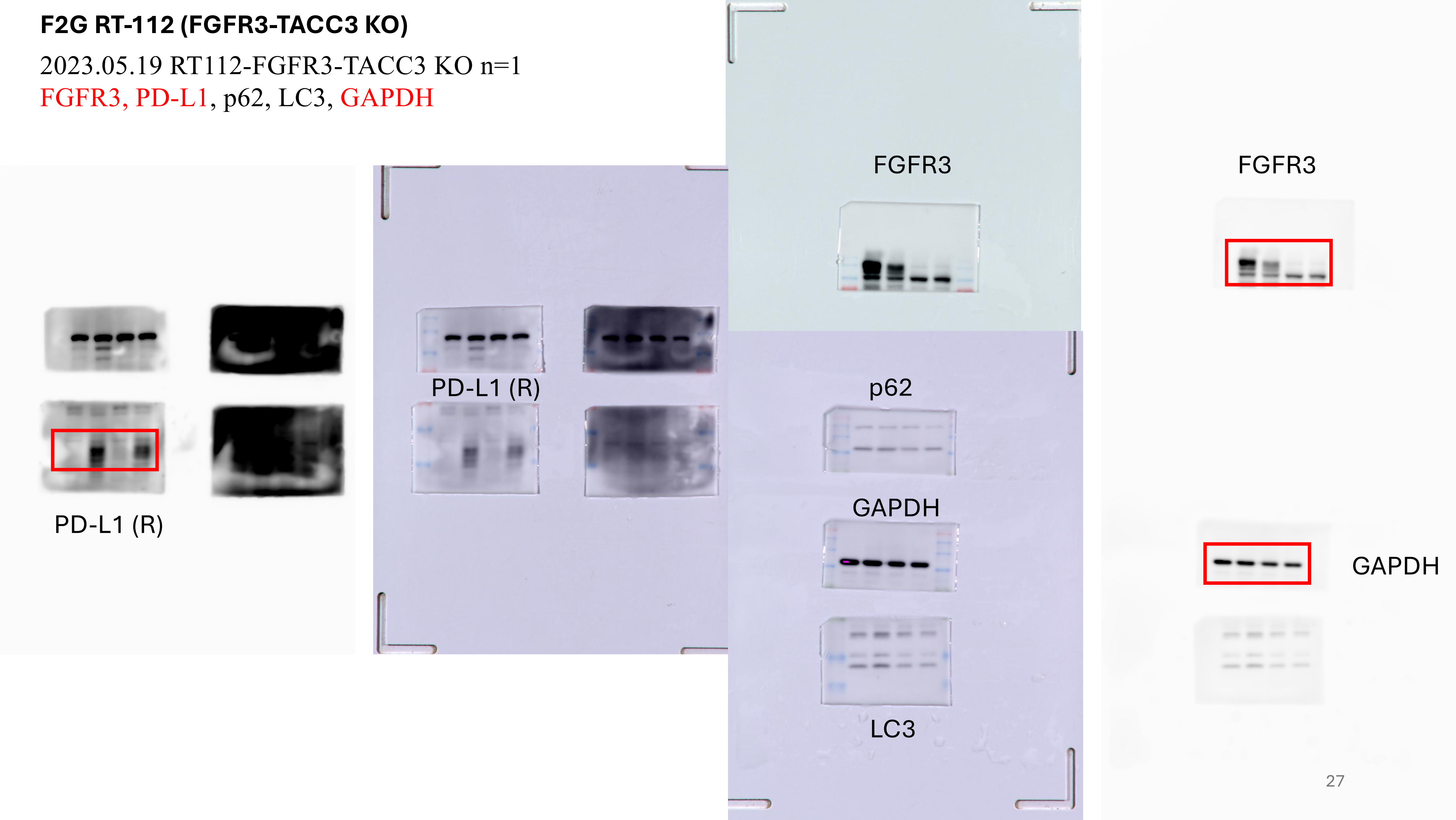

2023.06.07 RT112-FGFR3-TACC3 KO n=2
PD-L1, p62, LC3, GAPDH

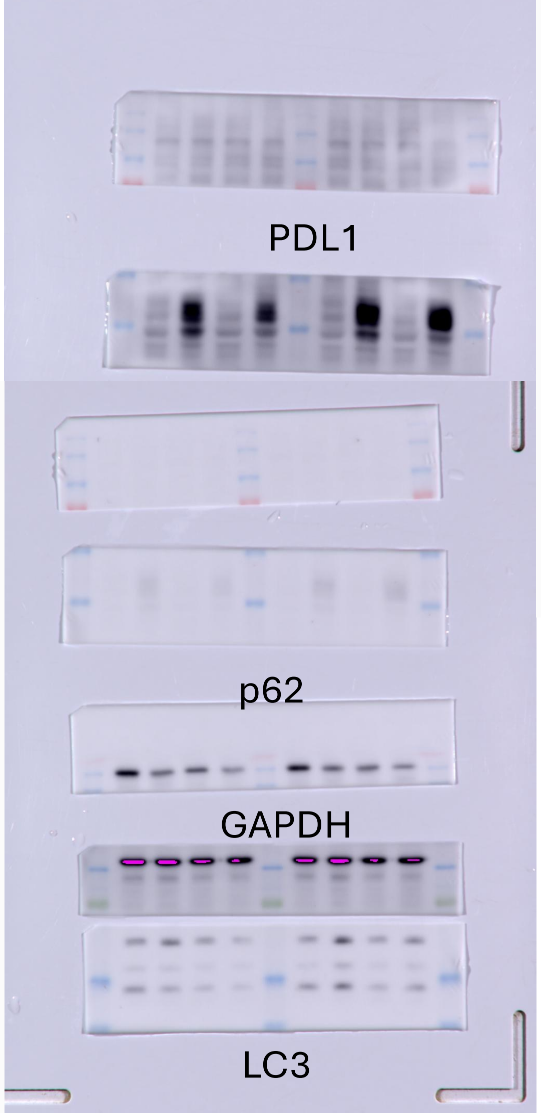

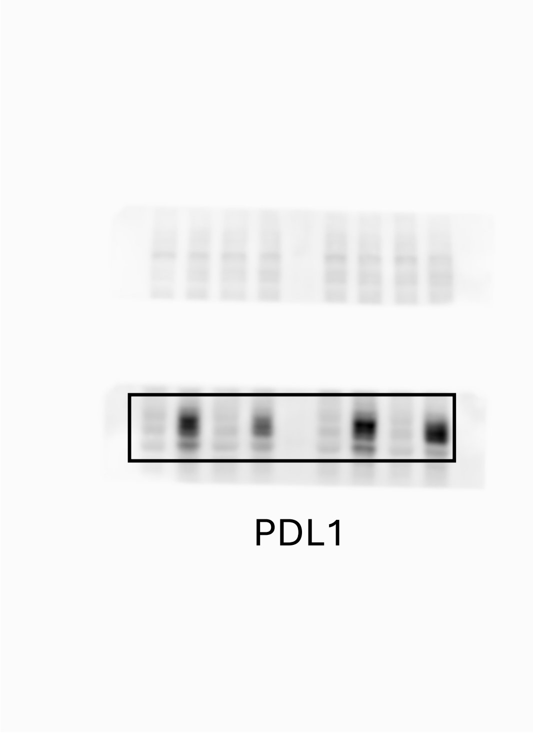

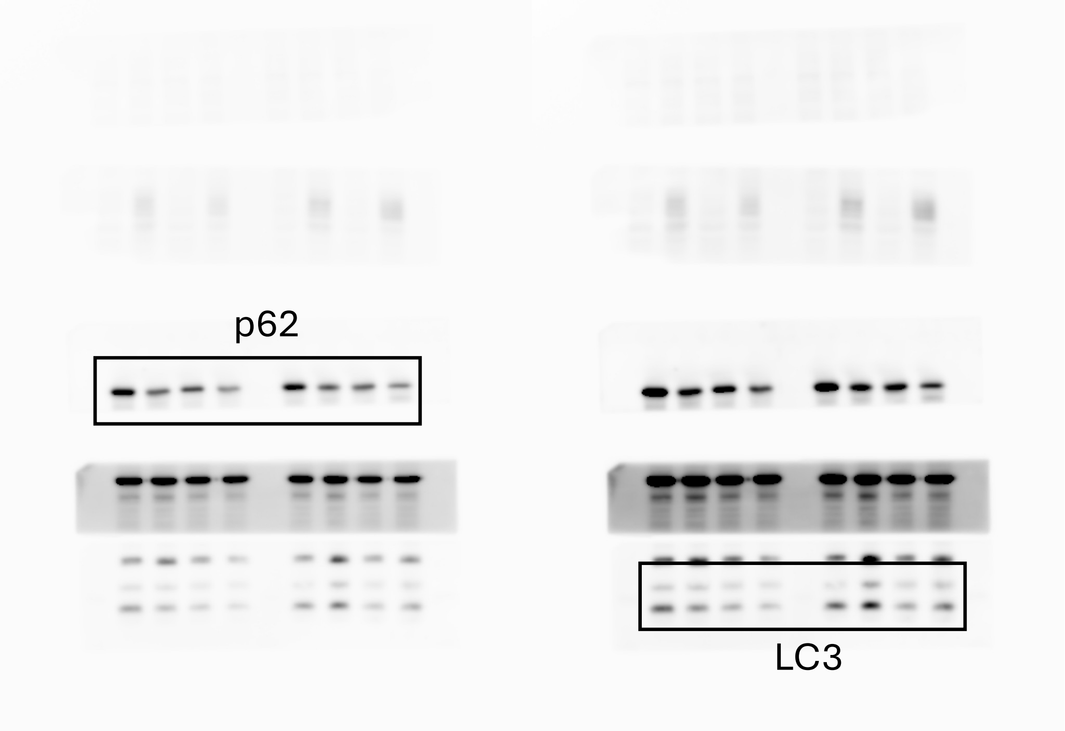

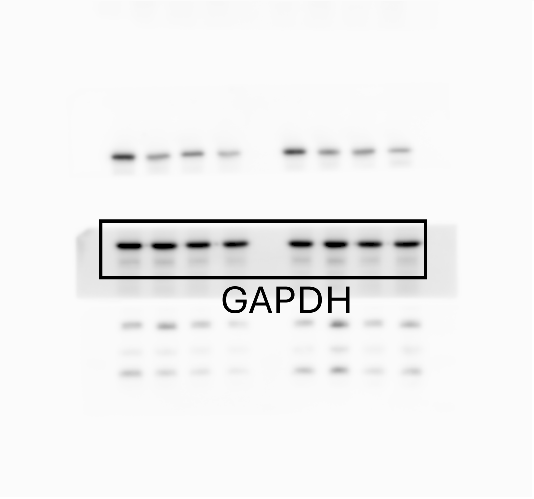

2023.06.13
RT112-FGFR3-TACC3 KO n=3
PD-L1, p62, LC3, GAPDH

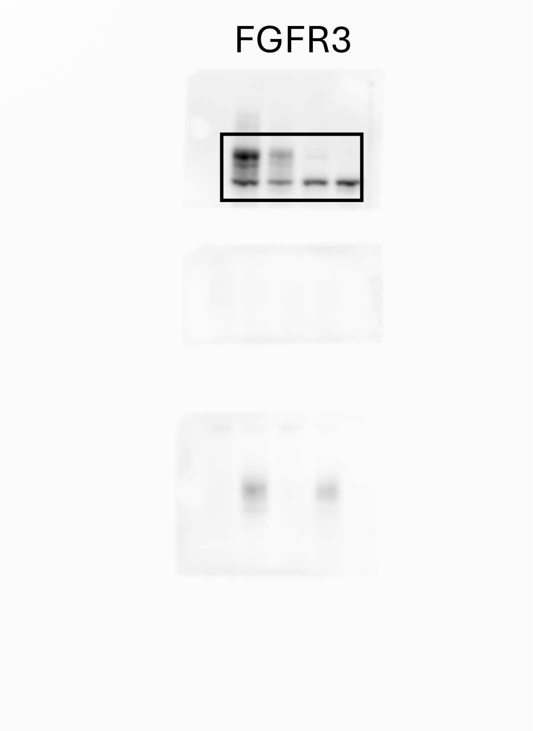

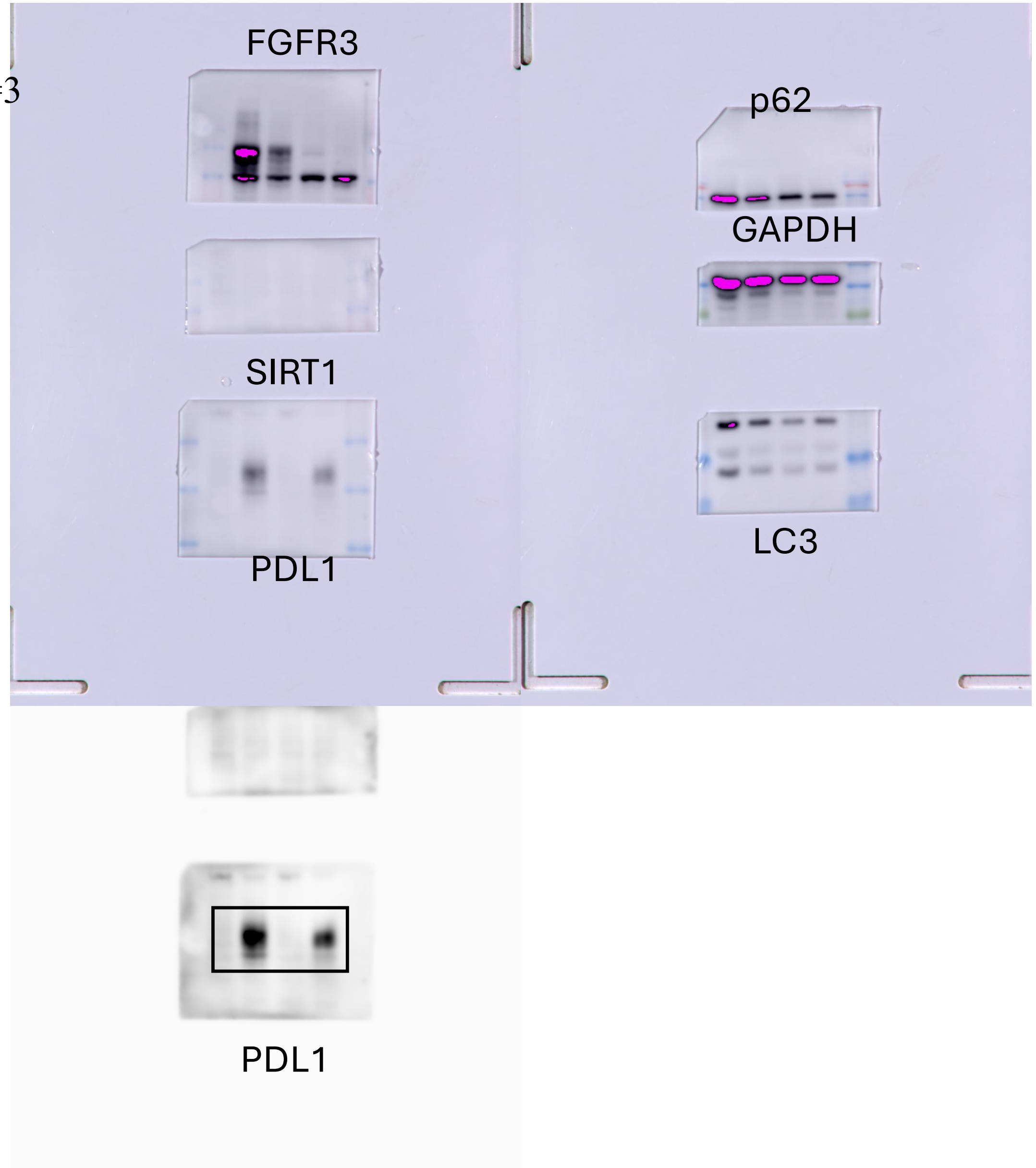

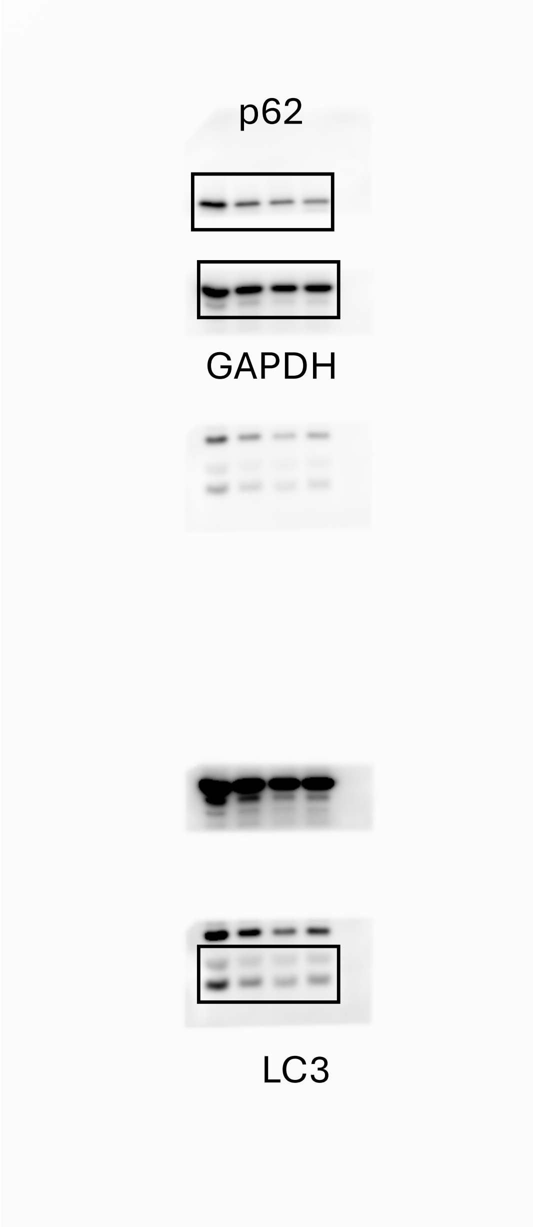

2019.03.29 n=1

L145-PD-L1, p62, LC3B, GAPDH

BGJ398-PD-L1, p62, LC3B, GAPDH

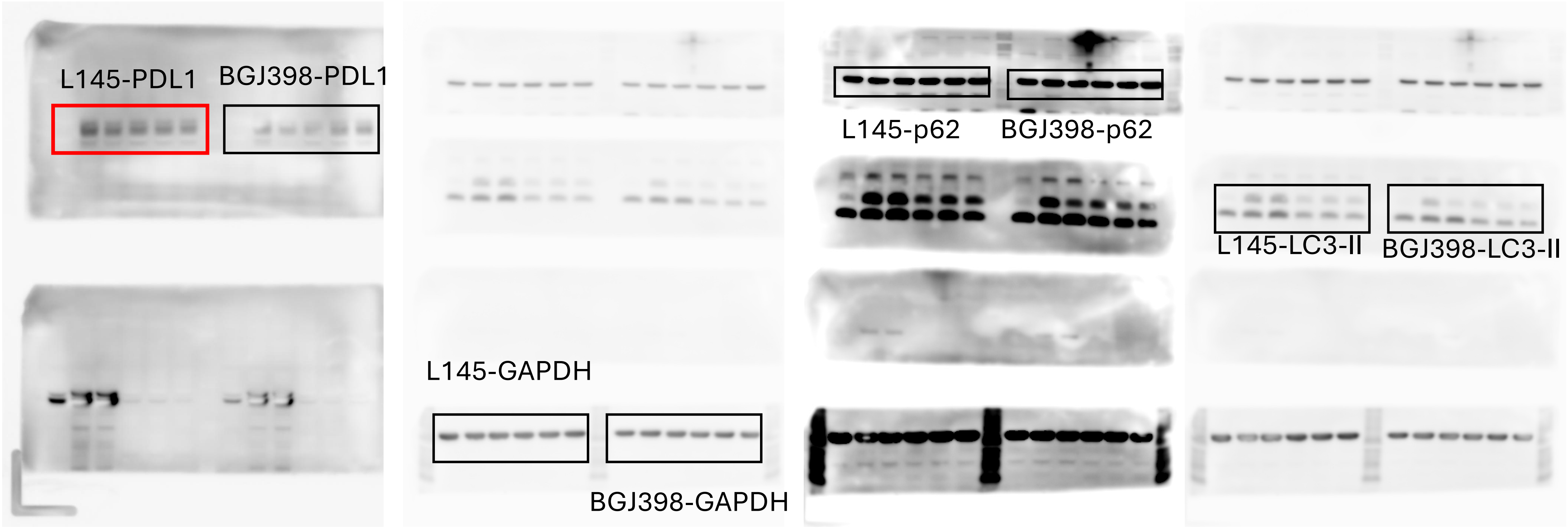

F3A RT112 (MG132)
2019.04.10 n=2
L145-PD-L1, p62, LC3B, GAPDH
BGJ398-PD-L1, p62, LC3B, GAPDH

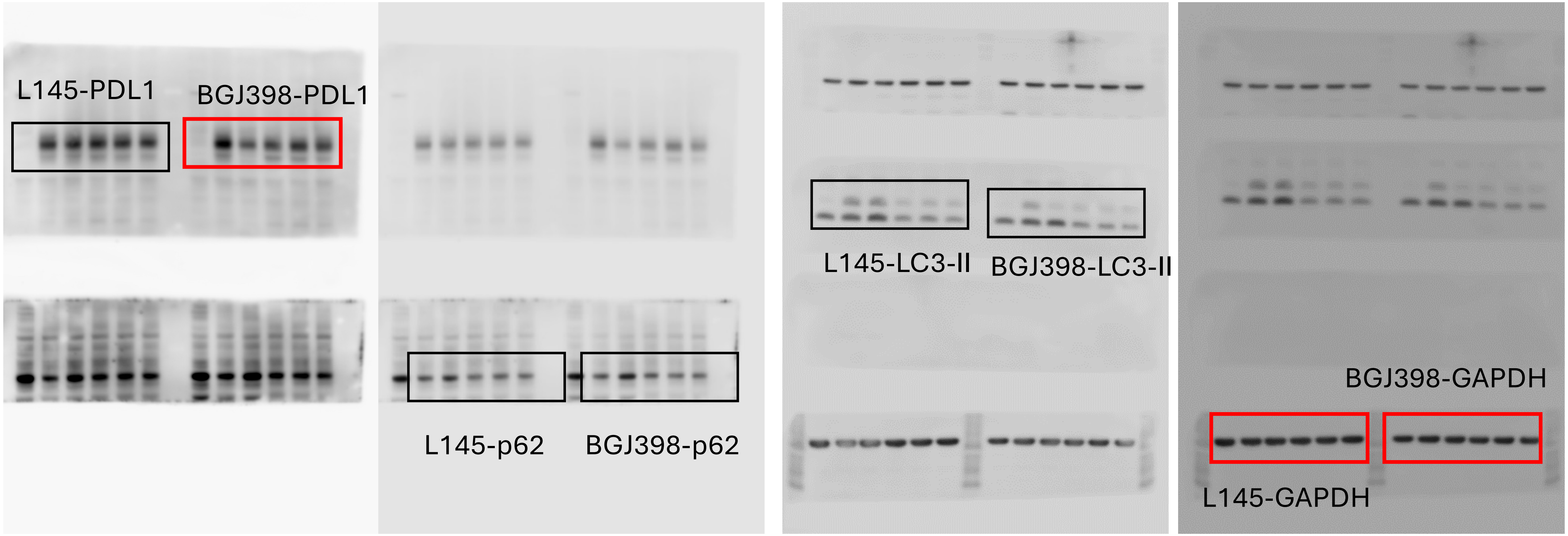

F3A RT112 (MG132)
2024.09.10 n=3
L145-MCL-1, PD-L1, GAPDH
BGJ398-MCL-1, PD-L1, GAPDH

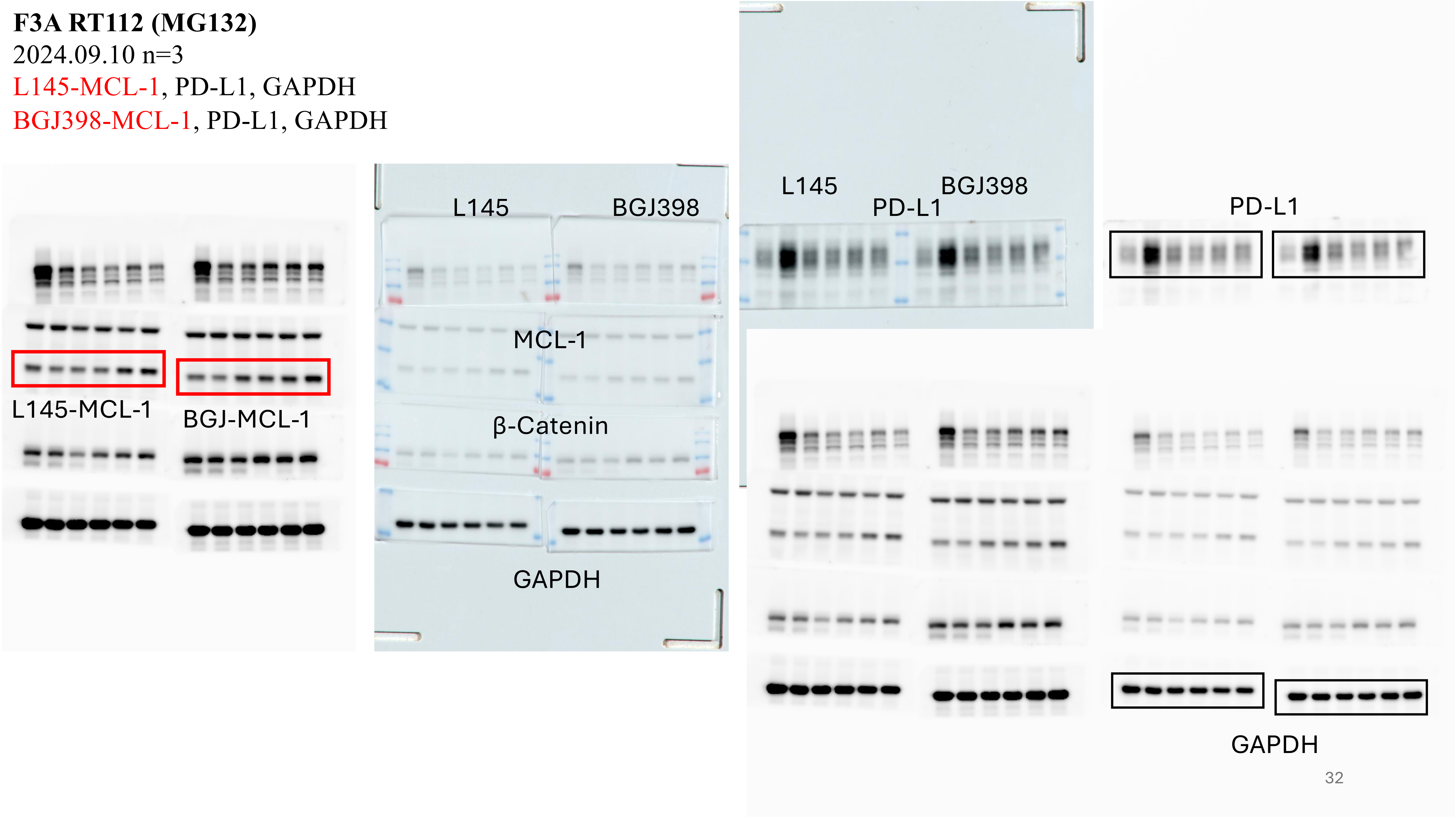

2024.09.10 MCL-1 n=2, 3
L145-MCL-1, GAPDH
BGJ398-MCL-1, GAPDH

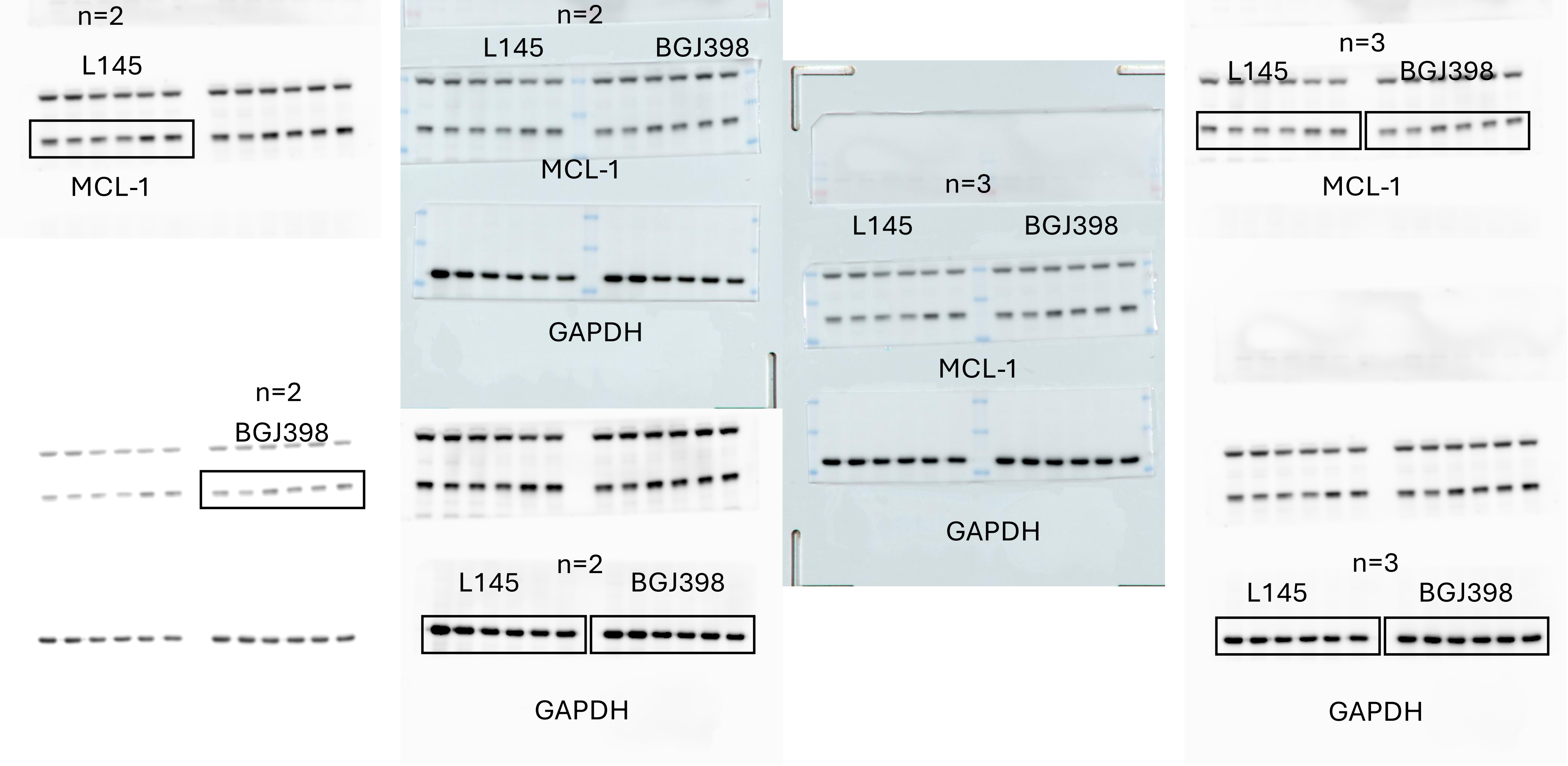

RT112 (L145-CQ)

2022.12.29 n=1

L145-PD-L1, p62, LC3B, GAPDH

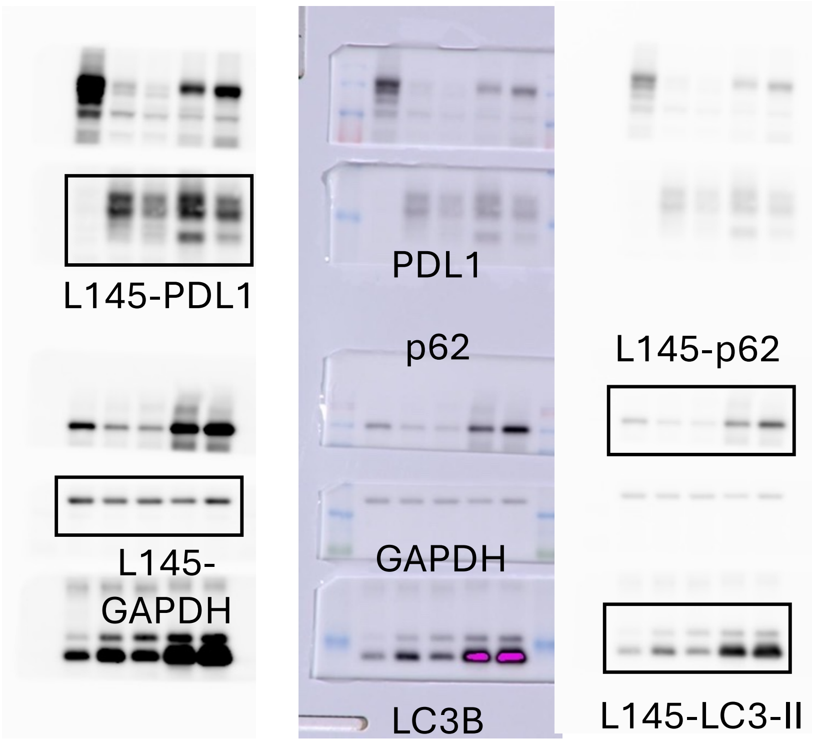

F3B RT112 (L145-CQ)

2022.12.30 n=2

L145-PD-L1, p62, LC3B, GAPDH

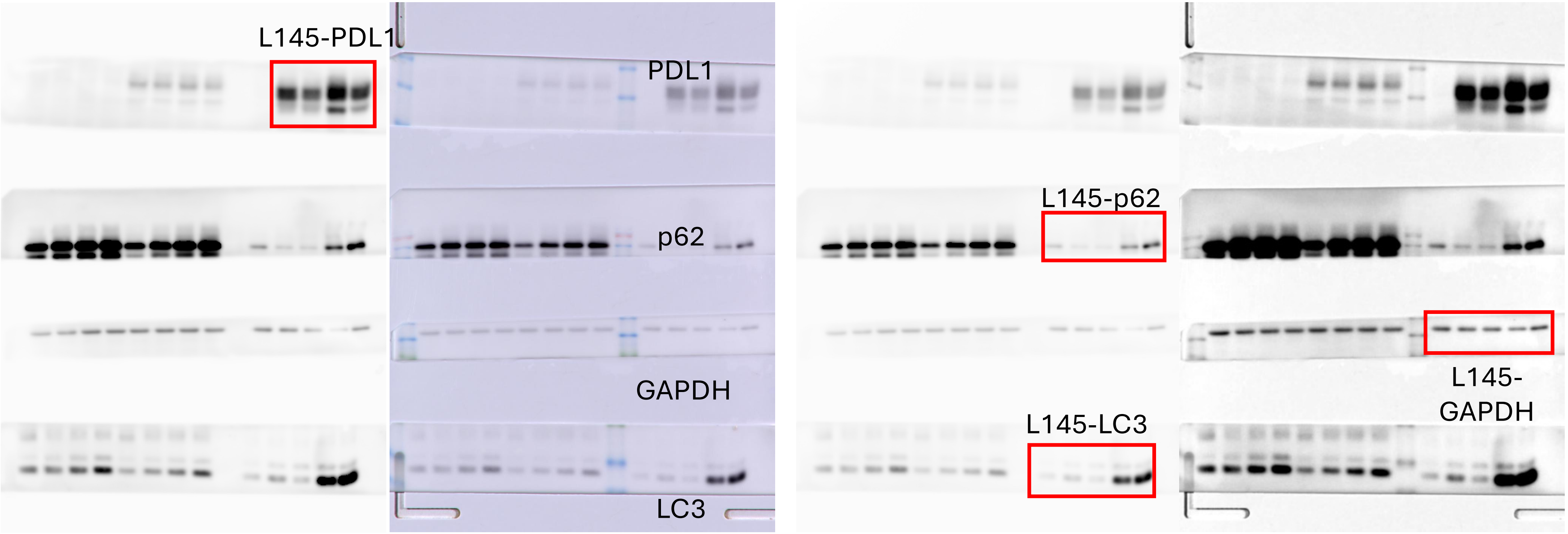

RT112 (L145-CQ)
2023.1.10 n=3
L145-PD-L1, p62, LC3B, GAPDH

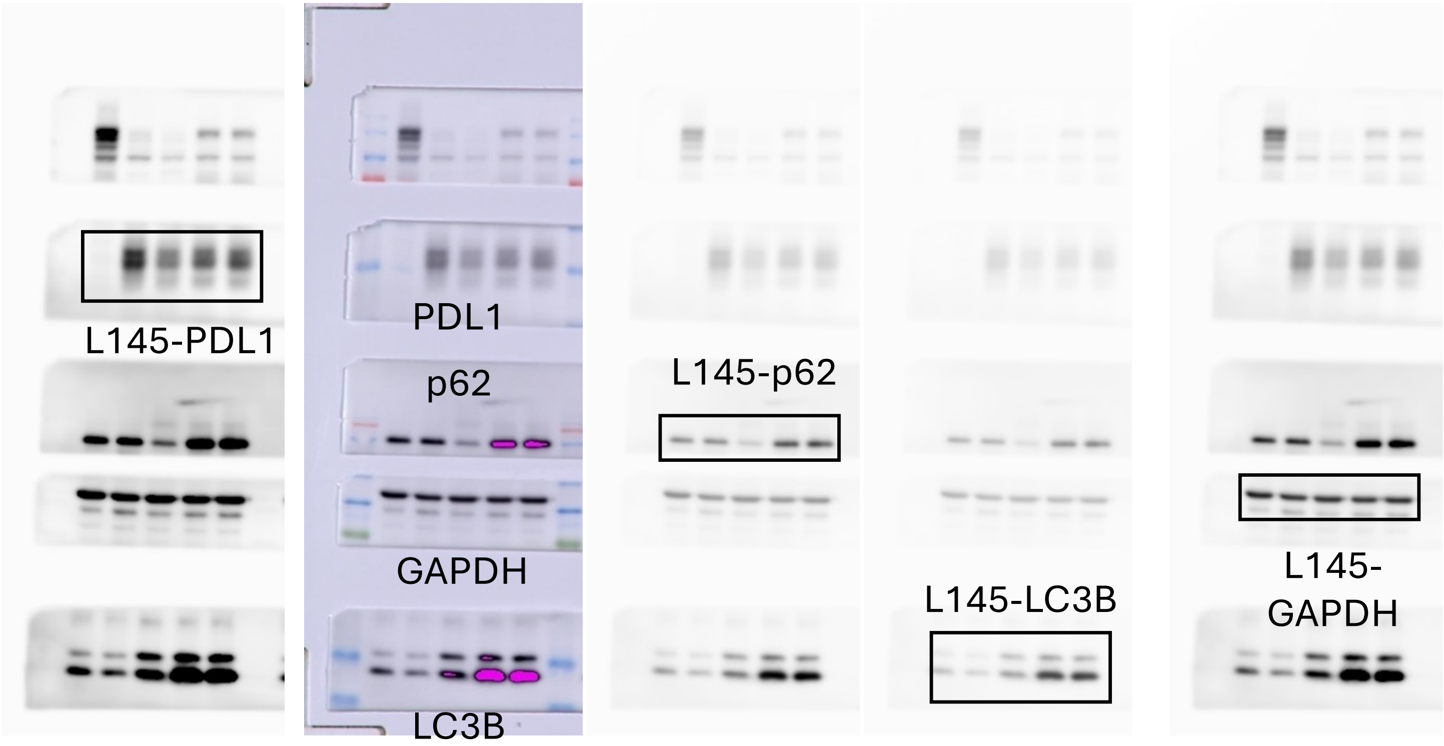

RT-112 (BGJ398-CQ)
2023.2.25 n=1
BGJ398-PD-L1, p62, LC3B, GAPDH

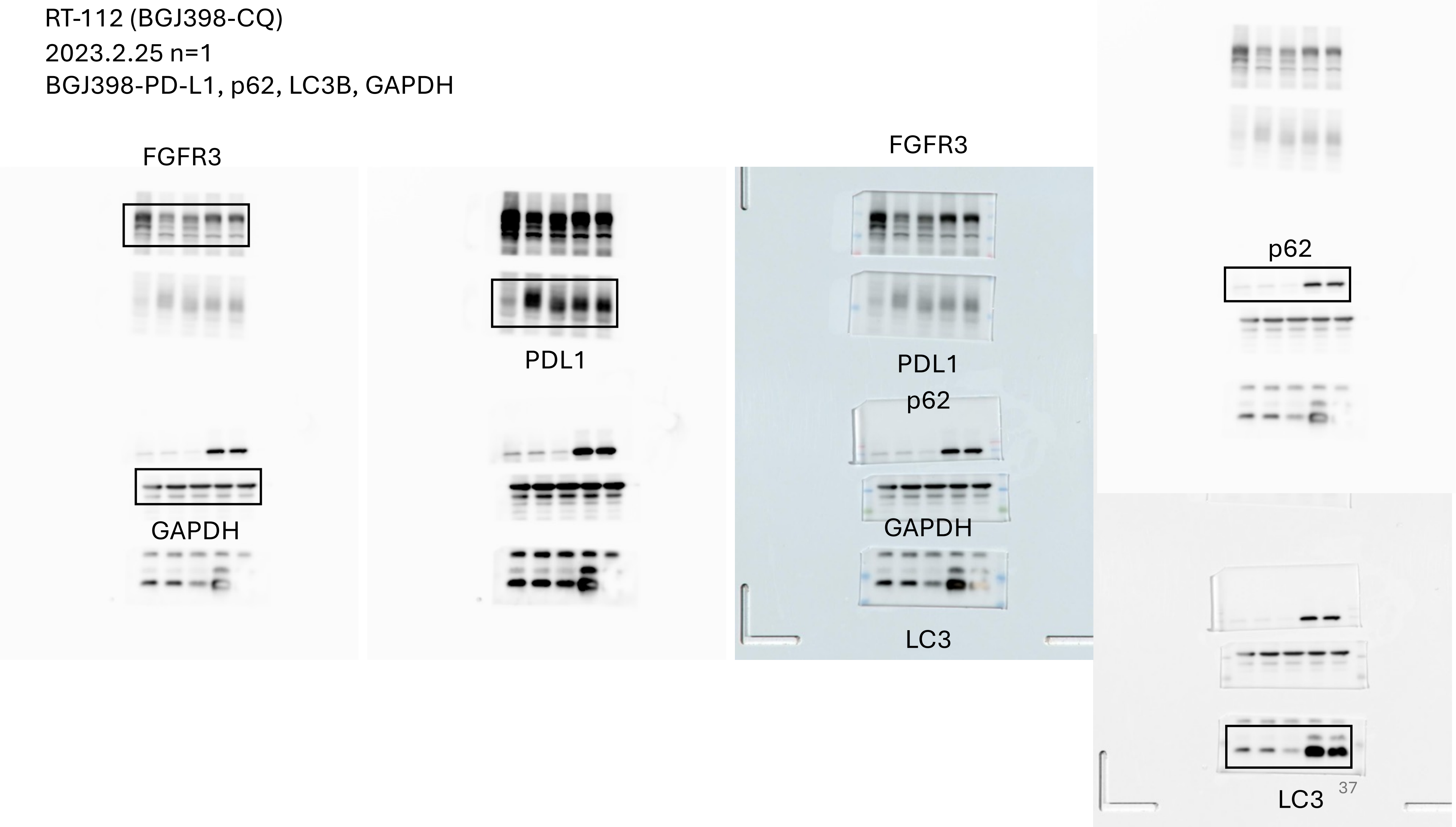

F3B RT112 (BGJ398-CQ)

RT112 (CQ) 2023.3.3 n=2, 3

BGJ398--PD-L1, p62, LC3B, GAPDH

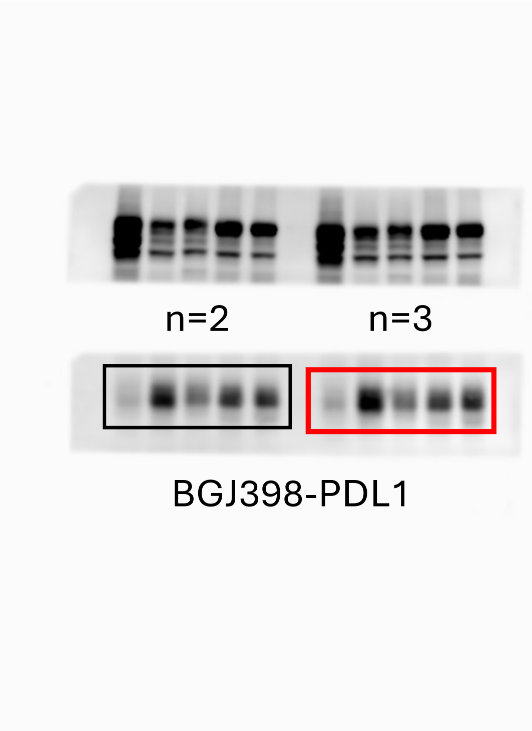

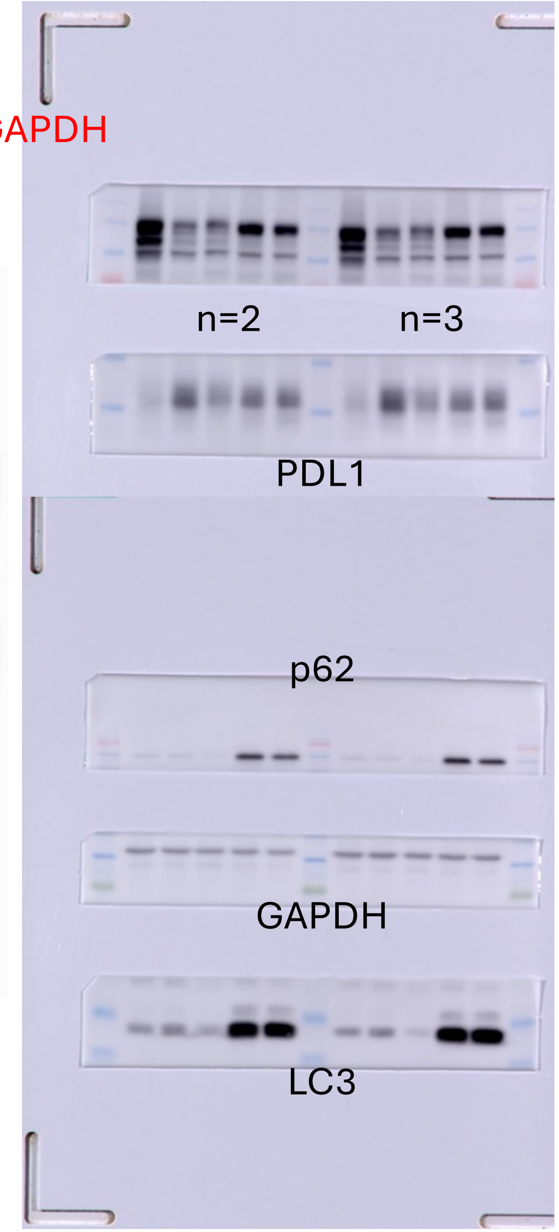

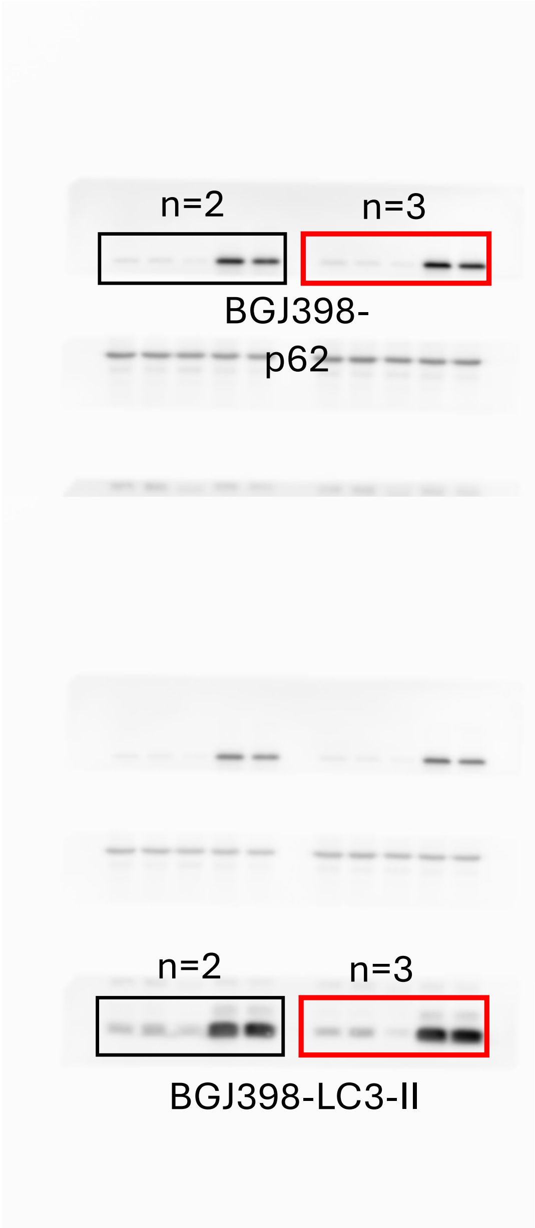

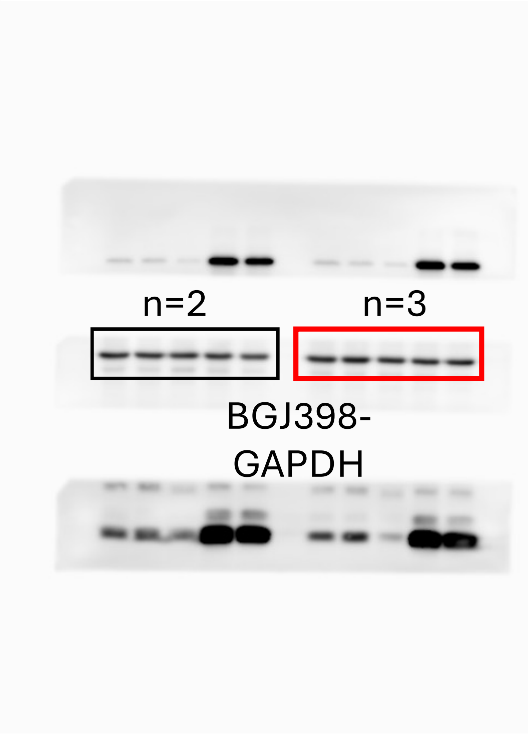

F3B RT4 (L145-CQ)(BGJ398-CQ)

2023.8.5 n=1

L145-PD-L1, p62, LC3B, GAPDH

BGJ398-PD-L1, p62, LC3B, GAPDH

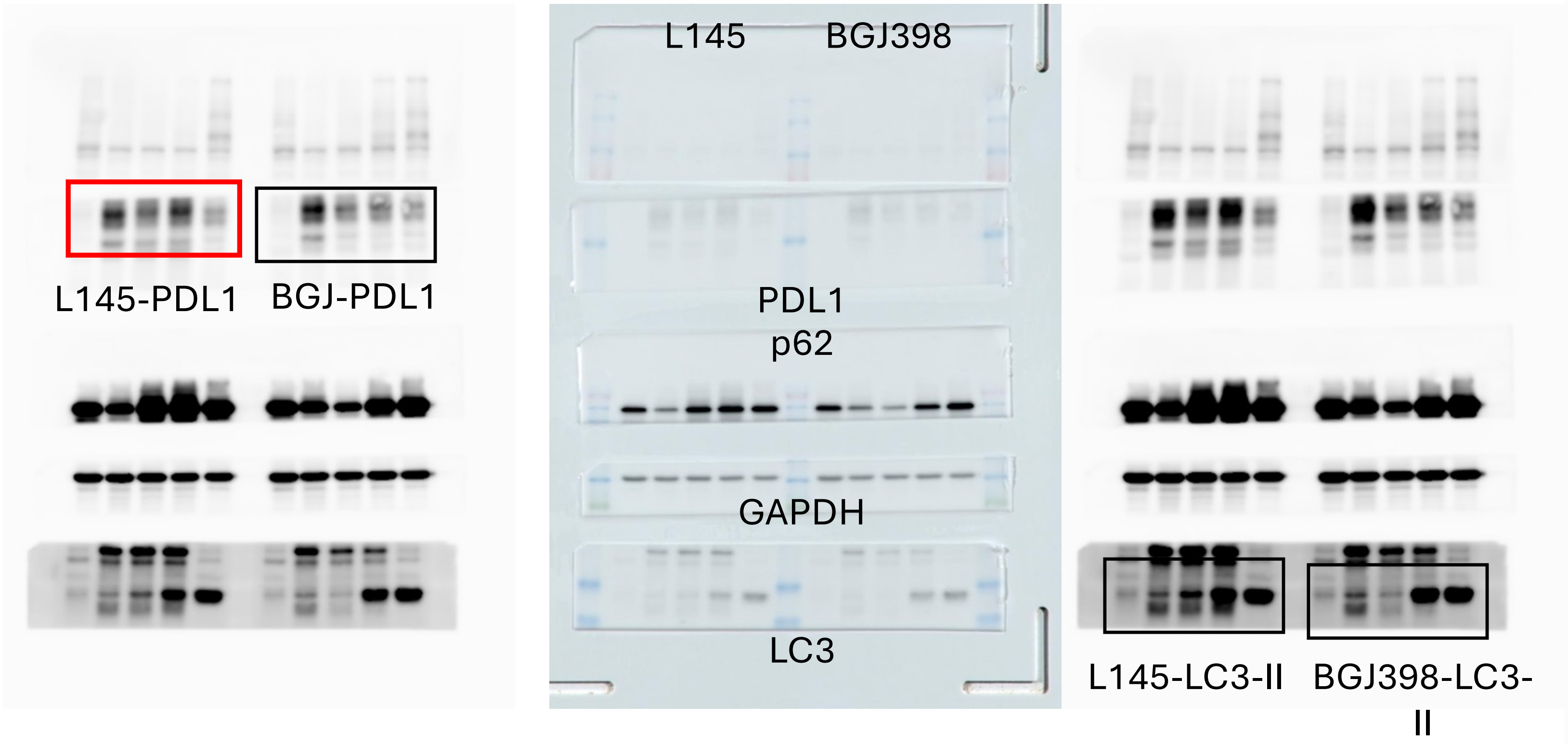

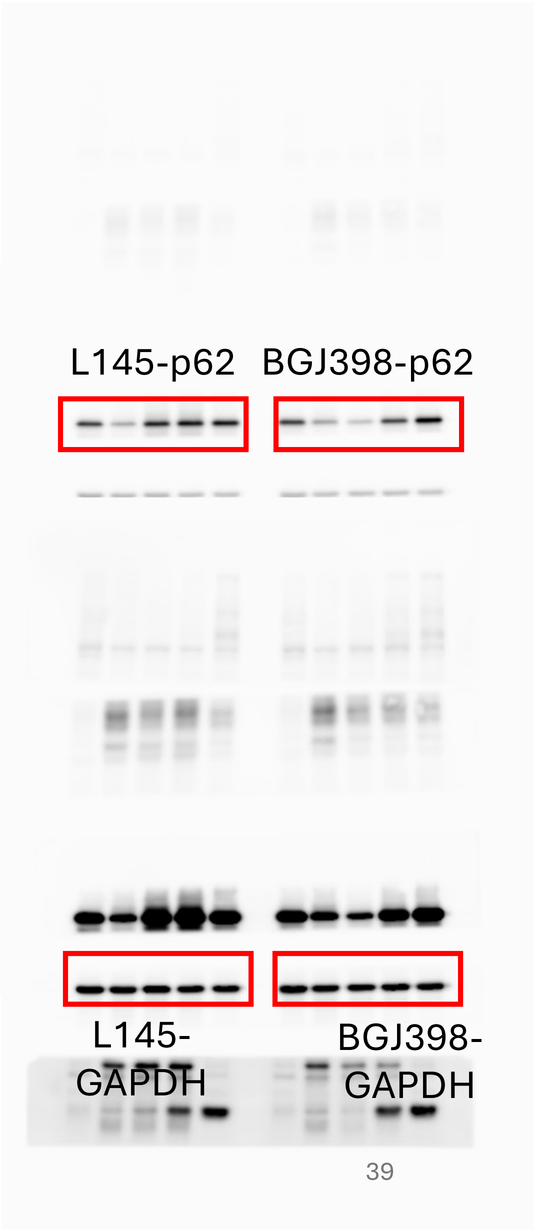

RT4 (CQ)
2023.8.12 n=2
L145-PD-L1, p62, LC3B, GAPDH
BGJ398-PD-L1, p62, LC3B, GAPDH

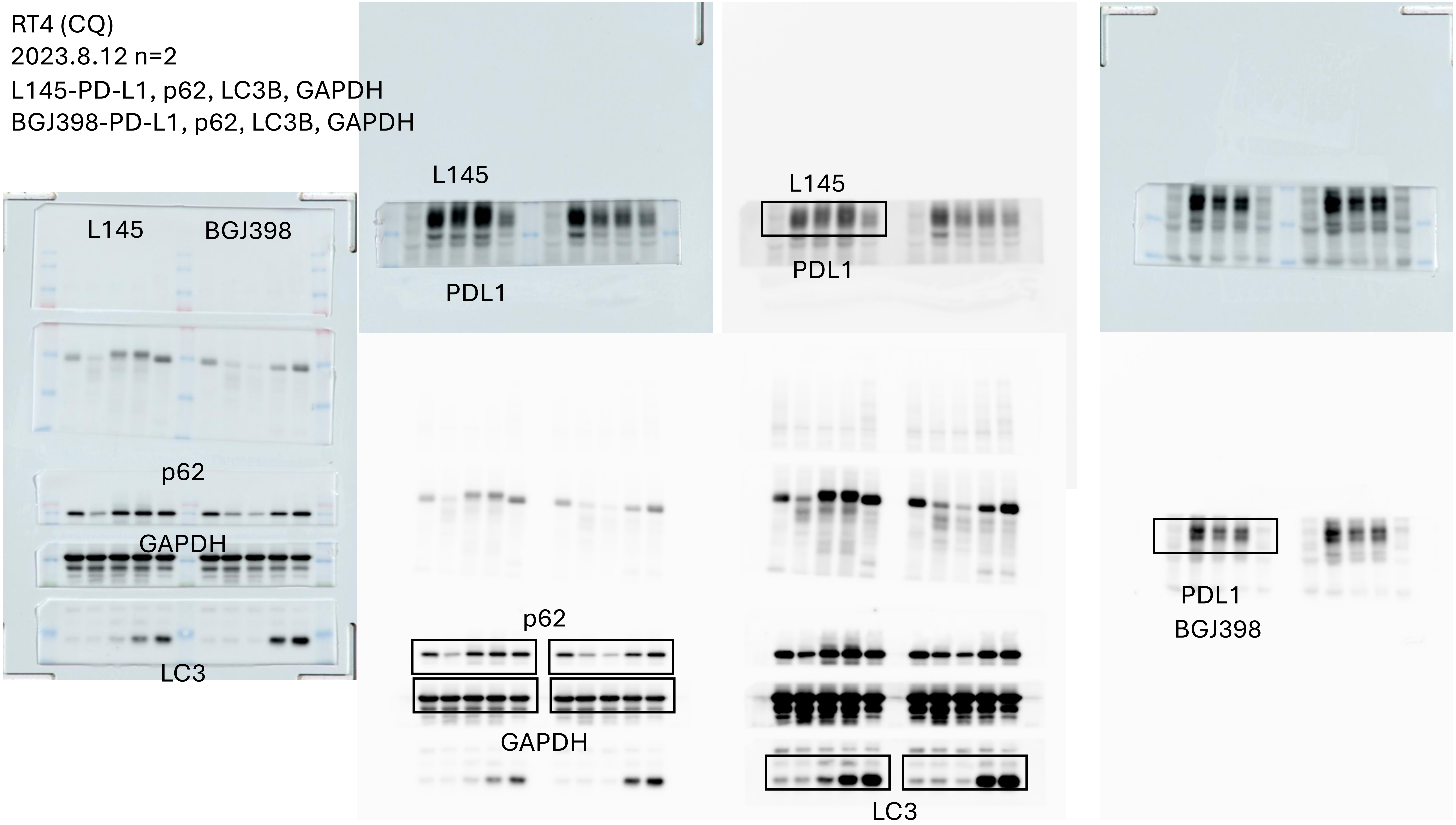

F3B RT4 (L145-CQ)(BGJ398-CQ)

2023.8.12 n=3

L145-PD-L1, p62, LC3B, GAPDH

BGJ398-PD-L1, p62, LC3B, GAPDH

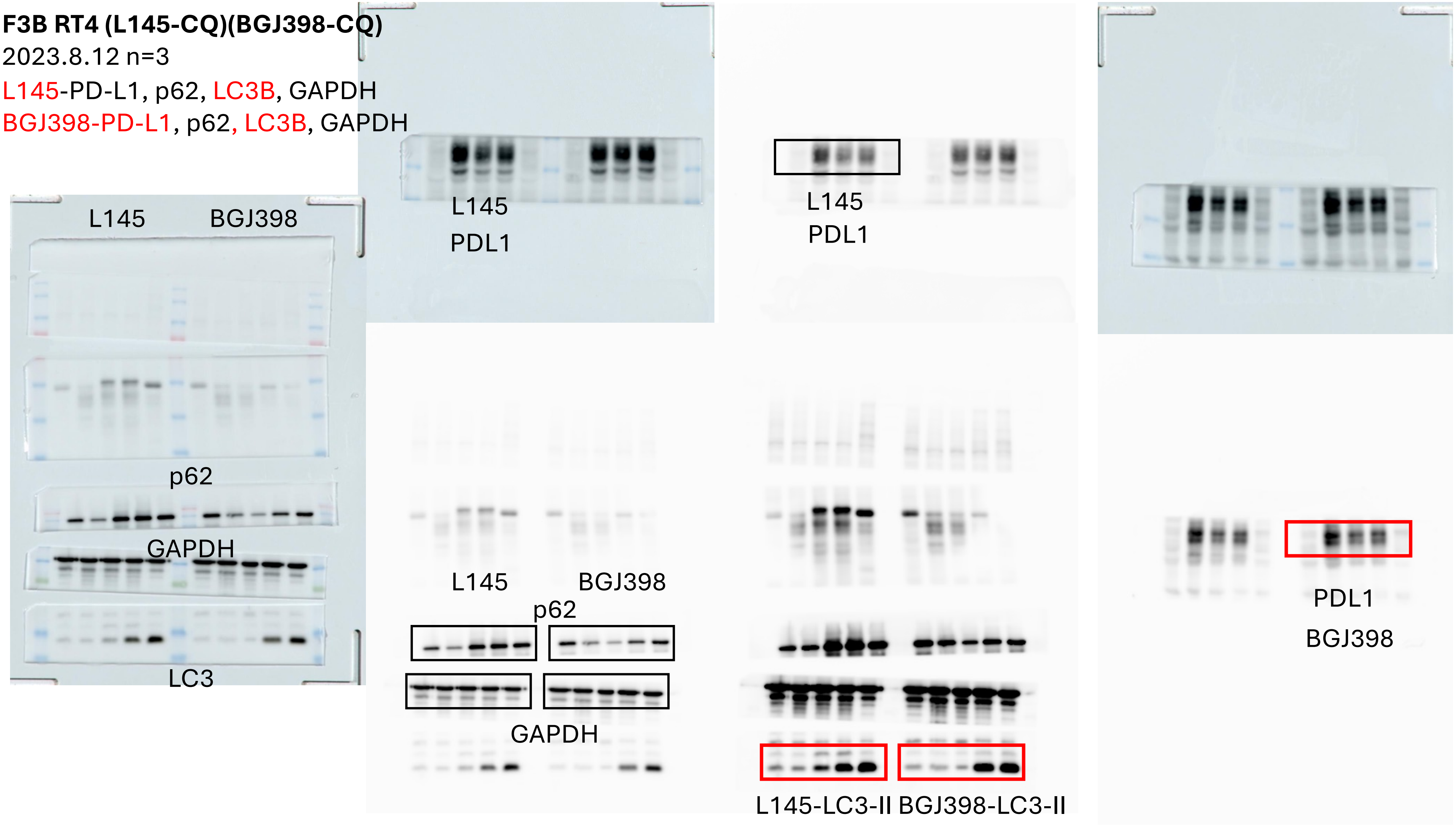

F3C RT-112 (ATG5 KO)

2023.8.5 n=1

ATG5, GAPDH

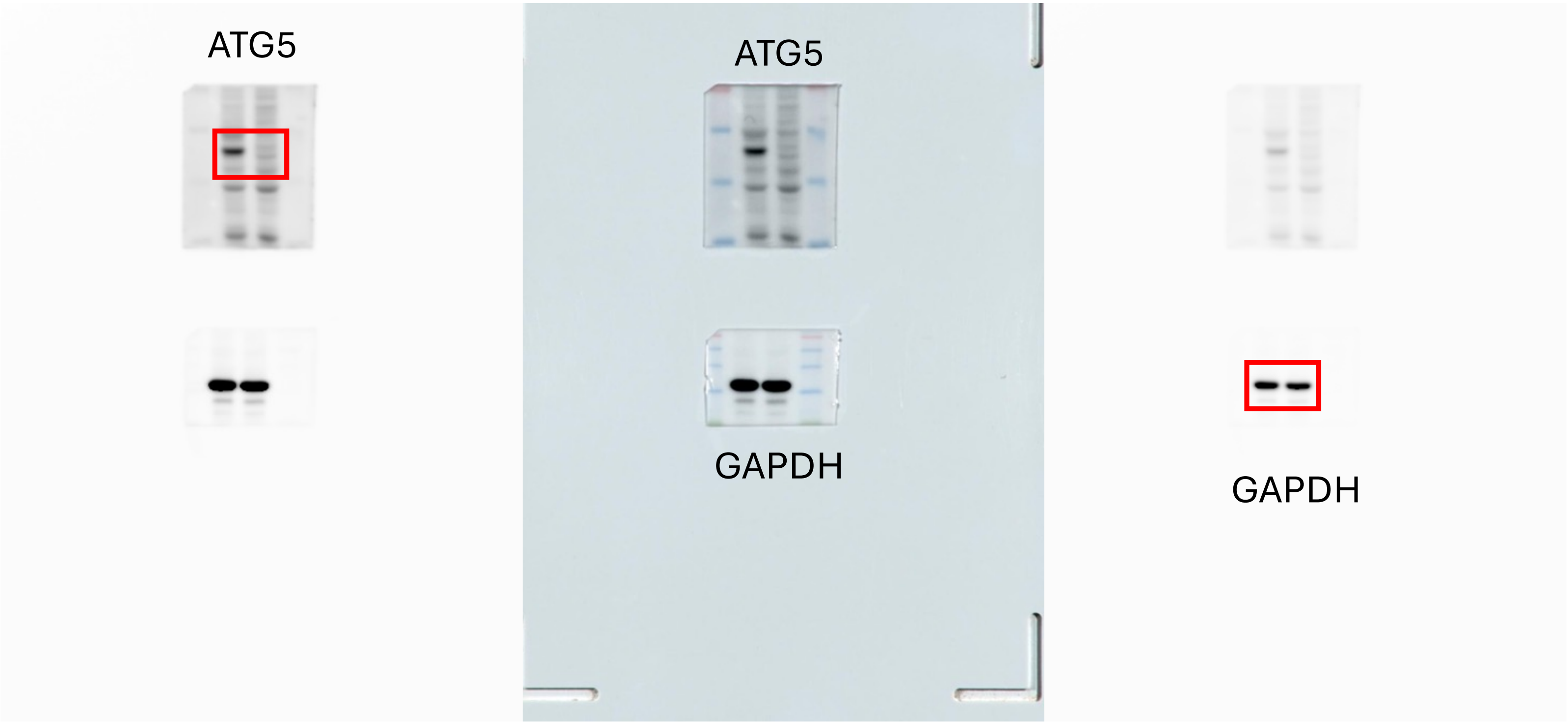

RT112
2023.8.12 n=2, 3
ATG5, GAPDH

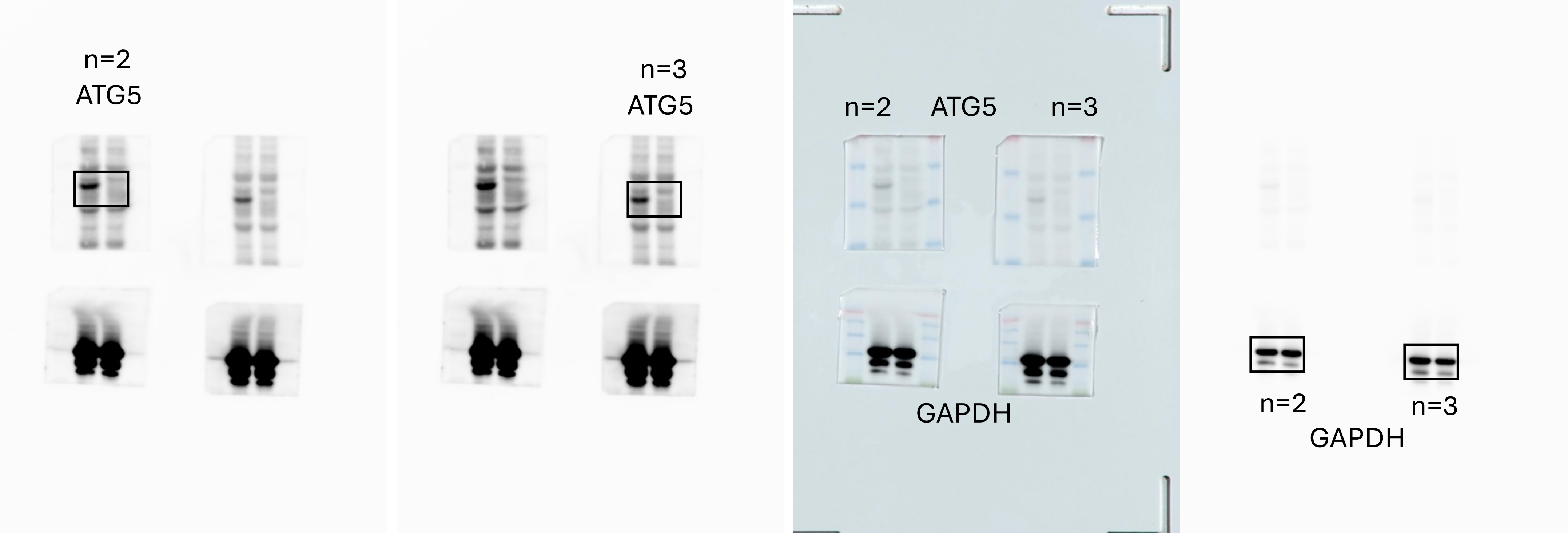

F3C RT-112 (ATG5 KO)

2023.12.22 n=1, 2, 3

p62, LC3B

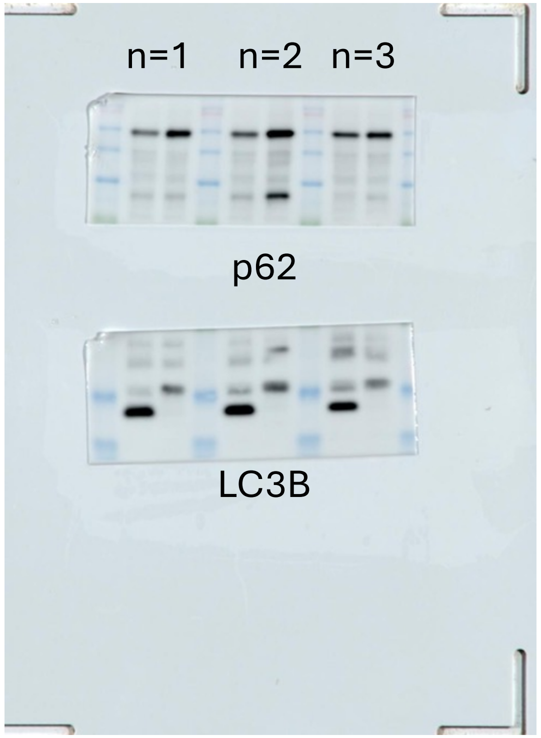

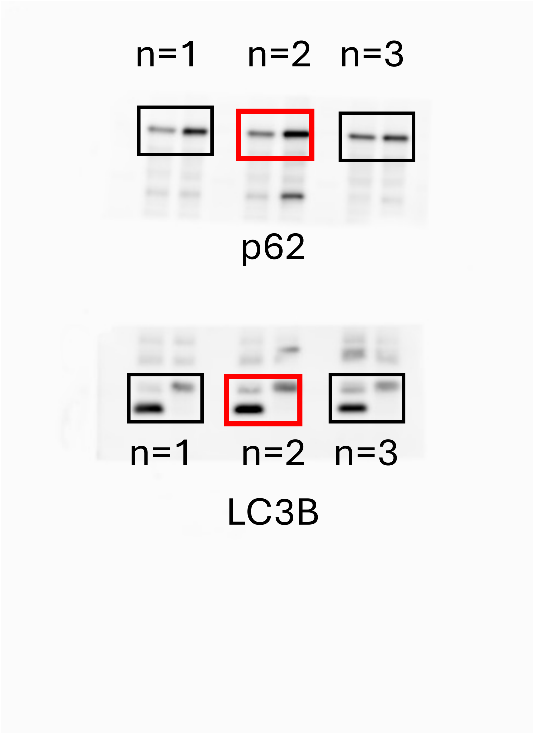

RT112 2019.1.4 n=1 L145-PD-L1, GAPDH
RT112-ATG5KO 2019.1.4 n=1 L145-PD-L1, GAPDH

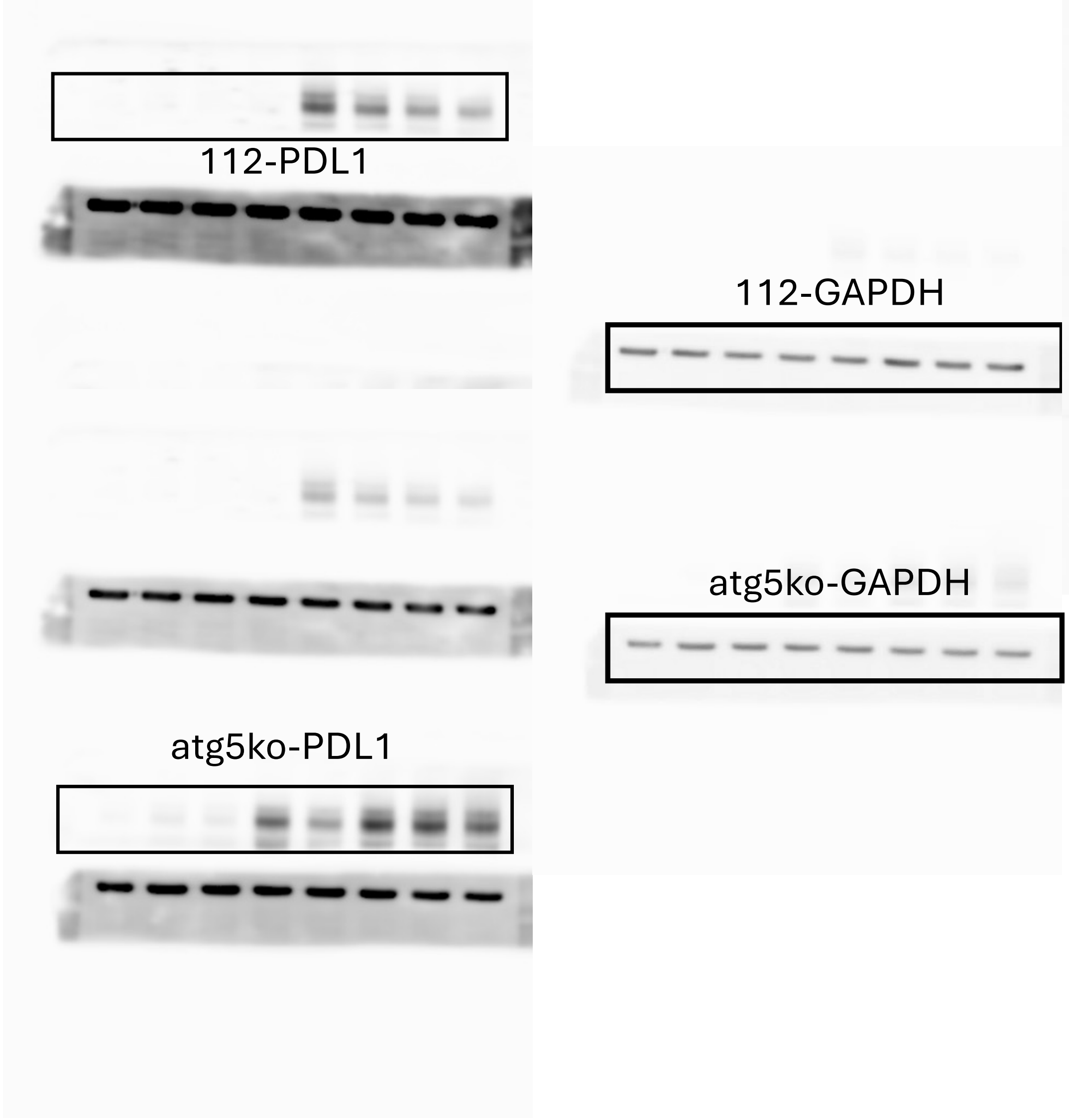

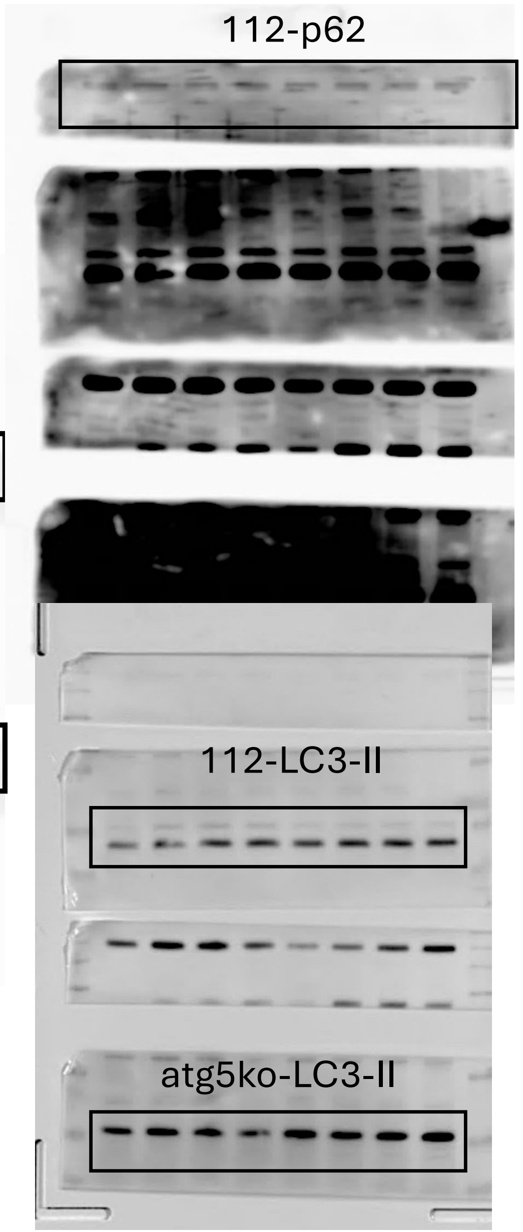

RT112 2019.1.10 n=1
L145-p62, LC3B
RT112-ATG5KO 2019.1.10 n=1
L145-p62, LC3B

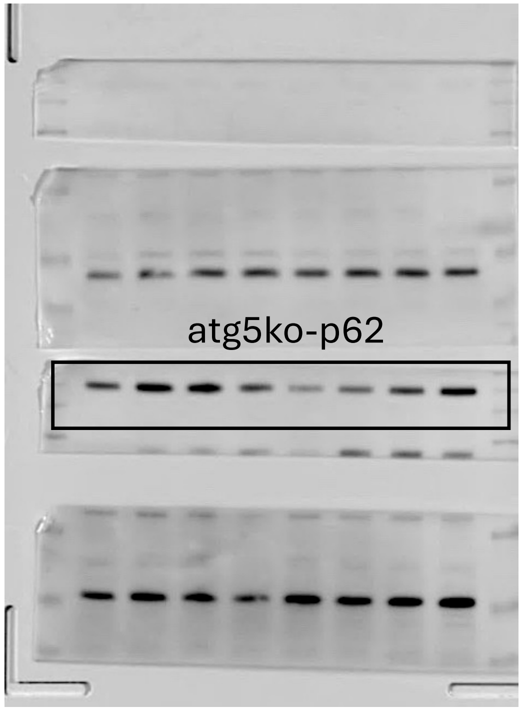

RT112, RT112-ATG5KO 2019.01.04 n=2
L145-PD-L1, GAPDH

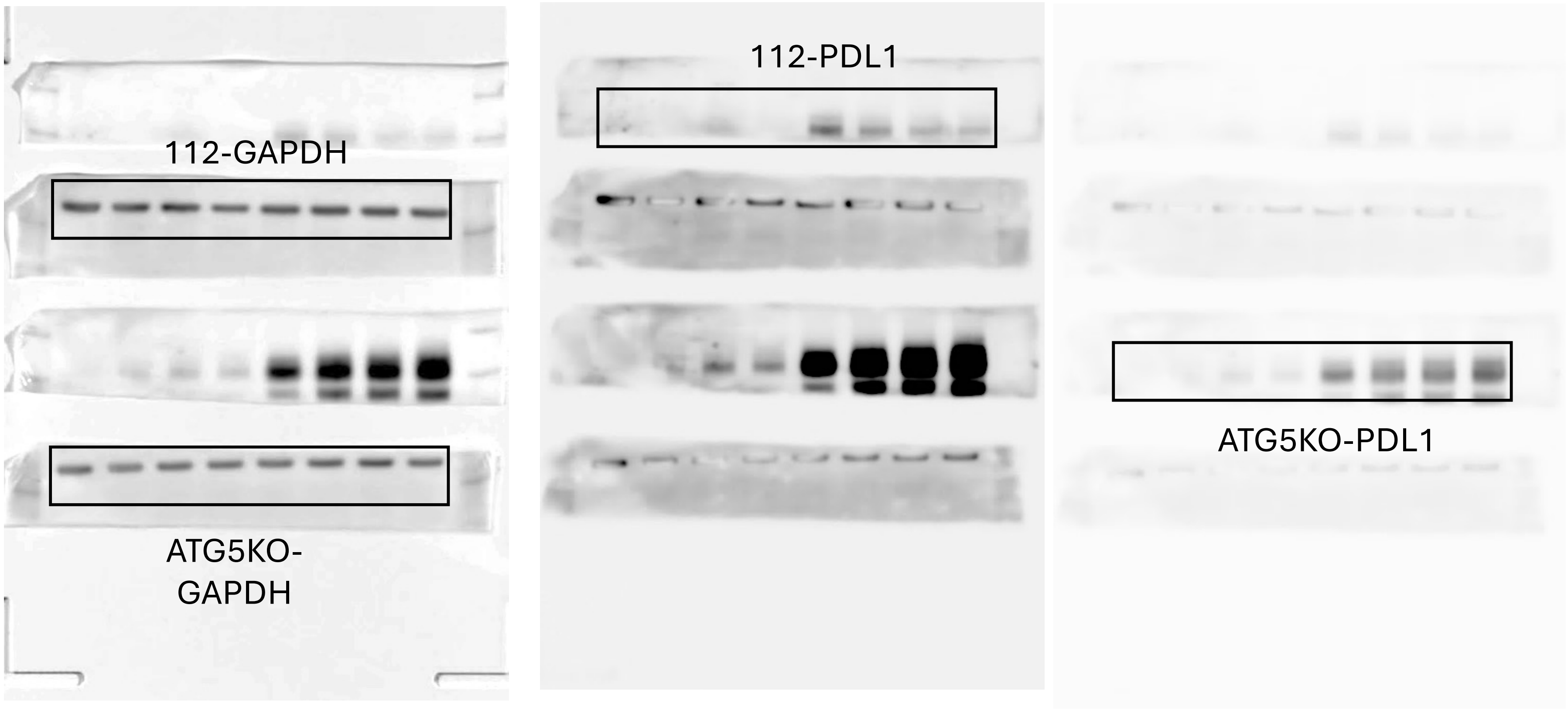

F3D RT-112, ATG5KO (L145)

2019.1.18 L145 n=3

RT-112-PD-L1, p62, LC3, GAPDH

ATG5KO-PD-L1, p62, LC3, GAPDH

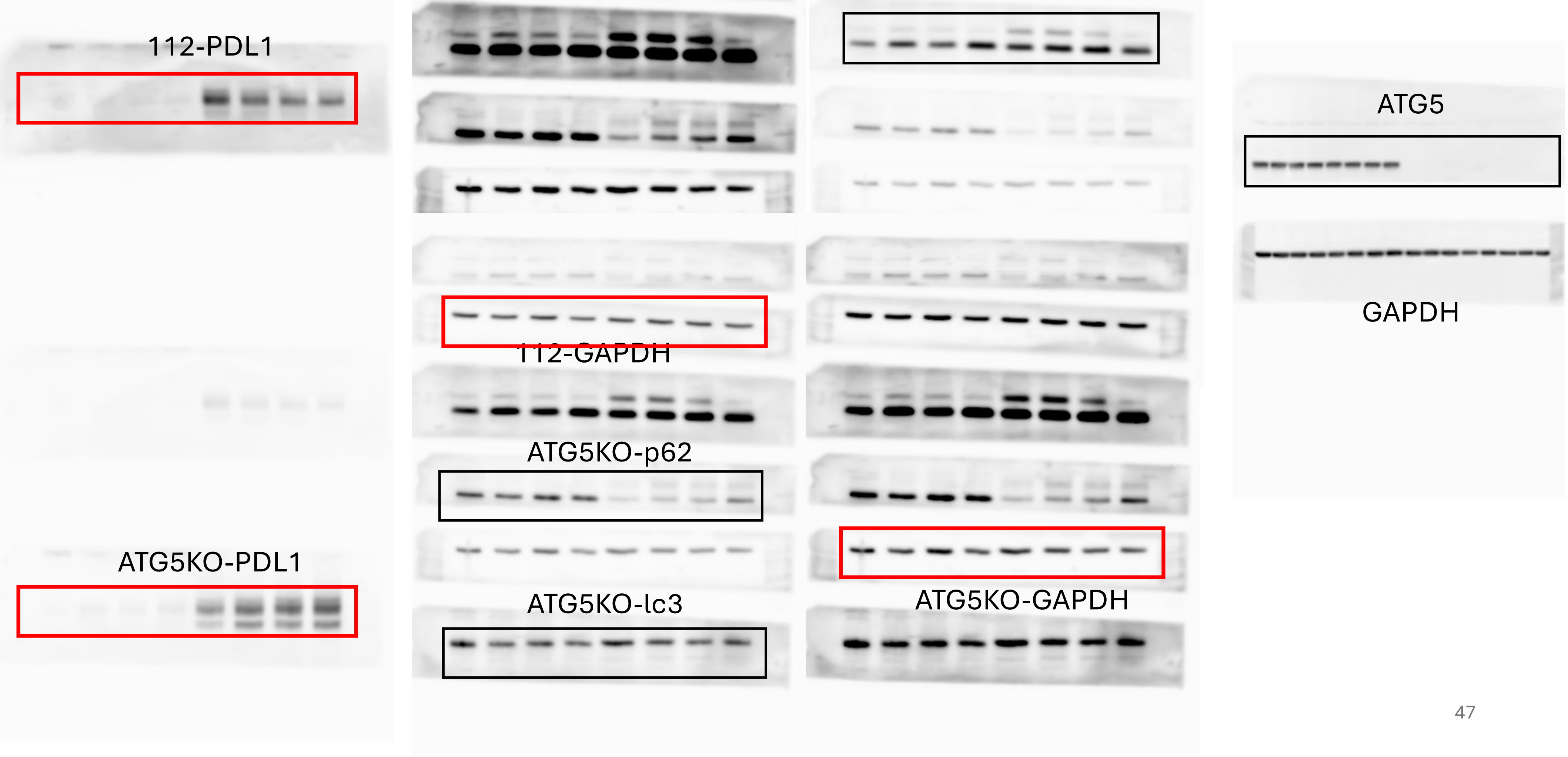

F3D RT-112, ATG5KO (L145)

2019.01.25 L145 n=3

RT-112-p62, LC3, ATG5, GAPDH

ATG5KO-p62, LC3, ATG5, GAPDH

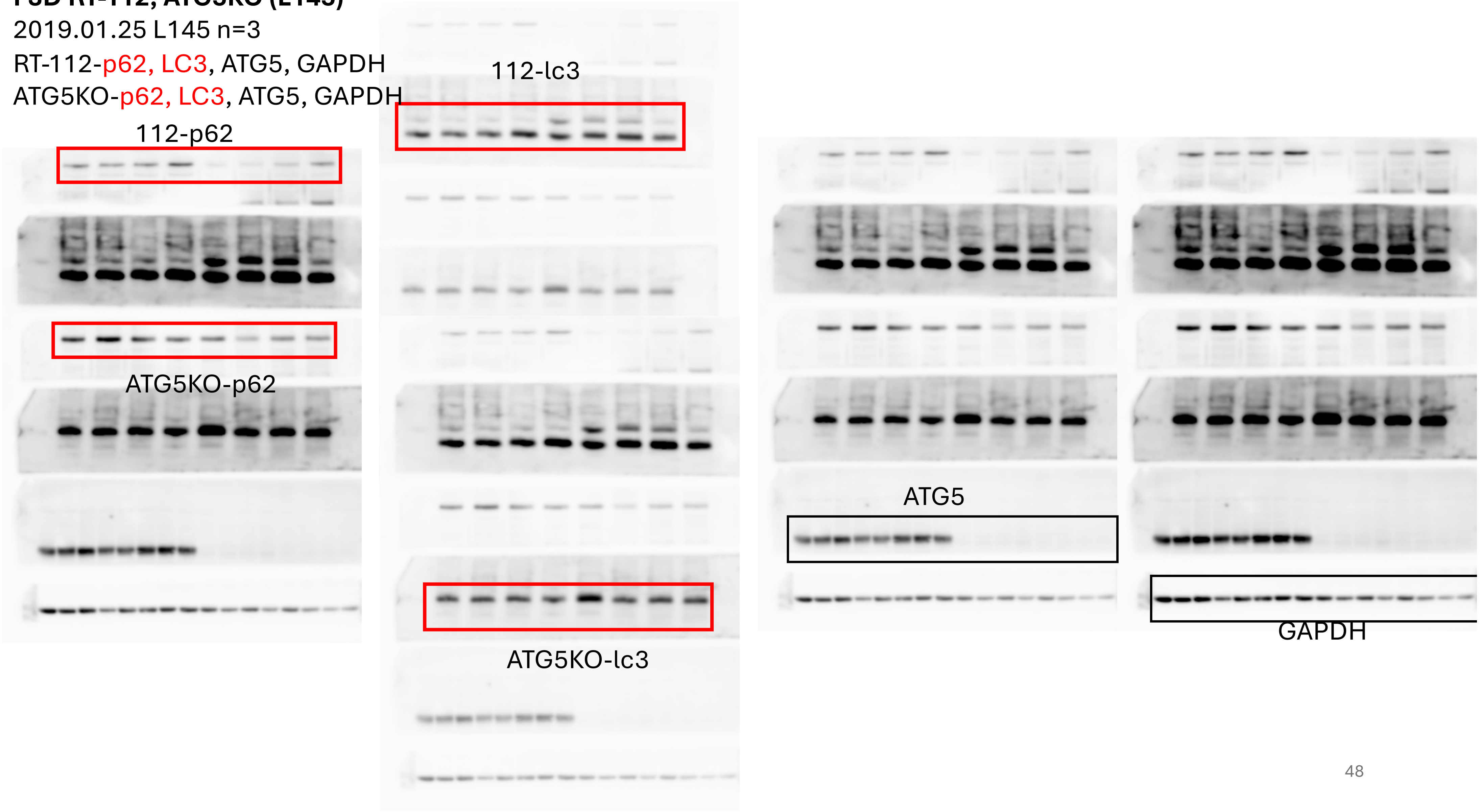

F3D RT-112, ATG5KO (BGJ398)

2019.02.14 BGJ398 n=1

RT-112-PD-L1, p62, LC3, GAPDH

ATG5KO-PD-L1, p62, LC3, GAPDH

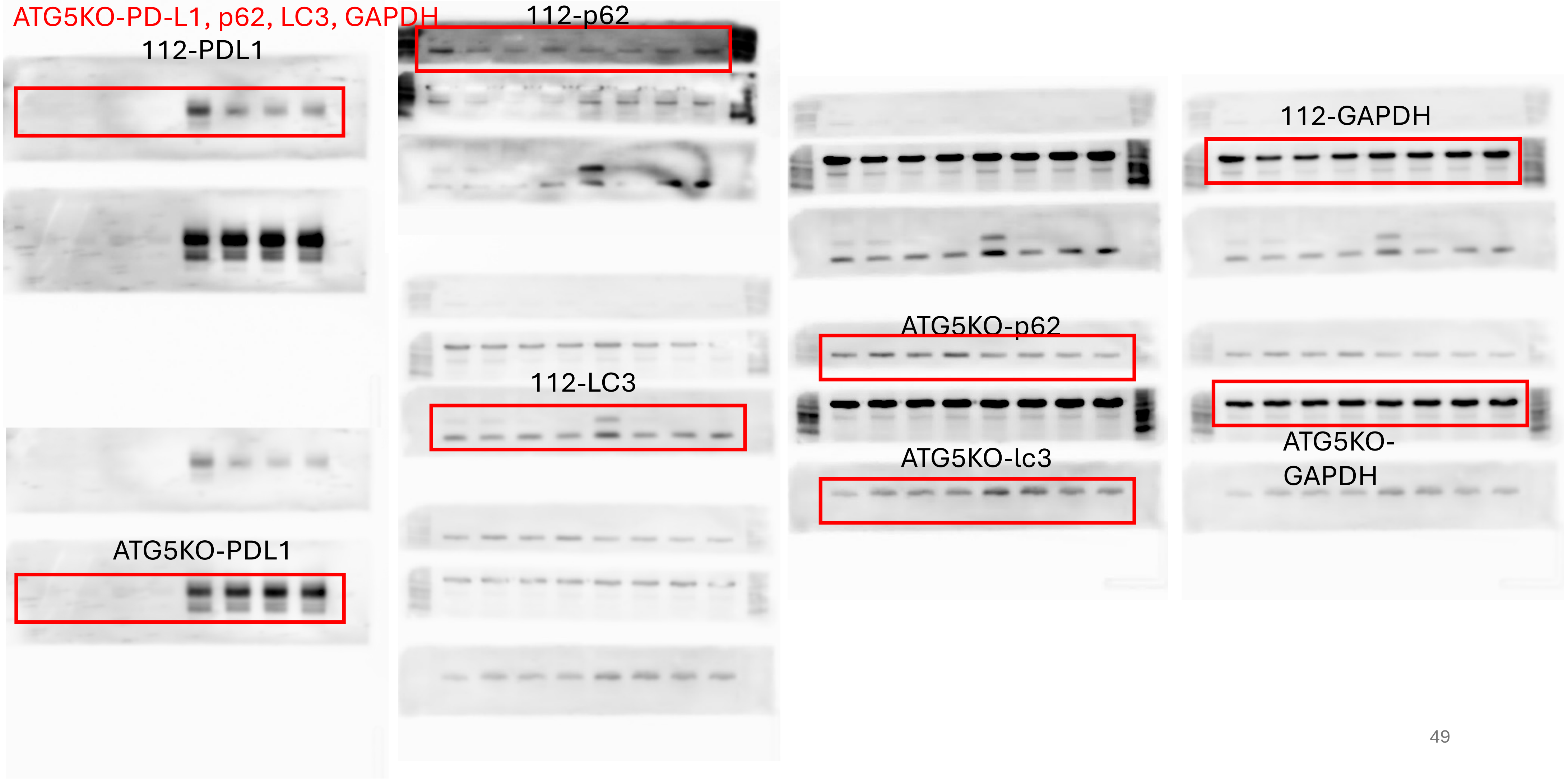

RT112
2023.8.5 n=2
BGJ398-PD-L1, p62, LC3B, GAPDH

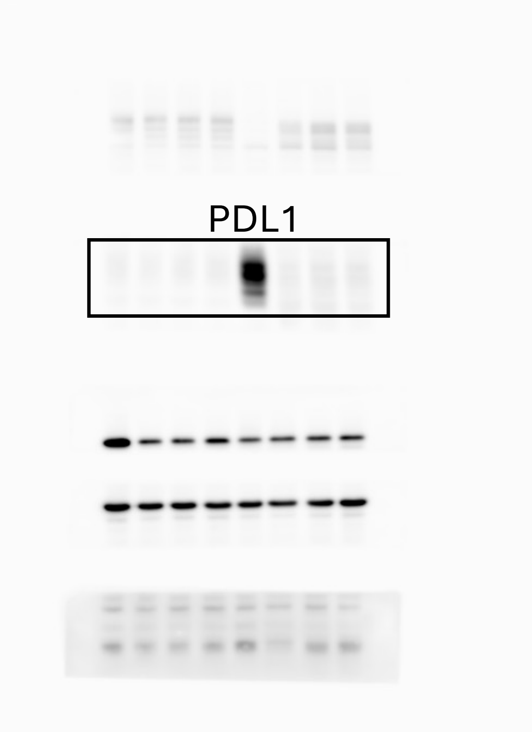

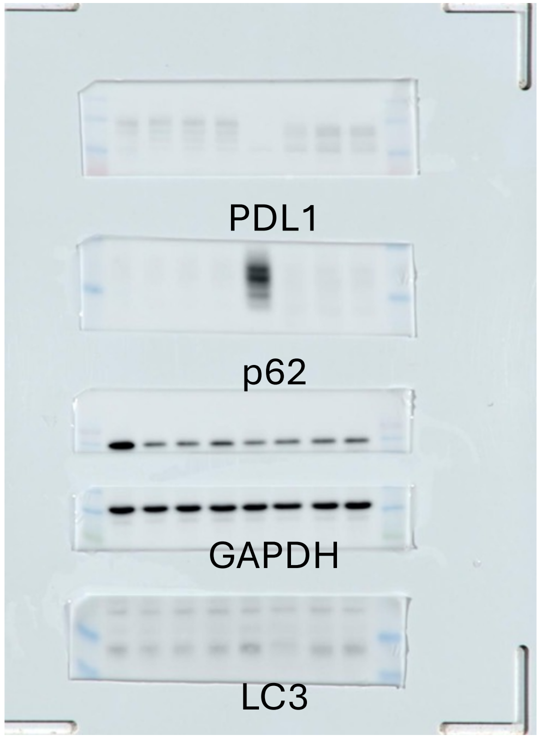

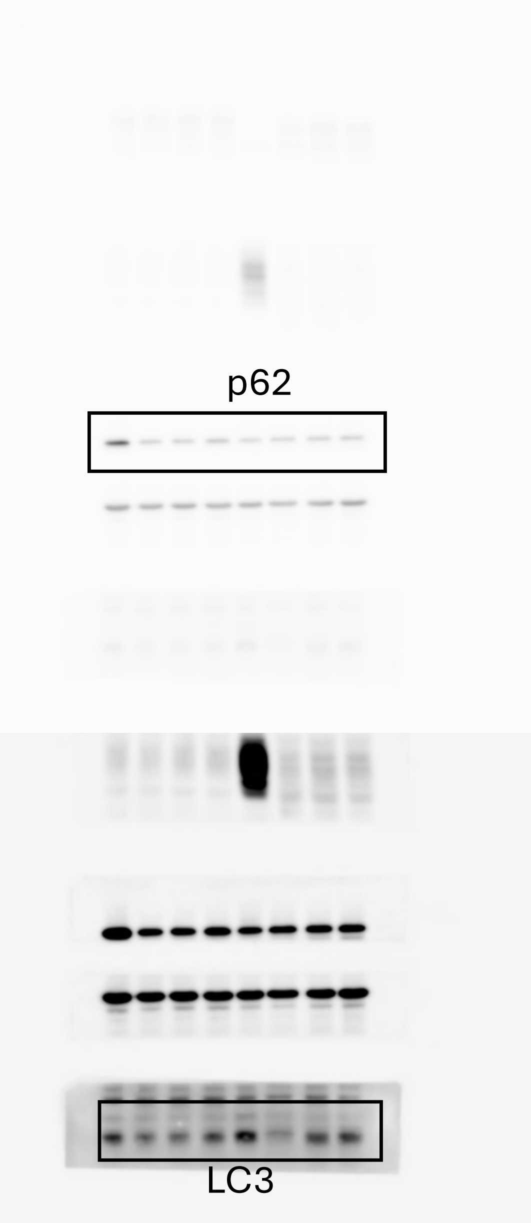

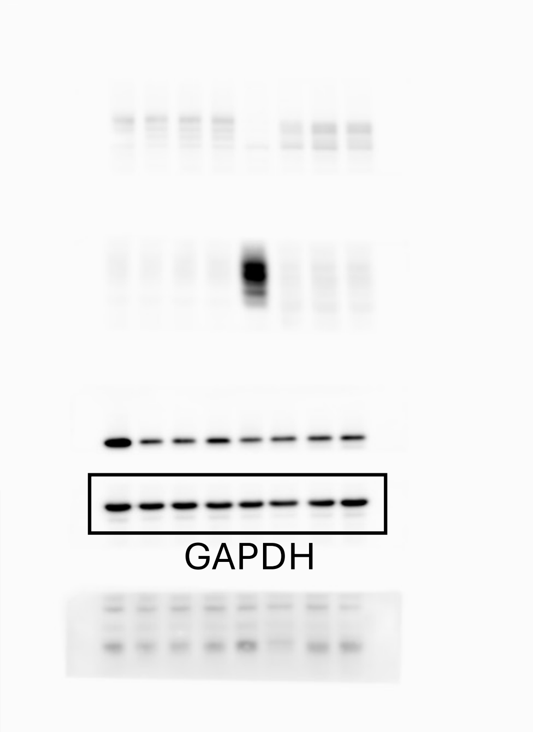

RT112-ATG5KO
2023.8.5 n=2
BGJ398-PD-L1, p62, LC3B, GAPDH

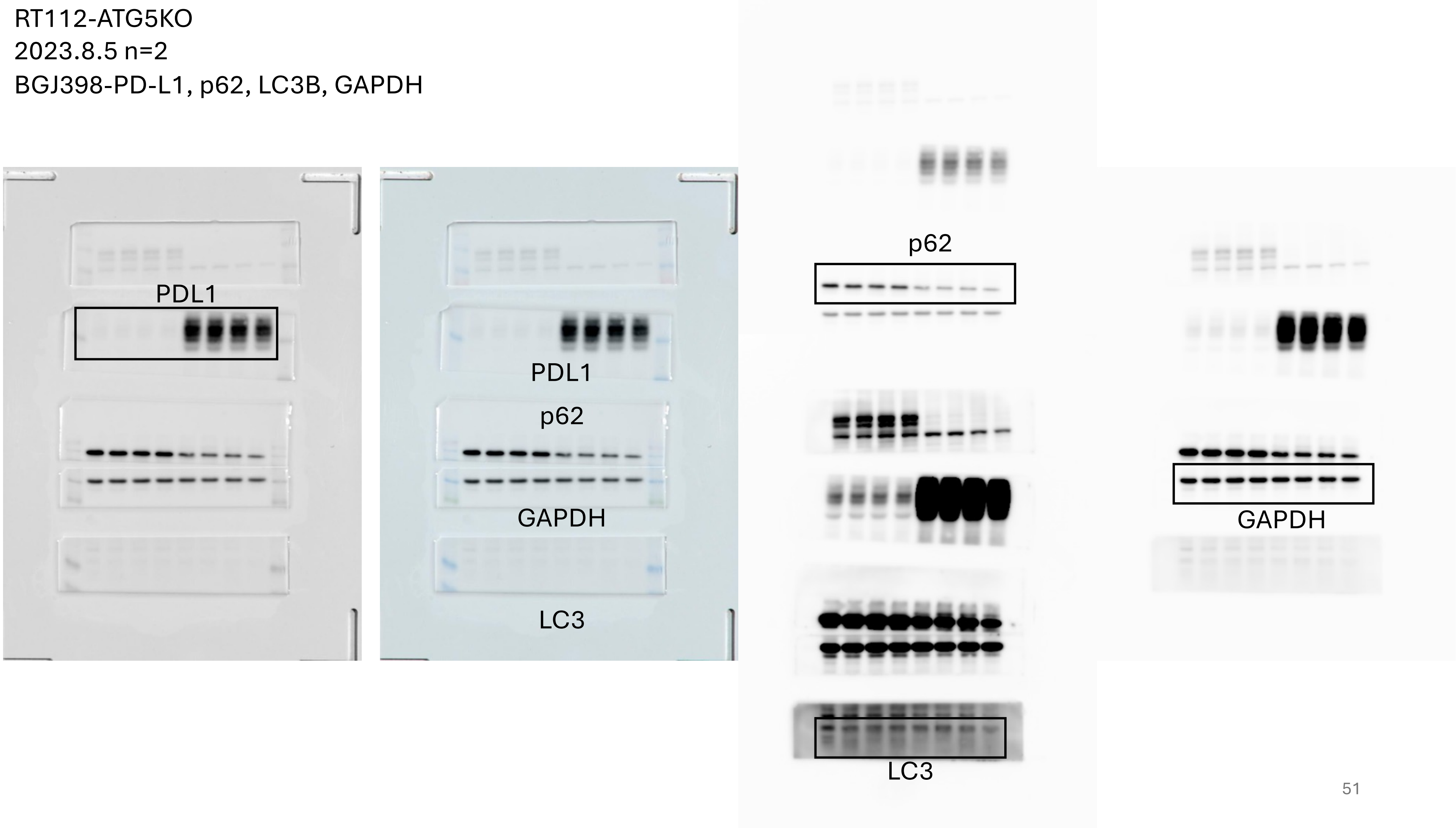

RT112
2023.8.12 n=3
BGJ398-PD-L1, p62, LC3B, GAPDH

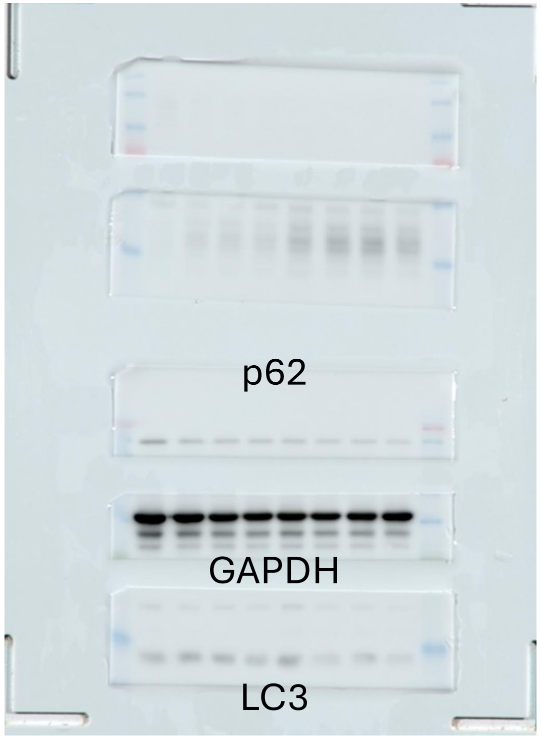

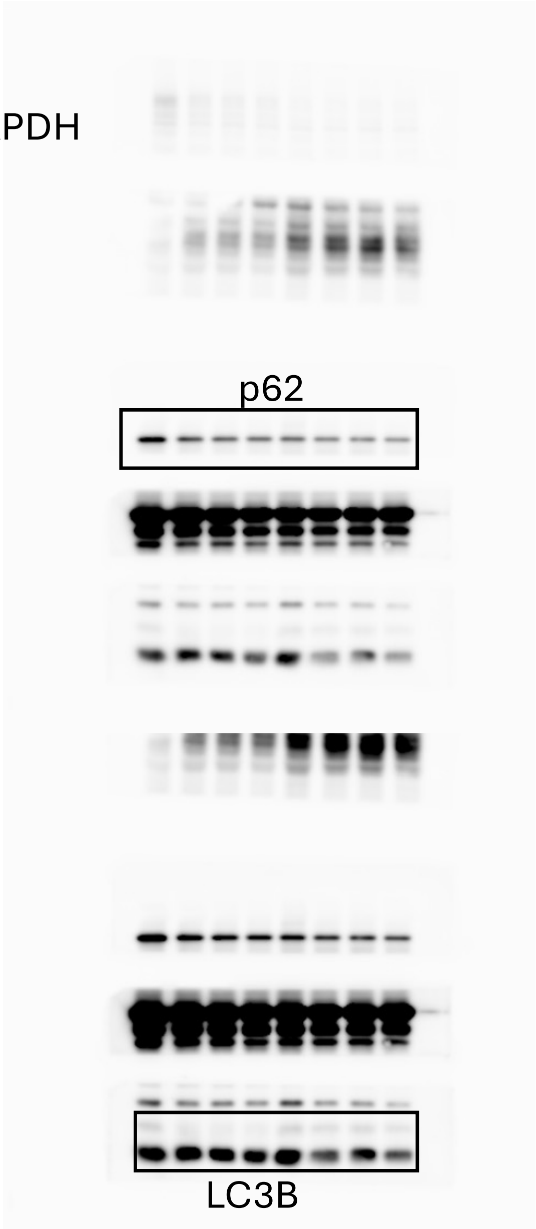

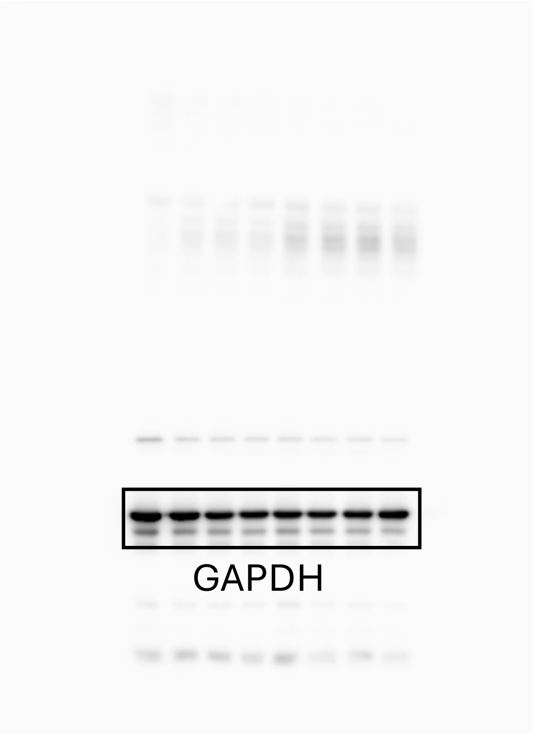

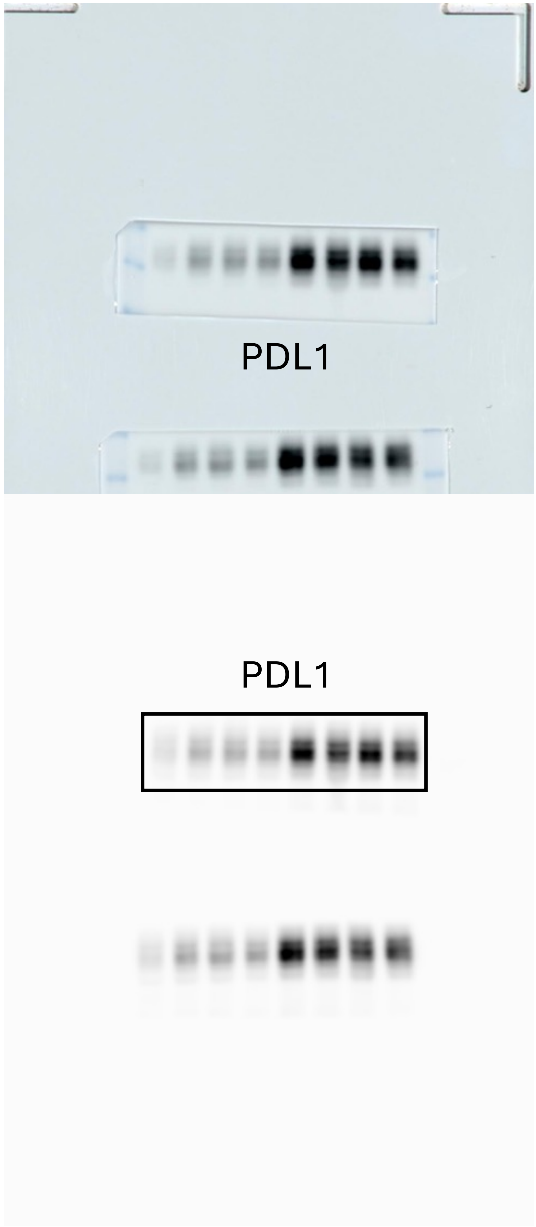

RT112
2023.8.12 n=4
BGJ398-PD-L1, p62, LC3B, GAPDH

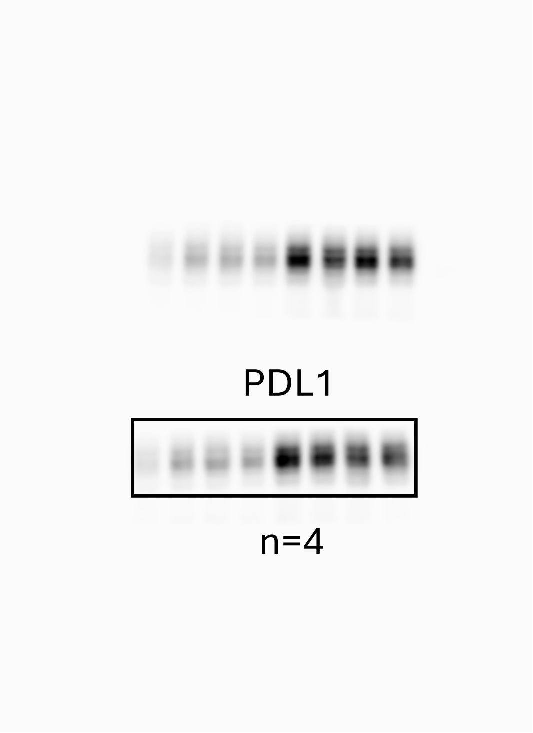

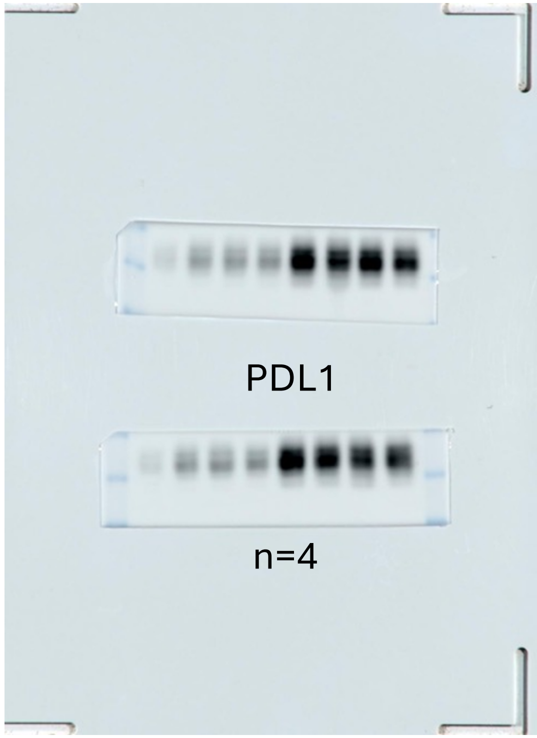

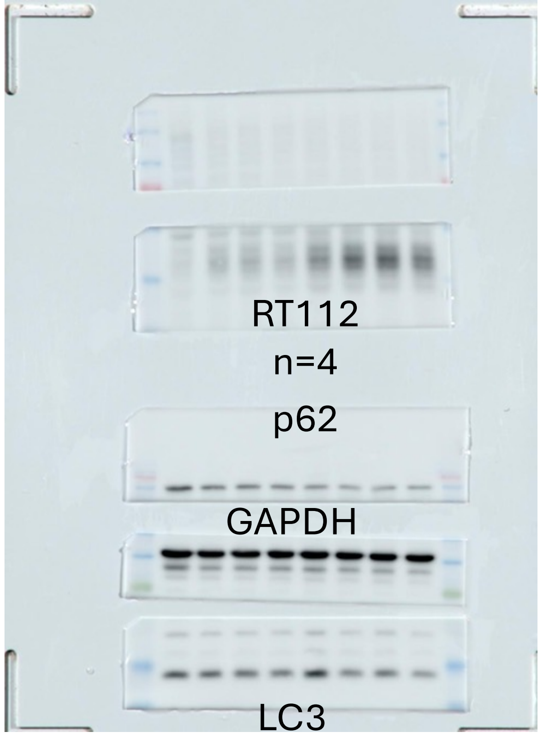

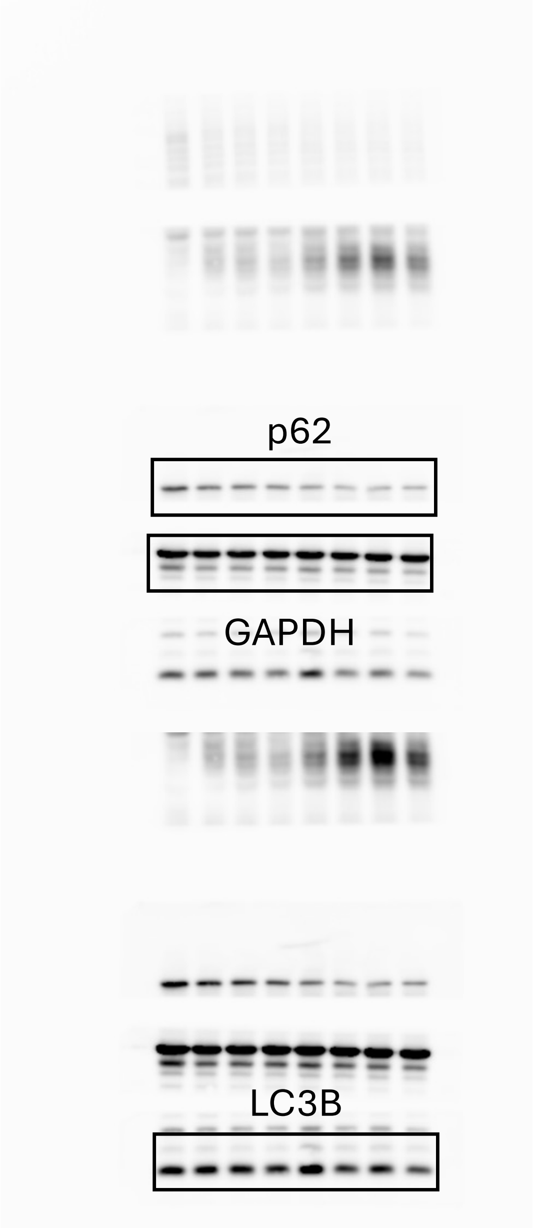

RT112-ATG5KO
2023.8.12 n=3
BGJ398-PD-L1, p62, LC3B, GAPDH

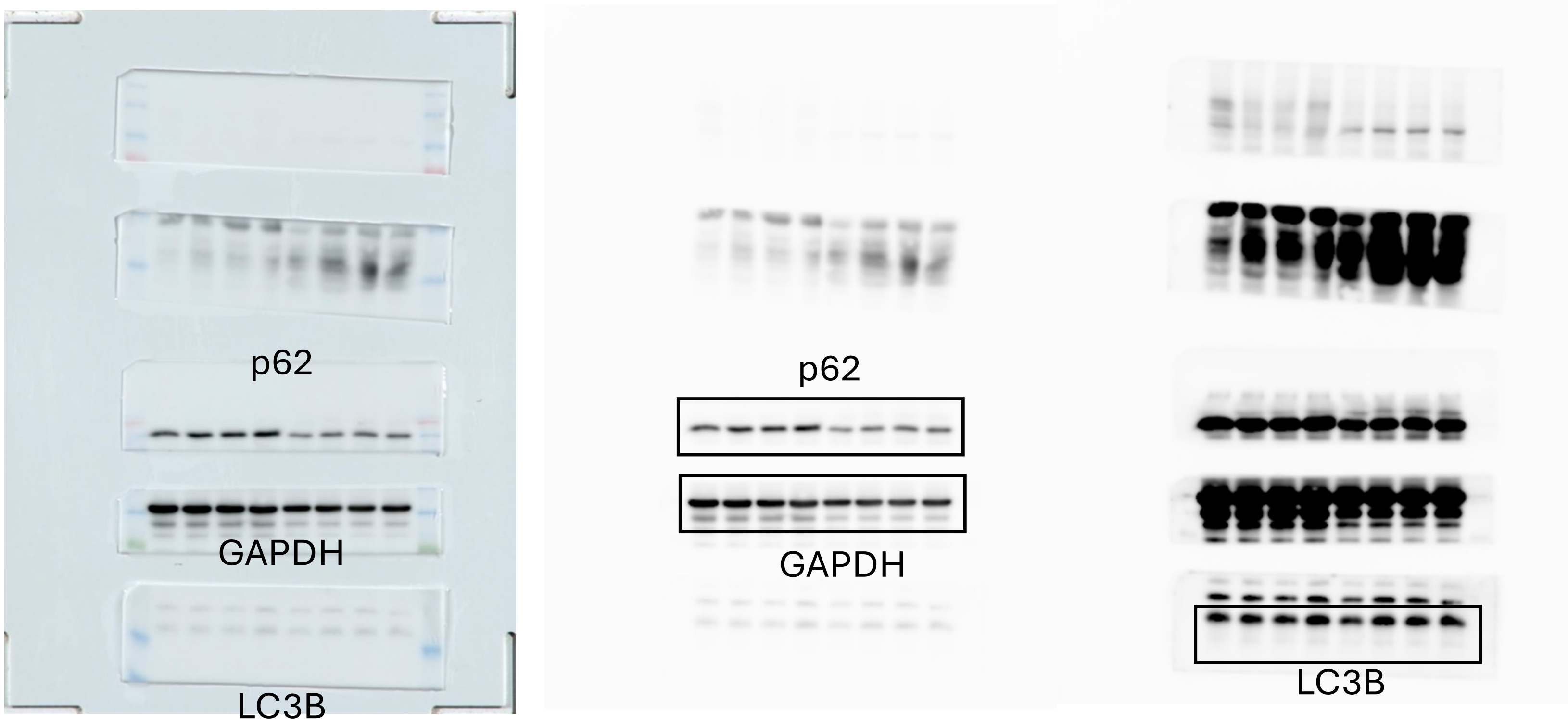

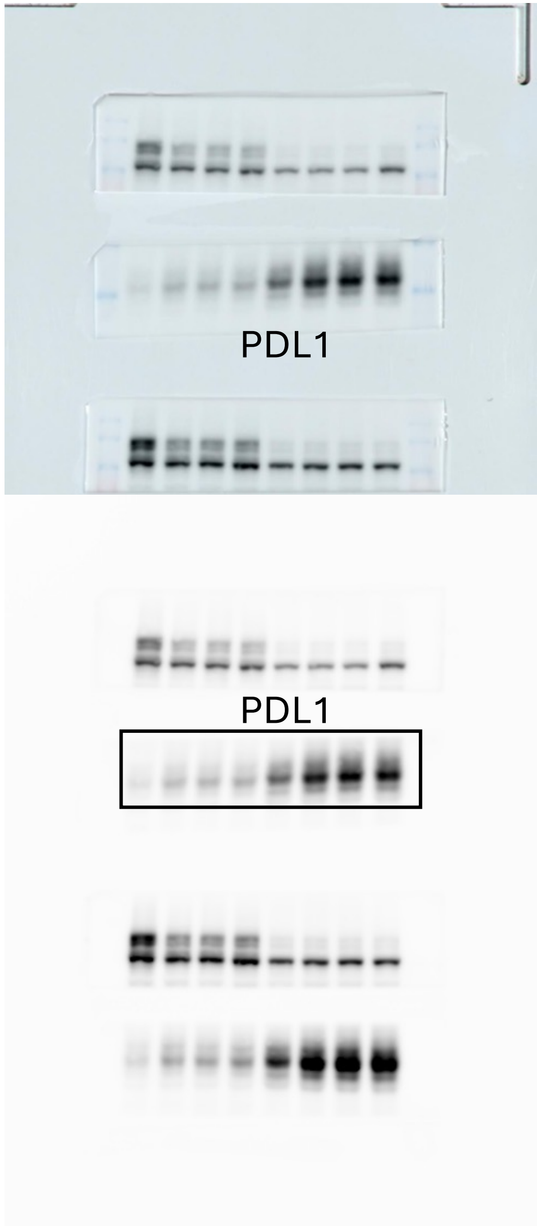

RT112-ATG5KO
2023.8.12 n=4
BGJ398-PD-L1, p62, LC3B, GAPDH

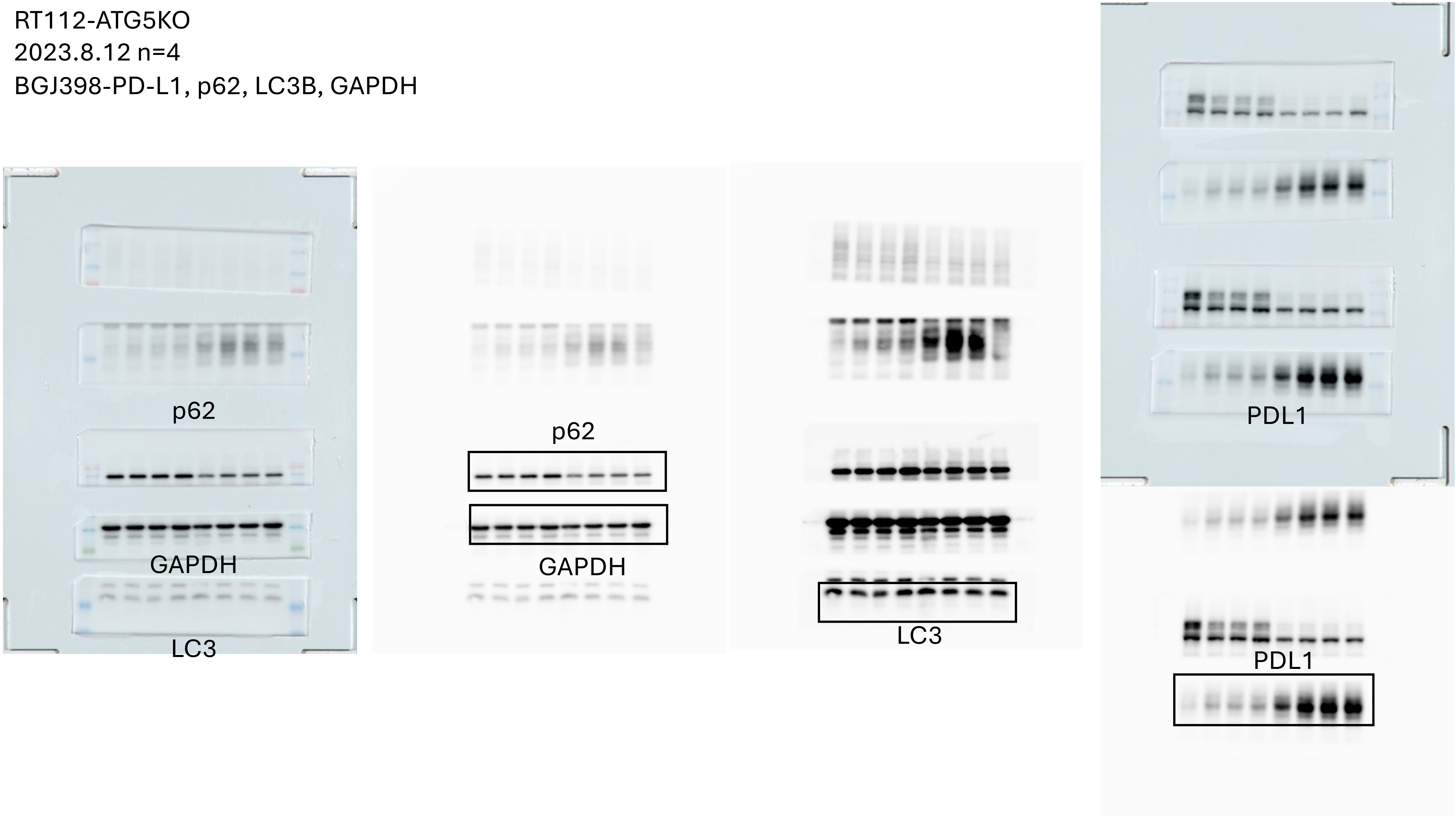

RT112-ATG5KO
2023.9.11 n=5
BGJ398-PD-L1, p62, LC3B, GAPDH

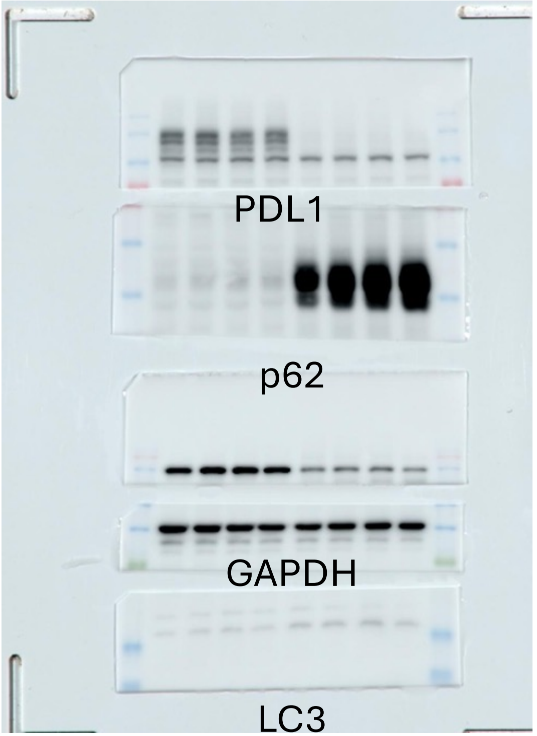

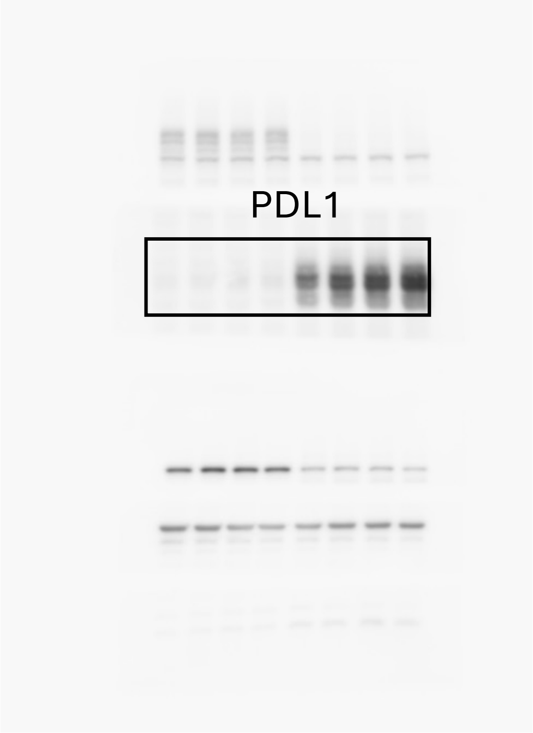

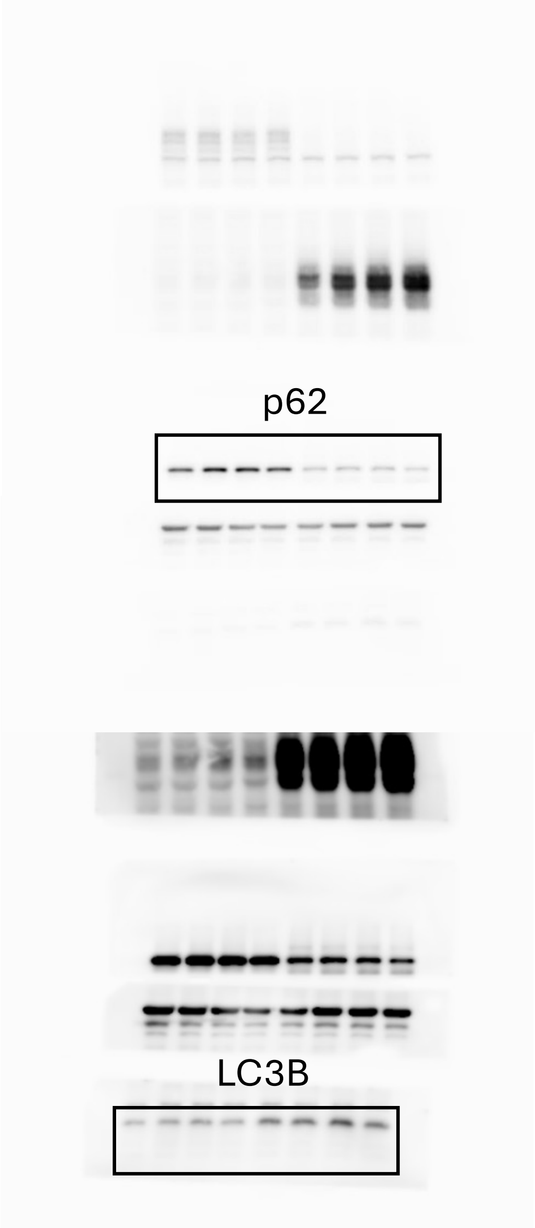

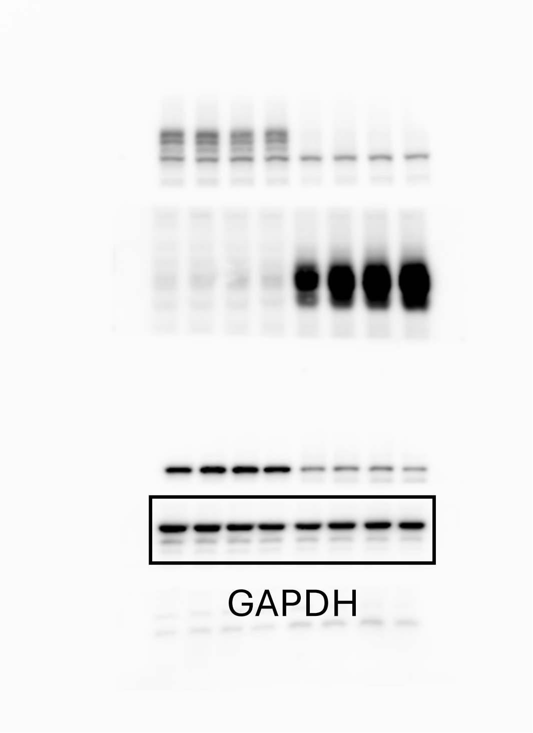

F3E RT112, SIGMAR1 OE (L145)
2022.6.21 n=1
L145-PD-L1, SIGMAR1, GAPDH

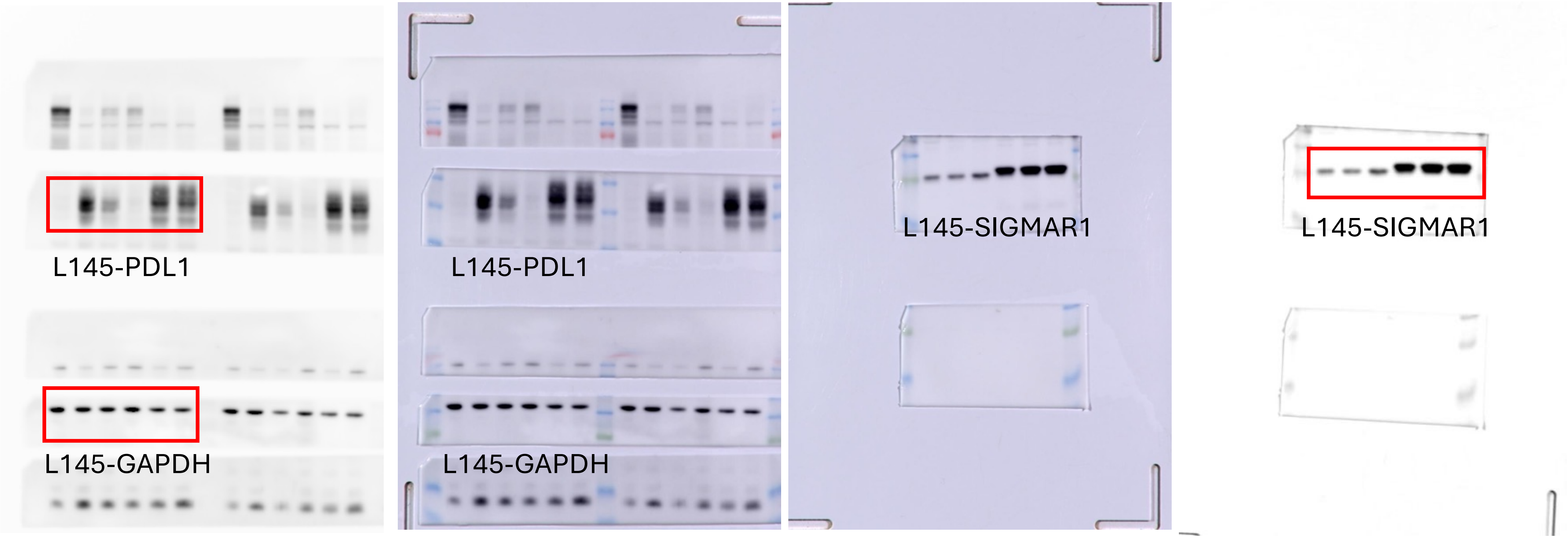

RT112, SIGMAR1 OE
2022.7.1 n=2
L145-PD-L1, SIGMAR1, GAPDH

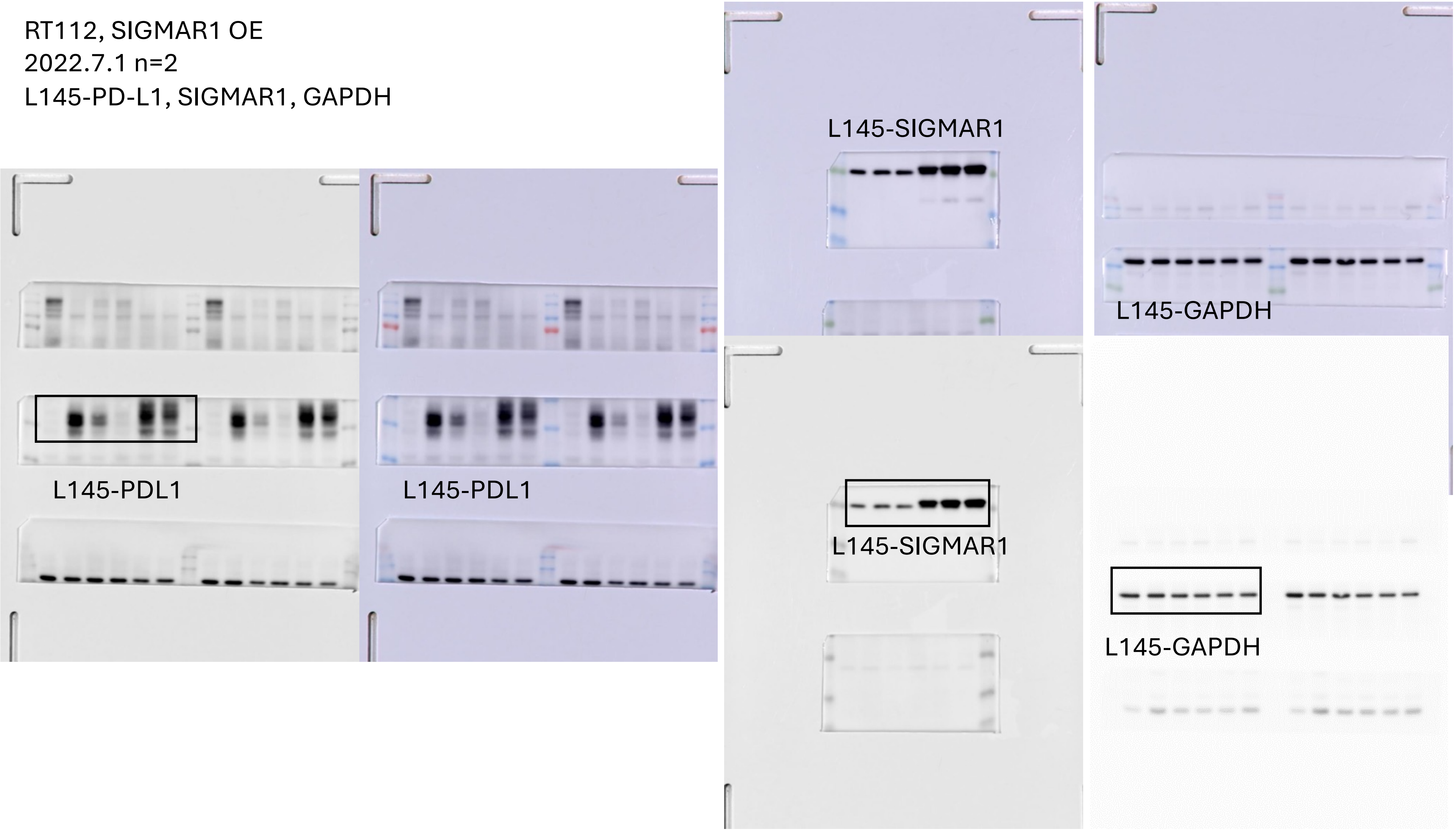

RT112, SIGMAR1 OE
2022.7.12 n=3
L145-PD-L1, SIGMAR1, GAPDH

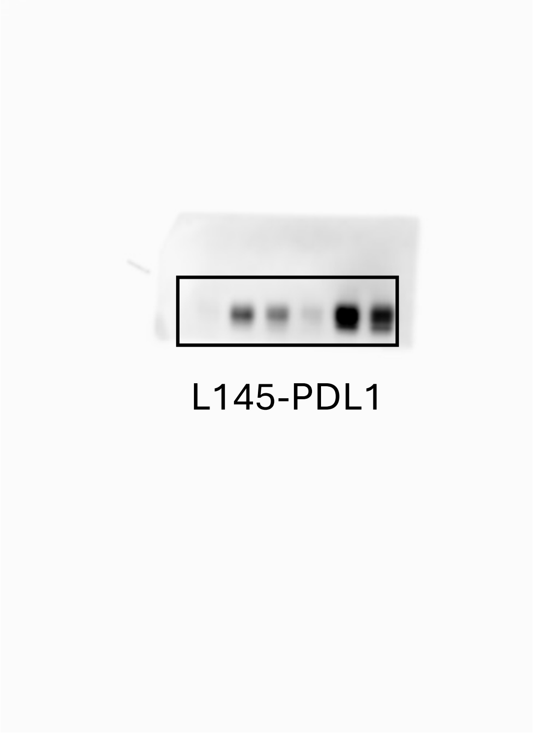

L145-PDL1

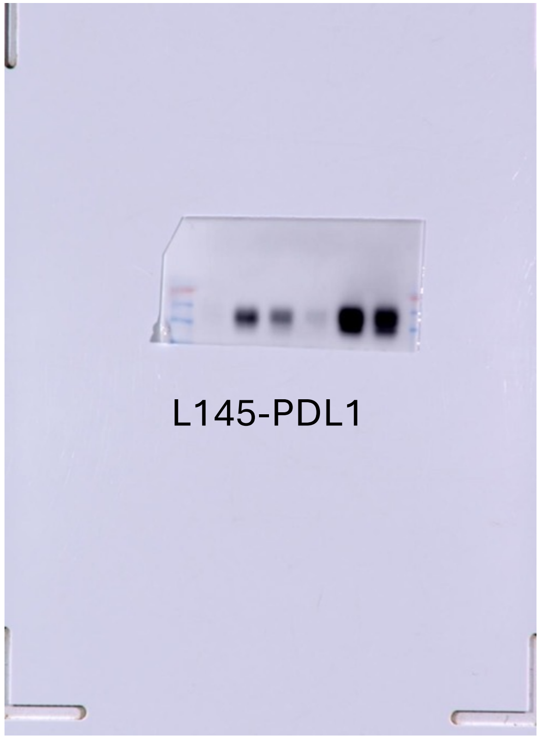

L145-PDL1

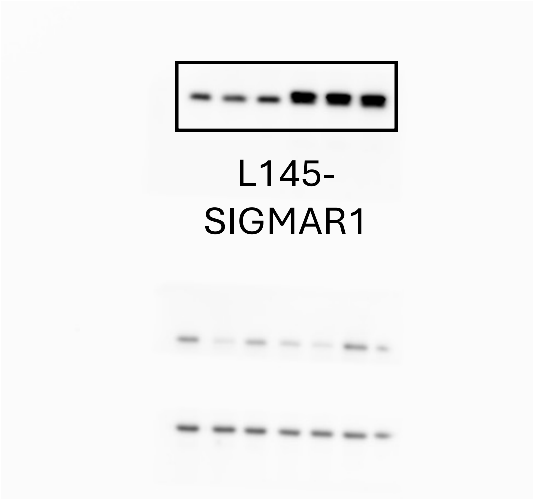

L145-
SIGMAR1

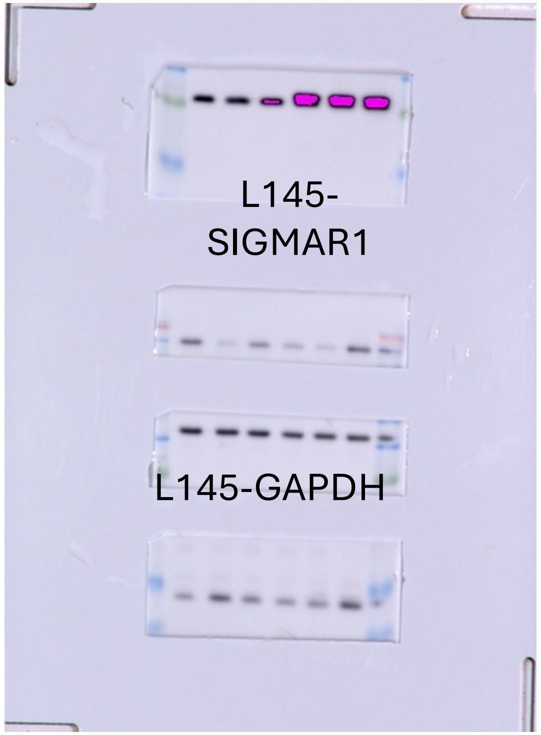

L145-
SIGMAR1

L145-GAPDH

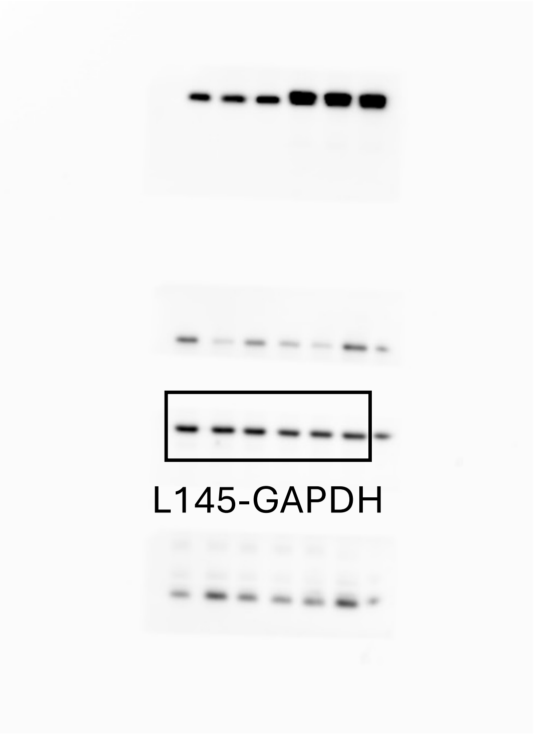

L145-GAPDH

RT112 SIGMAR1 OE
2020.6.18 n=1
BGJ398-PD-L1, SIGMAR1, GAPDH

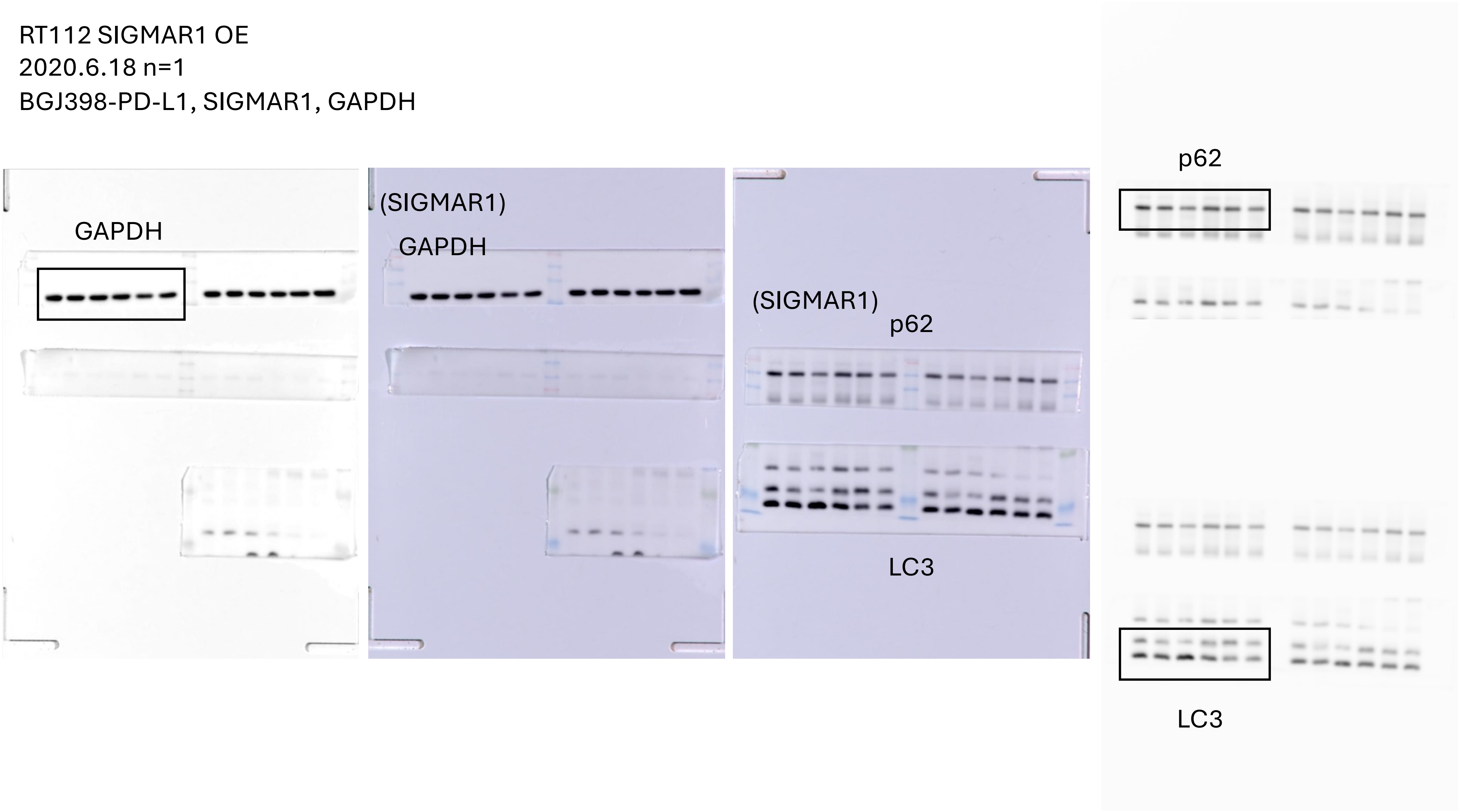

RT112 SIGMAR1 OE
2020.7.30 n=2
BGJ398-PD-L1, SIGMAR1, GAPDH

8/6 RT112
補 GAPDH

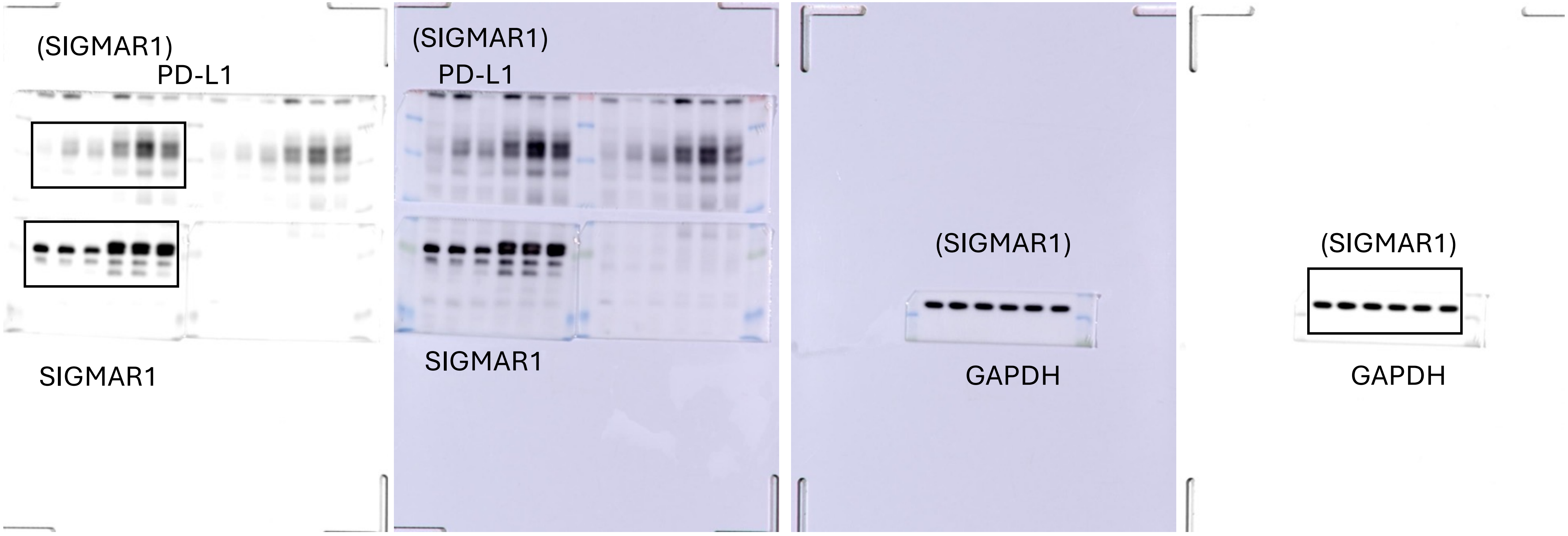

F3E RT112, SIGMAR1 OE (BGJ398)

2020.8.6 n=3

BGJ398-PD-L1, SIGMAR1, GAPDH

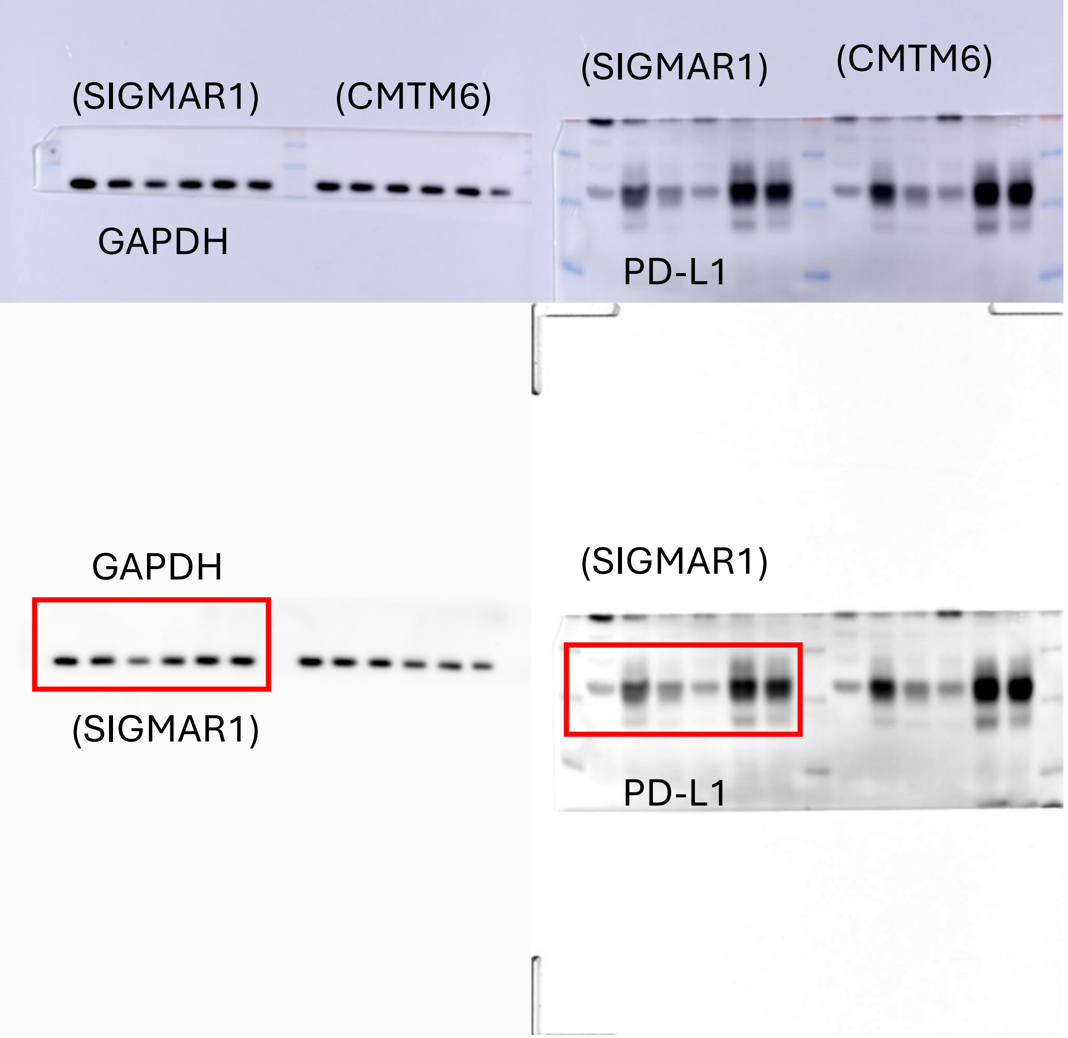

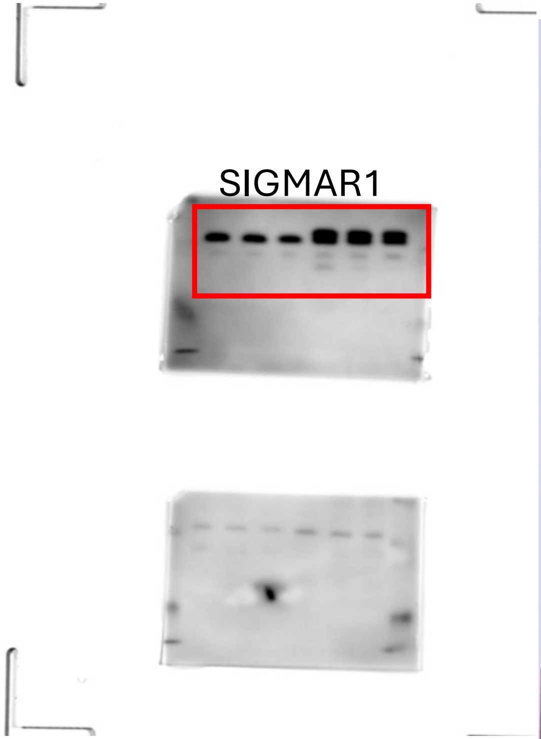

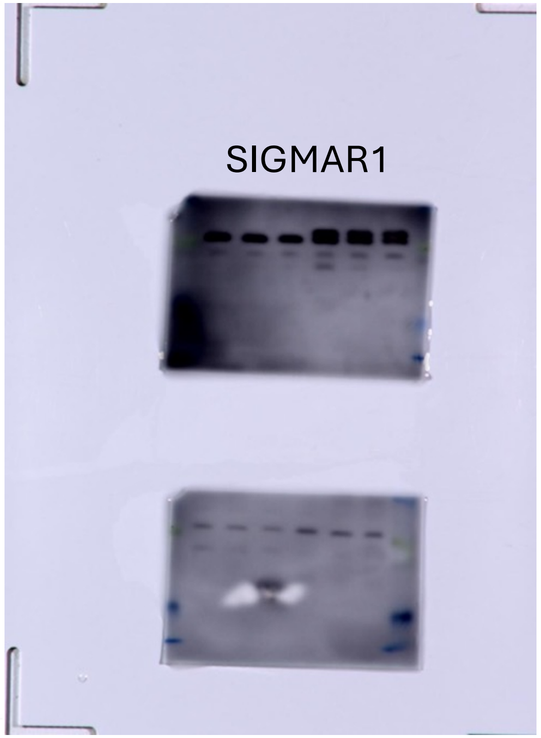

RT112 SIGMAR1 OE
2020.8.13 n=4
BGJ398-PD-L1, SIGMAR1, GAPDH

(SIGMAR1)

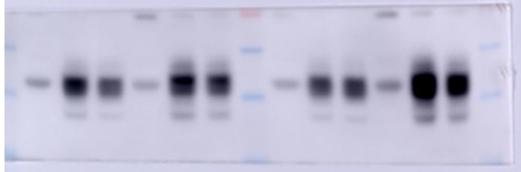

PD-L1

(SIGMAR1)

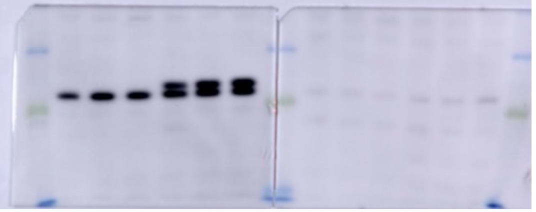

(SIGMAR1)

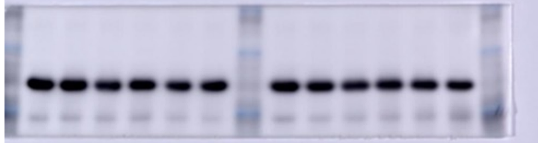

GAPDH

(SIGMAR1)

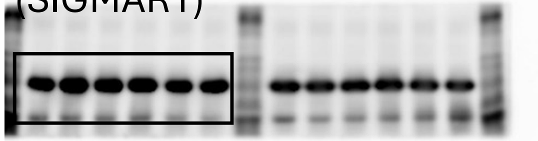

GAPDH

(SIGMAR1)

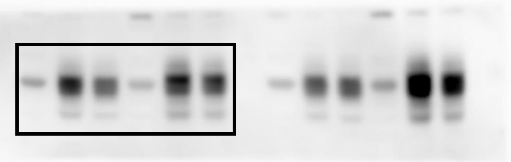

PD-L1

(SIGMAR1)

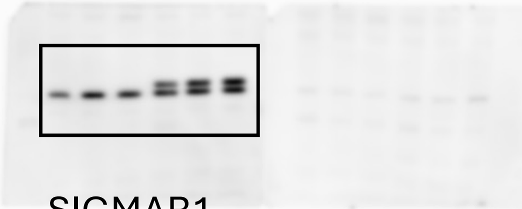

SIGMAR1

F3F RT112 (SA4503)

2022.8.26 n=1

L145-PD-L1, GAPDH

BGJ398-PD-L1, GAPDH

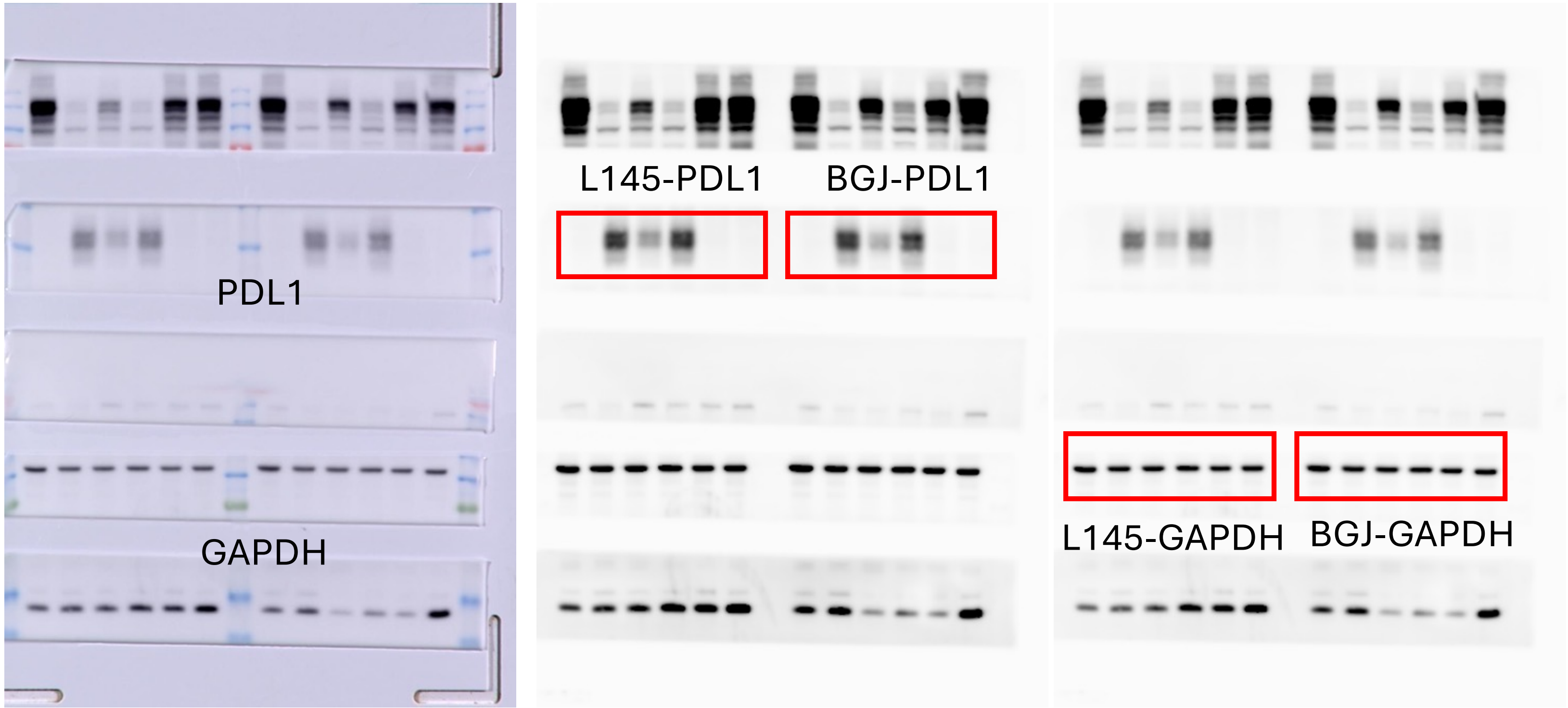

RT-112 (SA4503)
2023.8.25 n=2
L145-PD-L1, GAPDH
BGJ398-PD-L1, GAPDH

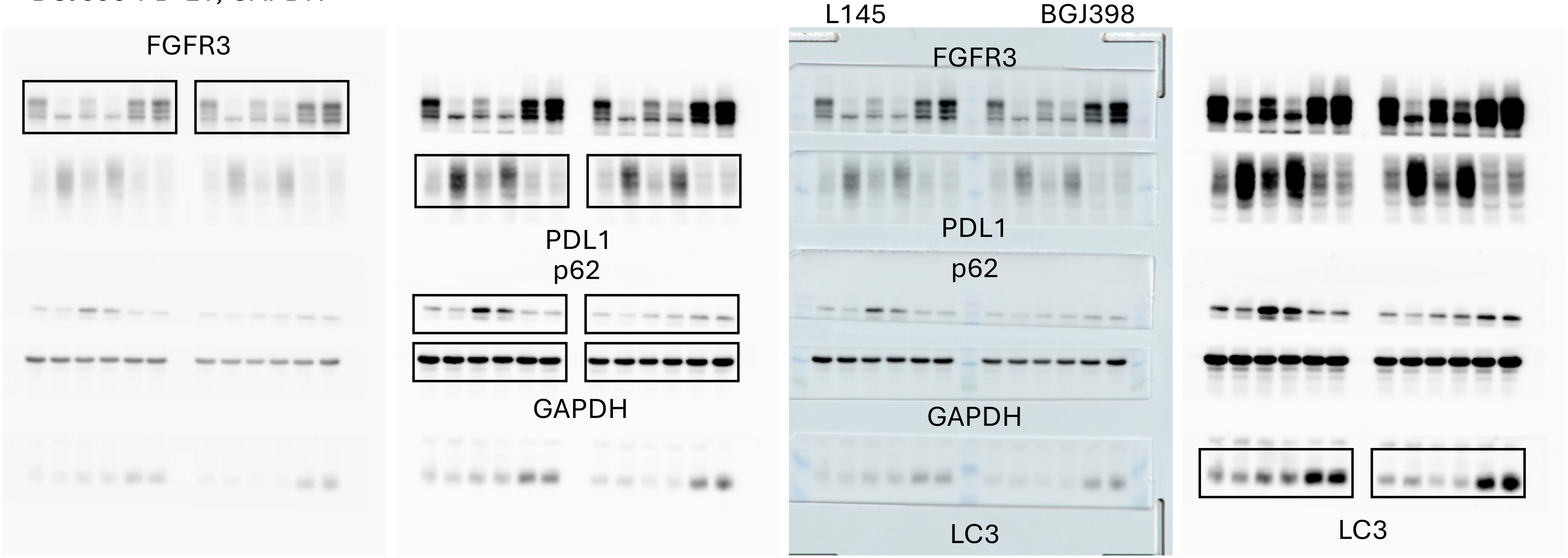

RT-112 (SA4503)
2023.8.25 n=3
L145-PD-L1, GAPDH
BGJ398-PD-L1, GAPDH

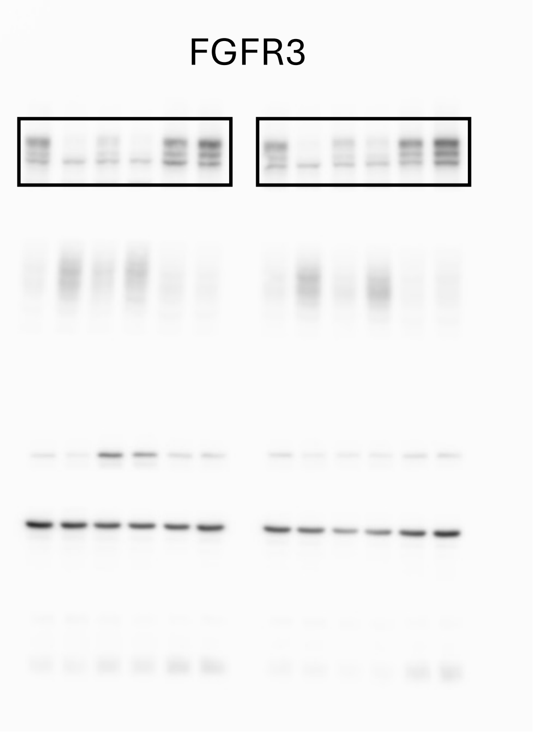

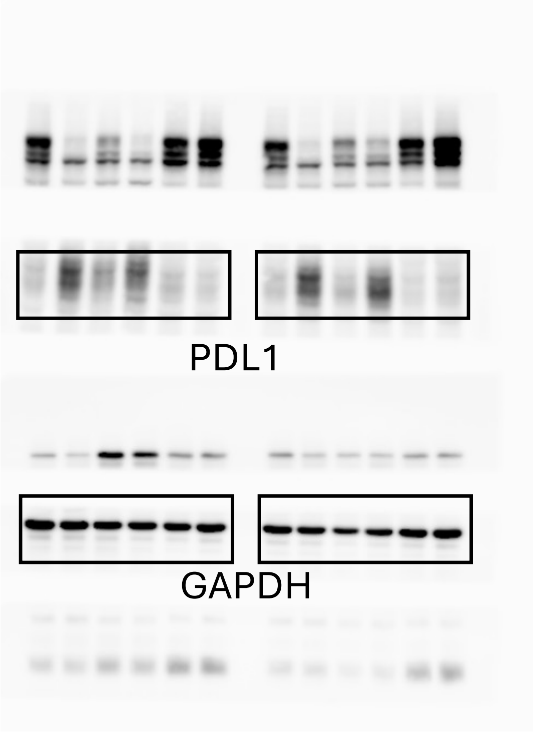

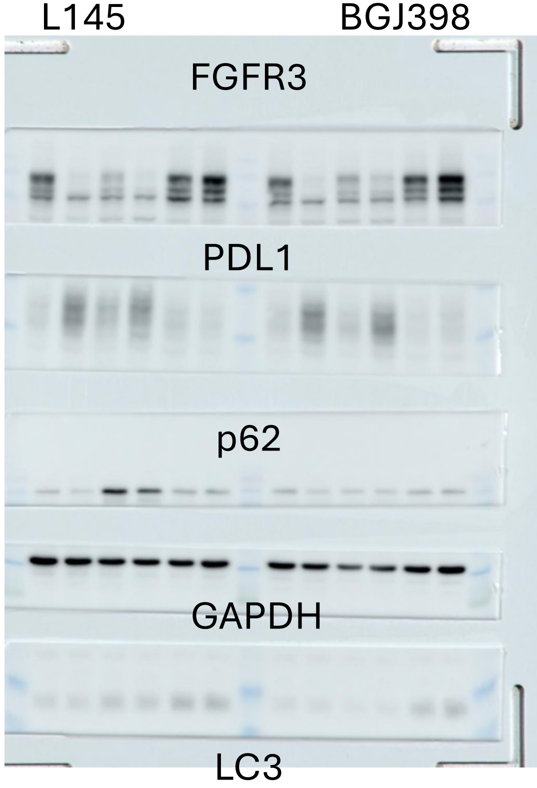

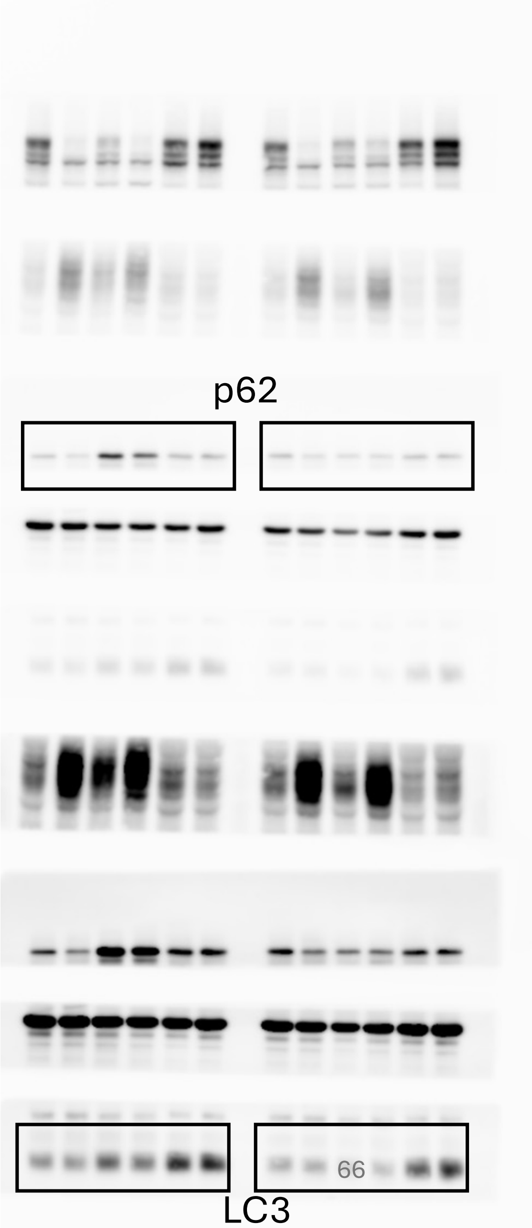

2021.1.05 RT112 n=1
L145-FGFR3, PD-L1, GAPHD
BGJ398-FGFR3, PD-L1, GAPHD

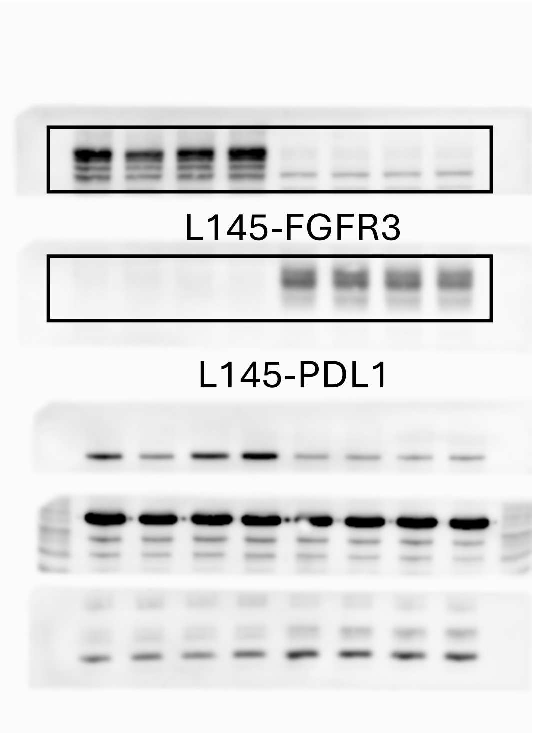

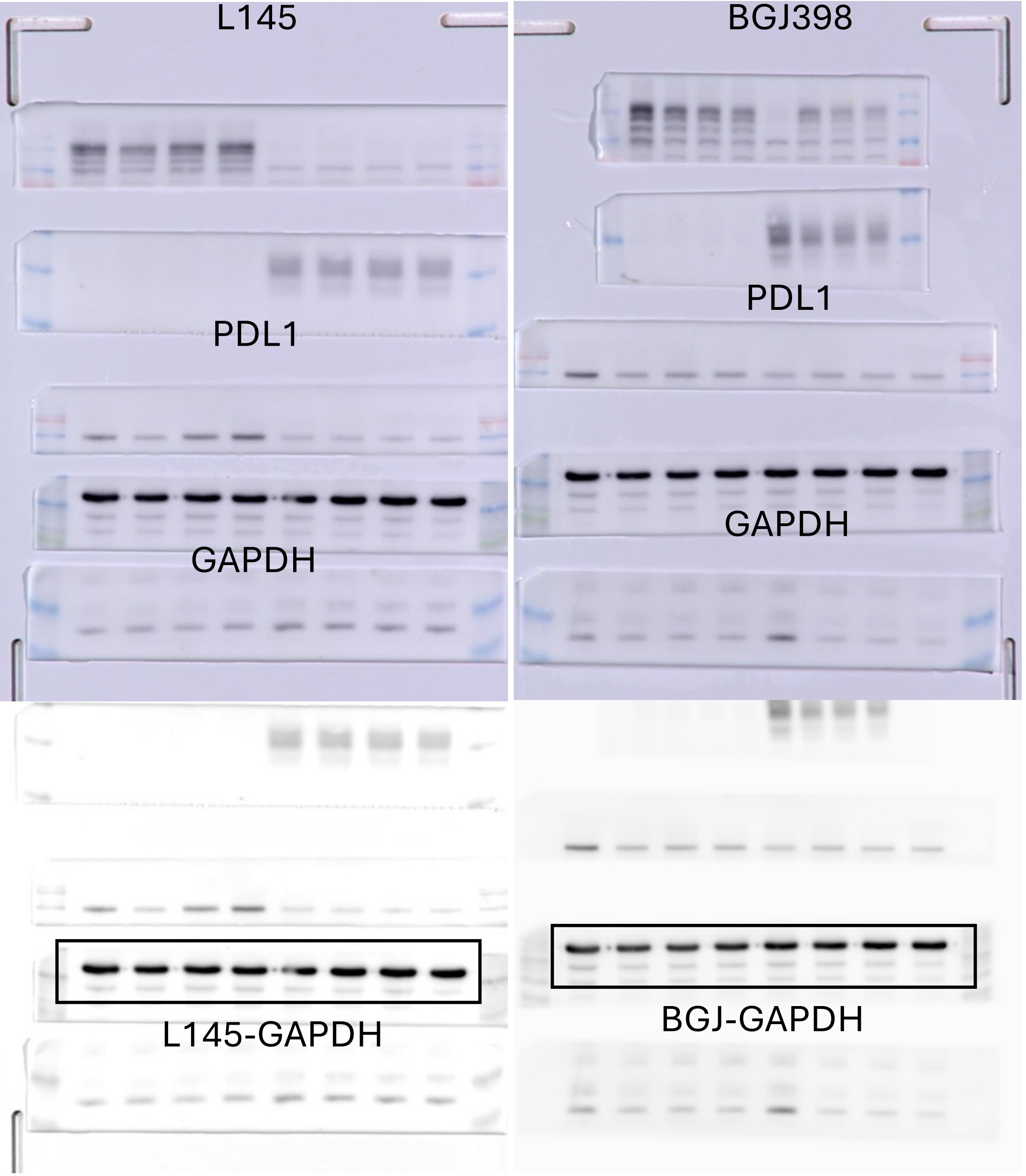

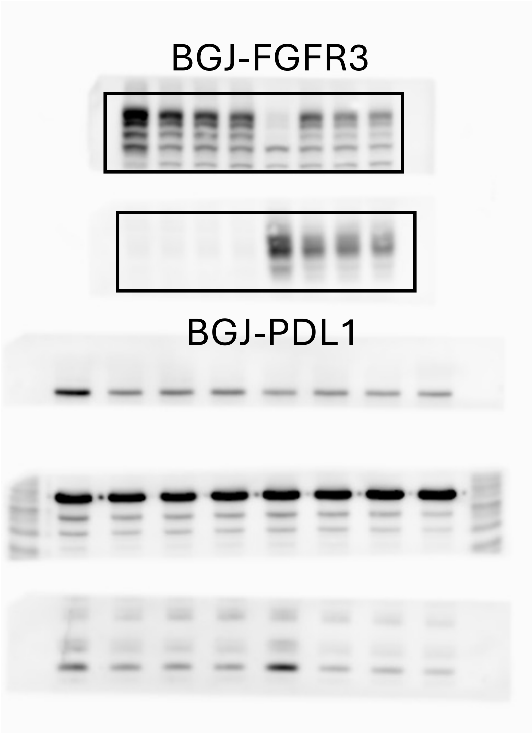

2021.1.26 RT112 n=2
L145-FGFR3, PD-L1, GAPHD
BGJ398-FGFR3, PD-L1, GAPHD

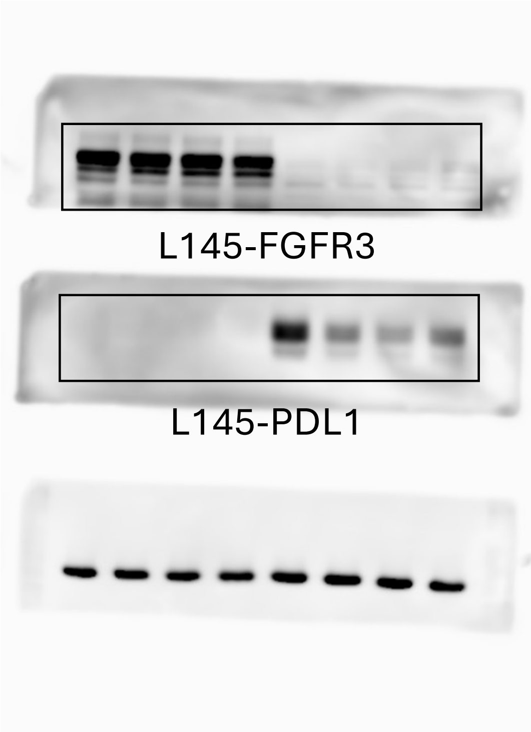

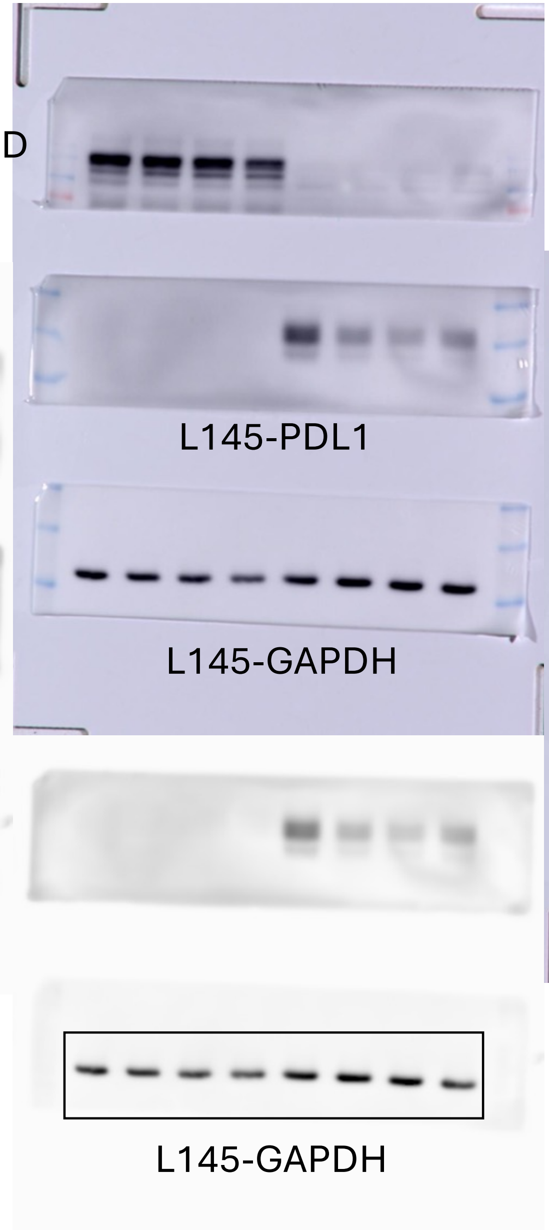

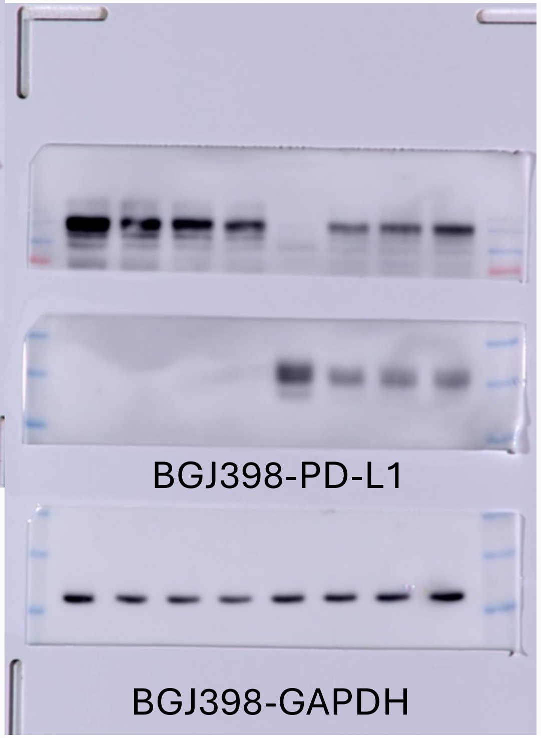

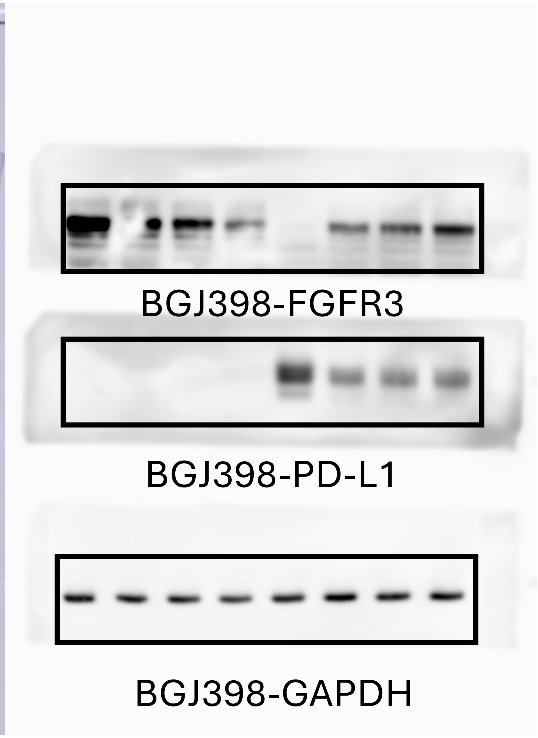

F5A RT112 (L145, BGJ398)

2022.04.21 n=3

L145-FGFR3, PD-L1, GAPDH

BGJ398-FGFR3, PD-L1, GAPDH

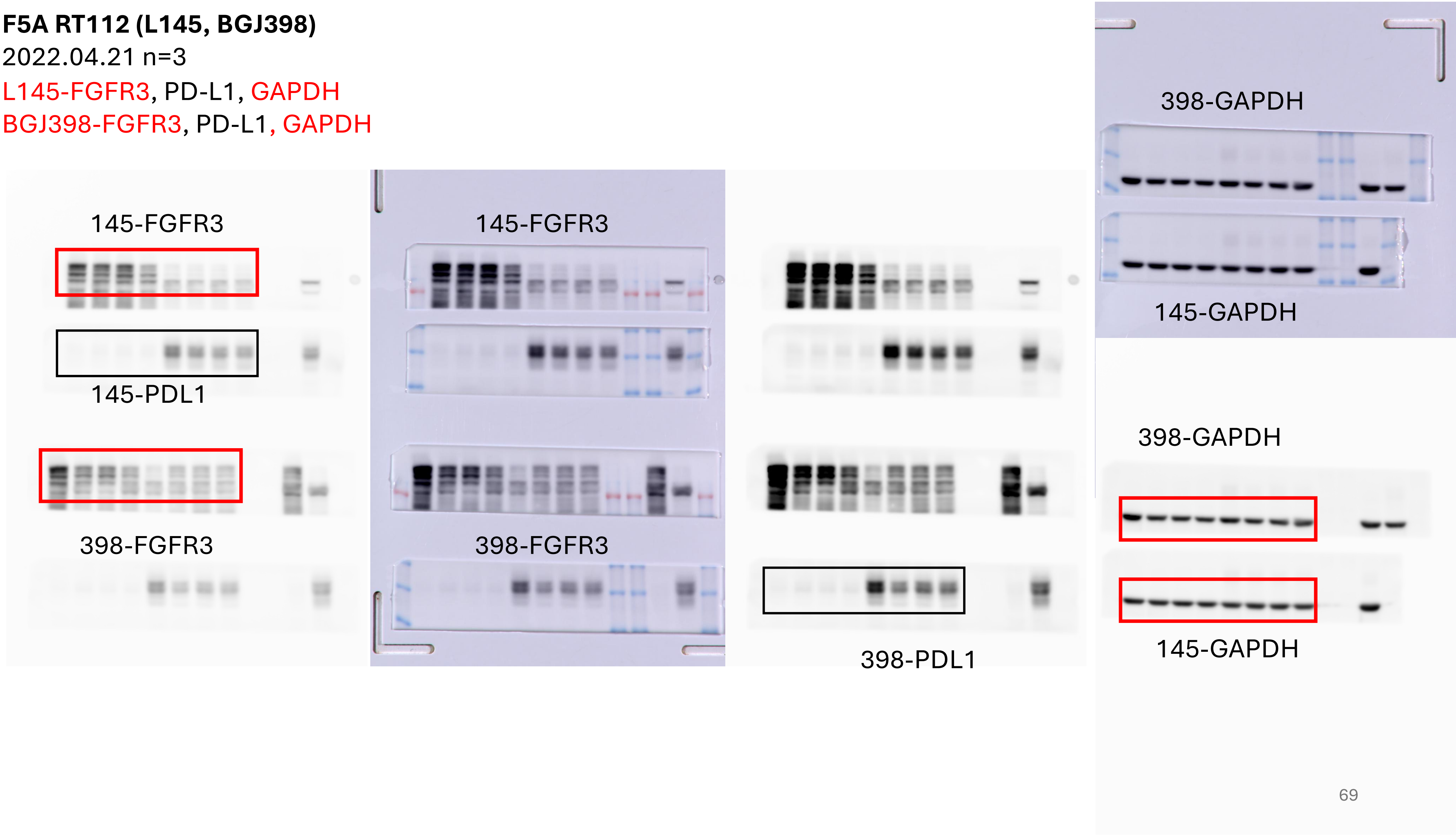

F5A RT4 (L145)

2023.03.17 n=1

L145-FGFR3, GAPDH

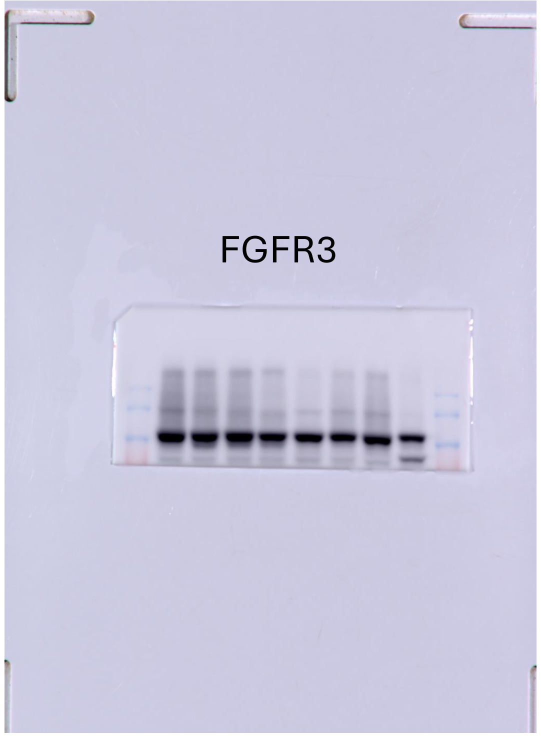

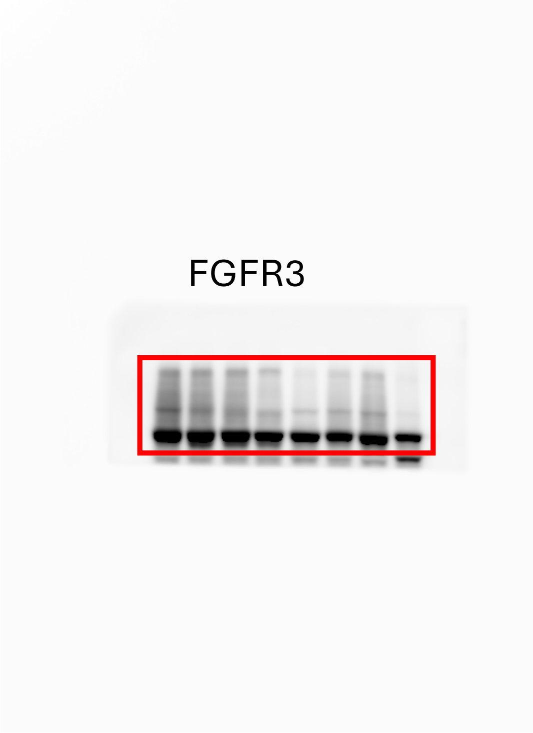

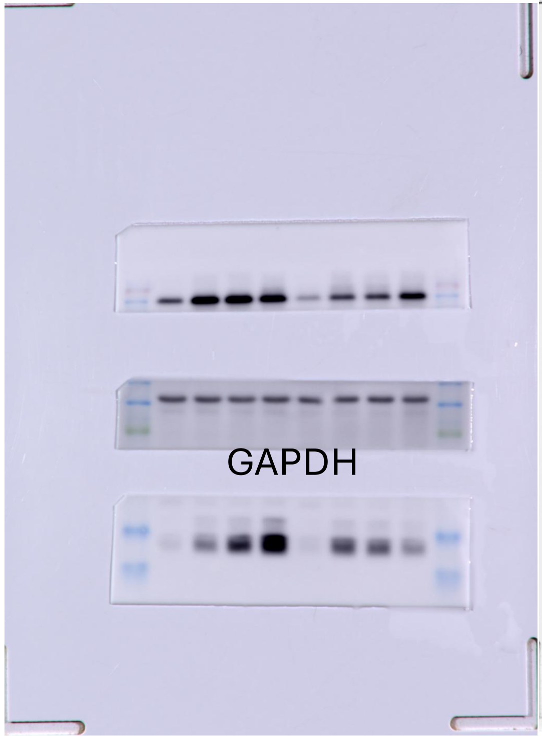

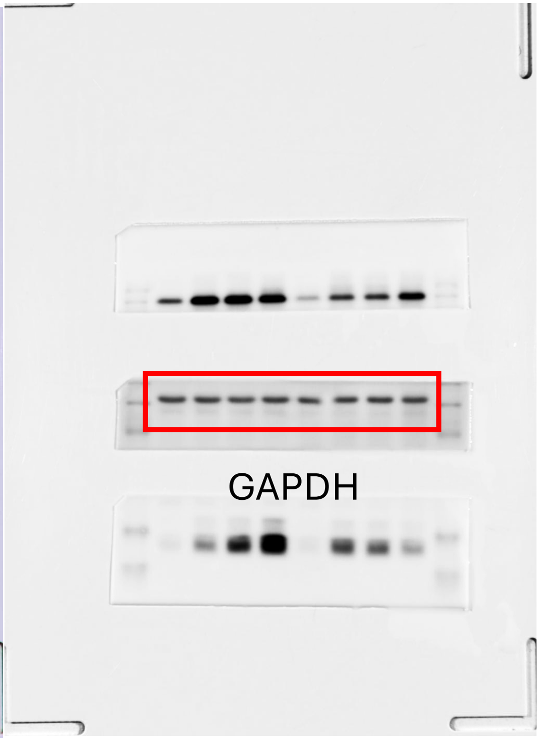

2022.7.25 RT112
L145-FGFR3, GAPHD n=2
BGJ398-FGFR3, GAPHD n=1

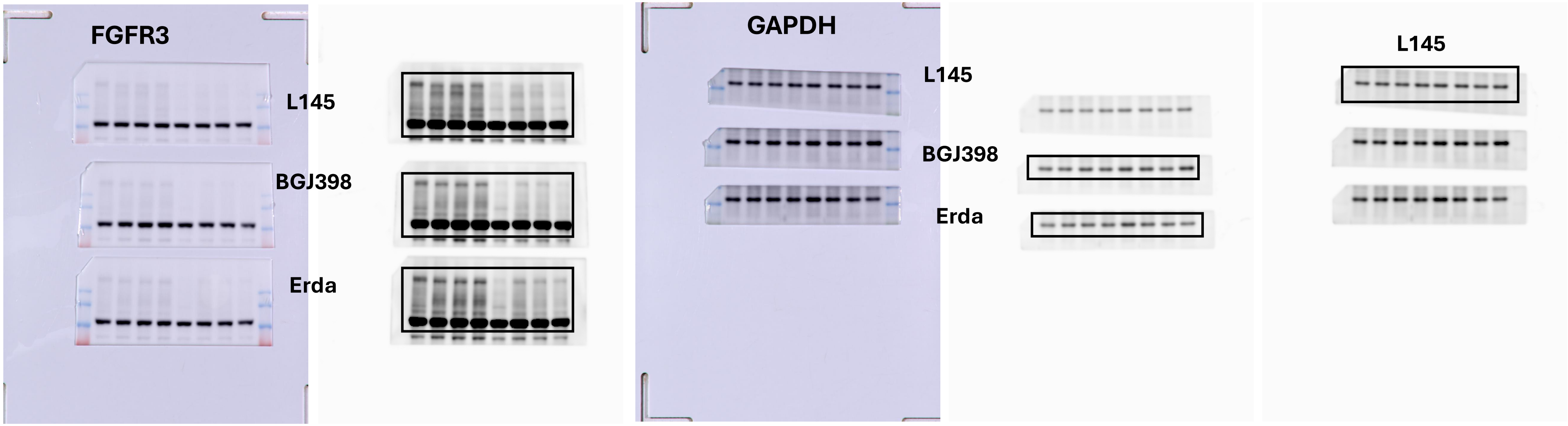

F5A RT4 (BGJ398)

2022.08.15 RT112

L145-FGFR3, GAPHD n=3

BGJ398-FGFR3, GAPDH n=2

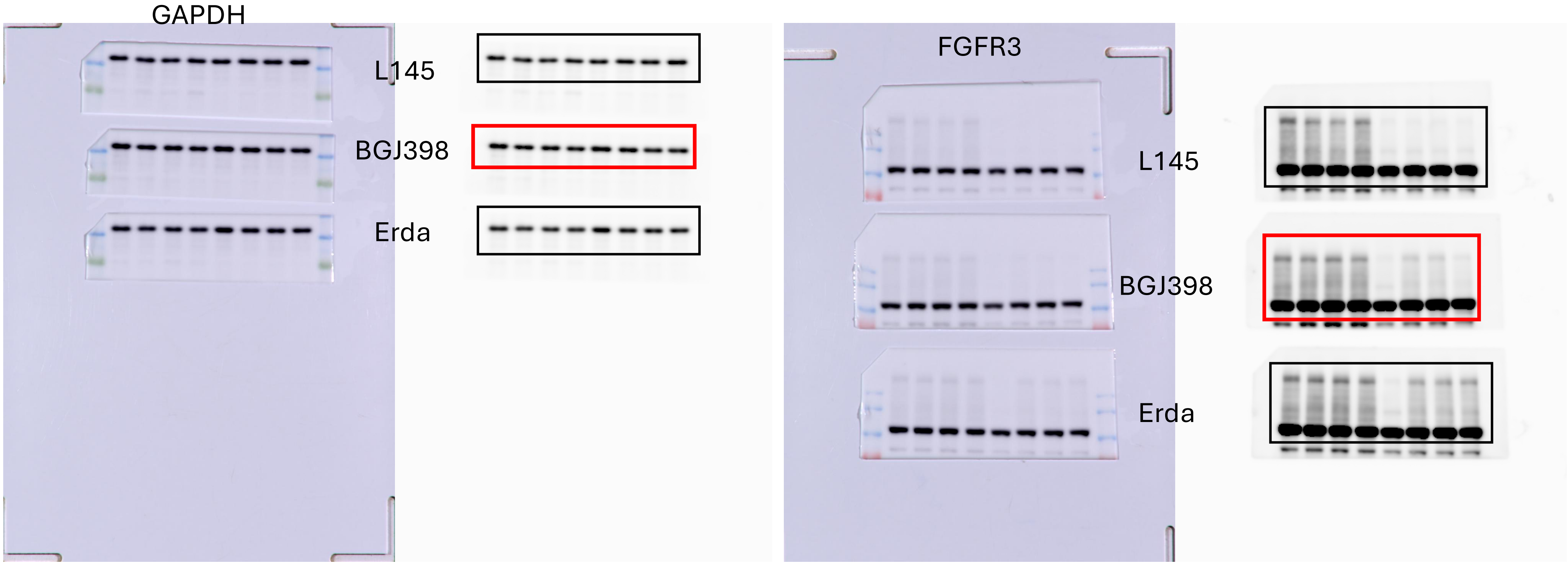

2022.9.14 RT112
L145-FGFR3, GAPHD n=4
BGJ398-FGFR3, GAPHD n=3

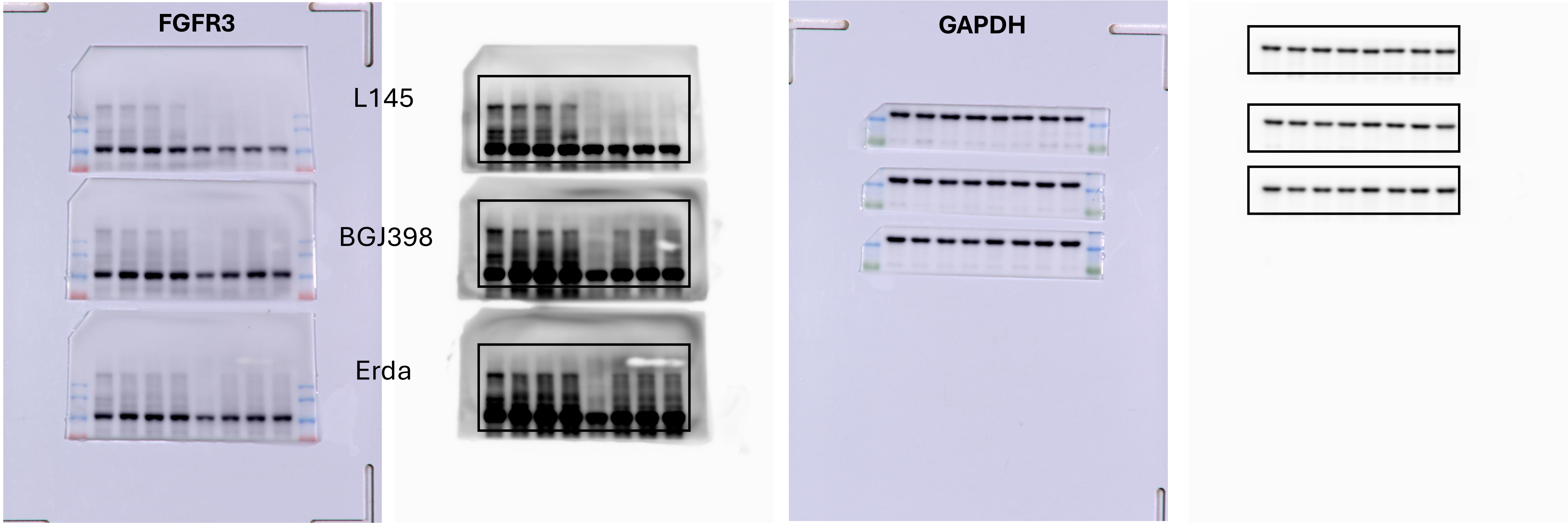

2022.4.19 RT112 n=1
L145-FGFR3, PD-L1,GAPDH

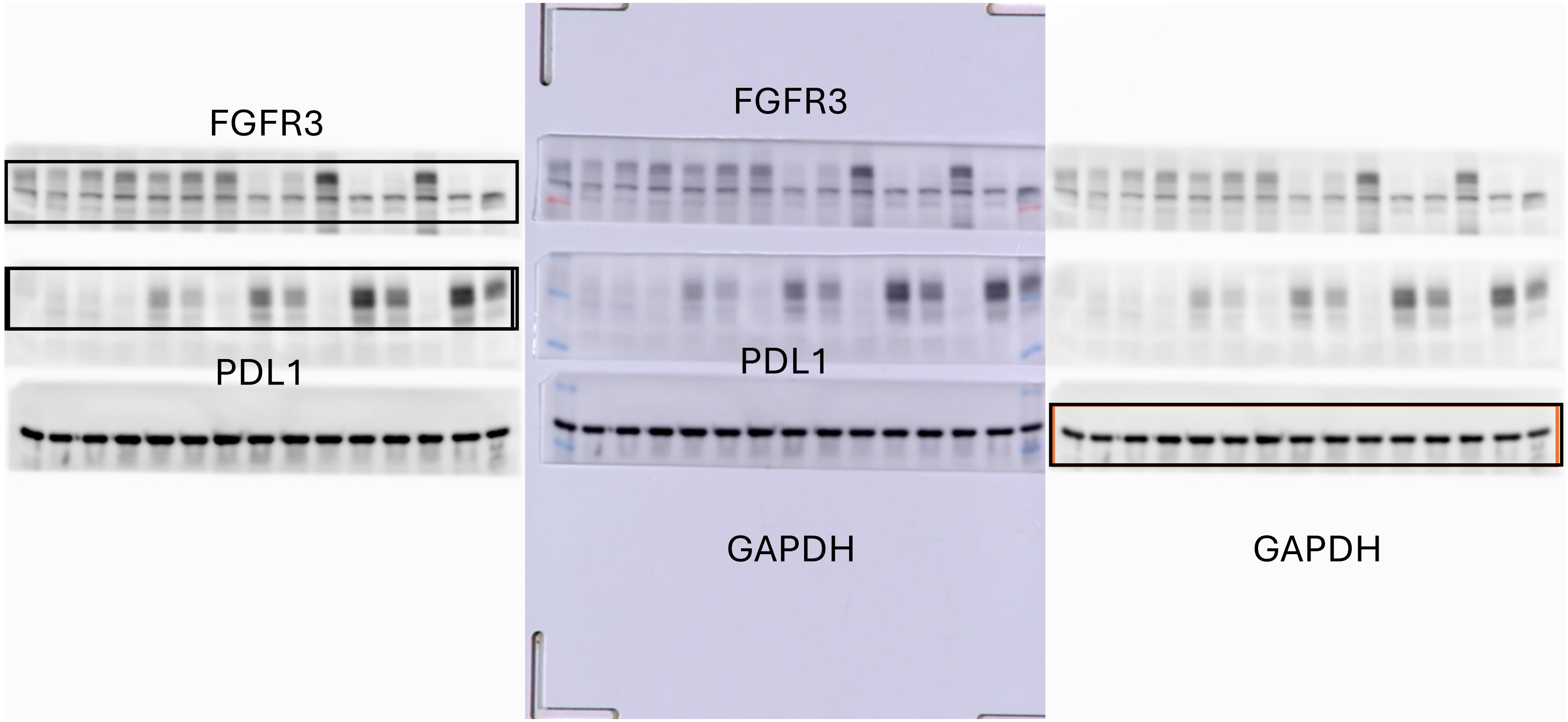

2022.4.19 RT112 n=1
BGJ398-FGFR3, PD-L1,GAPDH

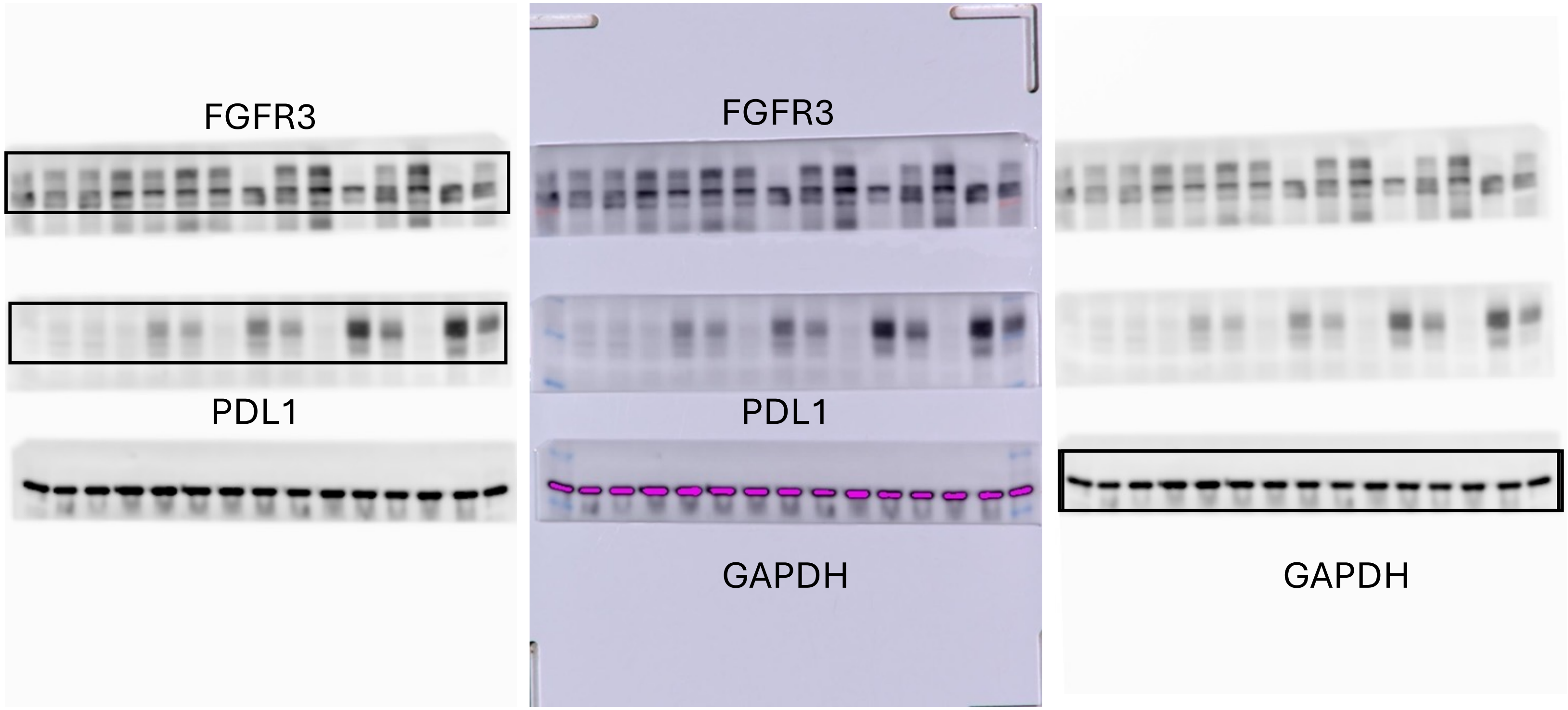

F5B RT-112 time course (L145)

2022.05.20 n=2

L145-FGFR3, PD-L1, GAPDH

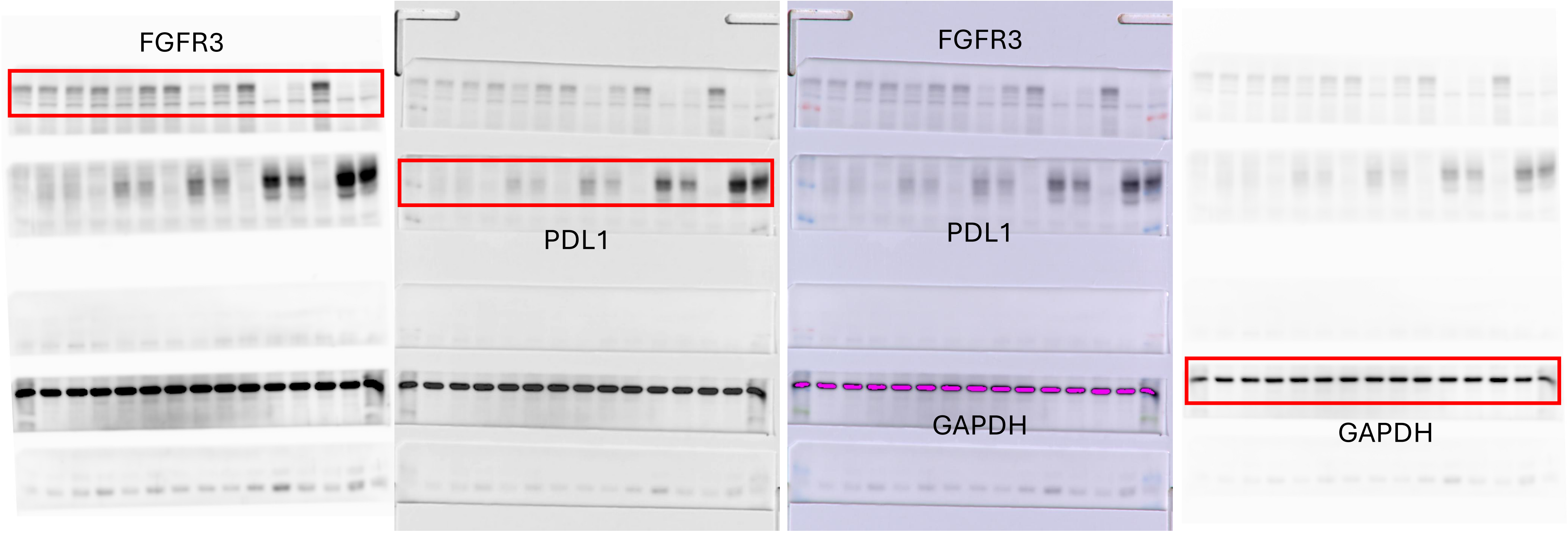

F5B RT-112 time course (BGJ398)
2022.05.20 n=2
BGJ398-FGFR3, PD-L1
2022.06.09
BGJ398-p62, LC3B, GAPDH

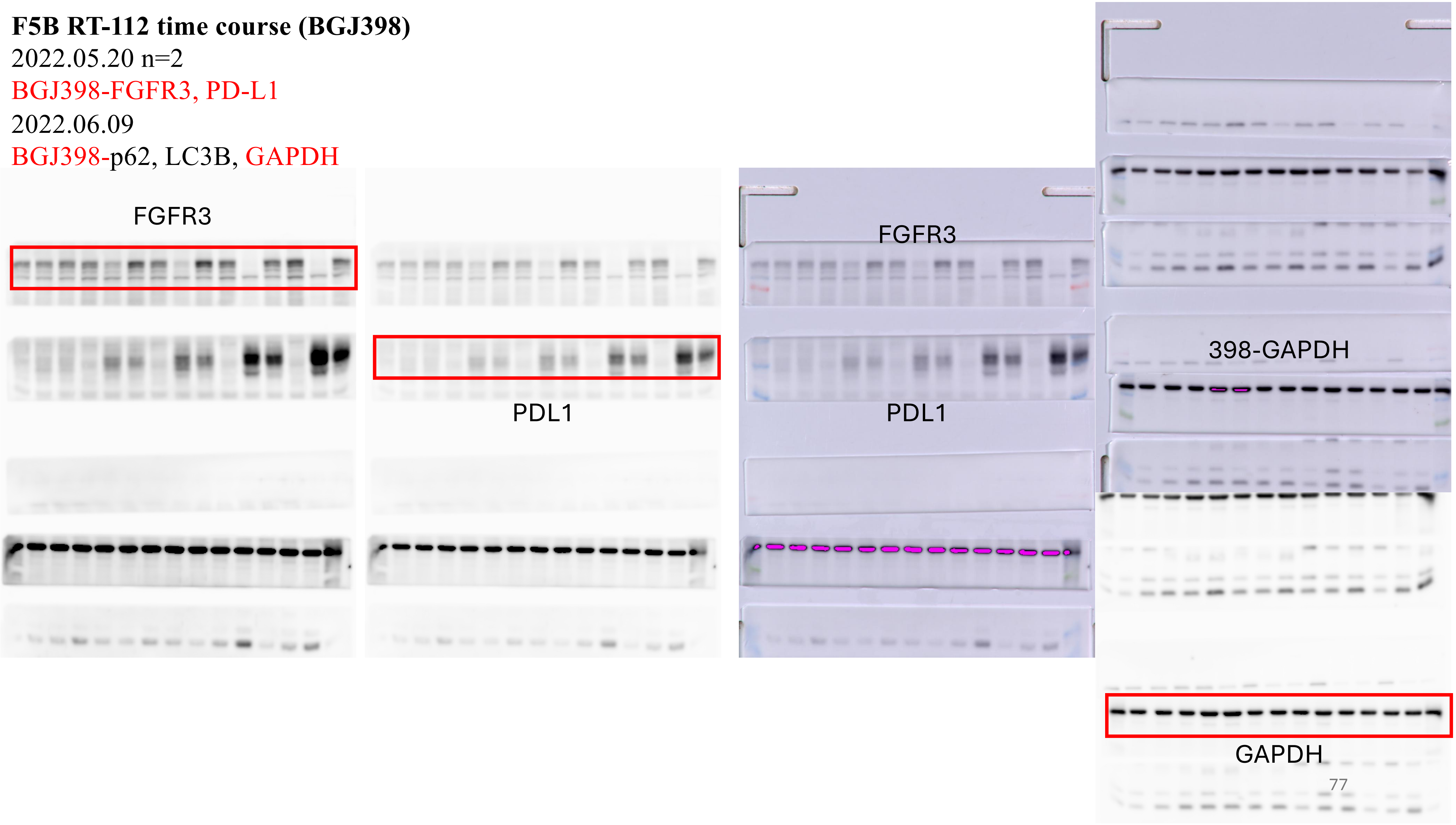

2022.6.17 RT112 n=3
L145-FGFR3, PD-L1,GAPDH

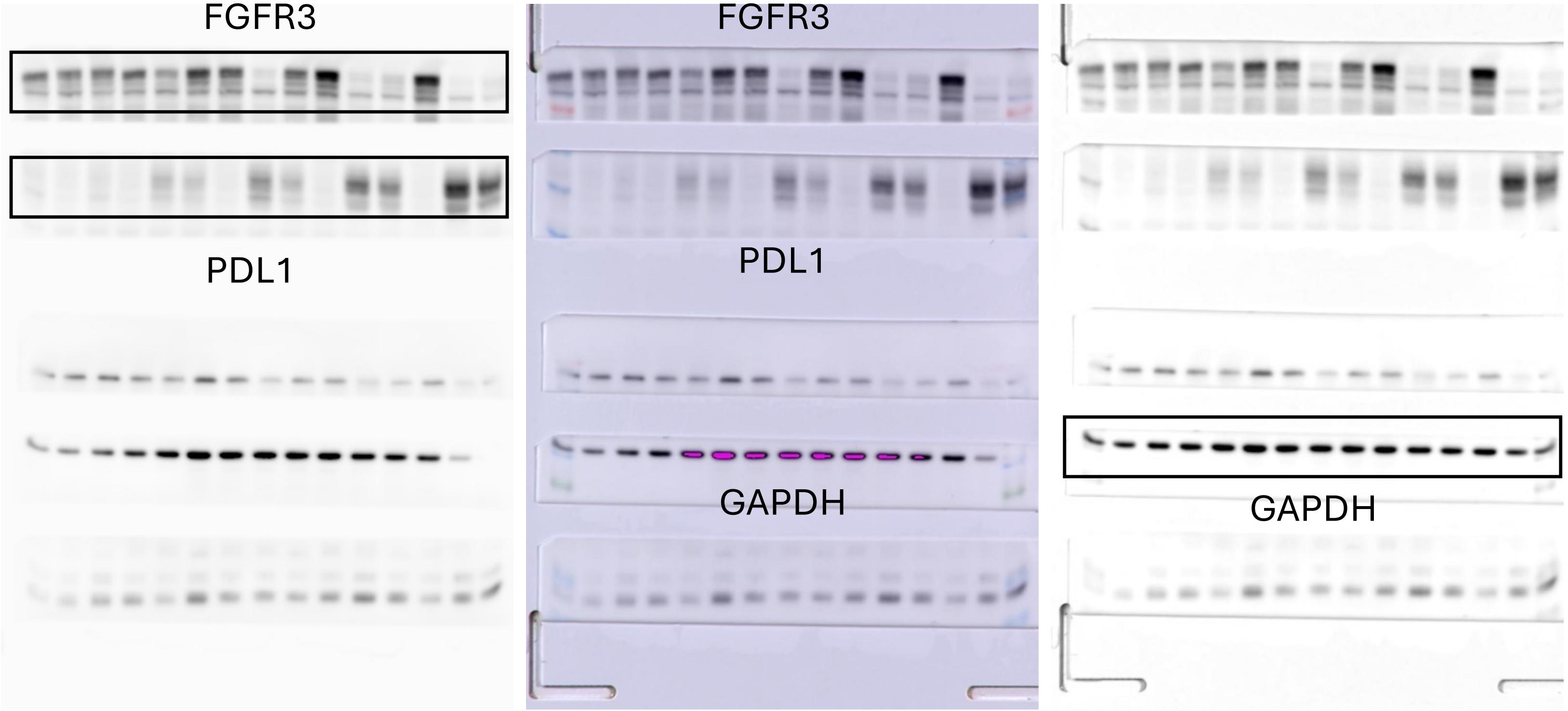

2022.6.17 RT112 n=3
BGJ398-FGFR3, PD-L1,GAPDH

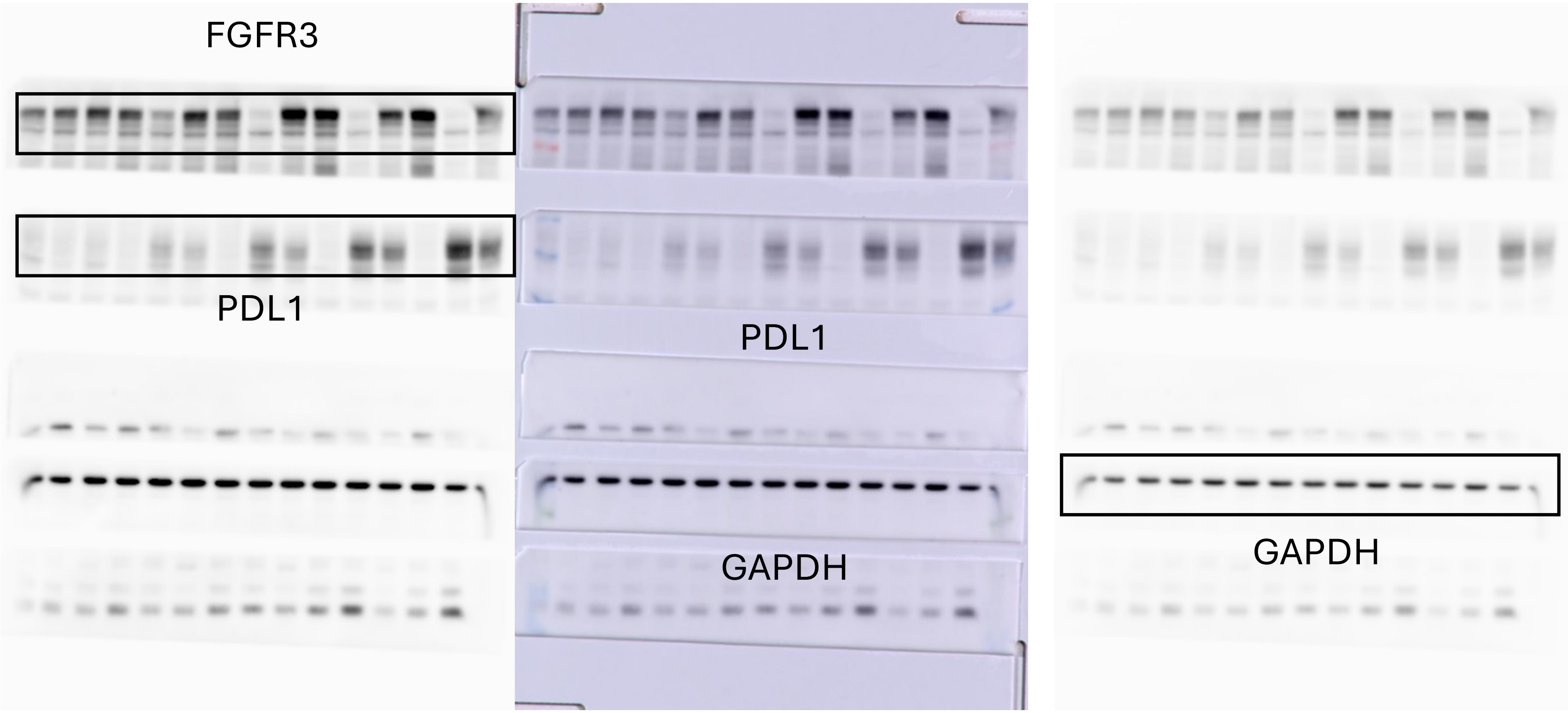

F5D RT-112 PD-L1 OE(stable)

2022.08.14 n=1, 2

FGFR3, PD-L1, GAPDH

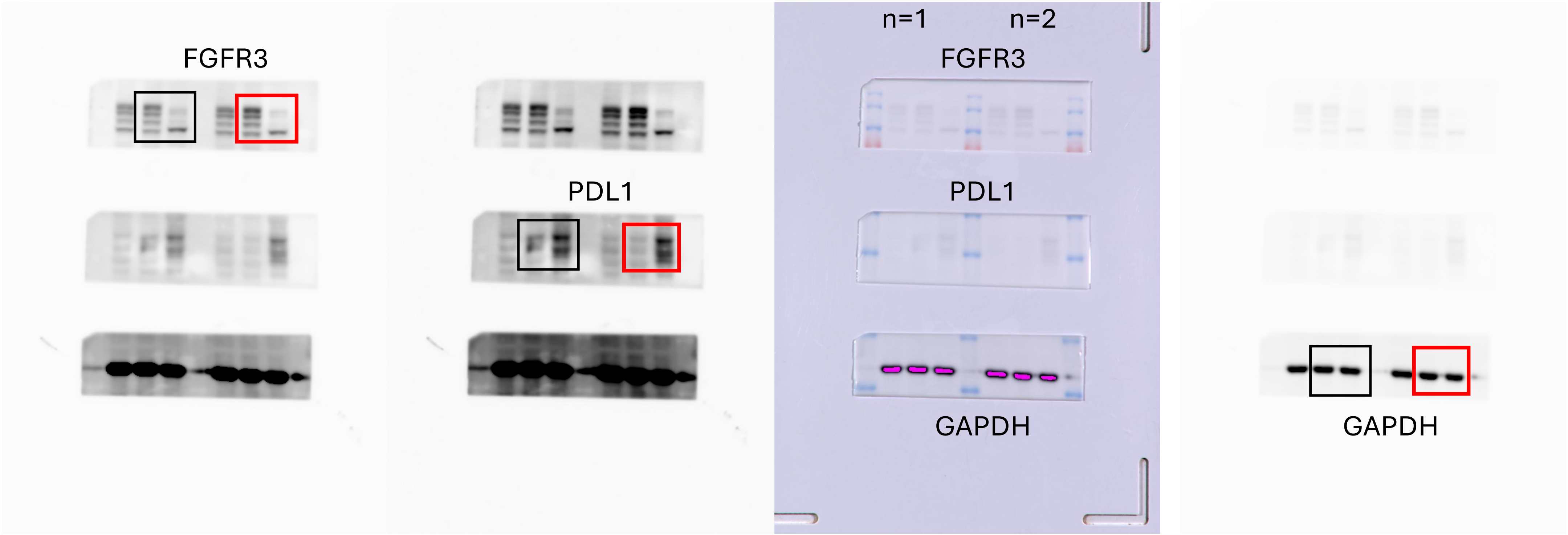

RT112 PD-L1 OE (stable)
2022.08.14 n=3
FGFR3, PD-L1, GAPDH

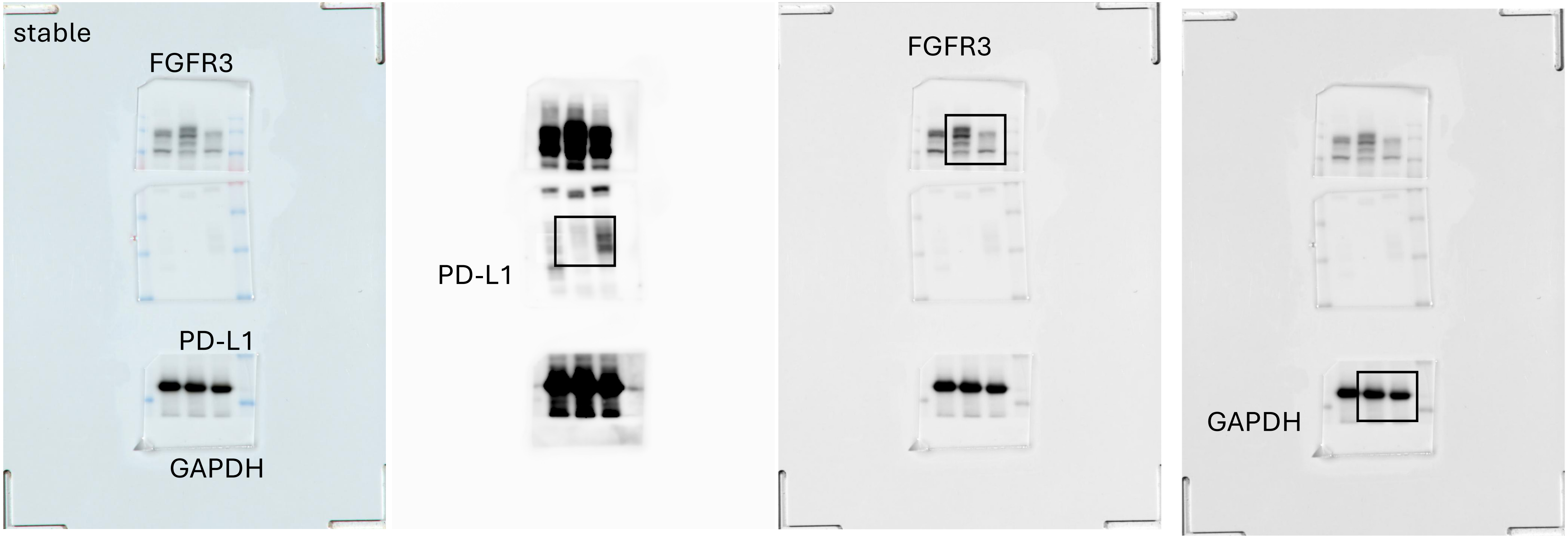

F5E RT-112 PD-L1 OE (transient)

2023.12.1 n=1, 2, 3

FGFR3, PD-L1, GAPDH

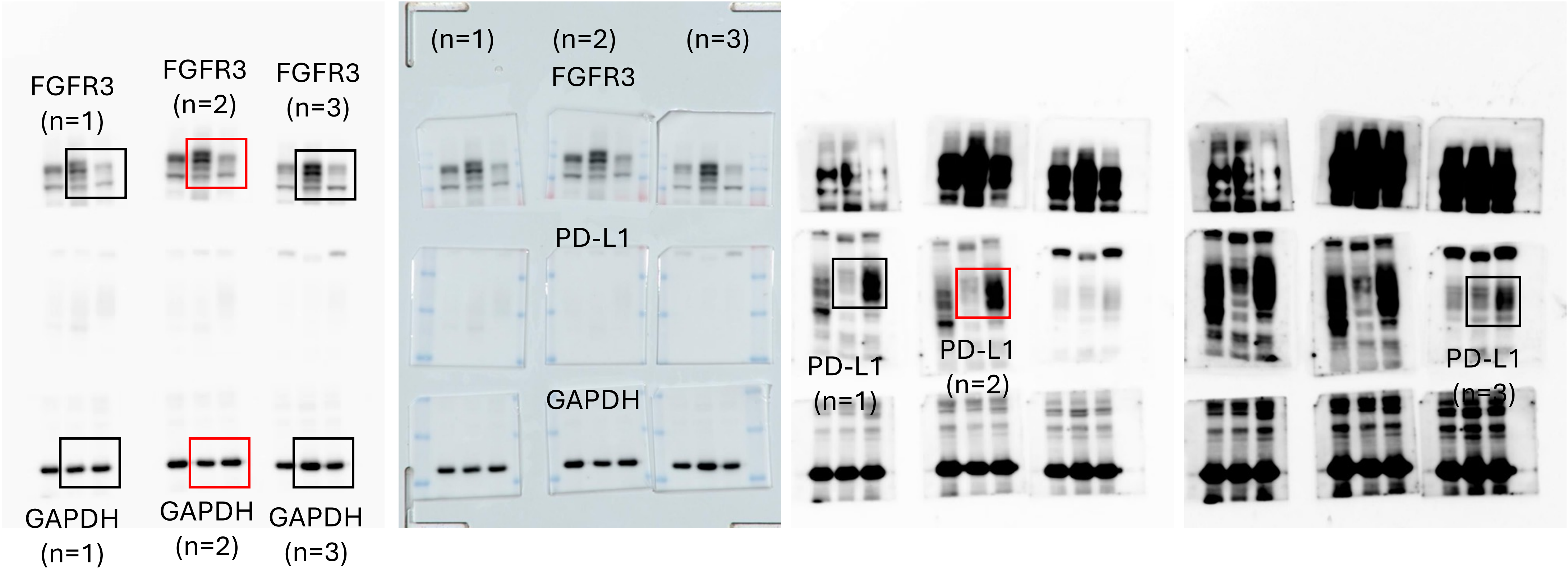

F5E RT4 PD-L1 OE (transient)

2023.12.7 n=1

FGFR3, PD-L1, HSP90, GAPDH

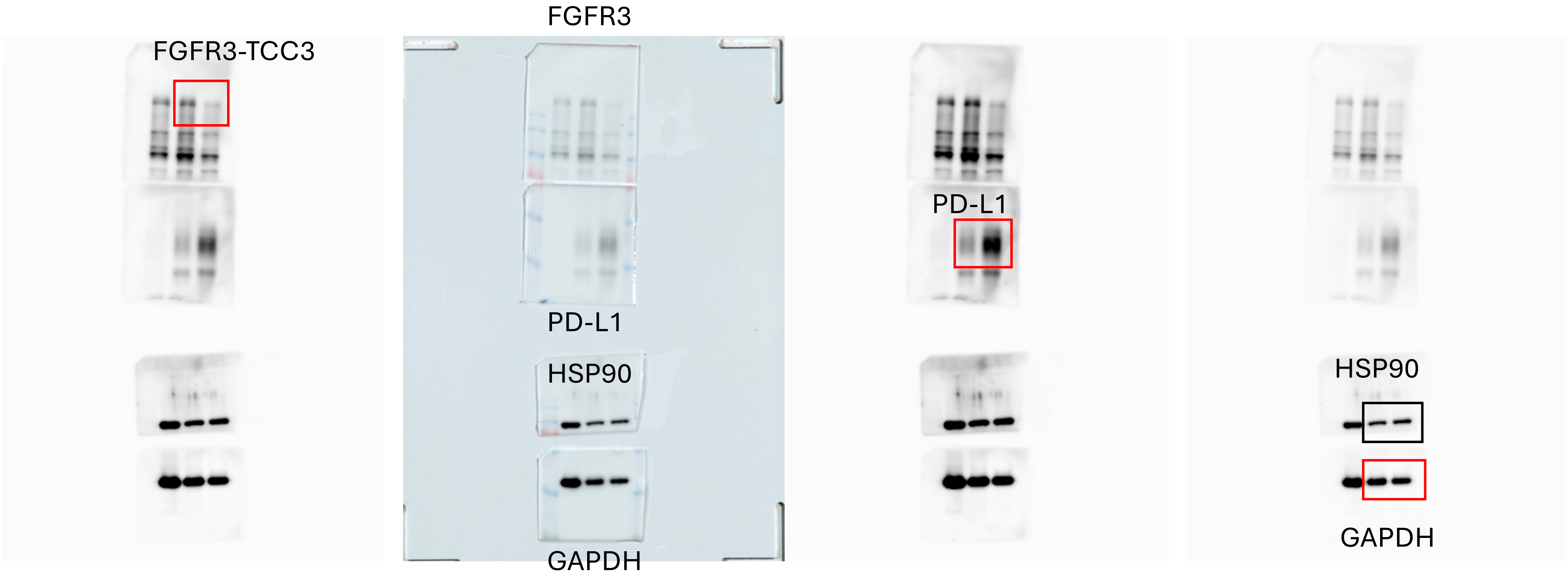

RT4 PD-L1 OE (transient)
2023.12.9 n=2
FGFR3, PD-L1, HSP90, GAPDH

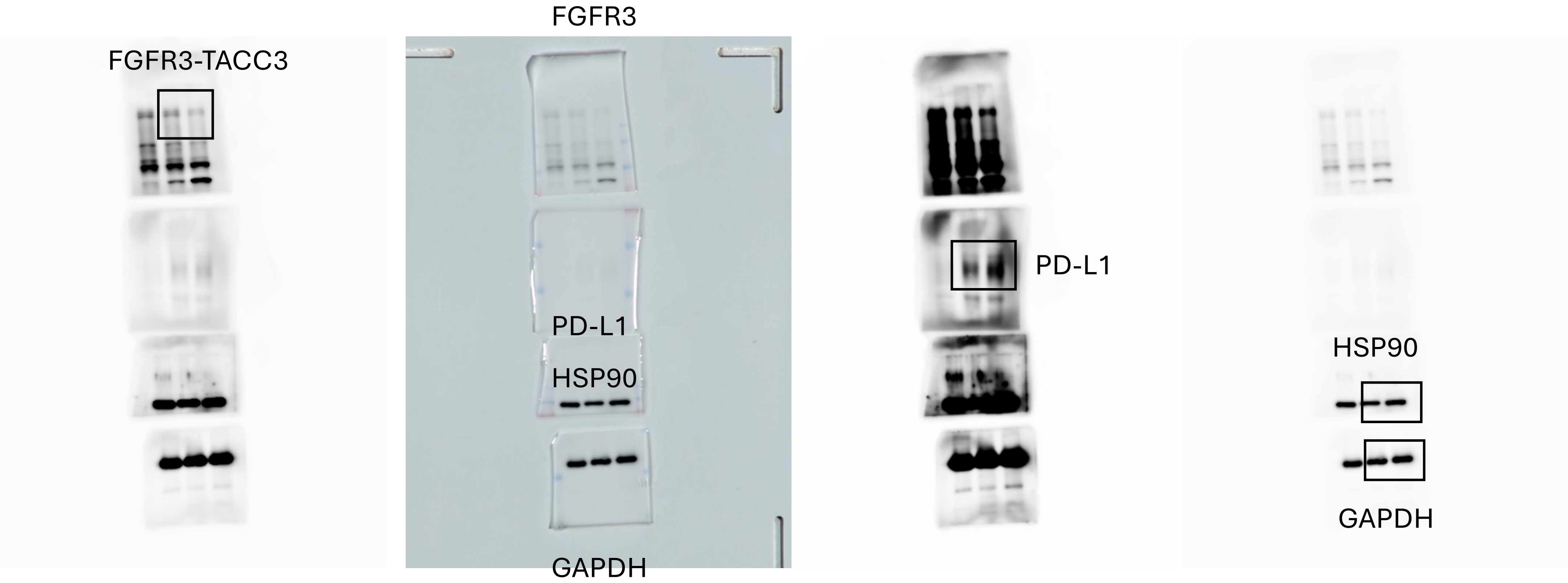

RT4 PD-L1 OE (transient)
2023.12.11 n=3
FGFR3, PD-L1, HSP90, GAPDH

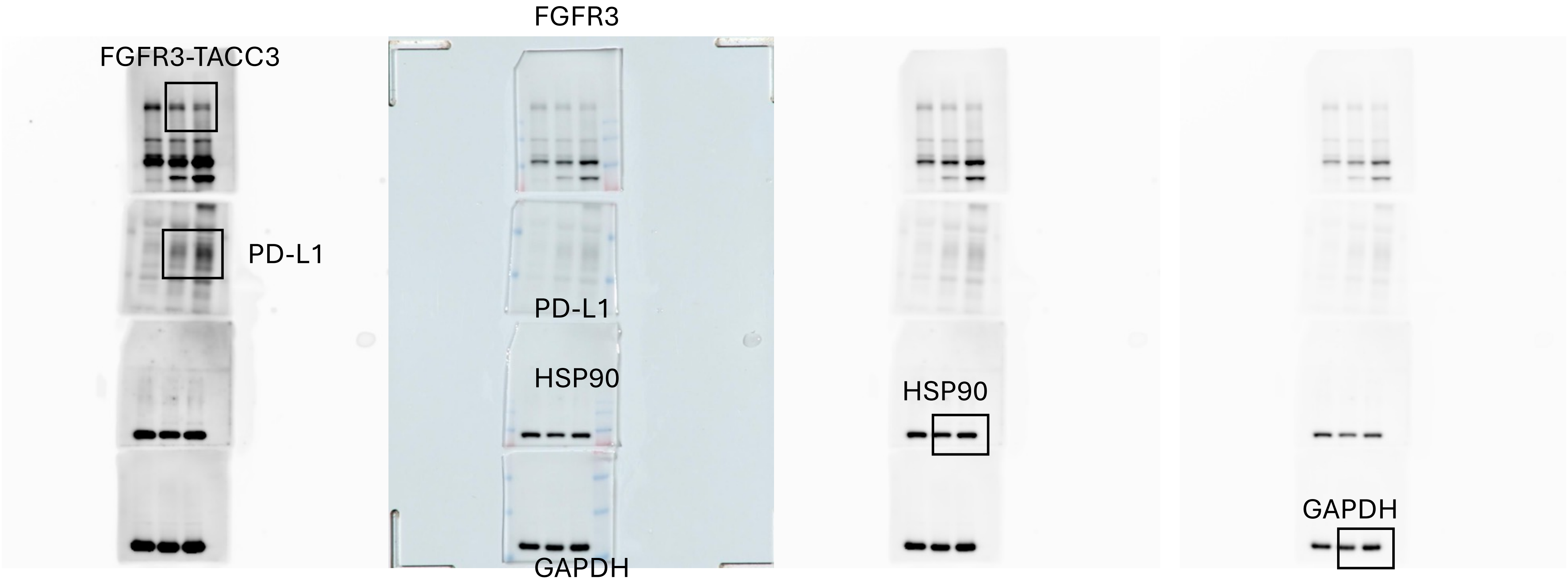

F5G RT-112 siPD-L1

2022.10.12 n=1

FGFR3, PD-L1, GAPDH

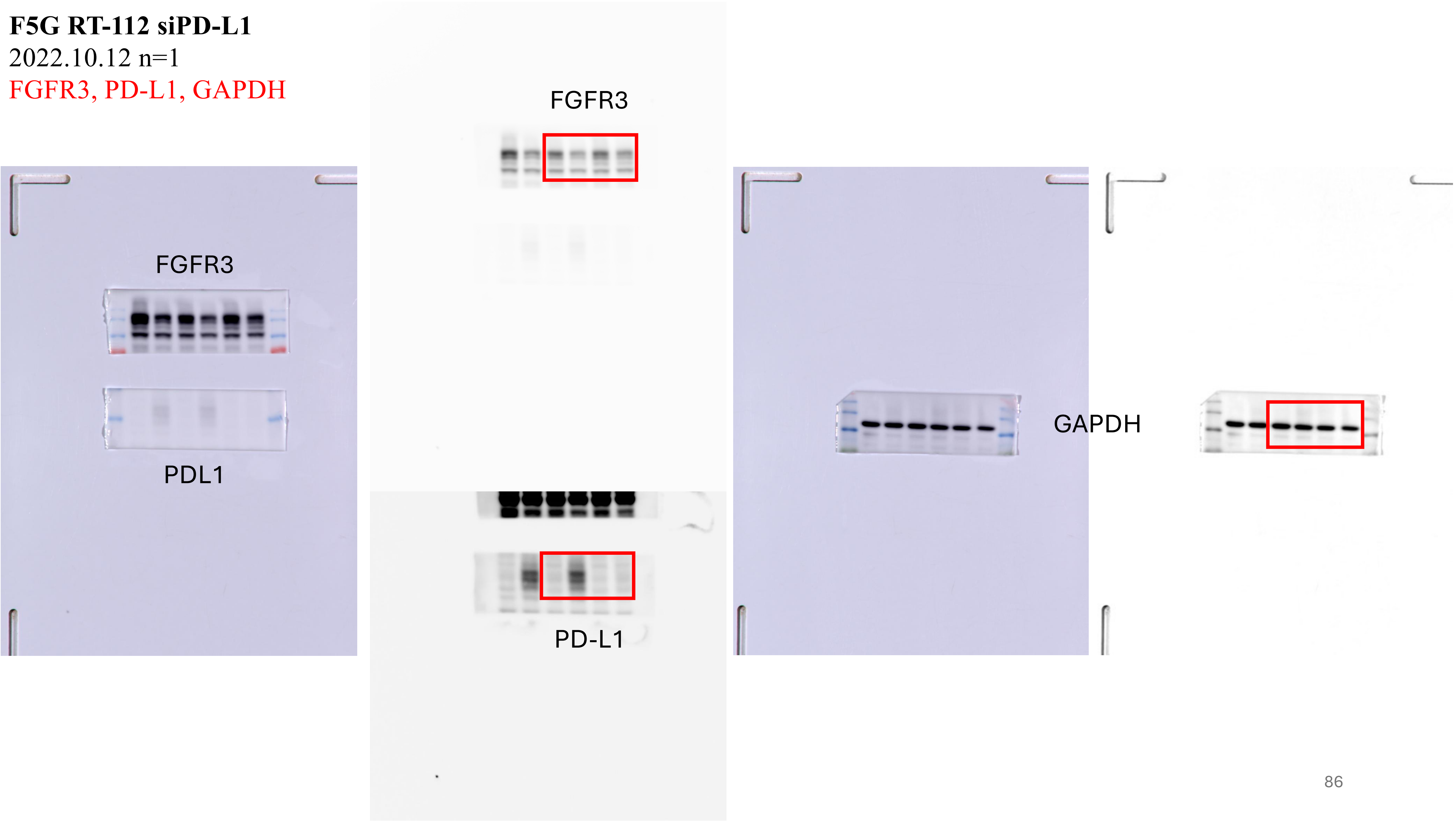

RT-112 siPD-L1
2022.10.27 n=2
FGFR3, PD-L1, GAPDH

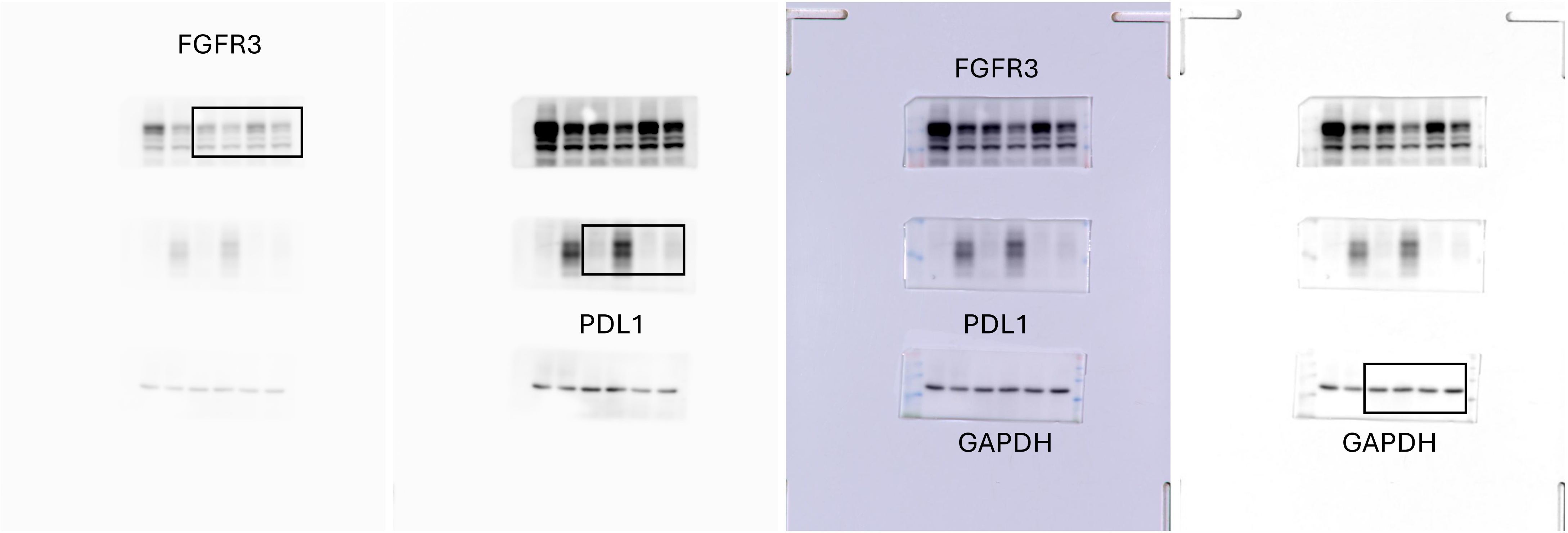

RT-112 siPD-L1
2022.10.27 n=3
FGFR3, PD-L1, GAPDH

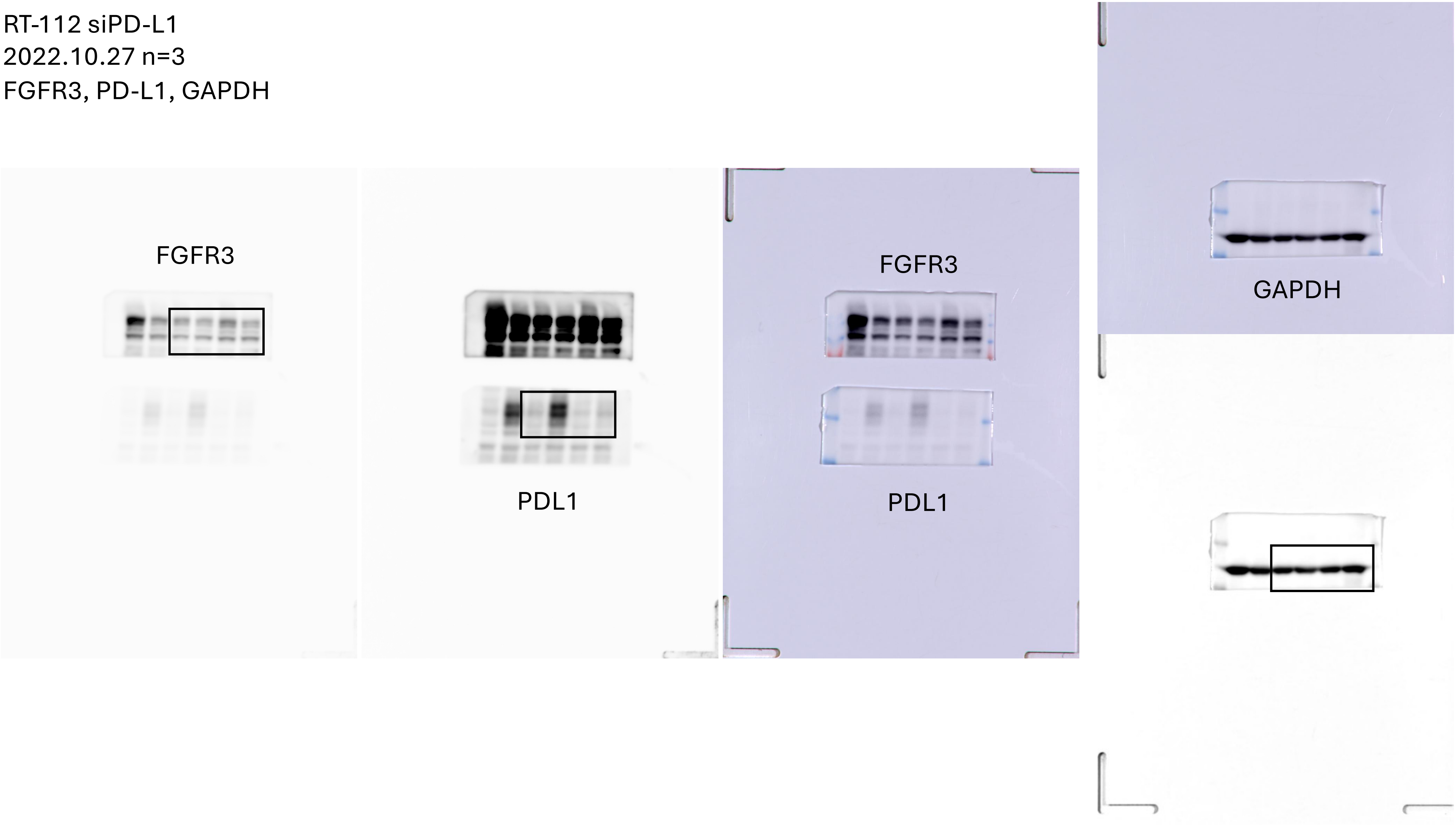

RT112 (Erda)

2022.5.20 n=1

FGFR3, PD-L1, GAPDH

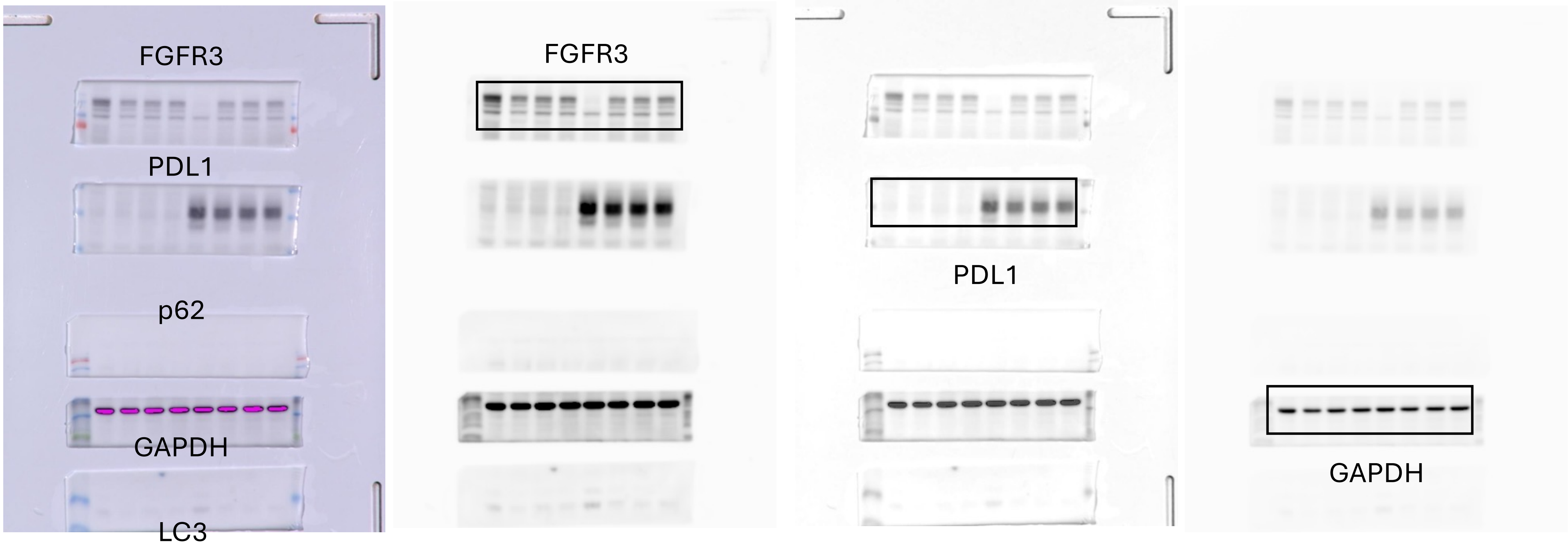

SubF1D RT-112 (Erda)

2022.08.26 n=2

Erda-FGFR3, PD-L1, p62, LC3B, GAPDH

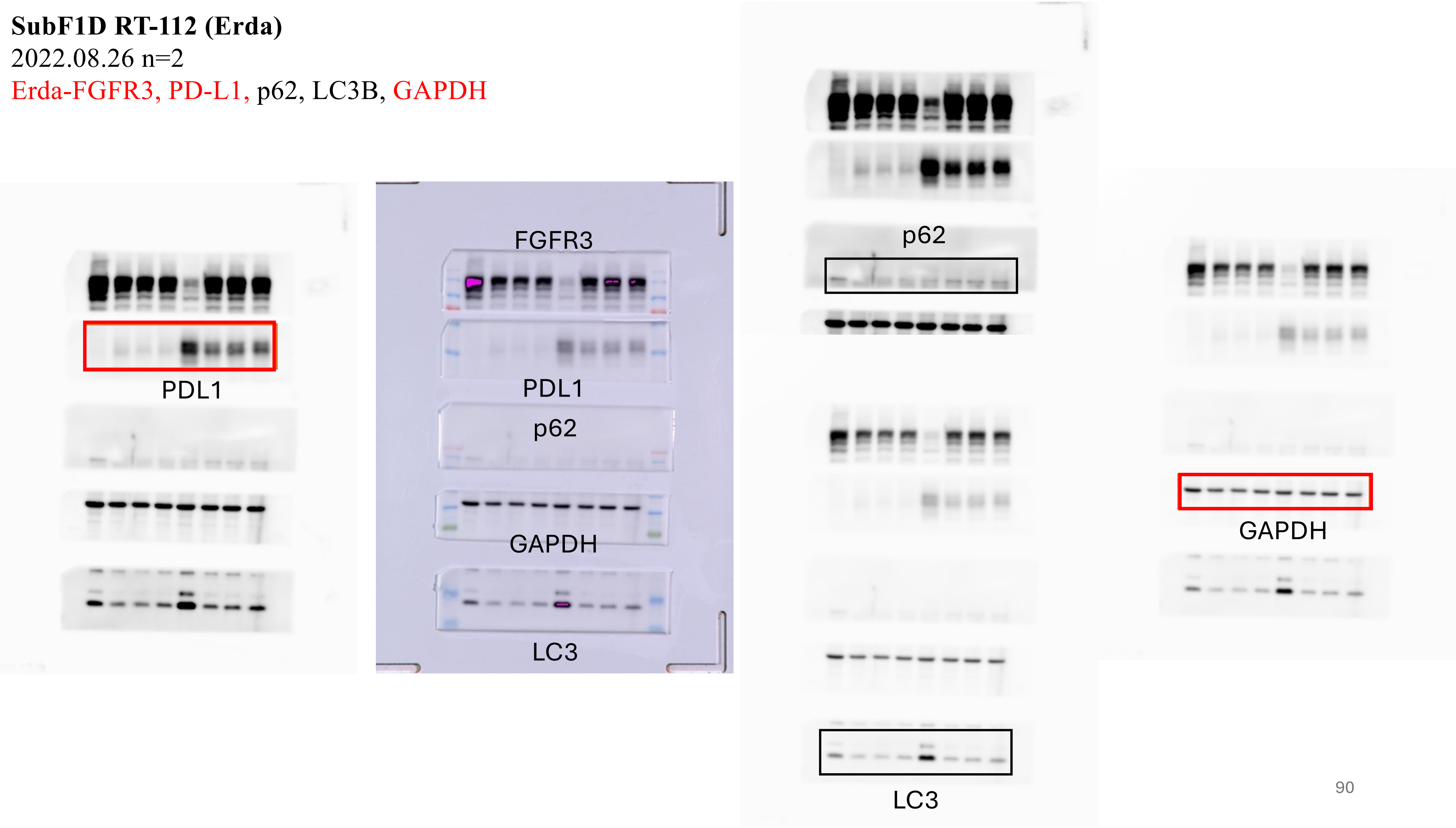

RT112 (Erda)

2022.9.16 n=3

FGFR3, PD-L1, GAPDH

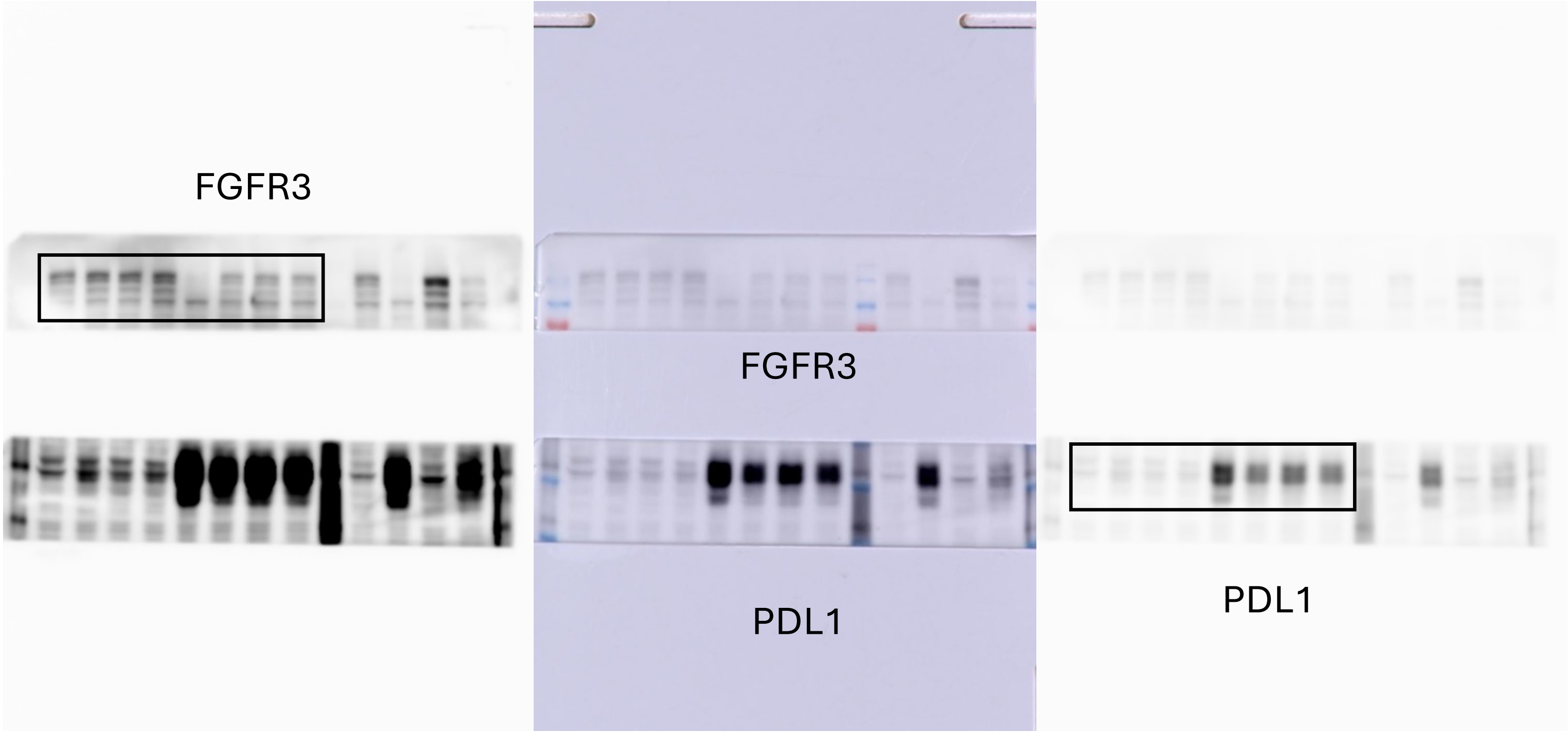

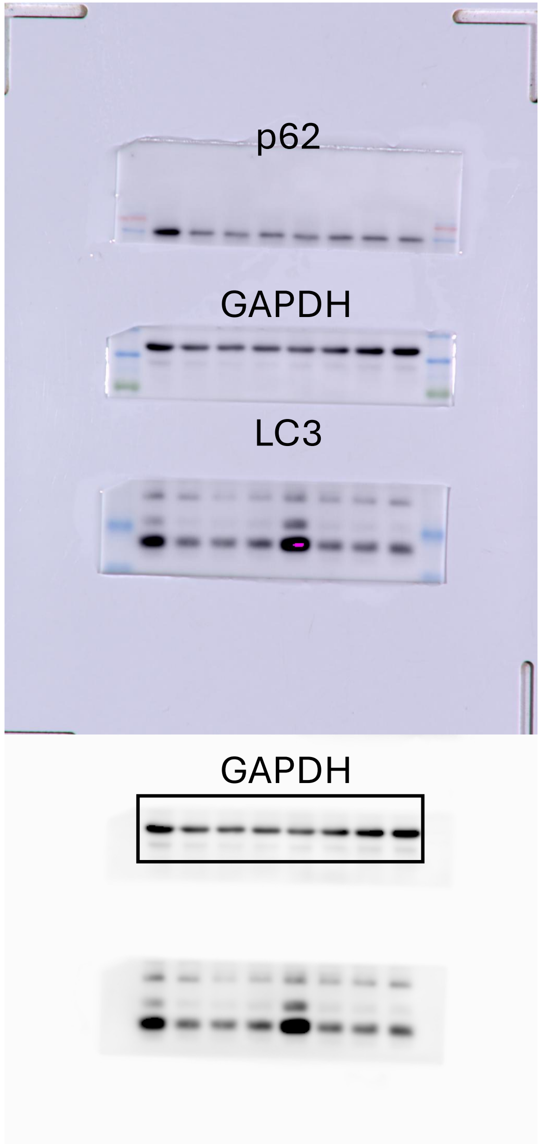

RT4 (Erda)

2022.7.13 n=1
Erda-FGFR3, PD-L1, GAPDH

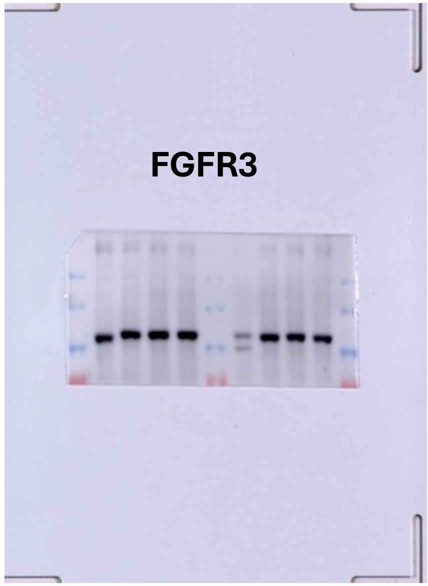

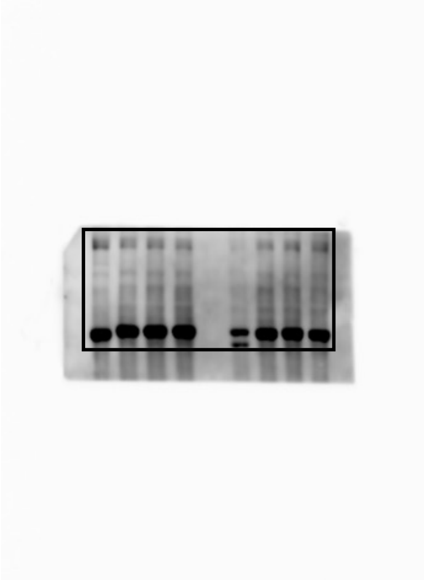

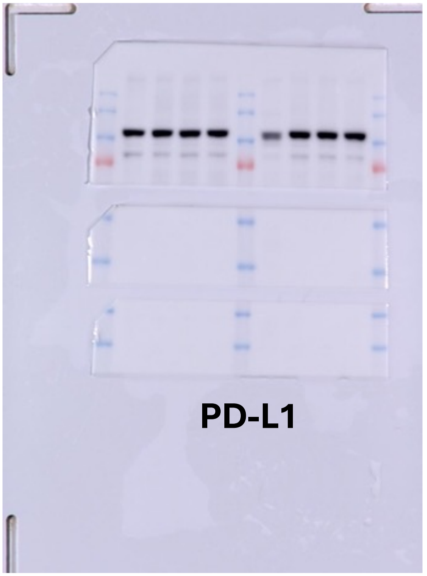

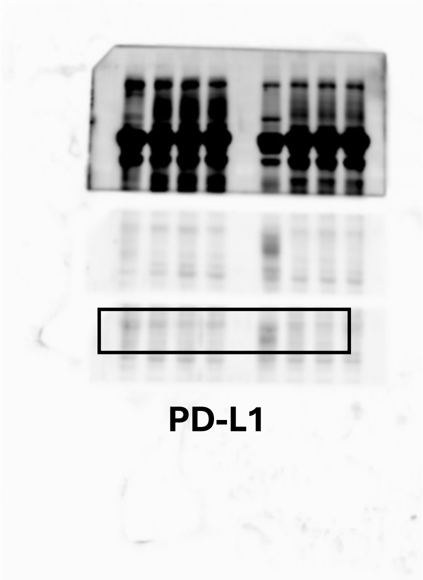

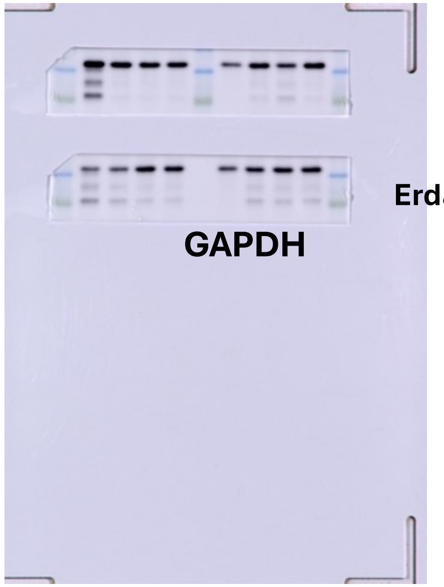

Erda

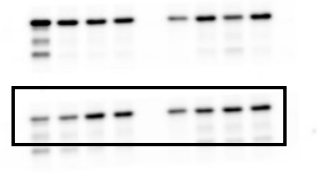

SubF1D RT4 (Erda)

2023.08.15 n=2

Erda-FGFR3, PD-L1, GAPDH

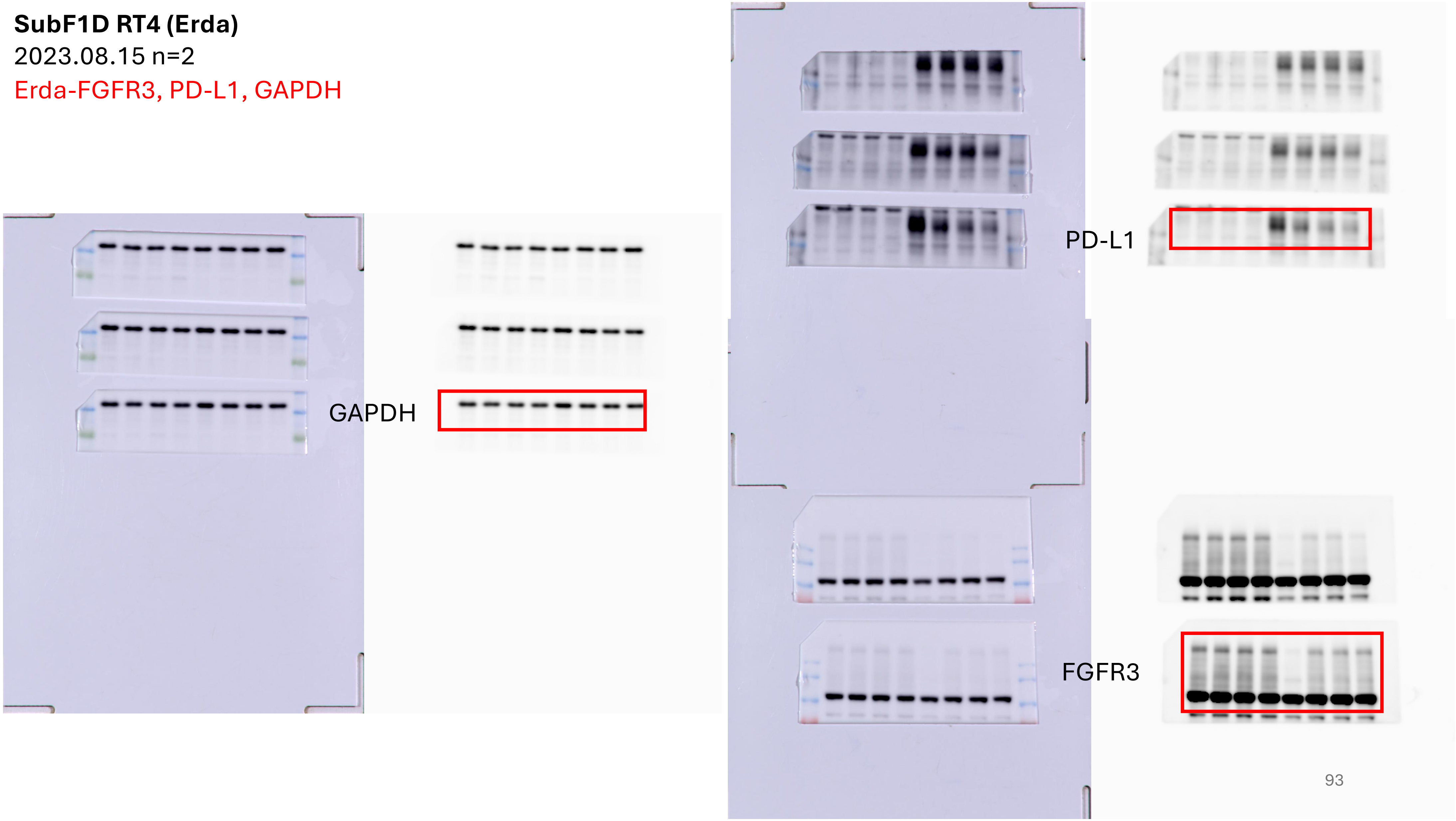

RT4 (Erda)

2022.9.14 n=3

FGFR3, PD-L1, GAPDH

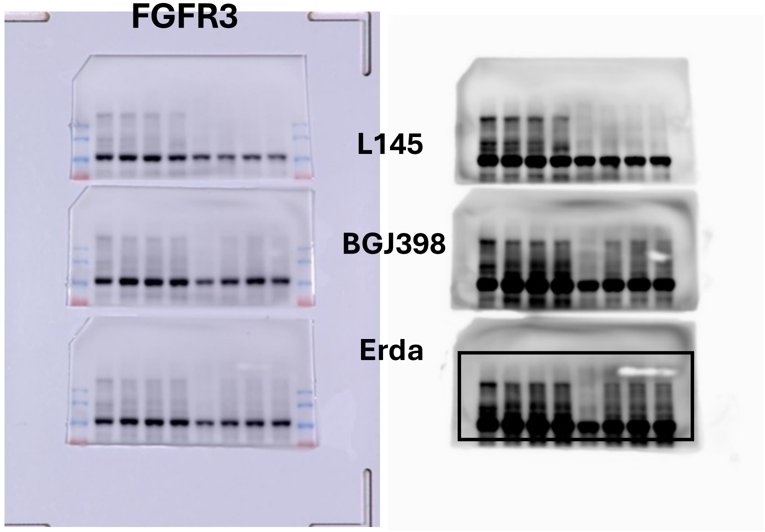

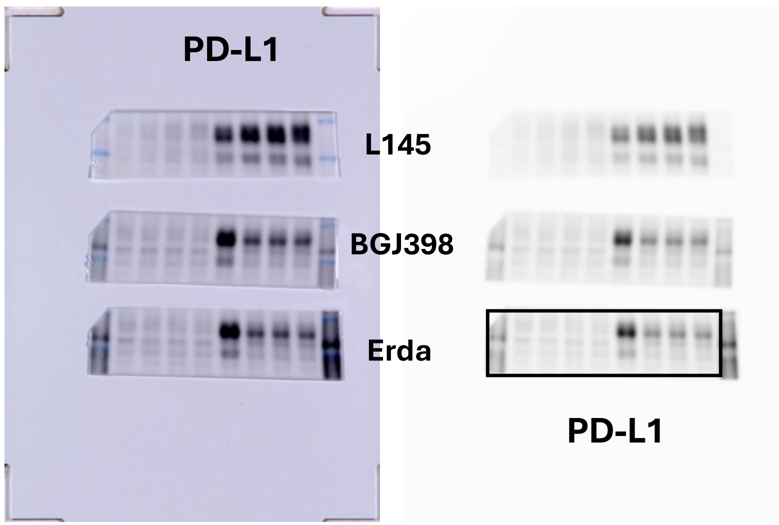

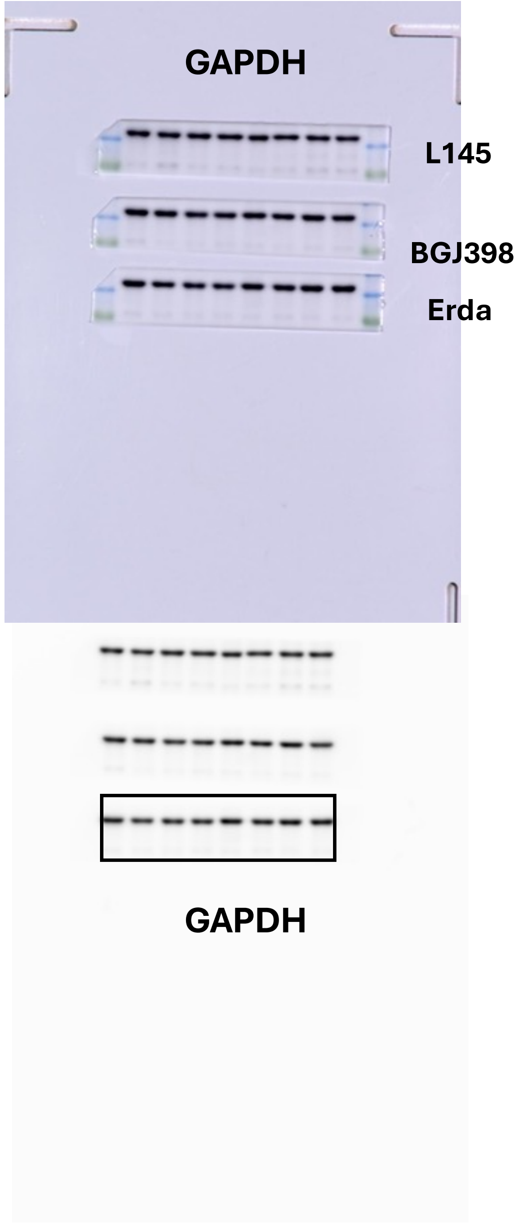

SubF1E RT-112 (Erda+CQ)

2023.01.13 n=1, 2

Erda-PD-L1, p62, LC3, GAPDH

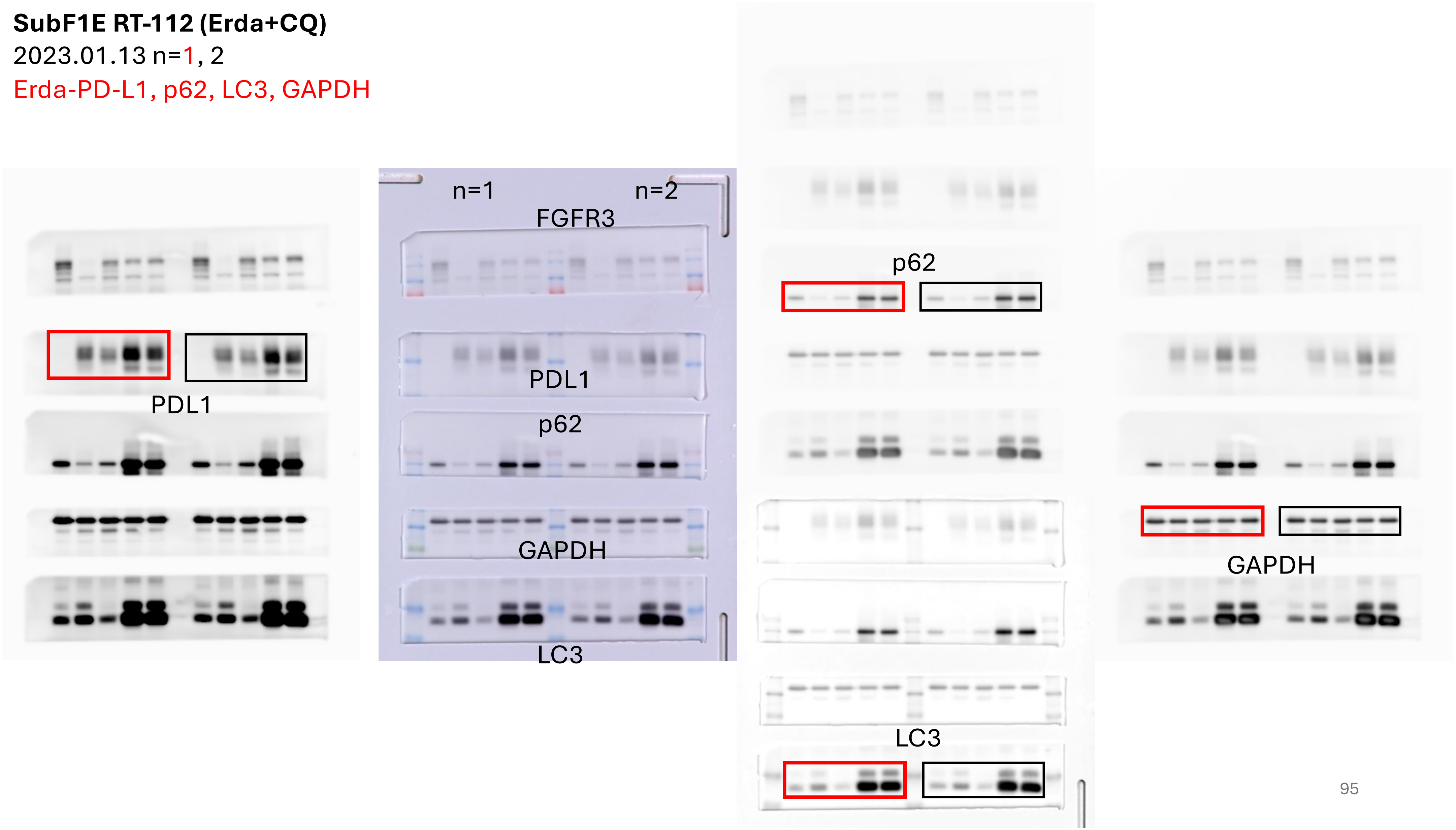

RT-112

2023.1.17 n=3
Erda-FGFR3, PD-L1, GAPDH

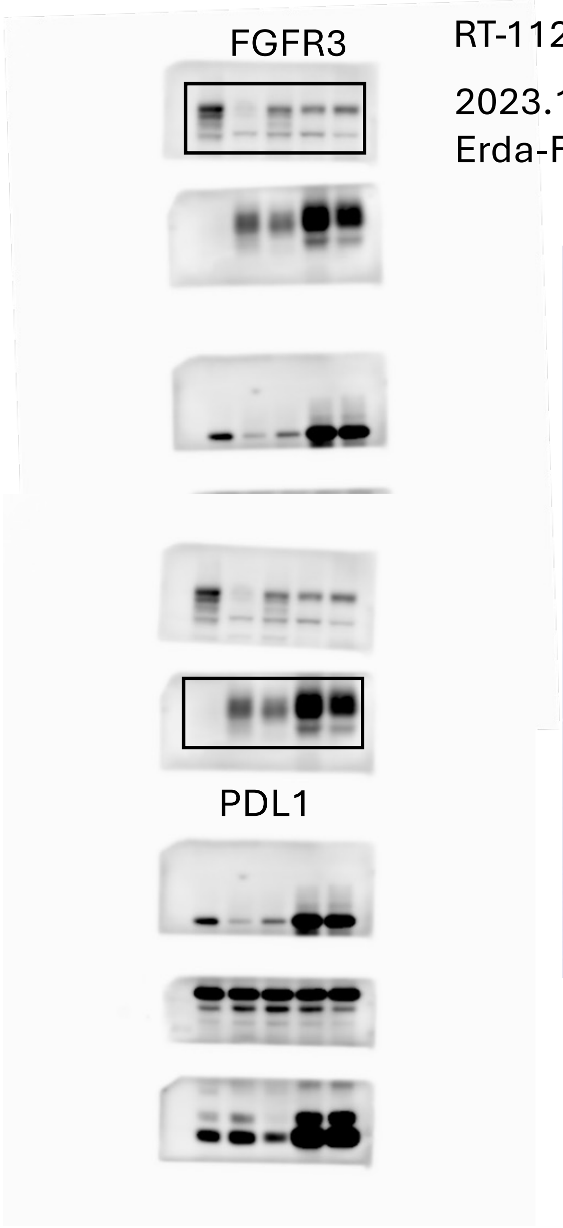

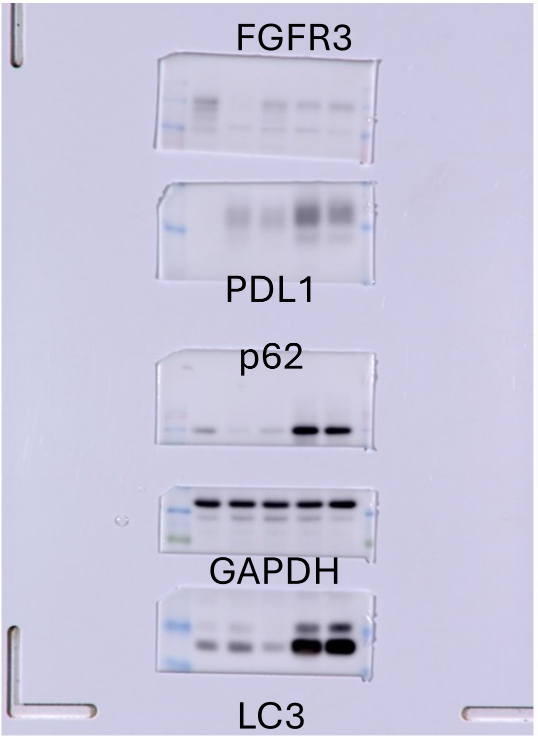

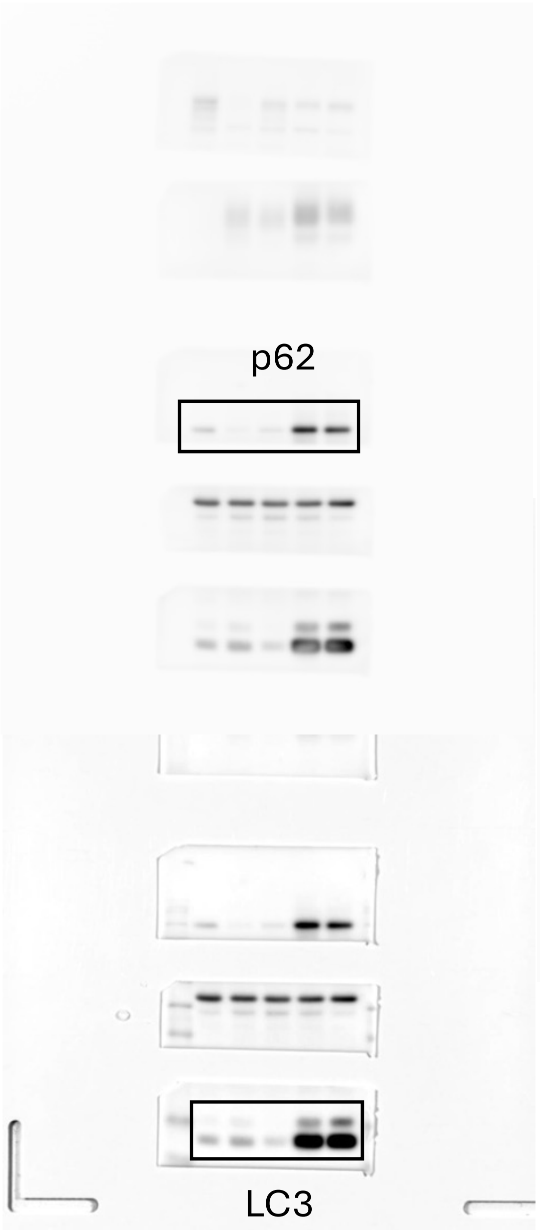

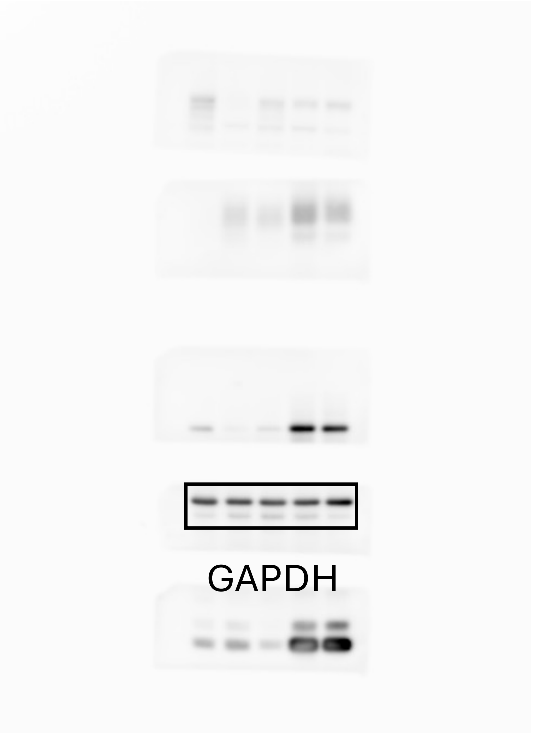

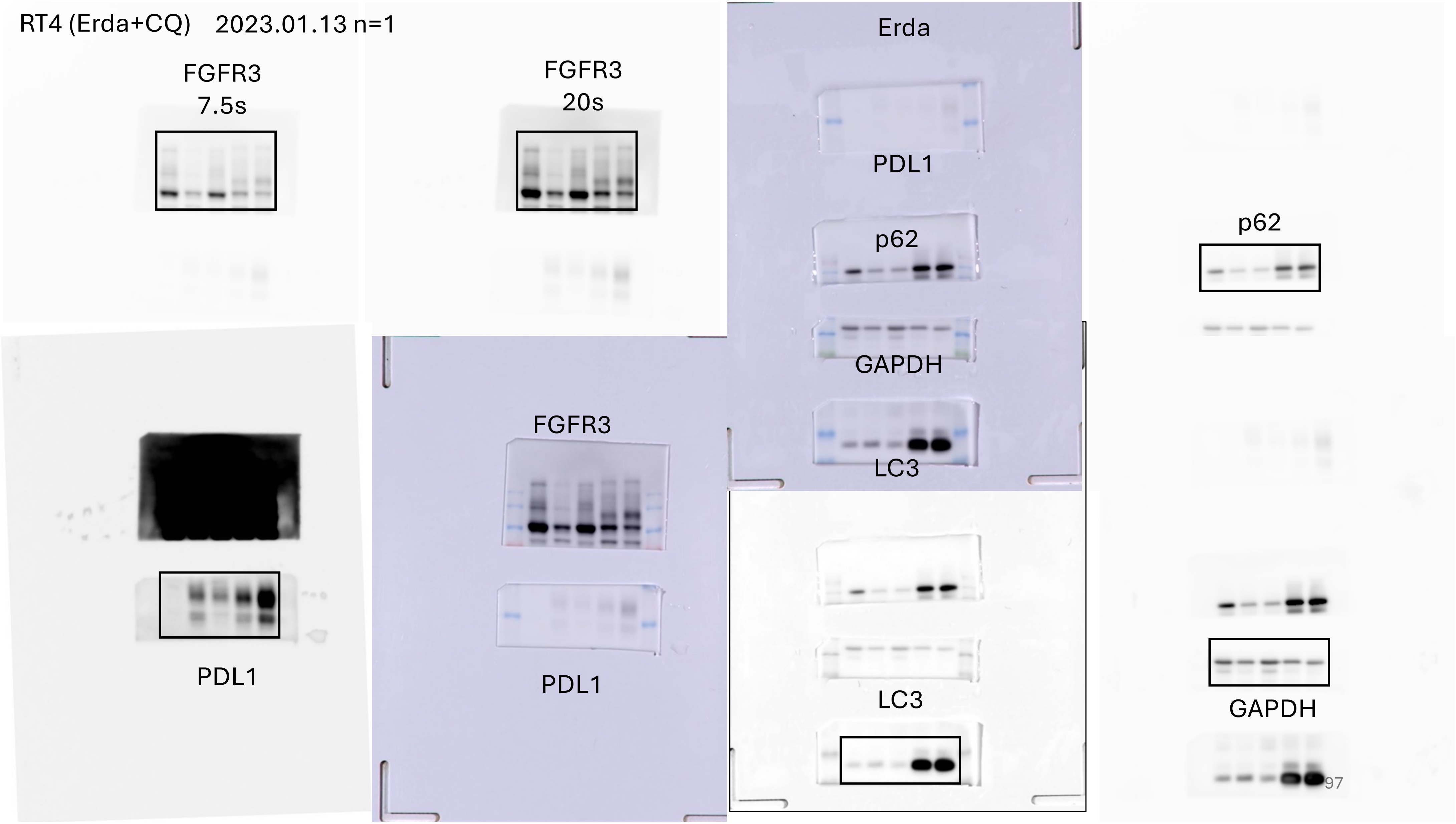
RT4 (Erda+CQ) 2023.01.13 n=1
FGFR3
7.5s
FGFR3
20s
Erda
PDL1
p62
GAPDH
LC3
p62
PDL1
FGFR3
PDL1
LC3
GAPDH

SubF1E RT4 (Erda+CQ)

2023.01.17 n=2, 3

Erda-PD-L1, p62, LC3, GAPDH

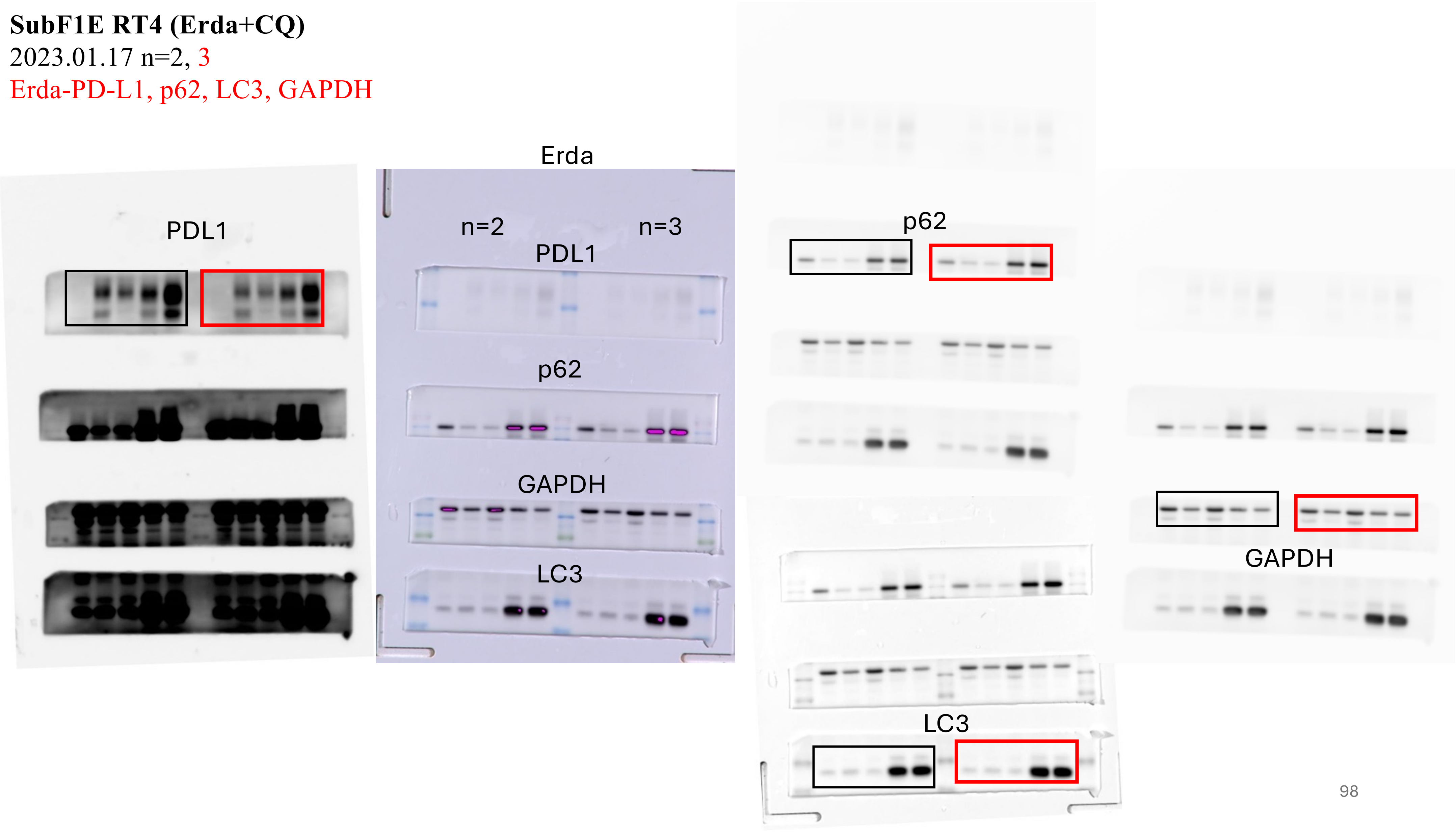

SubF2C RT112

2024.03.07 RT112 24 h

SIGMAR1, β-actin

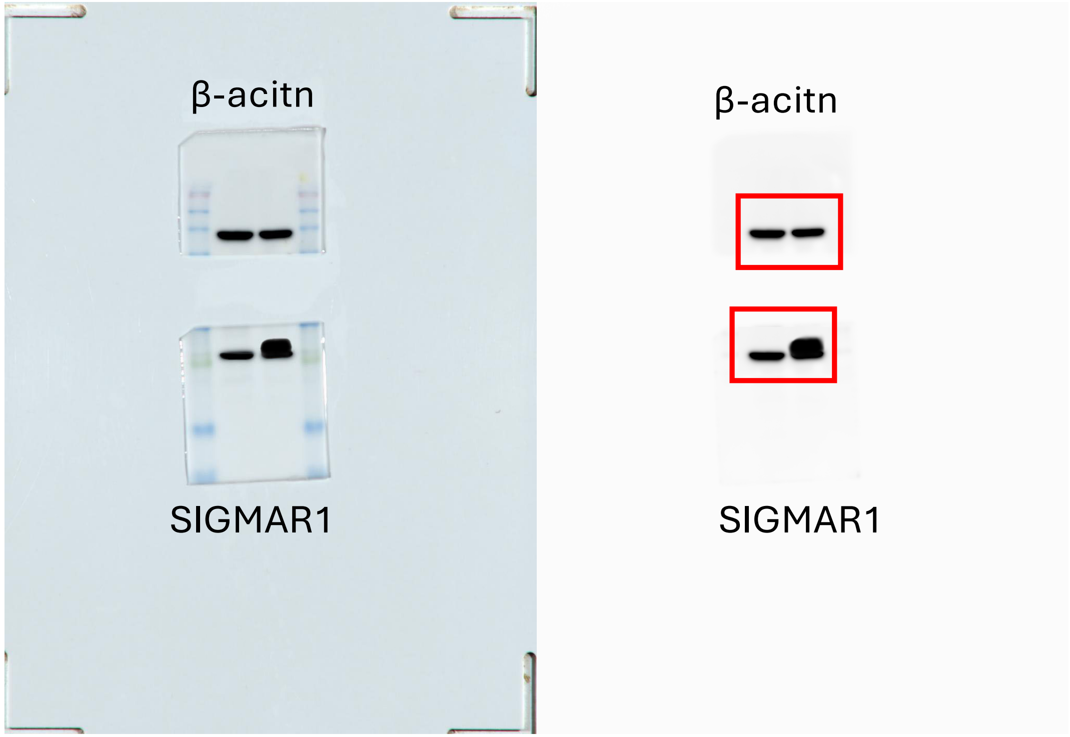

SubF3E RT112

2024.03.29 RT112 24 h n=1

GAPDH

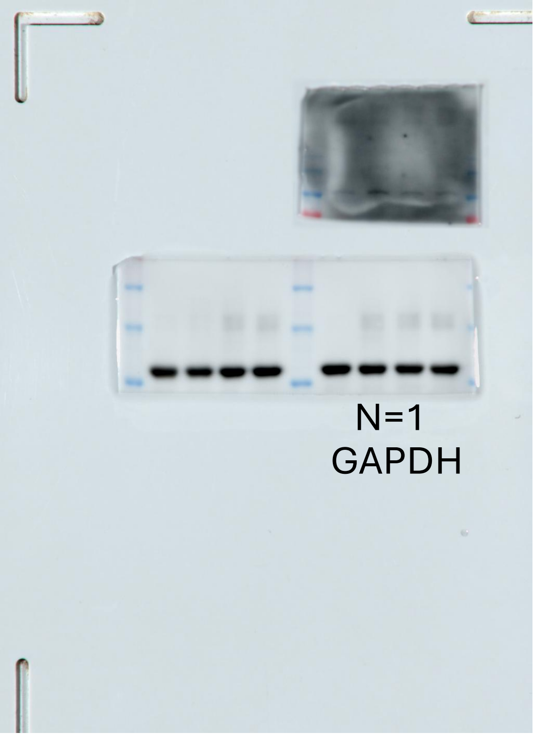

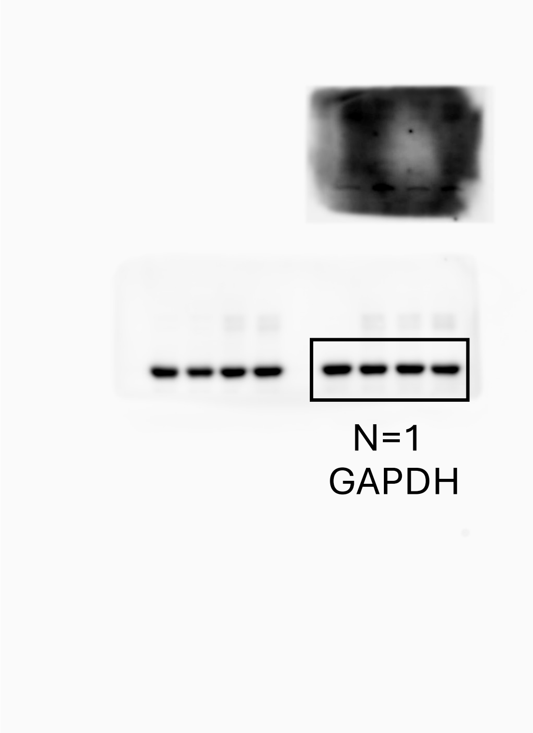

2024.04.04 RT112 24 h n=2, 3

GAPDH

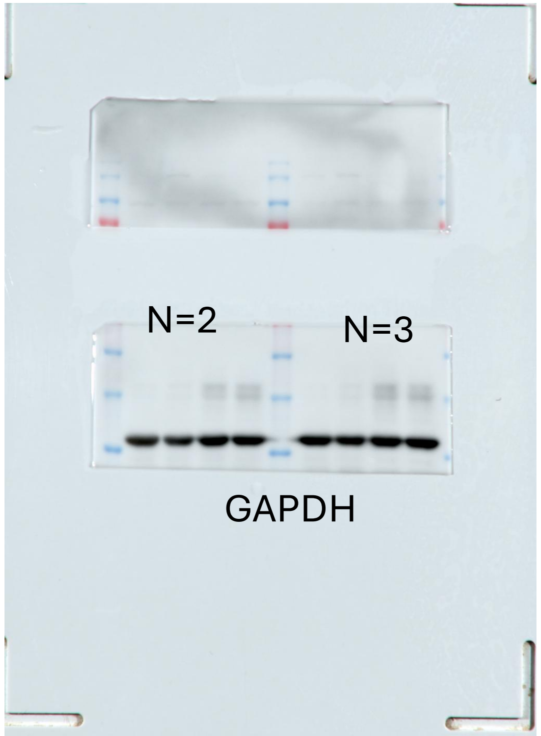

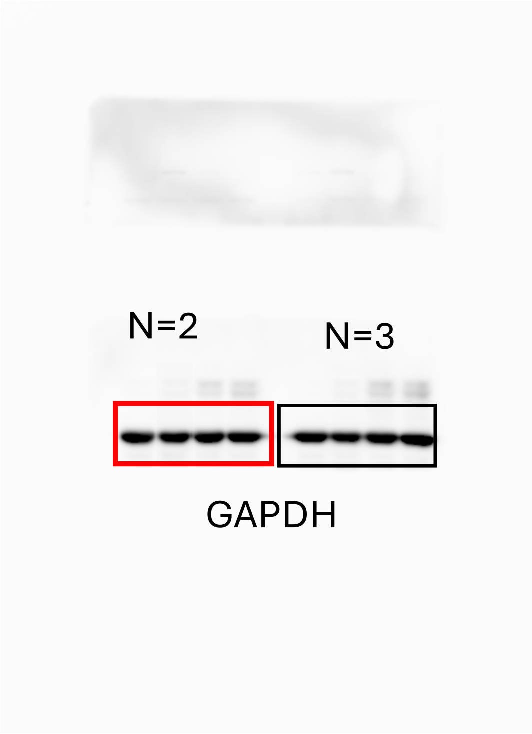

SubF3E RT112

2024.04.09 RT112 24 h n=1, 2, 3

CIITA

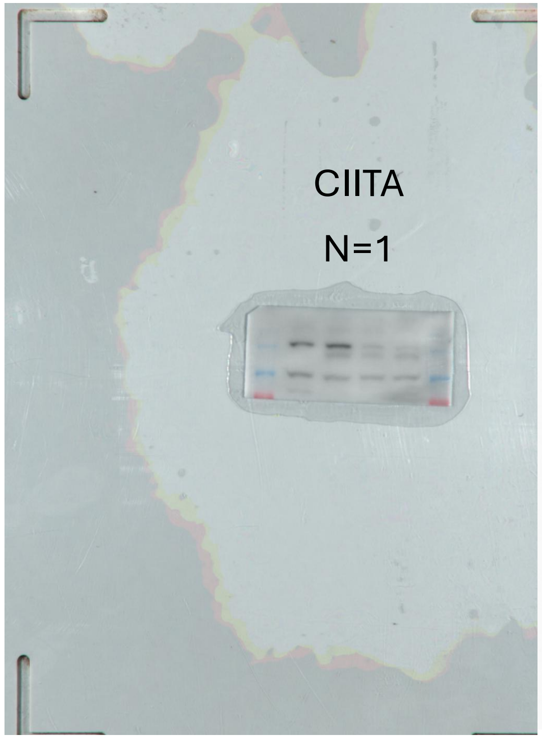

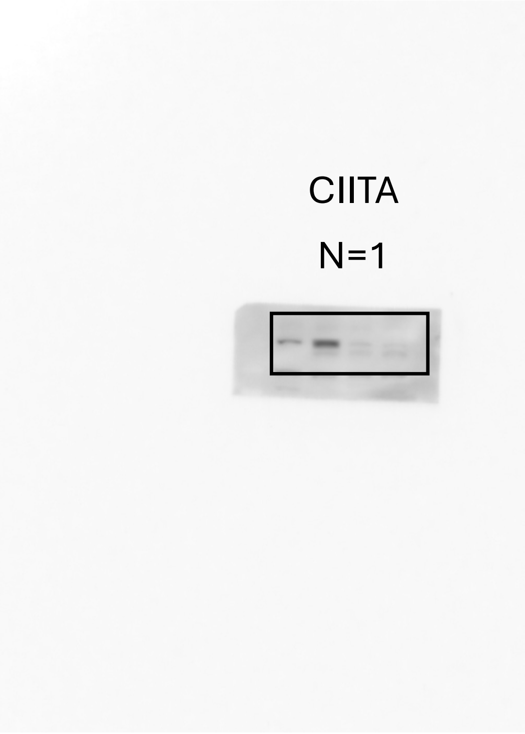

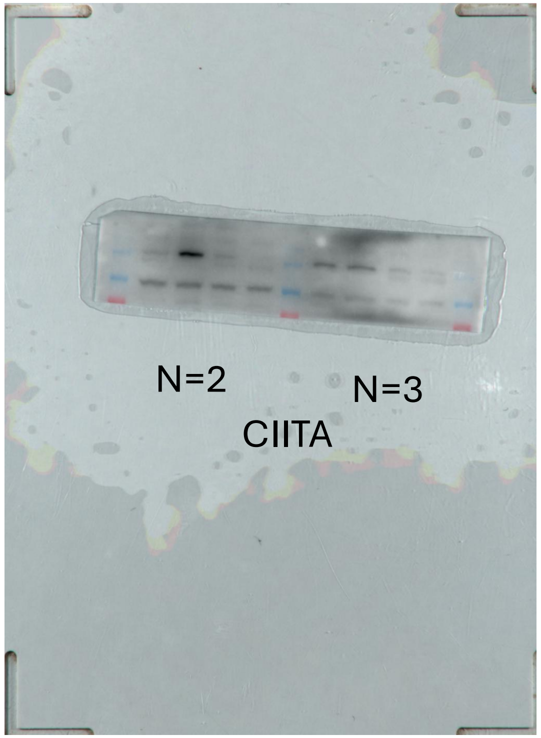

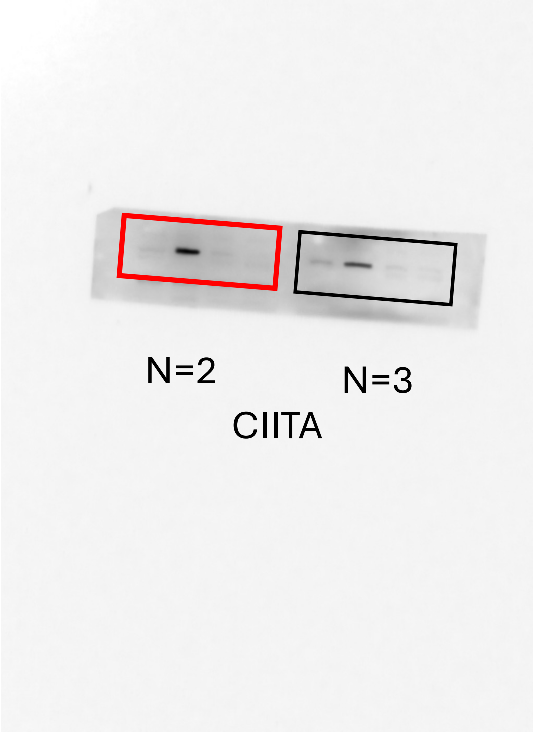

2024.03.22 RT112 24 h n=1
HistoneH3, (K4ac), (K27ac), GAPDH

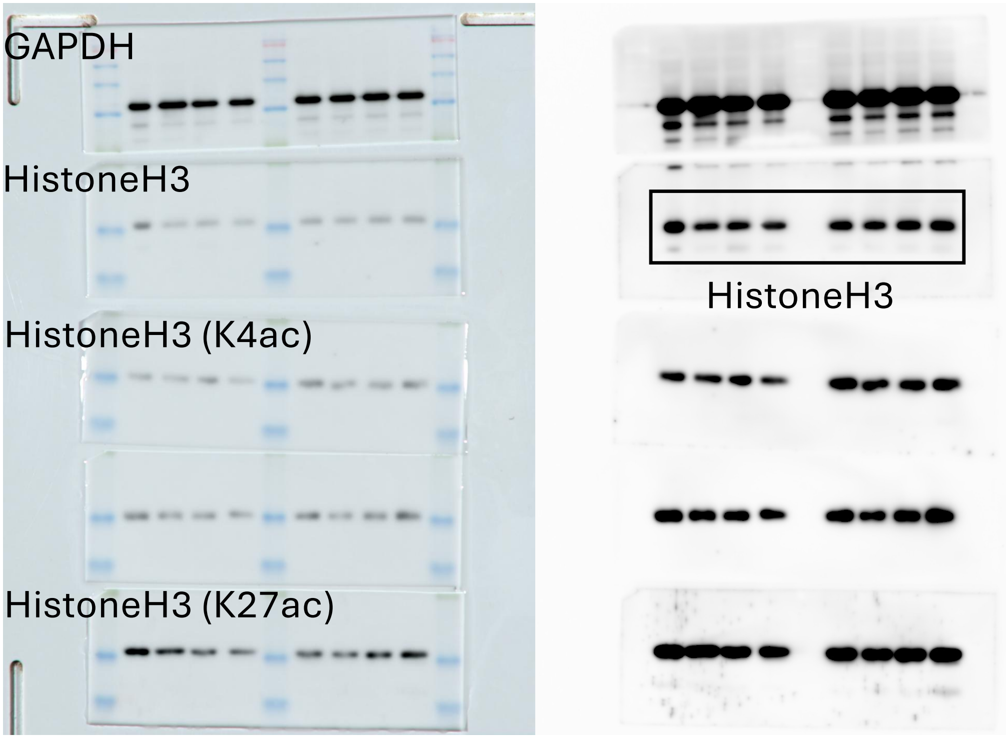

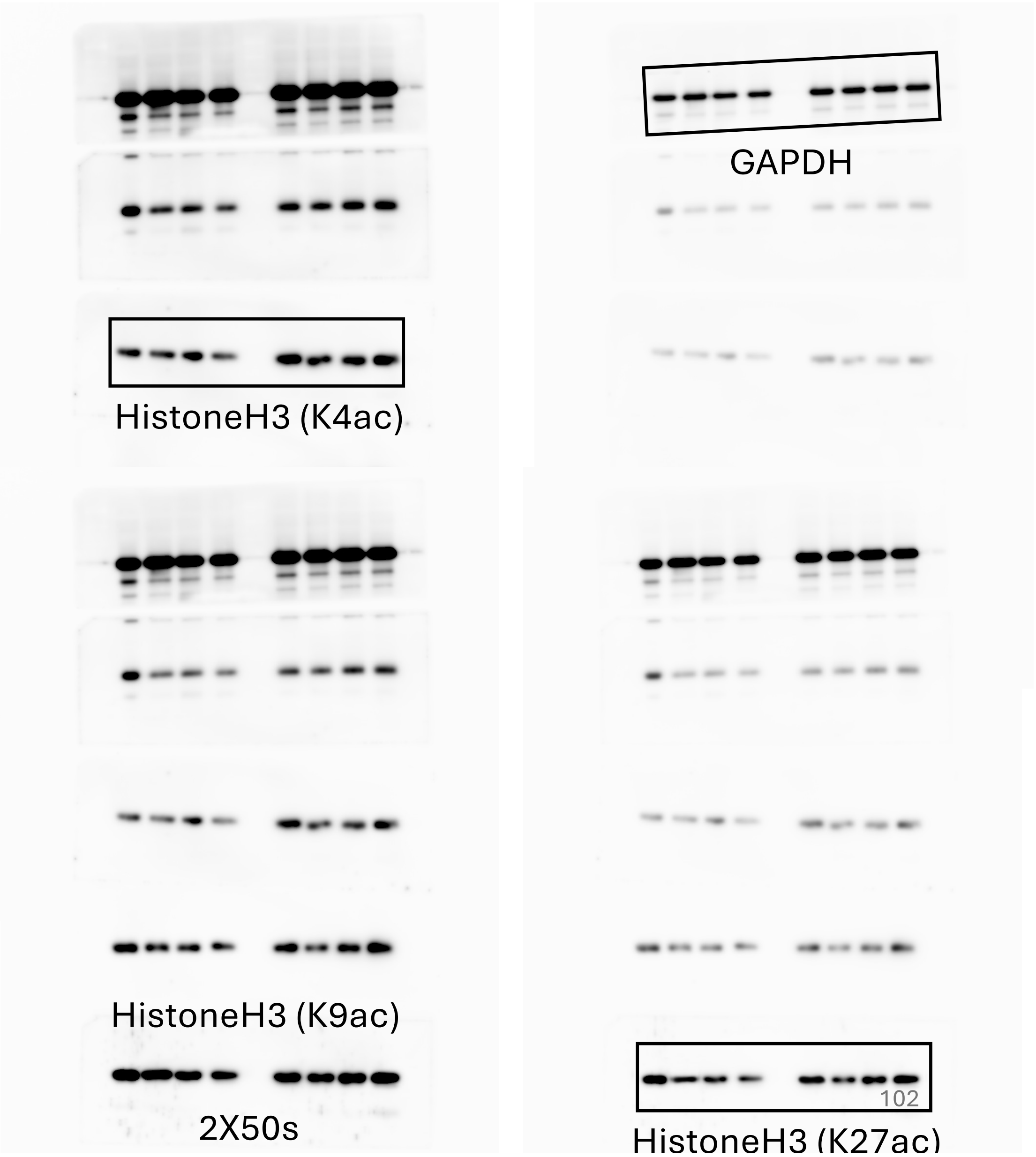

2024.03.26 RT112 24 h n=2
HistoneH3, (K4ac), (K27ac), GAPDH

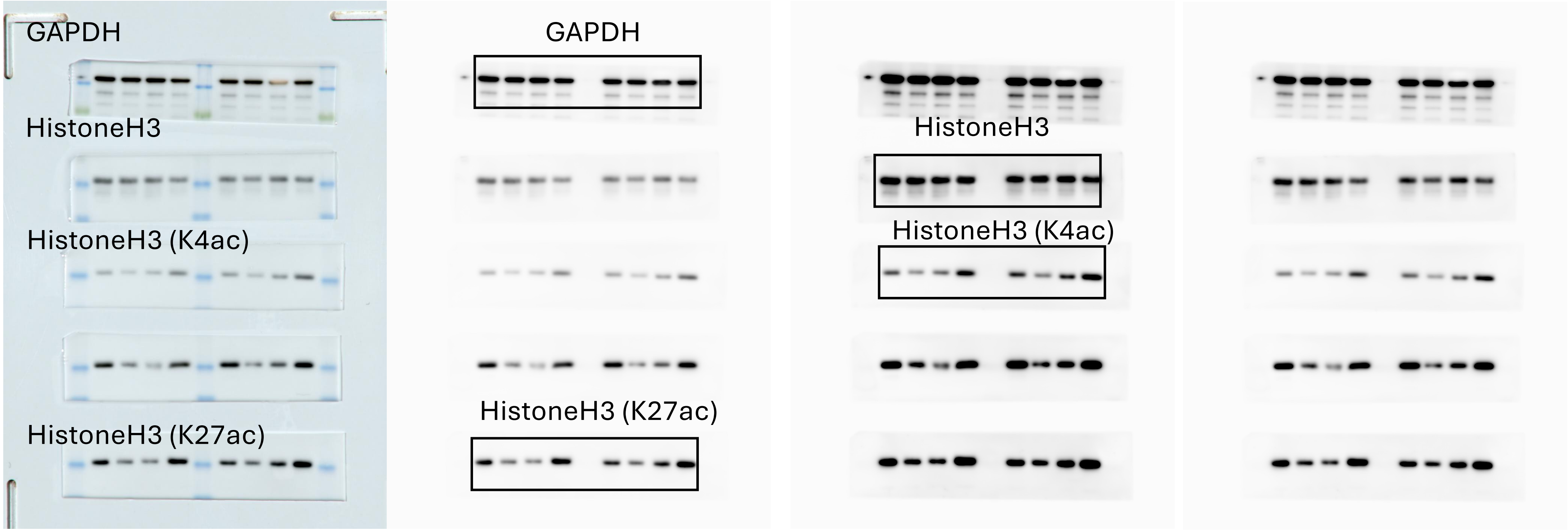

SubF3F RT112

2024.03.26 RT112 24 h n=3

HistoneH3, (K4ac), (K27ac), GAPDH

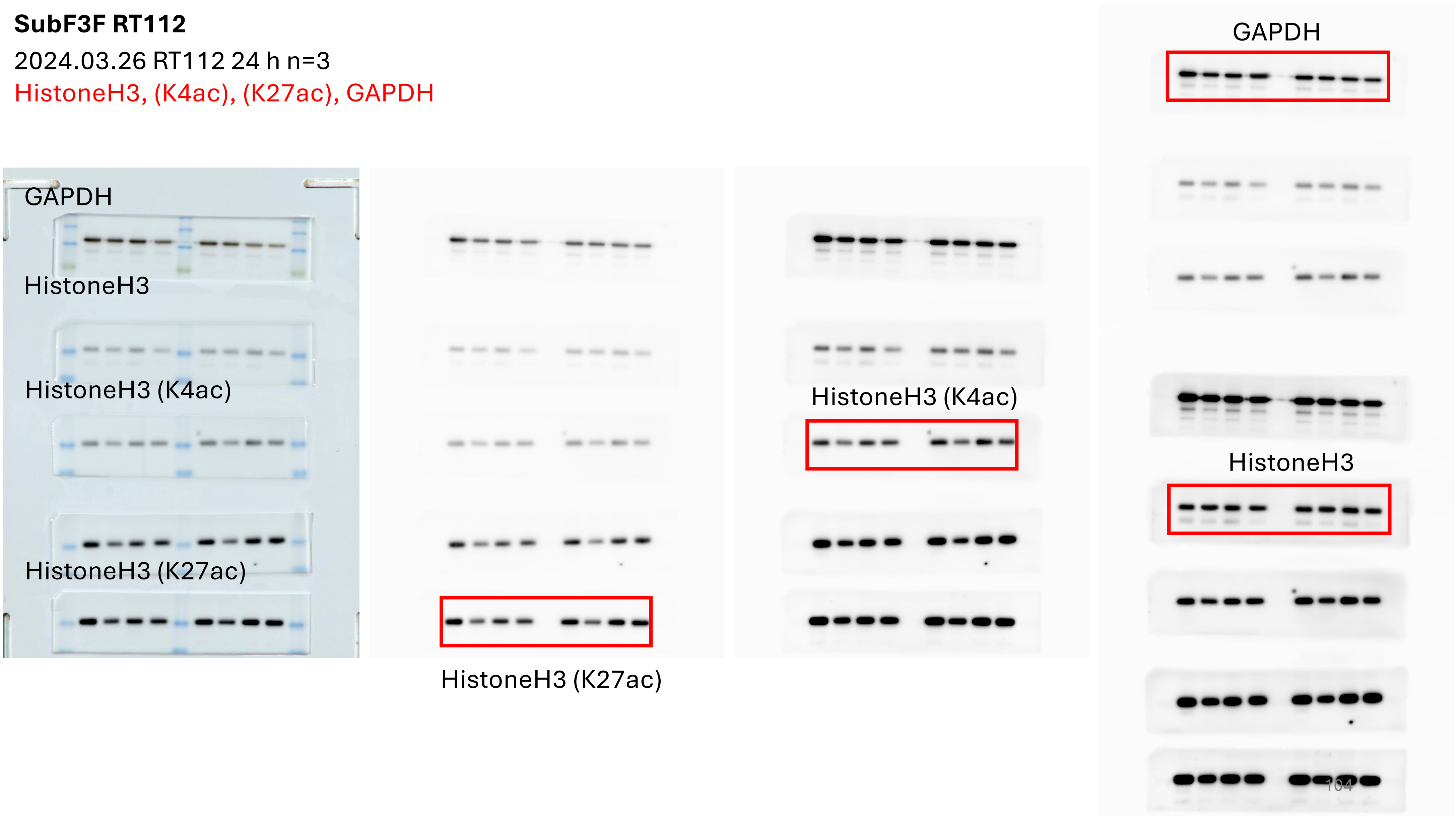

HistoneH3 (K27ac)

2024.03.28 RT112 24 h n=4
HistoneH3, (K4ac), (K27ac), GAPDH

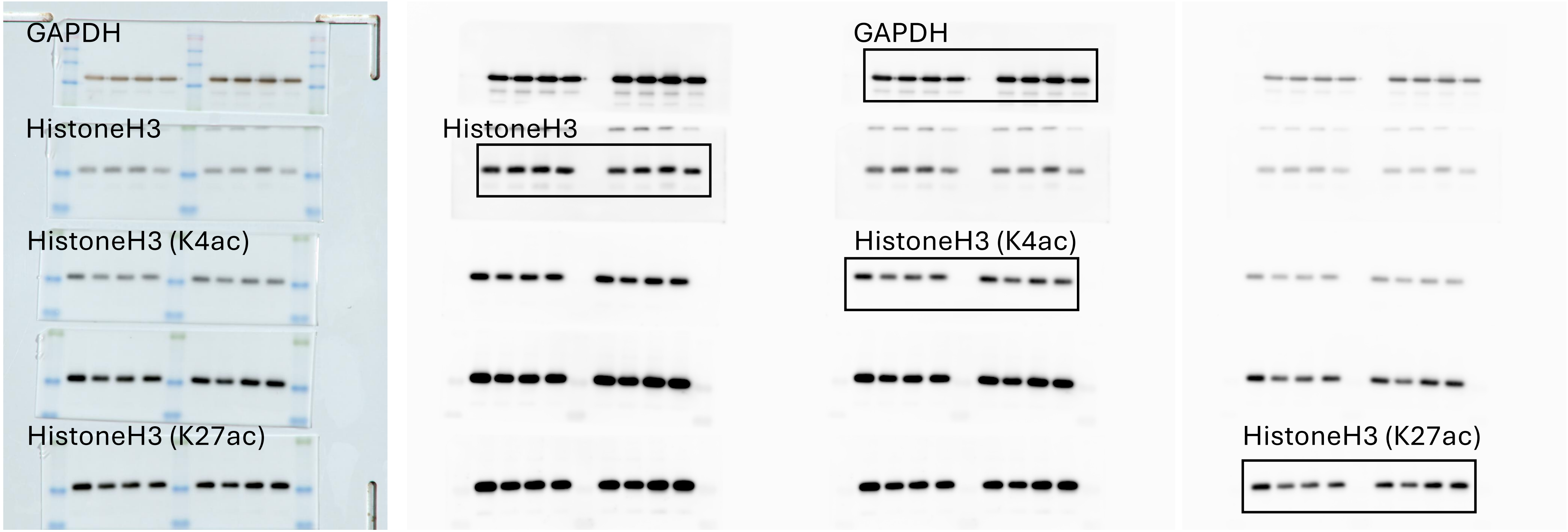

2024.04.02 RT112 24 h n=5
HistoneH3, (K4ac), (K27ac), GAPDH

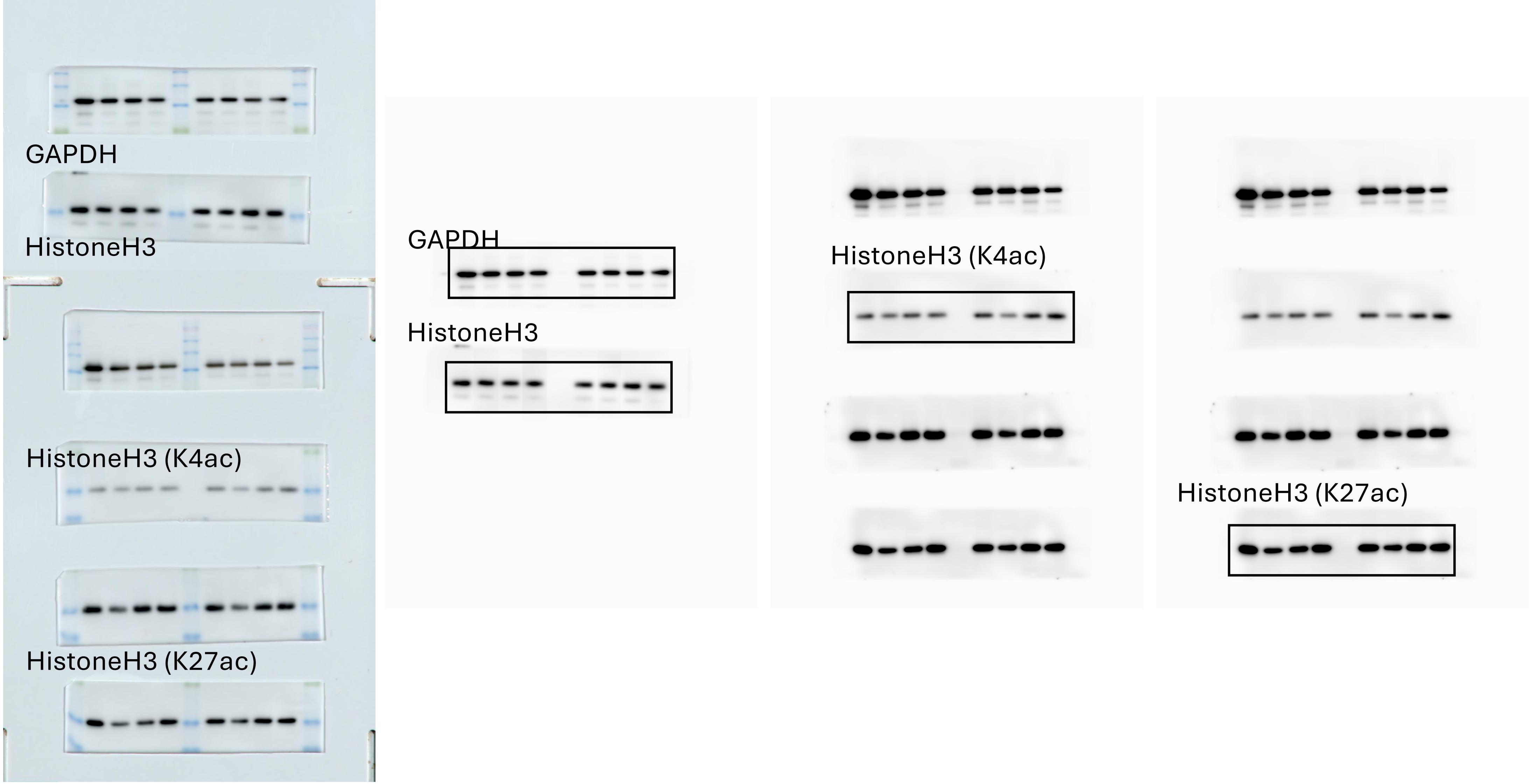

Supplement: Supplementary file 2 — Original WB data [file 41419_2025_7821_MOESM2_ESM.pdf]
